# A Systematic Review of Nonsugar Sweeteners and Cancer Epidemiology Studies

**DOI:** 10.1016/j.advnut.2025.100527

**Published:** 2025-09-30

**Authors:** Denali Boon, Satori A Marchitti, Kyle J Colonna, Ilkania M Chowdhury-Paulino, Wenchao Li, Axel Berky, Catalina Restrepo, Maia Jack, Julie E Goodman

**Affiliations:** 1 Gradient, Boston, MA, United States; 2 Geosyntec, 2501 Blue Ridge Road Suite 430 Raleigh, NC 27607, United States; 3 American Beverage Association, Washington, DC, United States

**Keywords:** nonsugar sweeteners, sweeteners, aspartame, sucralose, acesulfame potassium, cancer, humans, epidemiology, systematic review

## Abstract

Nonsugar sweeteners (NSSs) are added to foods and beverages to provide sweetness in place of sugar while reducing the total caloric content. Reducing sugar intake, and corresponding calories, may decrease the risk of diabetes and other health conditions associated with obesity (e.g., cancer). Numerous observational epidemiology studies have evaluated the effect of NSSs on cancer risk, sometimes focusing on a specific NSS or a specific cancer, other times focusing on all NSSs or all cancers. We conducted a systematic review of epidemiology studies of NSS intake (of all types in aggregate and individually) and the risks of all types of cancer published through Fall 2024 [preregistered with Open Science Framework (https://osf.io/gc8v6)]. We considered how major study quality concerns might have impacted the interpretation of individual study results, as well as the evidence as a whole. We identified 90 studies of acesulfame potassium (ace-K), aspartame, cyclamate, saccharin, sucralose, or nonspecific NSSs in aggregate [e.g., diet sodas, artificially sweetened beverages], and 17 specific types of cancer. We found no consistent associations between any NSS or NSSs in aggregate and any cancer overall, and no evidence for dose–response. NSS intake information was always self-reported, rendering exposure misclassification an ongoing challenge in all studies, and recall bias remains a significant possibility in all case-control studies. Many studies also did not fully account for potential confounders. Experimental animal and mechanistic evidence for NSSs does not support human-relevant carcinogenicity or any biologically plausible mechanisms by which NSSs could cause genotoxicity or cancer in humans. Overall, the epidemiology evidence does not support associations between any NSS and any cancer type.

**Statement of Significance**

We conducted a systematic review of epidemiology studies of nonsugar sweetener (NSS) intake (of all types in aggregate and individually) and the risks of cancer (of all types) published through Fall 2024. After an evaluation of the results, consideration of study quality factors and their impact on the interpretation of results, and integration of animal evidence, we found no association between NSS generally, or any individual NSS and cancer.

## Introduction

For nearly half a century, nonsugar sweeteners (NSSs) have been added to foods and beverages to provide sweetness in place of sugar while reducing the total caloric content [1]. By reducing sugar intake, NSSs may contribute to a decrease in diabetes and other health conditions associated with obesity. The NSSs, acesulfame-K, aspartame, neotame, saccharin, sucralose, advantame, stevia (including steviol glycosides), Luo Han Guo monk fruit extracts, and thaumatin, are approved in the United States, whereas the European Union (EU), in addition to many of these, has also approved the use of cyclamate and neohesperidine DC [2].

Numerous observational studies have evaluated the association between most of these NSSs and different cancers, and many of these studies have been reviewed in the last few years. Two recent reviews of all NSSs and all cancer types were conducted by WHO. In 2018, WHO commissioned a systematic review to evaluate studies of NSSs and risks of several health conditions, including cancer, published through 2017 [3]. Toews et al. [3] did not find "compelling evidence" of an association between NSSs generally and adverse health outcomes, including cancer. The largest group of studies they reviewed were on bladder cancer. They reported no increased risk of bladder cancer in a meta-analysis of case-control studies comparing NSS consumers to nonconsumers. They reported decreased risks of ovarian and pancreatic cancers with NSS consumption, but this was based on only a single study of each cancer type. The only specific NSS they reported on was aspartame. They reported no association with brain cancer in children based on a single study, and no associations with hematologic cancers in adults. They noted observational studies of NSSs and health effects, including cancer, should be "interpreted with caution" due to the high likelihood of significant methodological limitations including confounding and the potential for reverse causality [3].

WHO updated and expanded on this review and included studies of NSSs published through July 2021 [4]. In contrast to Toews et al. [3], Rios-Leyvraz and Montez [4] concluded that there was an association between NSSs and bladder cancer in case-control studies, and that it was likely due to tabletop use of saccharin, but the subgroup analyses were not significant and the authors deemed the certainty of evidence to be "very low" [4]. They reported an increased risk of laryngeal and nonobesity-related cancers, and a decreased risk of lung and ovarian cancers, associated with NSS consumption, but noted the small number of studies that evaluated each of these cancers. They reported that no other results were statistically significant and, similar to Toews et al. [3], concluded that the certainty of evidence across all of the included studies on cancer mortality or incidence was "very low" [4]. Also similar to Toews et al. [3], Rios-Leyvraz and Montez [4] focused their evaluation on NSSs combined (i.e., there were limited evaluations of specific NSSs).

Although study quality was evaluated in both the Toews et al. [3] and Rios-Leyvraz and Montez [4] reviews, these evaluations were not entirely transparent, and the bearing of study quality issues on the interpretation of study results was not always clear. As has been discussed previously by Goodman et al. [5], both WHO reviews used standard frameworks to evaluate risk of bias and study quality (i.e., ROBINS-I [Risk Of Bias In Non-randomized Studies of Interventions], Newcastle-Ottawa Scale, and GRADE [Grading of Recommendations Assessment, Development, and Evaluation]). Both reviews indicated that the overall evidence was of "very low" certainty or that the risk of bias in all of the studies reviewed was "serious." However, the rationale for the assessments was not transparently documented. Rather, the assessments were limited to numeric scores or categorized ratings without context or discussion of what factors within a study influenced those assessment conclusions or how the quality or potential for bias in specific domains were more or less likely to have a substantial impact on overall study quality or the interpretation of the results.

Building on these prior assessments, we conducted a systematic review of epidemiology studies that evaluated the relationship between any NSS and any type of cancer published through Fall 2024. Although we comprehensively evaluated study quality (i.e., issues with participant selection, the NSS intake assessment, the outcome evaluation, confounding, and temporality), we focused our analysis on the study quality domains that were most likely to impact the interpretation of NSS-specific results (i.e., NSS intake assessment, confounding/covariate consideration, outcome assessment, and selection bias). That is, we considered how any major study quality concerns might have biased individual study results and their interpretation, as well as the evidence for specific NSSs and NSSs in aggregate and each cancer outcome.

## Methods

The protocol for this systematic review was registered with Open Science Framework on 29 September 2023 (https://osf.io/gc8v6).

### Literature search

We identified individual studies using the following population, exposure, comparator, outcomes, and study design (PECOS) statement:

In the general human population (P), what is the risk of having or dying of cancer (O) among those who consume NSS (E1) compared with those who do not (C1), or for each NSS unit consumed (e.g., drink/day) (E2) compared with less consumption (C2), as observed in primary observational studies with data at the individual level (S)?

Corresponding inclusion and exclusion criteria are shown in Table 1. Literature searches were conducted using Scopus and PubMed to identify relevant studies published through 6 November 2024. Search terms are listed in Supplement A, Supplemental Table A.1. We also reviewed the reference lists of relevant studies, reviews, and meta-analyses to identify additional studies that met our inclusion criteria.

**TABLE 1:** PECOS elements and corresponding inclusion and exclusion criteria

PECOS element	Inclusion criteria	Exclusion criteria
Population	• General population, children or adults	• Cohort studies: individuals with cancer at baseline
Exposure Comparator	• Nonsugar sweeteners • No or lower exposure to nonsugar sweeteners or different sweetener than one of interest	• High calorie sweeteners • Exposure contrast not given
Outcomes	• Any type of cancer	• Noncancer outcomes
Study design	• Cohort • Case control • Cross-sectional	• Reviews, meta-analyses, commentaries, book chapters, or conference abstracts • Ecologic studies • Case studies/series • Animal or in vitro studies • Methodological studies • Studies with secondary analyses only (e.g., based on risk estimates from existing studies) • Not published in English • No results presented

Abbreviations: PECOS, Population, Exposure, Comparator, Outcomes, Study Design.

In cases where multiple eligible studies of the same population and outcome were noted, we focused on the most recent study or the study reporting the most informative data (e.g., greater population coverage, more reliable exposure, confounder, and outcome estimates, and/or longer duration of follow-up).

### Study selection and data collection

Titles, abstracts, and full article texts (when needed) of the relevant studies identified from the systematic literature search were independently screened by two reviewers and any discrepancies were resolved by a third reviewer. Eligible studies were selected based on the PECOS statement and corresponding inclusion and exclusion criteria; noneligible studies were excluded and the reasons for exclusion were documented. The study screening process was managed using the systematic review software tool, Covidence (https://www.covidence.org/).

Two reviewers reviewed each included study. The first reviewer independently extracted data (i.e., study characteristics and study results) and evaluated study quality; the second reviewer confirmed that the extracted data and study quality evaluation were accurate. Any disagreements between the reviewers were noted and resolved through discussion, or by a third reviewer when necessary. Collected data were stored and organized in Word or Excel tables.

If a study reported multiple exposure–outcome pairs of interest, each exposure–outcome pair was recorded as a separate record. If multiple effect estimates were reported for a single exposure–outcome pair, only the most adjusted one was extracted, unless the purpose of the most adjusted model was to evaluate potential mediation, effect modification, or sensitivity of the main study result, in which case the main study result was extracted. In addition, when subgroup effect estimates were available, we extracted those data, as well.

For each study, the following study characteristics were extracted: citation (first author and year), study design (i.e., cohort and case-control), location, population characteristics (e.g., age, sex, and sample size), sources of cases and controls, exposure [period, NSS (e.g., nonspecific and aspartame)], NSS source (e.g., beverages and tabletop packets), exposure ascertainment (e.g., food frequency questionnaire and 24-h dietary records), and cancer outcome [cancer type, incidence/mortality, method of ascertainment, follow-up period (cohort), and study period (case-control)]. In addition, the following information related to study results were extracted: risk metric (e.g., risk, hazard, or odds ratios), reference group, exposure group(s), number of exposed cases, number of expected cases or exposed noncases, risk estimate and 95% confidence interval (CI), *P*-trend, and covariates controlled for. Results for outcomes with fewer than three studies or aggregate cancer outcomes (e.g., all lymphohematopoietic cancers) are not discussed in the text but are included in tables for completeness. Studies were grouped by study design (i.e., cohort or case-control) and cancer type evaluated.

### Study quality evaluation

Before evidence synthesis, we evaluated study quality across several domains: NSS intake assessment, covariate consideration and confounding, outcome assessment, and selection bias. We focus on the domains most likely to impact the interpretation of results [6] (i.e., NSS intake assessment, confounding/covariate consideration, outcome assessment, and selection bias) to identify study strengths and any threats to validity, and to determine how reliable the results of each study are for addressing the research question. We based our study quality criteria on several frameworks, including the Newcastle-Ottawa Scale [7], the ROBINS-I tool [8], the National Toxicology Program, Office of Health Assessment and Translation Risk of Bias Rating Tool for Human and Animal Studies [9], and the United States Environmental Protection Agency Office of Research and Development Staff Handbook for Developing Integrated Risk Information System Assessments [10]. For each study included in our review, specific aspects of study quality (falling into one of the five domains) were classified as higher or lower quality. Criteria for quality classifications are described briefly below and summarized in Supplement A, Supplemental Table A.2. Studies meeting the criteria for higher quality—a relative term—for a given attribute are recorded as "Y." When a factor did not meet the criterion for higher quality or information on this factor was not reported, an "N" was recorded. These assessments are intended to present a high-level perspective on study quality. More detail on the impact of the major quality aspects that may have impacted the interpretation of results are described in the results and discussion sections.

The quality aspects that were considered when interpreting the results of each study and the body of literature as a whole are as follows.

#### NSS intake assessment

Although less than ideal, we considered studies that collected information on NSS consumption over at least some time period (even when based on a 24-h survey with no information on frequency of consumption) to be of higher quality than those that collected intake information only once or over a short time period (e.g., from two or three 24-h surveys taken over 2 wk). In addition, we considered any assessment that evaluated the amount or frequency (i.e., "dose") of NSS consumption to be higher quality than assessments that evaluated risks associated with ever compared with never consumption, although the ideal characterization of dose would include intake amount, frequency, and duration, as well as any changes over time. Therefore, our assessment of NSS intake quality was relative, where studies that evaluated intake dose or intake over time were deemed of higher quality than those that assessed any intake compared with no intake, or that assessed intake only at a single time point, although some of these studies did not likely capture NSS consumption appropriately. As a result, among studies that we categorized as higher quality, those that assessed intake level, frequency, and duration are more informative than studies that do not address all three metrics. We discuss the specific metrics analyzed in the results and discussion section.

Studies that assess a specific NSS (e.g., aspartame, sucralose, and saccharin) are of higher quality than studies that assess NSSs more generally [e.g., artificially sweetened beverages (ASBs)] because many foods and beverages contain mixtures of multiple NSSs [11]. Furthermore, assessments of NSSs in the full diet (foods, tabletop packets, and beverages) or from a major source (e.g., beverages) are more complete than studies that assess intake from one minor source (e.g., tabletop packets only). Also, the amount and type of NSSs in foods and beverages have changed over time; this was also considered to ensure an accurate exposure assessment.

#### Confounding/covariate consideration

Very few nutritional epidemiology studies have information on all potentially important confounders/covariates (including sex, age, height/weight/obesity, total caloric intake, genetic factors, prior or family history of cancer, dietary factors, smoking, and alcohol consumption). Because we were concerned with relative quality among the studies, we concluded that studies that matched for, adjusted for, or stratified by age, sex, and at least two key risk factors specified in Table 2 [[12], [13], [14], [15], [16], [17], [18], [19], [20], [21], [22], [23], [24], [25], [26], [27], [28], [29], [30], [31], [32], [33], [34], [35], [36], [37], [38], [39], [40], [41], [42], [43]] (at least one of which was smoking for lung cancer and bladder cancer) were deemed to be of higher quality, or as having lower risk of serious confounding. Higher quality cohort studies collected and considered information on covariates that can change over time (e.g., obesity and diabetes) using time-varying methods, because controlling only for a single or a few measurements at baseline can result in residual confounding.

**TABLE 2:** Common risk factors for specific cancers

Cancer type	Risk factors
Most cancer types	Sex, age, BMI/weight/obesity, physical activity, diet, total caloric intake, genetics, personal or family history of cancer, smoking [[12], [13], [14], [15], [16]]
Urinary tract	
Bladder/lower urinary tract	Chemicals exposures (e.g., arsenic and chemicals used in the manufacture of dyes, rubber, leather, textiles and, paint products), chronic bladder irritation (e.g., chronic or repeated UTI), pioglitazone (diabetes medicine), dietary supplements containing aristolochic acid, fluid intake [17]
Kidney	Blood pressure, certain medicines (e.g., acetaminophen), advanced kidney disease [18]
Gastrointestinal system cancers	
Oral cavity, larynx and pharynx	Betel quid or gutka use, UV light, viral infection (e.g., HPV and Epstein–Barr virus), gastroesophageal reflux disease [19,20]
Esophagus	Gastroesophageal reflux disease, Barrett's esophagus, achalasia, tylosis, Plummer Vinson syndrome, injury to the esophagus, history of certain cancers (lung, mouth, or throat), HPV infection [21]
Stomach/gastric cardia	Gastroesophageal reflux disease, geography (more common in East Asia, Eastern Europe, South and Central America), *H. pylori* infection, adenomatous polyps, pernicious anemia, Menetrier disease, history of gastritis or adenomatous polyps, certain occupations (e.g., working in coal, metal, or rubber industries) [22,23]
Colorectum	Smoking, alcohol consumption, colorectal polyps, history of inflammatory bowel disease, type 2 diabetes, radiation therapy, family history of colorectal cancer [23,24]
Pancreas	Diabetes, chronic pancreatitis [25]
Biliary system cancers	
Liver	Chronic hepatitis infection, cirrhosis, inherited metabolic disease (hereditary hemochromatosis), type 2 diabetes, heavy alcohol use, aflatoxin exposure, chemical exposure or drug use (e.g., vinyl chloride, thorium dioxide, and anabolic steroids) [23,26]
Gall bladder	Gallstones, other gallbladder diseases (e.g., polyps, chronic inflammation, or infection), inflammation of the bile duct, geography, abnormalities of the bile ducts [27]
Central nervous system/brain cancers	Radiation, weakened immune system [28,29]
Female breast	Height, dense breast tissue, benign breast conditions (e.g., atypical ductal hyperplasia, lobular carcinoma in situ), early in life menstruation (before age 12 y), later menopause (after 55 y), radiation to the chest, diethylstilbestrol, having first child after age 30 y, not breast feeding, hormonal birth control use, hormone therapy [30,31]
Respiratory system cancers	
Lung	Secondhand smoke, exposure to radon, asbestos, other workplace agents (e.g., radioactive ores, arsenic, beryllium, cadmium, silica, nickel, chromium, coal products, mustard gas, and chloromethyl ethers), dietary supplements (e.g., beta carotene), radiation, air pollution [32]
Female reproductive cancers	
Ovary	Endometriosis, menstrual history, never having a full-term pregnancy, hormone replacement therapy, personal or family history of breast cancer [33]
Uterine/endometrial	Hormone balance; hormone therapy; hormonal birth control use; menstrual history; parity; tamoxifen use; PCOS; personal or family history colon, breast, or ovarian cancer; genetics (e.g., BRCA1 or BRCA2); obesity [34,35]
Male cancers	
Prostate	Geography (North America, northwest Europe, Australia, and Caribbean islands) [36]
Hematologic and lymphoid cancers	
Leukemia	Previous cancer treatments, smoking, race/ethnicity, certain chemical exposures, family history/genetics [37,38]
Multiple Myeloma	Other plasma cell disease [39]
Hodgkin lymphoma	Epstein–Barr virus or HIV infection, weakened immune system [40]
Non-Hodgkin lymphoma	Geography, exposure to chemicals or drugs (e.g., certain chemotherapies and drugs used to treat rheumatoid arthritis), radiation, weakened immune system (e.g., HIV infection), autoimmune disease (e.g., lupus, Sjogren disease, celiac disease), certain infections (e.g., human T-cell lymphotropic virus, Epstein–Barr virus, human herpes virus 8, *H. pylori*, *Chlamydophila psittaci*, *Campylobacter jejuni*, hepatitis C virus) [41]
Thyroid	Radiation, iodine intake [42]
Skin cancer/melanoma	Sun exposure (UV light), use of tanning beds or sun lamps, moles, having lighter skin, hair or eye color, personal or family history of skin cancer, weakened immune system, *Xeroderma pigmentosum* [43]

Known risk factors that are common to most types of cancer are shown in the top row. Additional risk factors for individual cancers are listed below.

Abbreviations: ACS, American Cancer Society; BRCA1, breast cancer gene 1; BRCA2, breast cancer gene 2; CDC, Centers for Disease Control and Prevention; HPV, human papillomavirus; PCOS, polycystic ovary syndrome; UTI, urinary tract infection; UV, ultraviolet light.

All cancers have different etiologies and risk factors; this is also true for some subtypes of certain cancers [44]. As a result, in studies in which groups of cancers are combined as the outcome, there is likely to be uncontrolled confounding, as it is unlikely that all relevant confounders for all exposure–outcome relationships are controlled for. Also, as noted above, a variety of NSSs may be found in certain food and beverage products [45]. In a food or beverage that contains a mixture of NSSs, if there is a specific NSS that is associated with a particular cancer, this could confound observed associations between other NSSs and that cancer outcome.

#### Outcome assessment

Self-reported outcomes may be inaccurate or incomplete, and are of lower quality compared with those confirmed by medical professionals or in medical records, death certificates, or registries, which are more likely to be reliable and complete. To be considered higher quality, we required a minimum latency period for solid tissue tumors of 4 y and for hematologic or lymphatic cancers of 6 mo [46] to ensure that a sufficient duration of time has occurred between exposure and outcome with respect to the specific latency of the disease being examined. These latency periods are purposefully conservative (i.e., shorter) to err on the side of overestimating quality. Studies that evaluate aggregated cancer types (e.g., all cancers combined) that are nonspecific are generally not appropriate, because cancers all have different underlying etiologies [44].

#### Selection bias

We considered studies that had enrollment or retention rates >75% [47] and that were nondifferential (<15% difference in participation) by outcome (case-control studies) to be less likely to be subject to selection bias.

### Evidence synthesis

Guided by the Bradford Hill considerations [48], we determined the overall plausibility of a causal association between each NSS and each specific cancer type that was evaluated in at least three observational studies. For each cancer, we considered the strength and consistency of associations reported, whether NSS intake occurred sufficiently before cancer diagnosis, and whether there was evidence of a dose–response relationship. We considered whether any associations were specific, the biological plausibility of associations, and the coherence of evidence as a whole. Importantly, we took study quality into account when interpreting study results and synthesizing evidence across studies.

The reporting of this systematic review was guided by the PRISMA checklist (Supplement B, Supplemental Table B.1) [49].

## Results

In **Epidemiology study selection**, we describe the results of the epidemiology literature search and screening. A high-level overview of the epidemiology studies included in this review and the quality of the studies as a whole are described in **Epidemiology study characteristics** and **Epidemiology study quality**, respectively. In **Epidemiology study results**, we discuss epidemiology study results for each cancer type that was evaluated in at least three studies. We integrate the evidence, guided by Bradford Hill's considerations [48] and incorporating study quality, in **Evidence integration**. Further detail is provided in the Supplements.

### Epidemiology study selection

From the literature searches, we identified 1436 records from PubMed and 1745 from Scopus. Before title/abstract screening, 648 duplicates identified between the PubMed and Scopus searches were excluded. Among the remaining 2533 records that were then screened by title/abstract, 281 were kept and further screened by full-text. We included 87 papers identified through our literature screening as well as an additional three papers identified through screening the references of included studies; the final number of included papers in this review was 90. The results of our literature screening are detailed in Supplement B, Supplemental Figure B.1.

### Epidemiology study characteristics

Of the 90 studies included in this review, 30 were cohort studies and 60 were case-control studies. These studies were conducted in several countries over the last several decades. Collectively, the studies evaluated intake of ace-K, aspartame, cyclamate, saccharin, sucralose, and mixtures of NSSs (e.g., ASBs or diet soda) and several cancers. These include cancers of the biliary system, including gallbladder and liver cancer; urinary tract cancers (UTCs), including bladder and kidney cancer; cancers of the gastrointestinal tract, including oral/pharyngeal/laryngeal, esophageal, stomach, intestinal, colorectal, and pancreatic cancer; cancers of the lymphohematopoietic system, including leukemia, Hodgkin's lymphoma, non-Hodgkin's lymphoma, multiple myeloma, and thyroid cancer; female reproductive cancer, including ovarian and endometrial or uterine cancer; and cancers of the breast, lung, prostate, brain, and skin. Several studies also evaluated several or all cancers combined. Table 3 provides an overview of the sweeteners and cancer endpoints examined, with more detail for each cohort and case-control study in Supplement C, Supplemental Tables C.1 and C.2, respectively. The specific cohorts examined in the reviewed studies are shown in Supplement D, Supplemental Table D.1. The characteristics of the included cohort and case-control studies are shown in Supplement E, Supplemental Tables E.1 and E.2, respectively.

**TABLE 3:** NSS and cancers evaluated in included studies

NSS	Organ system	Cancer type
Nonspecific NSS	Respiratory	Lung
Nonspecific NSS, saccharin	Female reproductive	Ovarian and uterine/endometrial
Nonspecific NSS, saccharin	Gastrointestinal	Oral/pharyngeal/laryngeal, esophageal, stomach, intestine, colorectal, pancreatic, gallbladder, and liver
Nonspecific NSS, saccharin, cyclamate	Urinary	Bladder and kidney
Nonspecific NSS, aspartame, saccharin	Lymphohematopoietic	Leukemia, Hodgkin's and non-Hodgkin's lymphoma, multiple myeloma, and thyroid
Nonspecific NSS, aspartame, ace-K, sucralose, saccharin	Male reproductive	Prostate
Nonspecific NSS, aspartame	Central nervous system	Brain
Nonspecific NSS, aspartame, ace-K, sucralose, saccharin	Other	Breast and skin

Abbreviations: Ace-K, acesulfame potassium; NSS, nonsugar sweetener.

### Epidemiology study quality

We provide an overview of study quality in Table 4, and a more detailed study quality evaluation of all identified cohort and case-control studies in Supplement E, Supplemental Tables E.3 and E.4, respectively. A high-level overview of study strengths and weaknesses is described below, and critical aspects of quality relevant to the weight of evidence for each NSS and cancer type are discussed in more detail in the context of the results in **Epidemiology study results**. Most studies did not have adequate information on intake of specific types of NSSs, did not consider changes in intake over time, and did not consider several key potential confounders.

**TABLE 4:** Epidemiology study quality

Aspect	Cohort studies	Case-control studies
Exposure assessment	All evaluated NSS intake before diagnosis. Almost all considered some aspect of dose (i.e., frequency, duration, or level of NSS consumption) and used a validated questionnaire to assess intake. Most did not consider intake at more than one point in time over follow-up.	All evaluated NSS intake after diagnosis. Approximately two-thirds of the studies did not consider dose. Almost none used a validated questionnaire. Less than half considered major sources of NSS intake; tabletop use was common in the 1970s and 1980s.
Outcome assessment	All except one study included cancer cases that were identified via a reliable source (i.e., self- or proxy-reported and verified by medical record or medical professional, or in a national insurance database, board of health, or cancer/death registry). Almost all evaluated incidence in at least some analyses. Only seven studies appropriately considered latency in the design or analysis.	All studies included cancer cases that were identified via a reliable source (i.e., self- or proxy-reported and verified by medical record or medical professional, or in a national insurance database, board of health, or cancer/death registry). Only seven studies appropriately considered latency in the design or analysis.
Confounding/covariate consideration	All studies used proper statistical models. All except two studies considered age, sex, and at least two other key covariates. Almost no studies considered the time-varying nature of relevant covariates.	Almost all studies used proper statistical models. Approximately half of the studies controlled for age, sex, and at least two other key covariates.
Sample selection	Most studies did not report on retention. All studies used appropriate comparison groups.	Approximately half of the studies either had high exclusion rates, low participation rates, differential participation between cases and controls, or did not report participation rates.

Some studies evaluated more than one type of sweetener independently. A more detailed evaluation of study quality can be found in Supplement E, Supplemental Tables E.3 and E.4.

Abbreviation: NSS, nonsugar sweetener.

#### Cohort studies

All cohort studies assessed NSS intake before diagnosis, and all but one [50] assessed major sources of NSSs (e.g., whole diet or diet soda). All but one study [51] considered the frequency, duration, or level of NSS consumption, and almost all used a validated questionnaire to assess intake. Only a few studies assessed NSS intake at more than one point in time over follow-up, or assessed a specific type of NSS (i.e., most studies assessed NSS mixtures only). Only four studies [[52], [53], [54], [55]] were deemed higher quality with respect to exposure assessments, although they were not ideal (e.g., Debras et al. [52] evaluated levels of NSS intake, but did not assess consumption frequency and it is unclear how complete their prospective intake assessment was) (see **Study quality evaluation**). Limitations of intake assessments increased the likelihood of exposure misclassification in all studies, including these higher quality studies.

All cohort studies except one [56] included cancer cases that were identified *via* a reliable source (i.e., self- or proxy-reported and verified by medical record or medical professional or in a national insurance database, board of health, or cancer/death registry). Most studies evaluated incidence of a specific cancer in at least some analyses, but only seven studies appropriately considered disease latency either in the design or analysis [53,[57], [58], [59], [60], [61]].

All cohort studies used appropriate statistical models and appropriate forms of variables, but most did not consider the time-varying nature of relevant covariates. All but two studies controlled for at least age, sex and two other key risk factors [62,63].

All cohort studies used appropriate comparison groups in their analyses, but most did not report retention rates, limiting our ability to understand the potential for selection bias due to attrition.

#### Case-control studies

Case-control studies were much more heterogenous than cohort studies with respect to study quality. All case-control studies collected self-reported NSS intake information after cancer diagnosis, increasing the likelihood of recall bias. Most studies did not report using a validated questionnaire to assess intake, and most did not assess intake of specific NSSs, but rather evaluated NSS mixtures. A little less than half of the studies evaluated major sources of NSS intake, most that did not (particularly the studies in the 1970s and 1980s) evaluated tabletop use. Approximately one-third of the studies evaluated the frequency, duration, or level of NSS intake, but most studies limited analyses to any NSS intake or higher intake compared with no or lower intake. None of the case-control studies were high quality across all aspects of exposure assessment.

All case-control studies that evaluated cancer incidence used reliable sources to identify cases. All but seven studies [[64], [65], [66], [67], [68], [69], [70]] evaluated a specific type of cancer, but only seven [67,[71], [72], [73], [74], [75]] appropriately considered a latency period in the study design or analysis. Most studies used appropriate models and forms of variables, and most studies that did not failed to use an appropriate statistical model to account for matched variables in their analyses. Approximately half of the studies failed to control for age, sex, and at least two other key risk factors for the cancer being analyzed. Similar to the cohort studies, all case-control studies likely had residual and uncontrolled confounding.

Approximately half of the case-control studies were at higher risk for selection bias due to use of inappropriate control groups (i.e., hospital-based controls) or reported high rates of exclusion or low rates of participation among eligible participants. Approximately half of the studies either reported differential participation rates in cases and controls or, more often, did not report participation rates, limiting our ability to fully evaluate the potential for selection bias.

### Epidemiology study results

For cancers that were analyzed in at least three studies, we found no consistent associations with any NSS or NSS mixtures (e.g., diet sodas and ASBs), or evidence for dose–response. NSS intake generally occurred sufficiently before cancer diagnosis, but recall bias was likely an issue in all case-control studies. Evaluations of either NSS mixtures or aggregate cancers (e.g., all cancers, nonobesity related cancers, UTCs) were nonspecific. Even when one NSS and one cancer type were assessed, the specificity requirement was not met, as all cancers have many risk factors. There are no experimental (i.e., interventional) studies in humans for any NSS and any cancer due to ethical considerations. Furthermore, experimental animal and mechanistic evidence regarding genotoxicity and carcinogenicity for each NSS does not support a biologically plausible mechanism by which any NSS could cause cancer in humans. The overall evidence is coherent, in that the epidemiology and experimental evidence collectively does not support the hypothesis that NSSs individually or in combination can cause cancer in humans.

We report the results of each included study, for cancers that were analyzed in at least three studies, in tables in Supplements F through N. The supplements also include a narrative review of cohort and case-control study results for individual NSSs or NSSs in combination for each specific cancer type for which there were at least three studies. Below we discuss the evidence for cancer types for which there were at least three studies, followed by an evaluation of this evidence in the context of Bradford Hill's considerations in **Evidence integration**, accounting for the impact of study quality on the interpretation of results.

#### Breast cancer

Six cohort and three case-control studies evaluated NSS consumption and breast cancer risk [52,54,58,59,61,[76], [77], [78], [79]] (Supplement F, Supplemental Tables F.1 and F.2). The quality of epidemiology studies varied considerably, particularly with respect to exposure characterization and control for confounding. All but one case-control study [77] accounted for several covariates, including age, and at least two additional breast cancer risk factors, and all studies were limited to women. Similarly, all used proper models and forms of variables. Only the two Nurses' Health Study (NHS) studies [54,61] considered the time-varying nature of relevant covariates (e.g., weight change since age 18 y). However, no study included all key covariates, and uncontrolled confounding and reverse causality could not be ruled out in any study.

Most of the studies were subject to multiple sources of bias or could not establish temporality or appropriately account for disease latency. Most importantly, only four studies, two cohort and two case-control, evaluated specific NSSs; the other six studies (four cohort and one case-control) only evaluated nonspecific NSSs, preventing the ability to evaluate the specific relationships between each sweetener and breast cancer. Among the assessments of specific NSSs, only aspartame was evaluated in more than one study.

All cohort studies and one case-control study [79] assessed major sources of NSS (e.g., whole diet and diet soda). NSS intake was ascertained by FFQs, 24-h dietary records collected over time, or questionnaires. Romanos-Nanclares et al. [54,61] collected information on the frequency of consumption at multiple points in time, whereas Debras et al. [52] collected information on 24-h intakes (up to 15 times, but only 2–3 times for most participants) across a 2-y period to establish baseline consumption, and then up to three times every six mo thereafter. However, it is not clear how complete the baseline or follow-up data are because Debras et al. [52] did not report this information in detail (i.e., Debras et al. [52] report more than 50% of participants completed two or three surveys; fewer completed four or more surveys). In addition, the time-varying nature of NSS intake was only considered in sensitivity analyses without specifying the fraction of surveys completed or the fraction of participants who completed these surveys after baseline. Hodge et al. [76], McCullough et al. [58], and Mullee et al. [59] and all three of the case-control studies collected information at only one point in time. All but Hodge et al. [76], McCullough et al. [58], and Ewertz and Gill [77] used a validated questionnaire. Even when using a validated questionnaire, the likelihood of one or a few NSS intake assessments, particularly those that only capture level of intake (e.g., 24-h intake) and not frequency *and* level of intake, may not capture the true and often dynamic NSS intake over time. All but one cohort study assessed consumption frequency before diagnosis; Debras et al. [52] collected information on the level, but not frequency, of NSS consumption. All case-control studies evaluated intake after diagnosis, increasing the potential for recall bias.

All of the cohort studies used appropriate comparison groups. One case-control study [77] used appropriate controls but the other two case-control studies used hospital-based controls. Two case-control studies [77,79] and one of the cohort studies [59] reported high participation or retention rates, respectively, but the remainder of the studies (i.e., one case-control and six cohort studies) did not report participation or retention rates, limiting our ability to assess the potential impact of selection bias on reported results.

There were no consistent increased risks of breast cancer associated with NSSs generally or any specific NSS. For NSSs as a whole, reported associations were weak, with point estimates ranging from 0.78 to 1.34. Several studies reported decreased risks associated with increased NSS intake. No study reported an increased breast cancer risk with increased NSS intake, except for Debras et al. [52], which reported a higher risk in higher aspartame consumers compared with lower and nonconsumers based on sparse 24-h recall data. In contrast, Romanos-Nanclares et al. [54] reported no association between any level of aspartame intake and breast cancer overall and a decreased risk of ER+ (*P-*trend = 0.01) and luminal A (*P-*trend < 0.01) breast cancers associated with increasing aspartame intake. Romanos-Nanclares et al. [61] also reported a small decreased risk of luminal A breast cancer with increasing ASB consumption (*P-*trend = 0.02). There is no evidence for a consistent dose–response relationship for NSS generally or any specific NSS. For evidence integration, see **Evidence integration**.

#### Liver cancer

We identified four cohort studies that examined NSS intake and liver cancer [57,58,80,81] (Supplement G, Supplemental Table G.1). There were no case-control studies that evaluated NSSs and liver cancer. All cohort studies evaluated major contributors of NSS intake, considered frequency or duration of intake, validated cases with clinical records, accounted for multiple covariates, including age and at least two additional risk factors for liver cancer, and used appropriate statistical models [57,58,80,81]. All studies except McCullough et al. [58] used a validated questionnaire, but none of the studies collected intake data at multiple timepoints, considered any specific NSSs, or the time-varying nature of relevant covariates, increasing the likelihood of exposure misclassification [57,58,80,81]. The exposure assessment in McCullough et al. [58] reflected ASB consumption between 1972 and 1982, which would have been almost exclusively saccharin. Since that time saccharin has been almost completely replaced by other NSSs.

Overall, associations with NSS were weak, and none of the studies reported a dose–response trend. For evidence integration, see **Evidence integration**.

#### Bladder cancer

NSS intake and bladder cancer risk was evaluated in two cohorts and 27 case-control studies [58,63,[71], [72], [73], [74], [75],[82], [83], [84], [85], [86], [87], [88], [89], [90], [91], [92], [93], [94], [95], [96], [97], [98], [99], [100], [101], [102], [103]] (Supplement H, Supplemental Tables H.1 and H.3). The two cohort studies reported risk estimates between 0.75 and 1.09, and none were statistically significant. The risk estimates reported in the case-control studies varied widely, ranging from 0.35 to 7.52. Statistically significant increased and decreased risk estimates were reported. There were no consistent increased or decreased risks across studies. Most of the risk estimates were close to 1, and the few strong associations reported were based on a small number of exposed participants (e.g., six exposed cases and two exposed controls in Mommsen et al. [93]).

There were no consistent associations between NSS intake overall (e.g., ever compared with never) or any dose–response trends reported across studies. Several studies evaluated risk stratified by sex and, similar to results for all participants, there were no consistent associations between NSS intake overall (e.g., ever compared with never) or any dose–response trends reported across studies for either sex.

The quality of these studies varied considerably, particularly with respect to exposure assessments and control for confounders. Both cohort studies controlled for age, sex, smoking and at least one other bladder cancer risk factor, but neither controlled for the time-varying nature of relevant covariates. In contrast, only approximately one-third (*n* = 10) of the case-control studies met these minimum criteria; most did not adequately control for smoking, which has been estimated to cause approximately half of all bladder cancers [104]. Most of the case-control studies (*n* = 20) were conducted in the 1970s or 1980s, when smoking was more prevalent than it is currently [105], further increasing the likelihood of uncontrolled confounding from smoking in these studies.

The temporal relationship between NSS intake and bladder cancer was not addressed in most studies; only one of the cohort studies and five of the case-control studies appropriately considered the latency period in either the study design or analysis. Both cohort studies and most of the case-control studies used appropriate comparison groups or controls. One of the cohort studies [53] and approximately one-third of the case-control studies (*n* = 10) either did not report on loss-to-follow-up or participation rates or reported low retention or participation.

Both cohort studies considered major sources of NSSs, but only approximately half (*n* = 12) of the case-control studies considered major sources; the studies that did not consider major sources primarily considered tabletop use. Neither cohort study collected NSS intake information at multiple time points, and only one case-control study [102] reported using a validated questionnaire to assess intake, so exposure misclassification was likely an issue in both cohort studies and all but one case-control study. All case-control studies collected intake information after cancer diagnosis, increasing the potential for recall bias. Neither cohort study evaluated a specific NSS, and only half of the case-control studies evaluated specific NSSs (i.e., saccharin or cyclamate). Given that most of the case-control studies evaluated exposures in the 1980s or earlier, the primary sources of NSS in the studies would likely have been a combination of saccharin and cyclamate. For evidence integration, see **Evidence integration**.

#### Kidney cancer

NSS intake and kidney cancer risk was evaluated in five cohort studies [56,58,63,76,106] and four case-control studies [78,[107], [108], [109]] (Supplement H, Supplemental Tables H.2 and H.4). Most of the studies did not assess a specific NSS; only two case-control studies [78,107] evaluated a specific NSS sweetener (i.e., saccharin). Collectively the studies did not report any consistent associations. Most studies, including all five cohort studies, reported no associations between NSSs and kidney cancer, and most risk estimates were close to one.

Two case-control studies reported an increased risk of kidney cancer associated with increased NSS intake [108,109], but Asal et al. [109] reported an increased risk only among males, and neither study assessed a specific NSS. Exposure misclassification was likely, as half of the studies did not use a validated questionnaire to assess NSS intake, and only Lee et al. [56] assessed intake at multiple time points. All case-control studies assessed exposure after kidney cancer diagnosis, increasing the potential for recall bias.

Most of the studies evaluated a major source of exposure and all but one considered dose. Most of the studies evaluated kidney cancer incidence identified from or validated through reliable sources, but only one study [58] considered an appropriate duration of time between exposure and outcome in its design or analysis to sufficiently capture the latency period.

All studies except Goodman et al. [107] considered at least age, sex, and some additional key potential confounders. Only one cohort study [56] considered the time-varying nature of relevant covariates. All of the cohort studies and half of the case-control studies [108,109] used appropriate comparison groups or controls, respectively, but more than half of the studies either did not report on loss-to-follow-up [53,76,106] or participation rates [78,109]. Three studies reported high rates of retention (≥75%) or high and nondifferential rates of participation. One study [108] reported participation rates in controls (45%). For evidence integration, see **Evidence integration**.

#### Esophageal cancer

One cohort and three case-control studies evaluated NSS intake and esophageal cancer risk [58,78,110,111] (Supplement I, Supplemental Tables I.1 and I.5). The studies varied considerably in quality, particularly with respect to exposure characterization and sample selection. Only two studies, Gallus et al. [78] and Ibiebele et al. [110], reported using a validated questionnaire. The cohort study [58] did not collect exposure data at multiple time points, but was the only study to collect exposure data before cancer diagnosis, thus able to establish that exposure occurred before diagnosis (i.e., temporality). All case-control studies collected information on NSS after cancer diagnosis, increasing the potential for recall bias. Of the four studies, three assessed at least one major source of NSS (i.e., diet soft drinks or diet beverages); Gallus et al. [78] was the only study to only evaluate minor sources of NSS (i.e., tabletop sweeteners). Three studies considered frequency, duration, or level of NSS consumption; Ibiebele et al. [110] examined only ever/never intake.

All studies considered some key potential confounders, but the cohort study [58] did not consider the time-varying nature of relevant covariates. Three studies used appropriate comparison groups, but Gallus et al. [78] relied on hospital-based controls. McCullough et al. [58] did not report on loss to follow-up, and the three case-control studies either excluded a substantial number of participants (≥25%) or did not report participation rates. Only Ibiebele et al. [110] provided participation rates for both cases and controls, observing nondifferential rates (≤15%) between the two groups.

Risk estimates for NSS intake and esophageal cancer varied widely (0.43–1.58), although all statistically significant associations were below one. Only McCullough et al. [58] and Gallus et al. [78] evaluated a dose–response relationship, and neither reported an increased risk in any intake group or any significant trends. The Gallus et al. [78] study was the only study to evaluate a specific NSS; they reported no association between any tabletop saccharin intake and esophageal cancer. For evidence integration, see **Evidence integration**.

#### Stomach cancer

Two cohort and three case-control studies evaluated NSS consumption and stomach cancer risk [58,76,79,111,112] (Supplement I, Supplemental Tables I.2 and I.6). The studies varied considerably in quality, particularly with respect to the exposure assessment. Only two case-control studies, Bosetti et al. [112] and Palomar-Cros et al. [79], reported using a validated questionnaire. These were also the only studies to assess stomach cancer risk associations with specific NSS types. Of the five studies, four assessed at least one major source of NSS (i.e., diet soft drinks or ASBs), whereas Bosetti et al. [112] exclusively evaluated minor sources of NSS (i.e., tabletop sweeteners). Bosetti et al. [112] was also the only study that did not consider frequency, duration, or level of NSS consumption and only considered ever-use of low-calorie sweeteners or saccharin. None of the studies assessed exposure at multiple time points, although both cohort studies collected exposure information before cancer diagnosis.

All of the studies considered some key potential confounders, but neither cohort study considered the time-varying nature of relevant confounders. The two cohort studies and one case-control study [111] did not report on loss-to-follow-up or participation rates, respectively, impacting the ability to assess selection bias. The two other case-control studies used hospital-based controls, which can also increase the likelihood of selection bias.

There were no consistent increased or decreased risks of stomach cancer associated with general or specific NSS consumption. For NSSs as a whole, reported associations were weak, with risk estimates ranging from 0.50 to 1.24; most were between 0.8 and 1.2. None of the studies that considered levels of intake reported increased risks with any level of intake, and no significant dose–response trends were reported. Both Bosetti et al. [112] and Palomar-Cros et al. [79] reported no association between saccharin intake and stomach cancer. Palomar-Cros et al. [79] reported no association between aspartame and stomach cancer. For evidence integration, see **Evidence integration**.

#### Colorectal cancer

Six cohort and seven case-control studies evaluated NSS consumption and colorectal cancer risk [58,59,76,78,79,[113], [114], [115], [116], [117], [118], [119], [120]] (Supplement I, Supplemental Tables I.3 and I.7). The studies varied considerably in quality, particularly with respect to exposure characterization and sample selection. Three cohort studies [117,118,120] and two case-control studies [58,76] did not report using a validated questionnaire. The cohort studies all collected exposure information before cancer diagnosis, assessed major sources of NSS (i.e., beverages, diet beverages, and soft drinks), and considered frequency, duration, or level of NSS consumption. However, none of the cohort studies assessed specific NSS types, whereas two case-control studies [78,79] did. Only two case-control studies [79,119] considered major sources of NSS intake (i.e., beverages or low-calorie drinks) and four case-control studies [78,79,117,119] considered frequency, duration, or level of NSS intake. None of the case-control studies collected exposure information before cancer diagnosis, increasing the potential for recall bias.

In terms of participant selection, all cohort studies used appropriate comparison groups, whereas four case-control studies [78,79,116,120] used inappropriate controls (i.e., hospital-based controls or controls with other cancer types). Most cohort studies did not report on loss to follow-up, and only two case-control studies [79,116] had low exclusion/high participation rates. These two studies, as well as Theodoratou et al. [119], reported nondifferential participation rates (≤15%), whereas the other studies either reported differential participation in cases and controls or did not provide information on participation.

Most of the studies controlled for some key confounders, but Mahfouz et al. [118] and Wu et al. [120] only controlled for family history of colorectal cancer and age, sex and ethnicity, respectively. Only two studies [113,114] considered the time-varying nature of relevant covariates.

Overall, these studies did not report consistent increased or decreased risks of colorectal cancer associated with any NSS. For NSS as a whole, most reported associations were weak, with point estimates ranging between 0.7 and 1.5. Only one study reported a statistically significant increased risk of colorectal cancer [118]. Mahfouz et al. [118] reported a large increase in colorectal cancer risk among NSS users (OR: 20.8; 95% CI: 2.7, 159.7), but this risk estimate is statistically unstable and the study had many quality concerns. Two studies reported decreased risk with NSS intake [79,114]. For evidence integration, see **Evidence integration**.

#### Pancreatic cancer

Four cohort and six case-control studies evaluated NSS consumption and pancreatic cancer risk [58,60,62,112,[121], [122], [123], [124], [125], [126]] (Supplement I, Supplemental Tables I.4 and I.8). These studies varied considerably with respect to quality. The cohort studies were generally of higher quality than the case-control studies, although all had some critical quality concerns. Schernhammer et al. [121] was the only study to collect exposure information at multiple time points and to consider the time-varying nature of relevant covariates (e.g., diabetes, smoking, diet). Schernhammer et al. [121] was also the only study to adjust for diabetes, a key risk factor for pancreatic cancer. Only two cohort studies [60,62] reported high retention of participants. None of the cohort studies examined specific NSS types, whereas two case-control studies did [112,123]. Among the case-control studies, only Bosetti et al. [112] had a low number of excluded participants (<25%) and only Bosetti et al. [112] and Chan et al. [122] reported using validated questionnaires. None of the case-control studies collected exposure information before cancer diagnosis, or adequately address latency (i.e., ≥4 y).

There were no consistent increased or decreased risks of pancreatic cancer associated with NSS or saccharin intake. For NSS as a whole, risk estimates varied widely, with point estimates ranging from 0.19 to 1.8, but most were between 0.6 and 1.3. Three cohort studies [58,60,62] and three case-control studies [112,122,124] reported statistically significant findings. All three reported increased risks of pancreatic cancer associated with NSS intake [58,60,62]; although all estimates were close to one (i.e., <1.35). Of the case-control studies, Chan et al. [122] also reported an increased risk of pancreatic cancer, but Bosetti et al. [112] and Gold et al. [124] reported decreased risks in at least some analyses. Norell et al. [126] and Wynder et al. [125] reported no association with NSS and saccharin, respectively. Four studies evaluated a dose–response relationship between NSS and pancreatic cancer, and only McCullough et al. [58] reported a significant trend. Notably, Schernhammer et al. [121], the only study to adjust for diabetes and the only study to evaluate covariates or NSS intake at multiple timepoints, reported no association between NSS intake and pancreatic cancer at any level of intake, and reported no significant dose–response trend. The lack of adjustment for diabetes and reliance on exposure information collected only at baseline in most studies, including McCullough et al. [58], means that confounding or reverse causation cannot be ruled out as explanations for reported associations. For evidence integration, see **Evidence integration**.

#### Leukemia

NSS intake and leukemia risk was evaluated in three studies of four cohorts and one case-control study [55,58,127,128] (Supplement J, Supplemental Tables J.1 and J.4). All three cohort studies and the case-control study assessed major sources of NSS and considered frequency or intake level of consumption. Two of the studies assessed aspartame specifically [55,127], whereas McCullough et al. [58] and Li et al. [128] only evaluated NSS mixtures. Two of the cohort studies and the case-control study used a validated questionnaire [55,127,128], but only Schernhammer et al. [55] collected exposure data at multiple time points and evaluated the time-varying nature of relevant covariates. All three cohort studies collected exposure data before leukemia diagnosis, thus able to establish that exposure occurred before diagnosis (i.e., temporality). The case-control study collected information on NSS after cancer diagnosis, increasing the potential for recall bias.

All of the cohort studies used appropriate comparison groups but none reported on loss-to-follow-up. The case-control study [128] relied on hospital base-controls and the participation rates were nor reported. For evidence integration, see **Evidence integration**.

#### Non-Hodgkin’s lymphoma

Three cohort studies and one case-control study evaluated NSS and non-Hodgkin's lymphoma [53,55,79,127] (Supplement J, Supplemental Tables J.2 and J.5). All these studies assessed major sources of NSS and considered frequency of consumption or intake level. All four studies also assessed specific NSSs in at least some analyses and used a validated questionnaire [53,55,79,127] with only one of the studies collected exposure and covariate data at multiple time points [55]. All three cohort studies collected exposure data before diagnosis. The case-control study collected information on NSS after cancer diagnosis, making it vulnerable to recall bias. However, the authors asked about NSS consumption before diagnosis, and thus all four studies established exposure occurred before diagnosis (i.e., temporality). All cohort studies used appropriate comparison groups but Palomar-Cros et al. [79] relied on hospital-based controls. Loss-to-follow-up was not reported in any of the cohort studies. For evidence integration, see **Evidence integration**.

#### Multiple myeloma

Three cohort studies evaluated NSS consumption and multiple myeloma risk [53,55,127] (Supplement J, Supplemental Table J.3). There were no case-control studies of NSSs and multiple myeloma. All three studies assessed major sources of NSS and considered frequency of consumption or intake level. All three of the studies assessed specific NSSs in at least some analyses and used a validated questionnaire [53,55,127]. Two collected intake data at multiple time points [53,55], but only McCullough et al. [53] considered the time-varying nature of relevant covariates. All three cohort studies collected exposure data before multiple myeloma diagnosis, and thus were able to establish temporality. All studies used appropriate comparison groups but none reported on loss to follow-up. For evidence integration, see **Evidence integration**.

#### Ovarian cancer

Two cohort studies and one case-control study evaluated NSS intake and ovarian cancer risk [58,76,78] (Supplement K, Supplemental Tables K.1 and K.3). Both cohort studies evaluated major sources of NSS intake, considered frequency or duration of intake, validated cases with clinical records, and accounted for key covariates, including age and at least two additional risk factors for ovarian cancer, but neither considered the time-varying nature of relevant covariates. Neither study used a validated questionnaire, collected intake information at multiple timepoints, or evaluated a specific NSS, increasing the likelihood of exposure misclassification. The case-control study evaluated saccharin and NSS mixtures from a minor dietary source (i.e., tabletop use). The study also used a validated questionnaire and considered key potential confounders. Exposure was assessed after cancer diagnosis increasing the potential for recall bias.

Both cohort studies used appropriate comparison groups but neither reported on loss-to-follow-up. The case-control study [78] relied on hospital-based controls and did not report on participation rates. For evidence integration, see **Evidence integration**.

#### Uterine cancer

Three cohort studies and one case-control study evaluated NSS intake and risk of uterine cancer [58,76,112,129] (Supplement K, Supplemental Tables K.2 and K.4). All cohort studies evaluated major sources of NSS intake, and accounted for several key potential confounders [58,76,129]. None of the cohort studies collected data at multiple timepoints or considered the time-varying nature of relevant covariates and none evaluated a specific NSS. Only Inoue-Choi et al. [129] used a validated questionnaire, so there was a high likelihood of exposure misclassification in the other studies [58,76]. The case-control study evaluated a specific NSS (saccharin), had a valid exposure assessment, and considered several key potential confounders; however, it evaluated saccharin exposure from a minor source (tabletop) at a single time point before cancer diagnosis, which may have resulted in an underestimation of exposure and the potential for recall bias, respectively. All cohort studies used appropriate comparison groups but none reported on loss-to-follow-up. Bosetti et al. [112] reported high rates of participation but relied on hospital-based controls. For evidence integration, see **Evidence integration**.

#### Prostate cancer

Four cohort and two case-control studies evaluated NSS consumption and prostate cancer risk [52,58,59,76,78,79] (Supplement L, Supplemental Tables L.1 and L.2). All studies accounted for multiple covariates, including age and at least two additional risk factors for prostate cancer, considered frequency or duration of NSS intake, validated cases with clinical records, and used proper statistical models. Two of the cohort studies did not use a validated questionnaire [58,76], and none considered the time-varying nature of relevant covariates [52,58,59,76]. Debras et al. [52] was the only cohort study to evaluate specific NSSs (although limited to 24-h dietary recall data and lacking data on consumption frequency), and McCullough et al. [58] was the only study to have sufficient time between intake and health outcome to account for disease latency. Only Debras et al. [52] collected consumption data at multiple time points, but most participants completed only two or three surveys. The completion of subsequent prospective follow-up surveys is not entirely clear but appears sparse given their primary analysis relies solely on the baseline-period data. All cohort studies used appropriate comparison groups but only one [59] reported high retention rates (≥75%), the other studies did not report on loss to follow-up.

Both case-control studies accounted for specific NSSs but only collected intake at a single time point after diagnosis, increasing the potential for recall bias, and both used inappropriate comparison groups (i.e., hospital or health center recruited controls) [78,79]. For evidence integration, see **Evidence integration**.

#### Brain cancer

Two cohort and four case-control studies evaluated NSS consumption and brain cancer risk [58,127,[130], [131], [132], [133]] (Supplement M, Supplemental Tables M.1 and M.2). None of the studies reported consistent associations between NSS intake and brain cancer in adults or children. The only statistically significant finding was an association between NSS mixtures and brain cancer mortality among overweight individuals, but not normal weight or individuals with obesity, in an analysis stratified by BMI [58]. This study did not use a validated questionnaire to collect exposure data, increasing the possibility of exposure measurement error [58]. Both cohort studies collected NSS data at one time point and did not account for time-varying covariates [58,127]. In addition, both used appropriate comparison groups but did not report on loss-to-follow-up.

None of the case-control studies considered key potential confounders or used a validated questionnaire, two used an appropriate comparison group [130,133] and only one reported a high participation rate [133]. Temporality could be established in the cohort studies; however, all the case-control studies collected NSS intake data after brain cancer diagnosis, increasing the potential for recall bias. For evidence integration, see **Evidence integration**.

#### Lung cancer

Two cohort and one case-control study [51,58,134] evaluated NSS consumption and lung cancer risk (Supplement N, Supplemental Tables N.1 and N.2). All three studies that evaluated lung cancer risk consistently reported a decreased risk associated with NSS intake. All evaluated NSS mixtures only, and only collected NSS intake data at one time point [51,58,134]. Two studies did not use validated questionnaires to collect intake data, increasing the possibility of exposure measurement error [58,134]. All three studies considered some key confounders including age, sex, and smoking. Temporality could be established in the cohort studies, but data on NSS was collected after diagnosis in the case-control study, increasing the potential for recall bias. McCullough et al. [58] reported a decreased risk with increased NSS intake; however, the risk estimates for each level of intake were quite similar and the sample size was very large (>900,000), which may have increased the likelihood of finding a statistically significant *P-*trend. Both cohort studies used appropriate comparison groups, but only You et al. [51] reported high retention of participants. Mettlin et al. [134] recruited controls with other types of cancer and did not report on participation rates, so selection bias cannot be ruled out. For evidence integration, see **Evidence integration**.

### Evidence integration

Most epidemiology studies do not report an association between any NSS and cancer. Where associations have been reported, most are inconsistent across studies and evidence does not support dose–response for NSSs in aggregate or individual NSSs and any cancer type, except for lung cancer. Decreased risks of lung cancer were consistently reported in epidemiology studies of NSS intake. Only one study evaluated dose–response, and this study reported a significant trend of decreased lung cancer risk with increased NSS intake, although all risk estimates were quite similar in magnitude.

Collectively, these epidemiology studies have several methodological limitations—most notably evaluating NSSs in aggregate and a high likelihood of exposure misclassification. All studies evaluated some key confounders, but most did not consider the time-varying nature of relevant covariates, and it is likely that residual and uncontrolled confounding impacted the results. Selection bias could not be ruled out in most studies. There is no evidence for specificity for any type of cancer.

In conjunction with this evaluation, we reviewed the available and mechanistic evidence on NSSs and cancer [135]. The weight of the evidence indicates that ace-K, advantame, aspartame, cyclamate, neotame, saccharin, steviol glycosides, and sucralose lack genotoxic potential. In addition, high-quality animal cancer bioassays have demonstrated a lack of tumorigenic response for the NSSs evaluated with one exception. Early animal studies indicated saccharin and cyclamate could cause bladder cancer in animal models. Later studies showed that the mechanism by which urinary crystals were formed in the rat bladder from saccharin exposure was not biologically plausible in or relevant to humans, and the associations with cyclamate were ultimately not reproducible in studies that followed [135]. There is no other evidence that any other NSS can cause cancer in animal models [[136], [137], [138], [139], [140]], and the weight of evidence does not support any biologically plausible mechanisms or modes of action.

Finally, the evidence is coherent, in that the epidemiology and toxicology evidence does not demonstrate that NSSs increase the risk of any cancer type.

## Discussion

We conducted a systematic review of epidemiology studies that evaluated ace-K, aspartame, cyclamate, saccharin, steviol glycosides, sucralose, neotame, advantame, or NSS mixtures and cancer risk. Compared with prior reviews on the subject, we focused on study quality domains that we determined were most likely to impact the interpretation of study findings and the body of evidence, as whole. In addition, we also conducted a high-level review of existing animal and mechanistic studies to assess biological plausibility for each NSS and cancer type to complement the detailed analyses of the human observational studies. Overall, we found no consistent associations or evidence for dose–response for any NSS or any cancer type.

### Exposure measurement error

Issues in studies evaluating specific NSS-cancer relationships are detailed in the relevant results section. We identified limitations with respect to measurement of NSS intake across most cohort and case-control studies and thus, this issue impacts the larger body of evidence on NSSs and cancer. Although cohort studies generally obtained information about NSS intake before cancer diagnoses, almost no case-control studies did, and were thus potentially subject to recall bias. Case-control studies may also have been subject to reverse causation because individuals with cancer may have changed their NSS intake after diagnosis. In those cohort studies that evaluated cancer risk among individuals that had risk factors for specific cancer types (e.g., diabetes and liver cancer), reverse causality cannot be ruled out as a possible explanation of observed associations [e.g., 57,141].

Regardless of study type, exposure measurement error is a concern due to the use of self-reported NSS intake information. Exposure measurement error, if differential, can bias the study results in either direction [142]. Most studies relied on information collected at only one point in time (rather than repeatedly over the study duration). It is unlikely that a single 24-h dietary recall measurement or a few measurements (of 24-h dietary recalls) over a short time period (e.g., >2 y in the NutriNet-Santé cohort [52,115]) can accurately reflect NSS consumption frequency over the long term. When evaluating causal associations between NSSs and a chronic outcome that can take decades to develop (e.g., cancer), accurately capturing both NSS intake amount and frequency over time (i.e., cumulative exposure) is critical. Most studies did not evaluate both intake amount and frequency (e.g., the NutriNet-Santé cohort collected only 24-h dietary recall data but did not collect frequency of intake [52,115]) and most did not update consumption information over time, instead relying solely on a baseline assessment to characterize exposure (e.g., the Melbourne Collaborative Cohort Study used an FFQ to capture consumption over the prior 12 mo at baseline [76,143]). The only studies that evaluated intake level and frequency prospectively over time involved the NHS, NHS-II and Health Professionals Follow-up Study cohorts. In addition, most studies did not evaluate NSS exposure in the whole diet, so it is also not clear to what extent these studies fully captured NSS intake. For example, if a study evaluated ASBs, it may have missed the use of tabletop sweeteners or NSSs in certain foods (e.g., yogurt).

Uncertainty was also introduced when the consumption "dose" was not assessed. Dose can be assessed in various ways, duration, e.g., frequency, level or amount of intake, or a combination of these (i.e., cumulative intake). Instead, when only ever compared with never [112] or 24-hr dietary recall data are collected (e.g., in the NutriNet-Santé cohort study [52,115]), the potential for exposure misclassification increases, as there is not a clear distinction between nonconsumers and regular NSS consumers.

Importantly, many studies evaluated exposure based on intake of NSSs generally. The composition of NSSs in food and beverages have changed over time (e.g., almost all NSSs in beverages in the United States before 1983 were saccharin, and this has been almost completely phased out of beverages in the United States market). Evaluations of mixtures generally and the changing composition of mixtures over time make evaluating relationships between any specific NSS and any specific cancer difficult. In addition, NSSs include substances of different chemical classes with varying physiochemical, pharmacokinetic and toxicologic properties. Therefore, it is not appropriate to group these substances together to evaluate potential health risks.

### Obesity or diabetes as potential confounders or mediators

Obesity is an established risk factor for many types of cancer, including adenocarcinoma of the esophagus, postmenopausal breast cancer, meningioma, multiple myeloma, and cancers of the colon and rectum, uterus, gall bladder, upper stomach, kidneys, liver, ovaries, pancreas, and thyroid cancer. Cancer risks generally increase with additional excess weight and duration of obesity. It has been suggested that obesity can contribute to cancer risk by causing chronic inflammation and increased insulin, insulin-like growth factor, and sex hormones [144,145].

Similarly, type 2 diabetes is associated with cancers of the liver, pancreas, endometrium, colon, rectum, breast, and bladder, and a reduced risk of prostate cancer. It has been suggested that increased risks may be due to aging, obesity, diet, and physical activity (rather than diabetes itself) or, similar to obesity, hyperinsulinemia, hyperglycemia, and inflammation [146].

Because one purpose of NSSs is to reduce sugar and caloric intake, individuals with obesity or type 2 diabetes may be more likely to consume higher levels of NSSs in the diet in place of sugars. Therefore, obesity and type 2 diabetes should be evaluated as potential confounders or mediators in studies of NSSs and the aforementioned cancer types. In addition, it has been demonstrated in randomized controlled trials that replacement of sugars with NSSs in the diet can have beneficial effects on cardiometabolic outcomes (e.g., glycated hemoglobin, blood glucose, blood insulin, lipid profile) [3,4,[147], [148], [149]], which suggests hyperglycemia and hyperinsulinemia cannot be a mode of action, as hypothesized by IARC [150]. Finally, because excess dietary calories contribute to both obesity and diabetes, any associations between NSS intake and cancer risk could be due to these or other dietary factors contributing to either obesity or diabetes, and not specifically the NSSs themselves. Most studies attempted to control for diet, but because this is a complex variable, it is unlikely that any study fully controlled for diet sufficiently. Some studies we reviewed did not address obesity or diabetes at all, whereas others may have considered these factors, but could not totally control for them, particularly when information on obesity and diabetes was only obtained at one point in time.

### Other reviews

Our findings are consistent with other recent reviews, which also reported a lack of high-quality evidence. As noted above, Toews et al. [3] and Rios-Leyvraz and Montez [4] both evaluated NSS intake and several health outcomes, including cancer. Toews et al. [3] stated, "Most health outcomes did not seem to have differences between the NSS exposed and unexposed groups. Of the few studies identified for each outcome, most had few participants, were of short duration, and their methodological and reporting quality was limited; therefore, confidence in the reported results is limited."

Rios-Leyvraz and Montez [4] stated, "Results from case-control studies suggest an association between saccharin intake and bladder cancer (very low certainty evidence), but significant associations for other types of cancer were not observed in case-control studies or meta-analysis of prospective cohort studies (very low to low certainty evidence)." They further noted that "studies included in the current review are decades old; many lack important details, including information on doses being consumed in the studies; and nearly half have serious risk of bias. As a result, confidence (certainty) in the results for bladder cancer is very low, and therefore the results must be interpreted cautiously" [4].

As discussed in more detail in Goodman et al. [141], JECFA and IARC evaluated cancer risks of aspartame in 2023. IARC [150] concluded aspartame was "possibly carcinogenic" based on three studies that evaluated liver cancer risks: Jones et al. [57], McCullough et al. [58], and Stepien et al. [81]. However, there are several limitations with the IARC [150] analyses and interpretation [5,141]. Most importantly, IARC inappropriately considered ASB consumption to be a good proxy for aspartame consumption [151]. However, McCullough et al. [58] evaluated NSS exposure between 1972 and 1982, at which time aspartame was not yet approved for use in beverages in the United States. Also, as noted above, most of the analyses in McCullough et al. [58] did not demonstrate an association and the one reported statistically significant trend across levels of consumption in nonsmoking males was no longer statistically significant in analyses that controlled for BMI. Jones et al. [57] reported associations only among participants with diabetes and noted a lack of statistical significance after 6 y, which may suggest reverse causality as an explanation for the association. Finally, Stepien et al. [81] could not distinguish between NSSs consumed, which impacts the ability to evaluate a specific association between aspartame and liver cancer. Zhao et al. [80], which was not considered in the IARC evaluation, reported no association between ASB and liver cancer. Also, IARC hypothesized that an aspartame carcinogenic mechanism could involve diabetes and inflammation [150]. However, numerous clinical trials show no or beneficial effects on critical diabetes biomarkers such as glycated hemoglobin, blood glucose, and blood insulin [3,4]; these studies do not support the IARC hypothesis [150]. In contrast to IARC [150], JECFA [152] concluded evidence was not convincing and that "reverse causality, chance, bias and confounding by socioeconomic or lifestyle factors, or consumption of other dietary components, could not be completely ruled out."

### Future research

There is a strong toxicology database for NSSs, which is the primary database utilized to assess NSS safety. Animal and mechanistic studies are necessary to overcome ethical concerns associated with conducting human trials to evaluate cancer outcomes. While observational studies can be leveraged to study potential associations in humans between nutrients and adverse outcomes such as cancer, almost all epidemiology studies likely have substantial exposure measurement error and other significant shortcomings, including residual and uncontrolled confounding. In many cases, these studies did not provide sufficient information to adequately judge study quality. For example, some studies did not discuss loss to follow-up, whether and how questionnaires were validated, and whether information was collected or updated in a time-varying manner. Our review could not overcome the limitations in these individual studies. Future studies should ensure that all information necessary to judge quality is included and easily accessible. More detailed information should be included in appendices, in online supplementary materials, or available from the study authors.

Future studies should also examine NSS intake more completely and, ideally, in a time-varying manner. Studies should also better evaluate factors associated with diet to better understand whether NSSs or other dietary factors are causally associated with certain health outcomes or whether other factors that are correlated with dietary factors (e.g., obesity, diabetes) are playing a causal role.

Finally, there may be opportunities in future studies for better validation of self-reported data or quantitative bias analyses to determine the degree to which inaccurate information impacts the interpretation of study results.

### Strengths and limitations

Our review has many strengths, most notably that we evaluated all NSSs and all cancer types, including a thorough evaluation of study quality that was transparently integrated into the interpretation of the results. We also integrated animal and mechanistic information into our evaluation to inform biological plausibility and coherence across evidence streams. We conducted our review following PRISMA guidelines with only one deviation. Owing to the broad scope of the review and resource constraints, one reviewer independently extracted data and evaluated study quality, and a second reviewer confirmed the accuracy of the extraction and evaluation, rather than the extraction and evaluation conducted in duplicate. We do not feel that this process resulted in any bias or inaccuracies in our results, as our protocol was detailed, the work was conducted in a systematic manner, and disagreements between the reviewers were noted and resolved through discussion, or by a third reviewer when necessary.

In conclusion, we have conducted a comprehensive systematic review of NSS intake and cancer risk. There have been many epidemiology studies of various NSSs and cancer risks. Most studies had a high likelihood of exposure measurement error and confounding, as well as recall bias in case-control studies. It is also difficult to interpret results of analyses of NSSs in aggregate. Regardless, these studies did not report consistent associations for any NSS or group of NSSs and any cancer type, or evidence of dose–response. This is further supported by an extensive database of randomized controlled trials that evaluate key biomarkers of obesity and diabetes, and experimental evidence, which does not provide evidence for carcinogenicity, genotoxicity, or other biologically plausible modes of action in humans. Overall, the scientific evidence does not indicate that NSSs cause cancer in humans.

## Author contributions

The authors’ responsibilities were as follows – DB, MJ, JEG: designed research; DB, SAM, KJC, IMC-P, WL, AB, CR: conducted research; DB, SAM, KJC, IMC-P, AB: analyzed data; DB, SAM, KJC, IMC-P, AB: wrote paper; DB, SAM, MJ, JEG: had primary responsibility for final content; and all authors: read and approved the final manuscript.

## Data availability

Data described in the manuscript, codebook, and analytic code will be made available on request pending application and approval.

## Funding

ABA provided funding for this paper, which was written during the authors' normal course of employment.

## Conflict of interest

All authors are employed by Gradient, Geosyntec, or the American Beverage Association (ABA). Gradient and Geosyntec are environmental and risk sciences consulting firms. ABA is the trade association that represents America's non-alcoholic beverage industry. ABA provided funding for this paper, which was written during the authors' normal course of employment. This paper represents the professional opinions of the authors and not those of ABA.
