# Supplementary material for: A Systematic Review of Nonsugar Sweeteners and Cancer Epidemiology Studies
Source: Adv Nutr. 2025 Sep 30;16(12):100527. doi: 10.1016/j.advnut.2025.100527 (PMC12639567; doi:10.1016/j.advnut.2025.100527)
Supplement: Multimedia component 1 [file mmc1.pdf]

Supplements

Supplement A: Literature Search Strategy and Criteria for Evaluation of Study Quality of Included Studies

Supplemental Table A.1 Search Strategy

Search Number	Query
**Pubmed**	
1	("artificial* sweet*" OR "Aspartame"[Mesh] OR Aspartame OR acesulfame OR "acetosulfame" [Supplementary Concept] OR "ace K" OR "Saccharin"[Mesh] OR Saccharin OR "neotame" [Supplementary Concept] OR neotame OR sucralose OR Splenda OR "trichlorosucrose" [Supplementary Concept] OR "advantame" OR "Cyclamates"[Mesh] OR Cyclamate OR "alitame" OR "Neohesperidin" OR "Stevia"[Mesh] OR stevia OR steviol OR stevioside OR rebaudioside OR rebiana* OR thaumatin* OR brazzein* OR mogroside*)
2	("Non-Nutritive Sweeteners"[Mesh] OR " Non-Nutritive Sweeteners" OR "Nutritive Sweeteners"[Mesh] OR "Nutritive Sweeteners" OR "Sweetening Agents"[Mesh] OR "Artificially Sweetened Beverages"[Mesh] OR "Sugar-Sweetened Beverages"[Mesh])
3	("artificial* sweet*" OR "sugar substitute*" OR "diet soda*" OR "diet beverage*" OR "diet drink*" OR "diet cola*" OR "sugar-free" OR "calorie-free" OR "artificially sweetened" OR "non-nutritively sweetened" OR "non-calorically sweetened" OR "soft drink*" OR cola OR "low calorie sweet*" OR "non sugar sweet*" OR "Non-caloric artificial sweeteners" OR "sugar substitutes" OR "sugary drinks")
4	#1 OR #2 OR #3
5	(Cancer* OR leukemia OR carcinogen* OR lymphoma OR myeloma OR neoplasm* OR carcinoma OR tumor OR tumors OR tumorous OR tumour OR sarcoma)
6	("Humans"[Mesh] OR human[Title/Abstract] OR child* OR infant* OR boy OR girl OR adolescent* OR teenager* OR men OR women OR female OR male OR adult* OR elderly OR population OR group OR epidemiolog* OR occupation* OR worker* OR cohort OR "Cohort Studies"[Mesh] OR "Case-Control Studies"[Mesh] OR "Case Reports" [Publication Type] OR "Retrospective Studies"[Mesh] OR "Longitudinal Studies"[Mesh] OR "Observational Study" [Publication Type] OR "Cross-Sectional Studies"[Mesh] OR "Epidemiologic Studies"[Mesh] OR "Case-Control Study"[tiab:~3] OR "Case Control Studies"[tiab:~3] OR "Retrospective Study"[tiab:~2] OR "Retrospective Studies"[tiab:~2] OR "Longitudinal Study"[tiab:~2] OR "Longitudinal Studies"[tiab:~2] OR "Cross-Sectional Study"[tiab:~3] OR "Cross-Sectional Studies"[tiab:~3] OR "observational study"[tiab:~2] OR "observational studies"[tiab:~2] OR "observational study"[tiab:~2] OR "observational studies"[tiab:~2] OR "epidemiology" [Subheading] OR "Child"[Mesh] OR "Adult Children"[Mesh] OR "Infant"[Mesh] OR "Men"[Mesh] OR "Women"[Mesh] OR "Female"[Mesh] OR "Adult"[Mesh] OR "Young Adult"[Mesh] OR "Aged"[Mesh] OR "Middle Aged"[Mesh] OR "Population Groups"[Mesh] OR "Persons"[Mesh] OR "Occupational Groups"[Mesh] OR "Prospective Studies"[Mesh])
7	#4 AND #5 AND #6
8	(rat OR rats OR mice OR mouse OR rodent OR rodents)
9	(Review[Publication Type] OR Systematic Review[Publication Type])
10	#7 NOT (#8 OR #9) Filter: English
**Scopus**	
1	TITLE-ABS-KEY ("artificial* sweet*" OR aspartame OR "Non-Nutritive Sweetener*" OR "Sweetening Agent*" OR "Artificially Sweetened Beverage*" OR "Sugar-Sweetened Beverage*" OR "sugar substitute*" OR "diet soda*" OR "diet beverage*" OR "diet drink*" OR "diet cola*" OR "sugar-free" OR "calorie-free" OR "non-calorically sweetened" OR "soft drink*" OR cola OR "low calorie sweet*" OR "non sugar sweet*" OR saccharin OR "Non-caloric artificial sweetener*" OR "sugar substitute*" OR "sugary drink*" OR acesulfame OR {ace K} OR saccharin OR neotame OR sucralose OR splenda OR "advantame" OR "Cyclamates" OR "alitame" OR "Neohesperidin" OR stevia OR steviol OR stevioside OR rebaudioside OR rebiana* OR thaumatin* OR brazzein* OR mogroside*)

Search Number	Query
2	TITLE-ABS-KEY (cancer* OR leukemia OR carcinogen* OR lymphoma OR myeloma OR neoplasm* OR carcinoma OR tumor OR tumors OR tumorous OR tumour OR sarcoma)
3	TITLE-ABS-KEY (human OR humans OR child* OR infant* OR boy OR girl OR adolescent* OR teenager* OR men OR women OR female OR male OR adult* OR elderly OR population OR "population group" OR "demographic group" OR epidemiolog* OR occupation* OR worker* OR cohort OR "case-control stud*" OR "case report" OR "retrospective stud*" OR "longitudinal stud*" OR "observational stud*" OR "cross-sectional stud*" OR "human stud*" OR persons)
4	TITLE-ABS-KEY (rat OR rats OR mice OR mouse OR rodent OR rodents OR animals)
5	#1 AND #2 AND #3
6	#5 AND NOT #4
7	#6 AND LIMIT-TO (LANGUAGE, "English")
8	#7 AND (EXCLUDE (DOCTYPE,"re")

Supplemental Table A.2 Criteria for Evaluation of Study Quality of Included Studies

Aspect	Criteria for Higher Quality (Strengths)	Criteria for Lower Quality (Weaknesses)
Intake Assessment	▪ Assessed specific NSS (*e.g.*, aspartame, sucralose)<br>▪ If specific NSS not noted, based on dates of approvals, key NSS can be deduced<br>▪ Used validated questionnaire<br>▪ Information collected at multiple timepoints (cohort)<br>▪ Information collected before cancer diagnosis<br>▪ Assessed major contributors of NSS (*e.g.*, whole diet, beverages)<br>▪ Considered frequency/duration/intake level of NSS consumption	▪ Not specific NSS type (*e.g.*, diet soda, artificially sweetened beverage, all NSS combined)<br>▪ Questionnaire not validated<br>▪ Information collected at one time point (cohort)<br>▪ Information not collected before cancer diagnosis<br>▪ Only considered minor source of NSS exposure (*e.g.*, tabletop sweeteners)<br>▪ Did not consider frequency, duration, or intake level of NSS consumption
Outcome Assessment	▪ Physician-diagnosed, or self-or proxy-reported and validated clinically, or recorded in medical records, death certificates, or registries<br>▪ Assessed disease incidence<br>▪ Sufficient time between exposure and outcome to account for disease latency (≥4 years soft tissue tumors, ≥0.5 yrs lympho/hematopoietic cancers)	▪ Self- or proxy-reported without validation<br>▪ Aggregated cancer outcome (*e.g.*, obesity related cancers)<br>▪ Assessed mortality only<br>▪ Insufficient consideration of disease latency (<4 years soft tissue tumors, <0.5 yrs lympho/hematopoietic cancers)<br>▪ Did not consider disease latency
Confounding/Covariate Consideration	▪ Proper model and forms of variables<br>▪ Considered key potential confounders for each cancer evaluated (controlled for or considered age, sex, and two additional covariates listed in Table 2; one of which must be smoking for bladder or lung cancers)<br>▪ Considered time-varying nature of relevant covariates (*e.g.*, body weight, diabetes) (cohort studies)	▪ Improper model, or forms of variables<br>▪ Failed to consider key potential confounders (*i.e.*, did not control for or consider age, sex, and two additional covariates listed in Table 2; one of which must be smoking for bladder or lung cancers)<br>▪ Did not consider time-varying nature of relevant covariates
Sample Selection	▪ Appropriate comparison groups<br>▪ Exclusion (c-c) or loss to follow-up (cohort) of <25% of participants or >75% enrollment<br>▪ Nondifferential (≤15%) participation rates (c-c study)	▪ Exclusion or loss to follow-up of >25% of participants or <75% enrollment<br>▪ Inappropriate comparison groups<br>▪ Differential (>15%) participation rates between cases and controls (c-c study)

Notes:
c-c = Case-Control; NSS = Non-Sugar Sweetener; Yr = Year.

Supplement B: PRISMA Checklist and Flow Diagram

Supplemental Table B.1 PRISMA Checklist

Section and Topic	Item #	Checklist Item	Location Where Item Is Reported
**TITLE**			
Title	1	Identify the report as a systematic review.	Pg. 1
**ABSTRACT**			
Abstract	2	See the PRISMA 2020 for Abstracts checklist.	Pg. 2-3
**INTRODUCTION**			
Rationale	3	Describe the rationale for the review in the context of existing knowledge.	Pg. 4-5
Objectives	4	Provide an explicit statement of the objective(s) or question(s) the review addresses.	Pg. 6
**METHODS**			
Eligibility criteria	5	Specify the inclusion and exclusion criteria for the review and how studies were grouped for the syntheses.	Section 2.1<br>Table 1
Information sources	6	Specify all databases, registers, websites, organisations, reference lists and other sources searched or consulted to identify studies. Specify the date when each source was last searched or consulted.	Section 2.1
Search strategy	7	Present the full search strategies for all databases, registers and websites, including any filters and limits used.	Supplemental Table A.1
Selection process	8	Specify the methods used to decide whether a study met the inclusion criteria of the review, including how many reviewers screened each record and each report retrieved, whether they worked independently, and if applicable, details of automation tools used in the process.	Section 2.2
Data collection process	9	Specify the methods used to collect data from reports, including how many reviewers collected data from each report, whether they worked independently, any processes for obtaining or confirming data from study investigators, and if applicable, details of automation tools used in the process.	Section 2.2
Data items	10a	List and define all outcomes for which data were sought. Specify whether all results that were compatible with each outcome domain in each study were sought (e.g. for all measures, time points, analyses), and if not, the methods used to decide which results to collect.	Sections 2.2
	10b	List and define all other variables for which data were sought (e.g. participant and intervention characteristics, funding sources). Describe any assumptions made about any missing or unclear information.	Section 2.2
Study risk of bias assessment	11	Specify the methods used to assess risk of bias in the included studies, including details of the tool(s) used, how many reviewers assessed each study and whether they worked independently, and if applicable, details of automation tools used in the process.	Section 2.3, Supplemental Table A.2
Effect measures	12	Specify for each outcome the effect measure(s) (e.g. risk ratio, mean difference) used in the synthesis or presentation of results.	Section 2.2

Section and Topic	Item #	Checklist Item	Location Where Item Is Reported
Synthesis methods	13a	Describe the processes used to decide which studies were eligible for each synthesis (e.g. tabulating the study intervention characteristics and comparing against the planned groups for each synthesis (item #5)).	Section 2
	13b	Describe any methods required to prepare the data for presentation or synthesis, such as handling of missing summary statistics, or data conversions.	N/A
	13c	Describe any methods used to tabulate or visually display results of individual studies and syntheses.	Section 2.2
	13d	Describe any methods used to synthesize results and provide a rationale for the choice(s). If meta-analysis was performed, describe the model(s), method(s) to identify the presence and extent of statistical heterogeneity, and software package(s) used.	Sections 2.2 and 2.4
	13e	Describe any methods used to explore possible causes of heterogeneity among study results (e.g. subgroup analysis, meta-regression).	Section 2
	13f	Describe any sensitivity analyses conducted to assess robustness of the synthesized results.	N/A
Reporting bias assessment	14	Describe any methods used to assess risk of bias due to missing results in a synthesis (arising from reporting biases).	N/A
Certainty assessment	15	Describe any methods used to assess certainty (or confidence) in the body of evidence for an outcome.	Section 2.3-2.4
**RESULTS**			
Study selection	16a	Describe the results of the search and selection process, from the number of records identified in the search to the number of studies included in the review, ideally using a flow diagram.	Supplemental Figure B.1
	16b	Cite studies that might appear to meet the inclusion criteria, but which were excluded, and explain why they were excluded.	N/A
Study characteristics	17	Cite each included study and present its characteristics.	Tables E.1 and E.2
Risk of bias in studies	18	Present assessments of risk of bias for each included study.	Supplemental Table E.3 and E.4
Results of individual studies	19	For all outcomes, present, for each study: (a) summary statistics for each group (where appropriate) and (b) an effect estimate and its precision (e.g. confidence/credible interval), ideally using structured tables or plots.	Supplements F-N
Results of syntheses	20a	For each synthesis, briefly summarise the characteristics and risk of bias among contributing studies.	Section 3.4<br>Table 4
	20b	Present results of all statistical syntheses conducted. If meta-analysis was done, present for each the summary estimate and its precision (e.g. confidence/credible interval) and measures of statistical heterogeneity. If comparing groups, describe the direction of the effect.	N/A
	20c	Present results of all investigations of possible causes of heterogeneity among study results.	Section 3.4 and Supplements F-N
	20d	Present results of all sensitivity analyses conducted to assess the robustness of the synthesized results.	N/A
Reporting biases	21	Present assessments of risk of bias due to missing results (arising from reporting biases) for each synthesis assessed.	N/A
Certainty of evidence	22	Present assessments of certainty (or confidence) in the body of evidence for each outcome assessed.	Section 3.4

Section and Topic	Item #	Checklist Item	Location Where Item Is Reported
**DISCUSSION**			
Discussion	23a	Provide a general interpretation of the results in the context of other evidence.	Section 4.3
	23b	Discuss any limitations of the evidence included in the review.	Section 4.1-4.2
	23c	Discuss any limitations of the review processes used.	Section 4.5
	23d	Discuss implications of the results for practice, policy, and future research.	Section 4.4
**OTHER INFORMATION**			
Registration and protocol	24a	Provide registration information for the review, including register name and registration number, or state that the review was not registered.	Pg. 6
	24b	Indicate where the review protocol can be accessed, or state that a protocol was not prepared.	Pg. 6
	24c	Describe and explain any amendments to information provided at registration or in the protocol.	N/A
Support	25	Describe sources of financial or non-financial support for the review, and the role of the funders or sponsors in the review.	Pg. 1
Competing interests	26	Declare any competing interests of review authors.	TBD – created by journal at submission.
Availability of data, code and other materials	27	Report which of the following are publicly available and where they can be found: data extracted from included studies	Supplemental materials

From: Page MJ, McKenzie JE, Bossuyt PM, Boutron I, Hoffmann TC, Mulrow CD, et al. The PRISMA 2020 statement: an updated guideline for reporting systematic reviews. BMJ 2021;372:n71. doi: 10.1136/bmj.n71. For more information, visit: http://www.prisma-statement.org/

Supplemental Figure B.1 PRISMA Flow Diagram

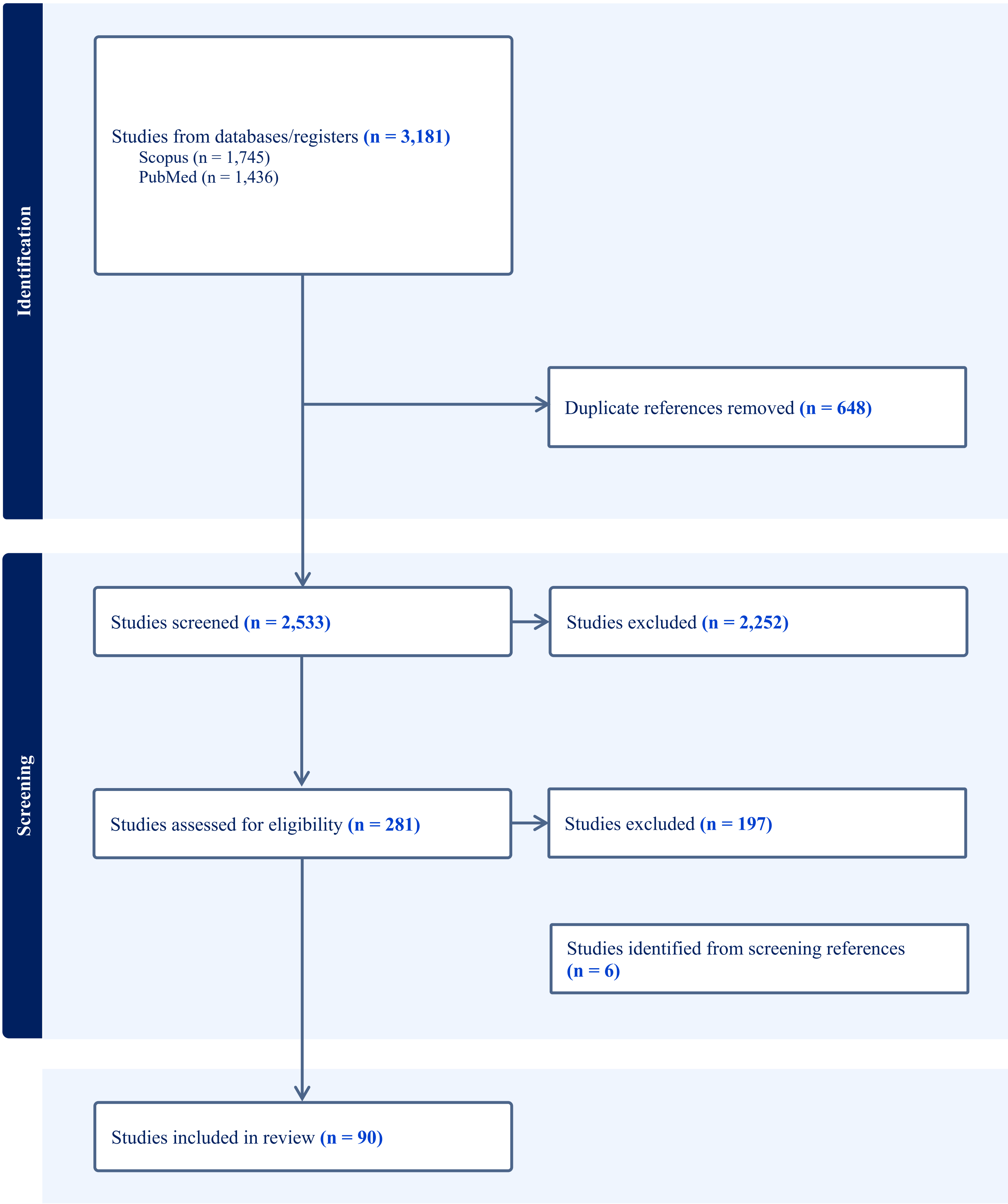

Supplement C: Overview of Included Studies

Supplemental Table C.1 NSS and Cancers Evaluated in Included Cohort Studies

Study	Population	Sweetener Types					Cancer Types																								
		Non-specific	Aspartame	Ace-K	Sucralose	Saccharin	Breast	Uterine	Ovarian	Lung	Oral/Pharyngeal/Laryngeal	Esophageal	Stomach	Intestinal	Colorectal	Pancreas	Gallbladder	Liver	Bladder	Kidney	Aggregate Urinary System	Leukemia	Hodgkin's Lymphoma	NHL	MM	Thyroid	Aggregate Lymphohematopoietic System	Prostate	Brain	Skin	Aggregate Cancer
Bao *et al*. (30)	NIH-AARP	✓														✓															
Bassett *et al*. (111)	MCCS	✓																													✓
Chazelas *et al*. (83)	NutriNet-Santé	✓					✓*								✓													✓*			✓
Debras *et al*. (20)	NutriNet-Santé	✓	✓	✓	✓		✓																					✓			✓
Fulgoni and Drewnowski (2022)[a]	NHANES	✓	✓			✓																									✓
Gao *et al*. (2024)[a]	NHANES					✓																									✓
Heath *et al*. (74)	EPIC	✓																		✓											
Hodge *et al*. (44)	MCCS	✓					✓	✓	✓				✓		✓					✓								✓			✓
Hur *et al*. (82)	NHS II	✓													✓																
Inoue-Choi *et al*. (97)	IWHS	✓						✓																							
Jones *et al*. (25)	NIH-AARP/PLCO	✓																✓													
Lee *et al*. (24)	NHS/HPFS	✓																		✓											
Lim *et al*. (95)	NIH-AARP		✓																			✓	✓	✓	✓		✓		✓		
Liu *et al*. (18)	UK Biobank	✓																													✓
McCullough *et al*. (21)	CPS-II Nutrition	✓	✓																					✓	✓						
McCullough *et al*. (26)	CPS-II	✓					✓	✓	✓	✓	✓	✓	✓	✓	✓	✓	✓	✓	✓	✓		✓		✓*	✓*			✓	✓	✓	✓
Mullee *et al*. (27)	EPIC	✓					✓								✓													✓			✓
Navarrete-Muñoz *et al*. (28)	EPIC	✓														✓															
Ringel *et al*. (31)	WHI	✓																	✓	✓	✓										
Romanos-Nanclares *et al*. (29)	NHS/NHS-II	✓					✓																								
Romanos-Nanclares *et al*. (22)	NHS		✓				✓																								
Schernhammer *et al*. (89)	NHS/HPFS	✓														✓															
Schernhammer *et al*. (23)	NHS/HPFS	✓	✓																			✓		✓	✓						

		Sweetener Types					Cancer Types																								
Study	Population	Non-specific	Aspartame	Ace-K	Sucralose	Saccharin	Breast	Uterine	Ovarian	Lung	Oral/Pharyngeal/Laryngeal	Esophageal	Stomach	Intestinal	Colorectal	Pancreas	Gallbladder	Liver	Bladder	Kidney	Aggregate Urinary System	Leukemia	Hodgkin's Lymphoma	NHL	MM	Thyroid	Aggregate Lymphohematopoietic System	Prostate	Brain	Skin	Aggregate Cancer
Stepien *et al*. (49)	EPIC	✓																✓													
Wang *et al*. (81)	HPFS/NHS/NHS-II	✓													✓																
You *et al*. (19)	PLCO	✓								✓																					✓
Zamora-Ros *et al*. (2023)[a]	EPIC	✓																								✓					
Zhang *et al*. (2021)[a]	NHANES	✓																													✓
Zhang *et al*. (2024)[a]	UK Biobank	✓																													✓
Zhao *et al*. (48)	WHI	✓																✓													

Notes:

Ace-K = Acesulfame Potassium; CPS = Cancer Prevention Study; EPIC = European Prospective Investigation into Cancer and Nutrition; HPFS + Health Professionals Follow-up Study; IWHS = Iowa Women's Health Study; MCCS = Melbourne Collaborative Cohort Study; MM = Multiple Myeloma; NHANES = National Health and Nutrition Examination Survey; NHS = Nurses' Health Study; NIH-AARP = National Institutes of Health American Association of Retired Persons Diet and Health Study; NHL = Non-Hodgkin's Lymphoma; NSS = Non-Sugar Sweetener; PLCO = Prostate, Lung, Colorectal and Ovarian Cancer Screening Trial; UK = United Kingdom; WHI = Women's Health Initiation Observational Study.

✓ indicates that sweetener or cancer type was examined in that study.

* = Results are not tabulated in this review because another study provided a more recent or a higher quality analysis of this outcome in an overlapping population.

(a) These studies are not cited in the main manuscript, but rather only in the supplements. The full references for these studies are listed at the end of the supplements.

Supplemental Table C.2 NSS and Cancers Evaluated in Case-Control Studies

	Sweetener Types						Cancer Types																							
**Study**	**Non-specific**	**Aspartame**	**Ace-K**	**Cyclamate**	**Sucralose**	**Saccharin**	**Breast**	**Uterine**	**Ovarian**	**Lung**	**Oral/Pharyngeal/Laryngeal**	**Esophageal**	**Stomach**	**Intestinal**	**Colorectal**	**Pancreas**	**Gallbladder**	**Bladder**	**Kidney**	**Aggregate Urinary System**	**Leukemia**	**Hodgkin's Lymphoma**	**NHL**	**MM**	**Thyroid**	**Aggregate Lymphohematopoietic System**	**Prostate**	**Brain**	**Skin**	**Aggregate Cancer**
Akdaş *et al.* (50)	✓																	✓												
Andreatta *et al.* (32)	✓																			✓										
Asal *et al.* (77)	✓																		✓											
Bosetti *et al.* (80)	✓					✓		✓					✓			✓														
Bruemmer *et al.* (51)	✓																	✓												
Bunin *et al.* (98)	✓																											✓		
Cabaniols *et al.* (100)		✓																										✓		
Cartwright *et al.* (43)						✓												✓												
Chan *et al.* (90)	✓															✓														
Chang *et al.* (85)	✓														✓															
Davis *et al.* (91)	✓															✓														
Ewertz and Gill (45)	✓						✓																							
Franceschi *et al.* (84)	✓														✓															
Gallus *et al.* (46)	✓					✓	✓		✓		✓	✓			✓				✓								✓			
Gold *et al.* (92)	✓															✓														
Goodman *et al.* (75)	✓					✓													✓											
Gurney *et al.* (99)		✓																										✓		
Hardell *et al.* (101)		✓																										✓		
Hoover and Strasser (42)	✓																	✓												
Howe *et al.* (52)	✓					✓												✓												
Howe *et al.* (53)	✓					✓												✓												
Ibiebele *et al.* (78)	✓											✓																		
Iscovich *et al.* (54)						✓												✓												
Kantor *et al.* (56)	✓																	✓												
Kantor *et al.* (55)	✓																	✓												
Kessler and Clark (57)	✓			✓		✓												✓												
Kobeissi *et al.* (58)	✓																	✓												
Li *et al.* (96)	✓																				✓									
Maclure and Willett (76)	✓																		✓											
Mahfouz *et al.* (86)	✓														✓															
Marrett *et al.* (59)	✓																	✓												
Mayne *et al.* (79)	✓											✓	✓																	
Mettlin (102)	✓									✓																				
Moller-Jensen *et al.* (41)	✓			✓		✓												✓												

Study	Sweetener Types						Cancer Types																							
	Non-specific	Aspartame	Ace-K	Cyclamate	Sucralose	Saccharin	Breast	Uterine	Ovarian	Lung	Oral/Pharyngeal/Laryngeal	Esophageal	Stomach	Intestinal	Colorectal	Pancreas	Gallbladder	Bladder	Kidney	Aggregate Urinary System	Leukemia	Hodgkin's Lymphoma	NHL	MM	Thyroid	Aggregate Lymphohematopoietic System	Prostate	Brain	Skin	Aggregate Cancer
Momas *et al*. (60)						✓												✓												
Mommsen *et al*. (61)						✓												✓												
Morgan and Jain (62)	✓																	✓												
Morrison and Buring (34)	✓																			✓										
Morrison (33)	✓																													✓
Morrison *et al*. (35)	✓																			✓										
Najem *et al*. (63)	✓					✓												✓												
Nomura *et al*. (36)	✓					✓														✓										
Norell *et al*. (94)	✓															✓														
Ohno *et al*. (64)	✓																	✓												
Palomar-Cros *et al*. (47)	✓	✓				✓	✓						✓		✓						✓						✓			
Piper *et al*. (65)	✓																	✓												
Risch *et al*. (66)	✓			✓		✓												✓												
Schulte *et al*. (67)	✓																	✓												
Silverman *et al*. (37)	✓																			✓										
Simon *et al*. (38)				✓		✓														✓										
Singh *et al*. (2020)[a]	✓																								✓					
Sturgeon *et al*. (68)	✓																	✓												
Sullivan (69)	✓																	✓												
Theodoratou *et al*. (87)	✓														✓															
Wang *et al*. (70)	✓																	✓												
Wu *et al*. (88)	✓													✓																
Wynder and Goldsmith (40)						✓												✓												
Wynder and Stellman (71)						✓												✓												
Wynder *et al*. (93)						✓										✓														
Yu *et al*. (39)						✓												✓												

Notes:
Ace-K = Acesulfame Potassium; MM = Multiple Myeloma; NHL = Non-Hodgkin's Lymphoma; NSS = Non-Sugar Sweetener.
✓ indicates that sweetener or cancer type was examined in that study.
(a) This study is not cited in the main manuscript, but rather only in the supplements. The full reference for this study is listed at the end of the supplements.

Supplement D: Cohorts Examined in Included Studies

Supplemental Table D.1 Cohorts Examined in Included Studies

Cohort	Study
Cancer Prevention Study-II (CPS-II) Nutrition Cohort	McCullough *et al*. (2014) (21)
	McCullough *et al*. (2022) (26)
European Prospective Investigation into Cancer and Nutrition (EPIC)	Heath *et al*. (2021) (74)
	Mullee *et al*. (2019) (27)
	Navarrete-Muñoz *et al*. (2016) (28)
	Stepien *et al*. (2016) (49)
	Zamora-Ros *et al*. (2023)[a]
Health Professionals Follow-up Study (HPFS)	Lee *et al*. (2006) (24)
	Schernhammer *et al*. (2005) (89)
	Schernhammer *et al*. (2012) (23)
	Wang *et al*. (2022) (81)
Iowa Women's Health Study (IWHS)	Inoue-Choi *et al*. (2013) (97)
Melbourne Collaborative Cohort Study (MCCS)	Bassett *et al*. (2020) (111)
	Hodge *et al*. (2018) (44)
National Health and Nutrition Examination Survey (NHANES)	Fulgoni and Drewnowski (2022)[a]
	Gao *et al*. (2024)[a]
	Zhang *et al*. (2021)[a]
National Institutes of Health American Association of Retired Persons (NIH-AARP) Diet and Health Study	Bao *et al*. (2008) (30)
	Jones *et al*. (2022) (25)
	Lim *et al*. (2006) (95)
Nurses' Health Study (NHS)	Lee *et al*. (2006) (24)
	Romanos-Nanclares *et al*. (2024) (22)
	Romanos-Nanclares *et al*. (2021) (29)
	Schernhammer *et al*. (2005) (89)
	Schernhammer *et al*. (2012) (23)
	Wang *et al*. (2022) (81)
Nurses' Health Study II (NHS-II)	Hur *et al*. (2021) (82)
	Romanos-Nanclares *et al*. (2021) (29)
	Wang *et al*. (2022) (81)
NutriNet-Santé	Chazelas *et al*. (2019) (83)
	Debras *et al*. (2022) (20)
Prostate, Lung, Colorectal and Ovarian (PLCO) Cancer Screening Trial	Jones *et al*. (2022) (25)
	You *et al*. (2022) (19)
United Kingdom (UK) Biobank	Liu *et al*. (2022) (18)
	Zhang *et al*. (2024)[a]
Women's Health Initiative (WHI) Observational Study	Ringel *et al*. (2023) (31)
	Zhao *et al*. (2023) (48)

Note:
(a) These studies are not cited in the main manuscript, but rather only in the supplements. The full references for these studies are listed at the end of the supplements.

Supplement E: Study Characteristics and Quality

Supplemental Table E.1 NSS and Cancer Cohort Study Characteristics

Study	Location	Study Population				Exposure Characterization				Cancer Outcome			
		Population	Sex	Age (yrs)[a]	N	Exposure Period	NSS	Source	Exposure Ascertainment	Outcome Type	Cancer Type	Outcome Ascertainment	Follow-Up Period
Bao *et al.* (2008) (30)	US	NIH-AARP Diet and Health Study	B	50-71	487,922	1994-1996	Non-specific	Diet soft drinks	FFQ on usual dietary intake prior yr	Inc	Pancreatic (primary adenocarcinoma of exocrine pancreas)[b]	State cancer registries and NDI	1995-2003
Bassett *et al.* (2020) (111)	Australia	MCCS	B	27-76	35,109	1989-1993	Non-specific	Diet (AS) soft drinks	FFQ, prior 12 mos	Inc	Obesity and non-obesity related cancers[c]	Victorian Cancer Registry, Australian Cancer Database, Victorian death records, NDI, and Australian Bureau of Statistics	1990-2015
Chazelas *et al.* (2019) (83)	France	NutriNet-Santé	B	$\bar{x}$ = 42.2	101,257	2009-2018	Non-specific	ASBs (*e.g.*, diet soft drinks, sugar-free syrups, and diet milk-based beverages)	24-hr dietary records (3x over 2 wks, every 6 mos)[d]	Inc	All cancers and colorectal	Self-reported, verified by medical records, physicians, and/or hospitals, with linkage to national health insurance system and mortality registry	2009-2018
Debras *et al.* (2022) (20)	France	NutriNet-Santé	B	$\bar{x}$ = 42.2	102,865	2009-2021	Aspartame, acesulfame-K, sucralose, and total AS[e]	Whole diet (*e.g.*, soft drinks, TT, yogurt/ cottage cheese)	24-hr dietary records (3x over 2 wks, every 6 mos)[d]	Inc	All cancers and obesity-related cancers[f]	Self-reported, verified by medical records, physicians and/or hospitals, with linkage to national health insurance system and mortality registry	2009-2021
			M	>18	22,154						Prostate		
			F		80,711						Breast		

Study	Location	Study Population				Exposure Characterization				Cancer Outcome			
		Population	Sex	Age (yrs)[a]	N	Exposure Period	NSS	Source	Exposure Ascertainment	Outcome Type	Cancer Type	Outcome Ascertainment	Follow-Up Period
Fulgoni and Drewnowski (2022)[g]	US	NHANES	B	x̄ = 44.52	15,948	1988-1994	Aspartame, saccharin, or non-specific	Whole diet (*e.g.*, soft drinks, TT, foods)	24-hr dietary recalls linked to the Food and Nutrient Database for Dietary Studies	Mort	All cancers	2019 Public-Use Linked Mortality Files	1988-1994
				x̄ = 46.53	64,702	1988-2018	Non-specific						1988-2018
Gao *et al*. (2024)[g]	US	NHANES	B	x̄ = 56.30-57.81	936	1988-1994	Saccharin	Whole diet	24-hr dietary recall	Mort	All cancers	NDI	1988-2019
Heath *et al*. (2021) (74)	Europe	EPIC[h]	B	30-70	281,483	1990-2000	Non-specific	AS soft drinks	Diet questionnaire on past yr	Inc & Mort	RCC	Population registries and active follow-up	–
Hodge *et al*. (2018) (44)	Australia	MCCS	B	40-69	35,593	1989-1993[i]	Non-specific	Diet soft drinks	FFQ past yr	Inc	Obesity-related cancer,[j] kidney, colorectal, and gastric cardia	Victorian Cancer Registry, Australian Cancer Database, NDI, and Australian Bureau of Statistics	1990-2013
			F		21,492						Ovarian, endometrial, and post-menopausal breast		
			M		14,101						Prostate		
Hur *et al*. (2021) (82)	US	NHSII	F	25-42	95,464	1990-2014	Non-specific	Low-calorie carbonated beverages	FFQ on past 12 mos, every 4 yrs	Inc	EO-CRC	Self-reported, NDI, tumor registries, or death certificates; confirmed by medical records	1991-2015
Inoue-Choi *et al*. (2013) (97)	US	IWHS	F[k]	x̄ = 61.6	23,039	1985-1986	Non-specific	Sugar-free soft drinks	FFQ past 12 mos	Inc	Type 1 and 2 endometrial	State Health Registry of Iowa/SEER and NDI	1986-2010

Study	Location	Study Population				Exposure Characterization				Cancer Outcome			
		Population	Sex	Age (yrs)[a]	N	Exposure Period	NSS	Source	Exposure Ascertainment	Outcome Type	Cancer Type	Outcome Ascertainment	Follow-Up Period
Jones *et al*. (2022) (25)	US	NIH-AARP	B	50-71	553,874	1995-1996	Non-specific	AS or diet beverages	Questionnaire	Inc	Liver	State cancer registries	1995-2011
		PLCO		55-74		1998						Self-report confirmed through medical records and/or linkage to state cancer registries	1998-2017
Lee *et al*. (2006) (24)	US	NHS	F	34-59	88,759	1979-1998	Non-specific	AS sodas	Semi-quantitative FFQ on past 12 mos, about every 4 yrs	Inc	RCC	Self/proxy-report confirmed by medical records or NDI	1980-2000
		HPFS	M	40-75	47,828	1985-1998							1986-2000
Lim *et al*. (2006) (95)	US	NIH-AARP Diet and Health Study	B	50-71	473,984	1994-1996	Aspartame	Beverages (*i.e.*, soda, fruit drinks, sweetened iced tea, and aspartame added to hot coffee and tea)	FFQ past 12 mos	Inc	All hematopoietic cancers, malignant gliomas, HL, MM, lymphoid malignancies, NHL, immunoblastic lymphoma and lymphoblastic lymphoma/ leukemia, non-lymphoid leukemia	State cancer registries	1995-2000
Liu *et al*. (2022) (18)	UK	UK Biobank	B	x̄ = 55.6	171,616	2009-2012	Non-specific	AS coffee	Web-based 24-hr dietary recall questionnaire (Oxford WebQ) up to 5 times during 1-yr period	Mort	All cancers	Death certificates *via* National Health Service Information Centre (England and Wales) and National Health Service Central Register Scotland (Scotland)	England and Wales: 2009-2018<br><br>Scotland: 2009-2017

Study	Location	Study Population				Exposure Characterization				Cancer Outcome			
		Population	Sex	Age (yrs)[a]	N	Exposure Period	NSS	Source	Exposure Ascertainment	Outcome Type	Cancer Type	Outcome Ascertainment	Follow-Up Period
McCullough *et al*. (2014) (21)	US	CPS-II Nutrition Cohort	B	x̄ = 69.2	100,442	1998 and 2002	Non-specific or aspartame	AS carbonated beverages +/- TT packets[l]	Self-reported mean consumption in past yr from 1999 and 2003 FFQs	Inc	MM and NHL	Self-reported or NDI, verified by medical records or linkage with state cancer registry	1999-2009
McCullough *et al*. (2022) (26)	US	CPS-II	B	M: 28-90; F: 28-95	934,777; M: 416,313; F: 518,464[m]	1972-1982	Non-specific	ASBs (*i.e.*, diet sodas and diet iced teas)	Questionnaire at enrollment	Mort	All cancers, obesity-related,[n] laryngeal/oral cavity/pharyngeal, esophageal, stomach, liver, pancreatic, gall bladder, lung, small intestinal, colorectal, post-menopausal breast, uterus, ovarian, prostate, kidney, bladder, melanoma, brain, NHL, MM, and leukemia	Personal inquiry validated by death certificates, and NDI	1982-2016
Mullee *et al*. (2019) (27)	Europe	EPIC	B	x̄ = 50.8	451,743	1992-2000	Non-specific	AS soft drinks	Self-administered questionnaire or in-person interview	Mort	All cancer and colorectal	Cancer registries, boards of health, death indices, municipal registries, regional health departments, and physicians/ hospitals	1992-2013 (end of follow-up was 2008-2013 depending on country)
			F	–	321,081						Breast		
			M		130,662						Prostate		

Study	Location	Study Population				Exposure Characterization				Cancer Outcome			
		Population	Sex	Age (yrs)[a]	N	Exposure Period	NSS	Source	Exposure Ascertainment	Outcome Type	Cancer Type	Outcome Ascertainment	Follow-Up Period
Navarrete-Muñoz *et al*. (2016) (28)	Europe	EPIC[h]	B	x̄ = 51	477,199	1991-2000	Non-specific	AS soft drinks	Dietary questionnaire on prior 12 mos, linked to EPIC Nutrient Database	Inc	Pancreatic (adenocarcinoma of the exocrine pancreas)	Cancer, pathology and mortality registries, self/proxy-report (confirmed by review of medical records), and health insurance records	1992-2009 (end of follow-up was 2004-2009 depending on country)
Ringel *et al*. (2022) (31)	US	WHI-OS	F	x̄ = 66.6	80,388	1996-2001	Non-specific	ASBs (*i.e.*, diet drinks)	Questionnaire on past 3 mos at 3 yr follow-up visit	Inc	Urinary tract, bladder, and kidney	Self-reported and confirmed by physicians	1996-2020
Romanos-Nanclares *et al*. (2021) (29)	US	NHS	F	30-55	82,713	1979-2016	Non-specific	ASB	FFQ of prior yr in 1980, 1984, 1986, and every 4 yrs thereafter	Inc	Breast	Self- or proxy-report, NDI, and confirmed by medical records	1980-2016
		NHSII		25-42	93,085	1990-2017			FFQ of prior yr in 1991 and every 4 yrs thereafter				1991-2017
Romanos-Nanclares *et al*. (2024) (22)	US	NHS	F	–	–	1983-2018	Aspartame	Diet soda and TT	FFQ of prior yr in 1984 and every 2-4 yrs thereafter	Inc	Breast (invasive)	Self- or proxy-report and confirmed by medical records or NDI	1984-2018
		NHSII				1990-2019			FFQ of prior yr in 1991 and every 2-4 yrs thereafter				1991-2019

Study	Location	Study Population				Exposure Characterization				Cancer Outcome			
		**Population**	**Sex**	**Age (yrs)[a]**	**N**	**Exposure Period**	**NSS**	**Source**	**Exposure Ascertainment**	**Outcome Type**	**Cancer Type**	**Outcome Ascertainment**	**Follow-Up Period**
Schernhammer *et al*. (2005) (89)	US	NHS	F	30-55	88,794	1979-2000	Non-specific	Diet soft drinks	FFQ, of prior 12 mos, about every 4 yrs	Inc	Pancreatic	Self-reported or NDI, verified by medical record, physician, death certificate, or family member	1980-2000
		HPFS	M	40-75	49,364	1985-2000							1986-2000
Schernhammer *et al*. (2012) (23)	US	NHS	F	30-55	77,218	1983-2006 (diet soda)<br><br>1993-2006 (aspartame)	Non-specific or aspartame	Diet soda +/- TT packets[l]	FFQ of prior 12 mos, about every 4 yrs	Inc	Leukemia, MM, NHL	Self/proxy-reported, cancer registries, or NDI and verified by medical records	1984-2006 (diet soda)<br><br>1994-2006 (aspartame)
		HPFS	M	40-75	47,810	1985-2006 (diet soda)<br><br>1993-2006 (aspartame)							1986-2006 (diet soda)<br><br>1994-2006 (aspartame)
Stepien *et al*. (2016) (49)	Europe	EPIC	B	Cases: $\bar{x}$ = 59.6; non-cases: $\bar{x}$ = 51.2	424,123	1991-1998	Non-specific	AS drinks	FFQ prior 12 mos	Inc	HCC	National cancer registries, national health insurance records, cancer or pathology registries, and active follow-up	1992-2010

Study	Location	Study Population				Exposure Characterization				Cancer Outcome			
		Population	Sex	Age (yrs)[a]	N	Exposure Period	NSS	Source	Exposure Ascertainment	Outcome Type	Cancer Type	Outcome Ascertainment	Follow-Up Period
Wang *et al.* (2022) (81)	US	HPFS	M	40-75	46,341	1986-2014	Non-specific	ASBs	FFQs every 4 yrs	Inc	Colorectal	Questionnaire every 2 yrs (self or proxy-reported) and confirmed by physicians, medical records, pathology reports, NDI, and death certificates	1986-2014
		NHS	F	30-55	67,425	1986-2014							1986-2014
		NHS II		25-42	92,482	1991-2015							1991-2015
You *et al.* (2022) (19)	US	PLCO Cancer Screening Trial	B	55-74	97,133	–	Non-specific	Diet soft drinks	DHQ (*i.e.*, FFQ) at enrollment	Inc	Lung and all cancers	Annual self-reported questionnaire, confirmed by physicians, medical records, or state registries	1994-2009 (max 10-yr follow-up)
Zamora-Ros *et al*. (2023)[g]	Europe	EPIC[o]	B	x̄ = 51.1	450,064	1991-2000	Non-specific	ASBs	Center/country-specific dietary questionnaires of prior yr	Inc	All thyroid, papillary thyroid, and follicular thyroid	National and regional registries, health insurance records, cancer and pathology registries, and active follow-up	–
Zhang *et al*. (2021)[g]	US	NHANES	B	≥20	31,402	1999-2014	Non-specific	ASBs	24-hr dietary recall interviews	Mort	All cancers	NDI	1999-2015
Zhang *et al*. (2024)[g]	UK	UK Biobank	B	x̄ = 55.0	58,098	2009-2012	Non-specific	AS cereal	24-hr dietary recall interviews	Mort	All cancers	NHS Information Centre and Central Register Scotland	2006-2023

Study	Location	Study Population				Exposure Characterization				Cancer Outcome			
		Population	Sex	Age (yrs)[a]	N	Exposure Period	NSS	Source	Exposure Ascertainment	Outcome Type	Cancer Type	Outcome Ascertainment	Follow-Up Period
Zhao *et al*. (2023) (48)	US	WHI-OS	F	50-79	64,787	1995-2001	Non-specific	Diet drinks or diet fruit drinks	Self-administered FFQ on past 3 mos consumption	Inc	Liver	Self-administered questionnaire verified through medical reports and death certificates	1996-2020

Notes:

AARP = American Association of Retired Persons; AS = Artificial Sweetener/Artificially Sweetened; ASB = Artificially Sweetened Beverage; B = Both Males and Females; CPS = Cancer Prevention Study; DHQ = Dietary History Questionnaire; EO-CRC = Early-Onset Colorectal Cancer; EPIC = European Prospective Investigation into Cancer and Nutrition; F = Females; FFQ = Food Frequency Questionnaire; HCC = Hepatocellular Carcinoma; HL = Hodgkin's Lymphoma; HPFS = Health Professionals Follow-Up Study; hr = Hour; Inc = Incidence; IWHS = Iowa Women's Health Study; M = Males; MCCS = Melbourne Collaborative Cohort Study; MM = Multiple Myeloma; Mort = Mortality; mo = Month; N = Sample Size; NDI = National Death Index; NHS = National Health Service; NHANES = National Health and Nutrition Examination Survey; NHL = Non-Hodgkin's lymphoma; NHS = Nurses' Health Study; NIH = National Institutes of Health; NSS = Non-Sugar Sweetener; PLCO = Prostate, Lung, Colorectal, and Ovarian Cancer Screening Trial; RCC = Renal Cell Carcinoma; SEER = Surveillance, Epidemiology, and End Results; TT = Tabletop; UK = United Kingdom; US = United States; WHI-OS = Women's Health Initiative Observational Study; wk = Week; yr = Year.

$\bar{x}$ = Mean; – = Not Reported.

(a) Age at baseline unless otherwise noted.

(b) Bao *et al*. (30) excluded endocrine pancreatic tumors.

(c) Obesity-related cancers in this study were defined as follows: "13 cancer types (esophagus (adenocarcinoma); pancreas; colorectum; breast (postmenopausal women); endometrium; kidney; ovary; gallbladder; liver; gastric cardia; meningioma; thyroid; and MM) identified in 2016 by IARC for which there was sufficient evidence to be linked to overweight or obesity (Lauby-Secretan, 2016). All other confirmed cancers were defined as not related to obesity" (111).

(d) Chazelas *et al*. (83) and Debras *et al*. (20) report that 3 surveys were to be collected in a 2 wk period every 6 mo for the study duration. Neither study reports how complete follow-up data are and, in the 2 yr baseline period, both studies report that most participants only completed 2 or 3 surveys out of 15.

(e) Total AS equaled the sum of acesulfame-K, aspartame, cyclamates, saccharin, sucralose, thaumatin, neohesperidine dihydrochalcone, steviol glycosides, and salt of aspartame-acesulfame (20).

(f) Obesity-related cancers in this study were colorectal, stomach, liver, mouth, pharynx, larynx, esophageal, breast (with opposite associations pre- and post-menopause), ovarian, endometrial, and prostate cancers (20).

(g) These studies are not cited in the main manuscript, but rather only in the supplements. The full references for these studies are listed at the end of this supplement.

(h) Heath *et al*. (74) and Navarrete-Muñoz *et al*. (28) did not include EPIC centers in Italy and Spain, or one center in Sweden.

(i) A second wave of data collection occurred in 2003-2007; however, this data was not used for analyses involving AS soft drinks (44).

(j) Obesity-related cancers defined as cancers of the liver, prostate, ovary, gallbladder, kidney, colorectal, esophagus, postmenopausal breast, pancreas, endometrium, and stomach (44).

(k) Only post-menopausal women (97).

(l) TT aspartame packets specified as NutraSweet or Equal manufactured by the NutraSweet Corporation (formerly Searle and Co.) (21, 23).

(m) Women who reported hysterectomy (n = 132,292), uterine surgery (n = 6,056), or surgically induced menopause (n = 31,803) were excluded from uterine cancer analyses. Women who reported a history of oophorectomy (n = 10,162) were excluded from ovarian cancer analyses. Breast cancer analyses were restricted to post-menopausal women at baseline (n = 367,978) (26).

(n) Obesity-related cancers defined as cancers of the esophagus, stomach, colorectum, liver, gallbladder, pancreas, post-menopausal breast, uterus/endometrium, ovary, kidney, and MM (26).

(o) Zamora-Ros *et al*. (2023) did not include EPIC centers in Greece.

Supplemental Table E.2 NSS and Cancer Case-Control Study Characteristics

Study	Location	Study Population						Exposure Characterization				Cancer Outcome		
		Sex	Age (yrs)	Cases		Controls								
				N	Source	N	Source	Exposure Period	NSS	Source	Exposure Ascertainment	Cancer Type	Outcome Type	Study Period
Akdaş *et al*. (1990) (50)	Turkey	B	x̄ = 60	194	Hospitals	194	Hospitals	–	Non-specific	AS/TT[a]	Self-reported *via* interview	Bladder	Inc	1980-1987
Andreatta *et al*. (2008) (32)	Argentina	B	Median = 56-65	197	Hospitals	397	Hospitals	1994-2006	Saccharin, cyclamate, aspartame, and acesulfame-K combined	TT	Self-reported *via* interview and FFQ of 5 yrs before diagnosis or hospitalization	Urinary tract[b]	Inc	1999-2006
Asal *et al*. (1988) (77)	US	B	Cases: x̄ = 60 (M) 62 (F)<br><br>Controls: x̄ = 58 (M) 61 (F)	315	Hospitals	336	RDD	–	Saccharin and cyclamate	TT	Self-reported *via* interview and questionnaire	RCC	Inc	1981-1984
Bosetti *et al.* (2009) (80)	Italy	F	Cases Median = 60 Controls Median = 61	454	Hospitals	908	Hospitals (acute, non-neoplastic admissions)	1990-2006	AS (mainly aspartame) or saccharin	TT	FFQ on consumption 2 yrs prior to diagnosis/ hospitalization	Endometrial	Inc	1992-2006
		B	Median = 63	326		652		1989-2007				Pancreatic		1991-2007
			Median = 63	230		547		1995-2007				Stomach		1997-2007

Study	Location	Study Population						Exposure Characterization				Cancer Outcome		
		Sex	Age (yrs)	Cases		Controls								
				N	Source	N	Source	Exposure Period	NSS	Source	Exposure Ascertainment	Cancer Type	Outcome Type	Study Period
Bruemmer *et al*. (1997) (51)	US	B	Cases x̄ = 58.3 Controls x̄ = 57.1	262	SEER	405	RDD	1975-1988	Non-specific	Diet soft drinks	Self-reported *via* interview, reported consumption during the 10-yr period ending at the reference date (*i.e*., 2 yrs prior to diagnosis)	Bladder	Inc	1987-1990
Bunin *et al*. (2005) (98)	US	B	Cases x̄ = 4.9 Controls x̄ = 5.6	315	COG register	315	RDD	Periconception and second trimester	Non-specific[c]	Diet soda	Self-reported maternal diet *via* phone interview and FFQ	Childhood medulloblastoma/ PNET	Inc	1991-1997
Cabaniols *et al*. (2011) (100)	France	B	Cases: x̄ = 57 Controls: –	122	Hospitals	122	Hospitals (non-cancer neurosurgical department)	2000-2005	Aspartame	–	Self-reported *via* questionnaire and interview, frequency of intake prior 5 yrs	MPBT	Inc	2005
Cartwright *et al*. (1981) (43)	UK	B	–	841	Hospital	1060	Hospital		Saccharin	TT	Self-reported *via* interview and questionnaire	Bladder	Inc	–
Chan *et al*. (2009) (90)	US	B	Median = 60-69	532	Northern CA Cancer Center and UCSF clinics	1,701	RDD and HCFA lists	1994-1999	Non-specific	Total sugar-free carbonated beverages, and low-calorie colas or carbonated beverages	In-person interview and FFQ of 1 yr prior to diagnosis (cases) or interview (controls)	Adeno-carcinoma of exocrine pancreas	Inc	1995-1999

Study	Location	Study Population						Exposure Characterization				Cancer Outcome		
		Sex	Age (yrs)	Cases		Controls								
				N	Source	N	Source	Exposure Period	NSS	Source	Exposure Ascertainment	Cancer Type	Outcome Type	Study Period
Chang *et al*. (2021) (85)	Canada	B	Cases x̄ = 43 Controls x̄ = 40	175	Ontario Cancer Registry	253	RDD	2016-2017	Non-specific	AS/TT[a]	FFQ of 2 yrs prior[d]	EO-CRC	Inc	2018-2019
Davis *et al*. (2023) (91)	US	B	Cases: x̄ = 61.1 Controls: x̄ = 60.85	213	Roswell Park Comprehensive Cancer Center PEDS (cases), Roswell Park cancer registry (vital status)	852	Roswell Park Comprehensive Cancer Center PEDS with non-cancer diagnosis	–	Non-specific	Diet cola	FFQ on a "few yrs prior to diagnosis"	Pancreatic	Inc & Mort	1982-1998
Ewertz and Gill (1990) (45)	Denmark	F	<70	1,363	Danish Cancer Registry and clinical trial of Danish Breast Cancer Cooperative Group	1,223	Central Population Register	1982-1983	Non-specific	AS (use in coffee and tea)	FFQ administered 1 yr after diagnosis on diet 1 yr prior to diagnosis	Breast	Inc	1983-1984
Franceschi *et al*. (1997) (84)	Italy	B	Cases: median = 62 Controls: median = 58	1,225	Major teaching and general hospitals	4,154	Major teaching and general hospitals	1990-1994	Non-specific	AS/TT[a]	Interview and FFQ on diet 2 yrs prior to diagnosis/ admission	Colon	Inc	1992-1996
			Cases: median = 62 (range: 23-74) Controls: median = 58	728								Rectal		

Study	Location	Study Population						Exposure Characterization				Cancer Outcome		
		Sex	Age (yrs)	Cases		Controls								
				N	Source	N	Source	Exposure Period	NSS	Source	Exposure Ascertainment	Cancer Type	Outcome Type	Study Period
Gallus *et al*. (2007) (46)	Italy	B	Cases: median = 62 Controls: median = 58 (colon and rectal combined)	1,225	Hospitals	4,154 (colon and rectal combined)	Hospitals	1989-2004	Non-specific or saccharin	TT	FFQ of 2 yrs prior to diagnosis/ hospitalization	Colon	Inc	1991-2004
				728								Rectal		
			Median = 60	304		743						Esophageal		
			Median = 61	460		1,088						Laryngeal		
			Cases: median = 58 Controls: median = 57	598		1,491						Oral cavity & pharyngeal		
			Median = 62	767		1,534						RCC		
		F	Cases: median = 55 Controls: median = 56	2,569		2,588						Breast		
			Cases: median = 56 Controls: median = 57	1,031		2,411						Ovarian		
		M	Cases: median = 66 Controls: median = 63	1,294		1,451						Prostate		
Gold *et al*. (1985) (92)	US	B	x̄ = 66.1	274	Hospitals	201	Hospitals: non-cancer hospital records<br><br>Population: RDD	–	Non-specific	Diet soda	In-person interview and FFQ on consumption prior to illness (cases) or 1 yr prior to interview (controls)	Pancreatic	Inc	1978-1980

Study	Location	Study Population						Exposure Characterization				Cancer Outcome		
		Sex	Age (yrs)	Cases		Controls								
				N	Source	N	Source	Exposure Period	NSS	Source	Exposure Ascertainment	Cancer Type	Outcome Type	Study Period
Goodman *et al*. (1986) (75)	US	B	Cases: x̄ = 58 (M) 58.2 (F) Controls: x̄ = 57.7 (M) 58.1 (F)	267	Hospitals	267	Hospitals	Lifetime	Saccharin or non-specific	TT, diet beverages, and AS	Self-reported *via* in-person interview	RCC	Inc	1977-1983
Gurney *et al*. (1997) (99)	US	B	>19	56	Medical records in 19 West Coast US counties	94	RDD	–	Aspartame	Whole diet or diet drinks	Participants' mothers *via* in-person interview	Childhood Brain	Inc	1984-1991
				49		90				Mother's consumption of aspartame for in-utero or breastmilk exposure				
Hardell *et al*. (2001) (101)	Sweden	B	x̄ = 50[e]	136	Hospitals	425	Population register	–	Aspartame[f]	Low-calorie beverages	Self-reported *via* questionnaire, and nurse-trained telephone interview for unclear answers	Brain	Inc	1994-1996
Hoover and Strasser (1980) (42)	US	B	21-84	3,010	SEER and NJ Cancer Registry	5,783	RDD (ages 21-64) and HCFA (ages 65-84)	Lifetime	Non-specific	TT, diet drinks, and diet foods	Self-reported *via* in-person interview and questionnaire	Bladder	Inc	1977-1978
Howe *et al*. (1977) (52)	Canada	B	Cases: x̄ = 67.7 (M) 69.1 (F) Controls: x̄ = 67.2 (M) 68.4 (F)	M: 480, F: 152	Provincial cancer registries and pathologists/ urologists	M: 480, F: 152	Electoral list census division and neighborhood	Lifetime	Saccharin or non-specific	TT, diet drinks, and diet foods	Self-reported *via* in-person interview and questionnaire	Bladder	Inc	1974-1976

Study	Location	Study Population						Exposure Characterization				Cancer Outcome		
		Sex	Age (yrs)	Cases		Controls								
				N	Source	N	Source	Exposure Period	NSS	Source	Exposure Ascertainment	Cancer Type	Outcome Type	Study Period
Howe *et al*. (1980) (53)	Canada	B	Cases: x̄ = 67.7 (M) 69.1 (F) Controls: x̄ = 67.2 (M) 68.4 (F)	M: 480, F: 152	Provincial cancer registries and pathologists/ urologists	M: 480, F: 152	Electoral list census division and neighborhood	Lifetime	Saccharin or non-specific	TT and dietetic drinks	Self-reported *via* in-person interview	Bladder	Inc	1974-1976
Ibiebele *et al*. (2008) (78)	Australia	B	x̄ = 60	EAC: 294<br>EGJAC: 325<br>Esophageal SCC: 238	Treatment centers and cancer registries	1,484	Australian Electoral Roll	2000-2004	Non-specific	Low calorie soft drinks	Self-administered FFQ on prior yr	EAC, EGAC, and esophageal SCC	Inc	2001-2005
Iscovich *et al*. (1987) (54)	Argentina	B	Median = 65-74	117	Hospitals	117<br>117	Hospitals<br>Neighborhood/ street block	–	Saccharin	–	In-person interview and questionnaire	Bladder	Inc	1983-1985
Kantor *et al*. (1985) (55)	US	B	Median >65	2,900	SEER and NJ Cancer Registry	5,684	RDD and HCFA	Lifetime	Non-specific	AS/TT[a]	Self-reported *via* in-person interview and questionnaire	Bladder	Inc	1977-1978
Kantor *et al*. (1988) (56)	US	B	21-84	SCC: 33<br>Adenocarcinoma: 24<br>TCC: 2,277	SEER and NJ Cancer Registry	5,782	RDD (ages 21-64) and HCFA (ages 65-84)	Lifetime	Non-specific	AS/TT[a]	In-person interview with structured questionnaire	SCC, adeno-carcinoma, and TCC of bladder	Inc	1977-1978
Kessler and Clark (1978) (57)	US	B	Cases: x̄ = 67.5; Controls: x̄ = 67.7	519	Hospitals	519	Hospitals	>1 yr before diagnosis	Saccharin and/or cyclamate	TT, diet beverages, and diet foods	Self-reported *via* interview	Bladder	Inc	1972-1975
Kobeissi *et al.* (2013) (58)	Lebanon	M	Cases: x̄ = 67.1<br>Controls: x̄ = 65.6	54	Hospitals	90	Hospitals	Lifetime	Non-specific	AS	In-person interview and questionnaire	Bladder	Inc	2002-2008

Study	Location	Study Population						Exposure Characterization				Cancer Outcome		
		Sex	Age (yrs)	Cases		Controls								
				N	Source	N	Source	Exposure Period	NSS	Source	Exposure Ascertainment	Cancer Type	Outcome Type	Study Period
Li *et al*. (2006) (96)	US	B	Males: x̄ = 54 Females: x̄ = 55	111	RPCI	439	RPCI	–	Non-specific	Diet cola	FFQ	AML	Inc	1982-1998
Maclure and Willett (1990) (76)	US	B	≥30	203	Boston metropolitan area hospitals	227 matched; 378 unmatched	MA town residence lists	Early 1970s	Non-specific	Diet soft drink	In-person interview FFQ	Renal adeno-carcinoma	Inc	1976-1983
Mahfouz *et al*. (2014) (86)	Egypt	B	Cases: x̄ = 46.1 Controls: x̄ = 46.7	150	Minia Oncology Centre	300	Case communities	2008-2011	Non-specific	AS/TT[a]	FFQ of previous 2 yrs prior to diagnosis	Colorectal	Inc	2010-2011
Marrett *et al*. (1985) (59)	US	B	21-84	412	CT Tumor Registry	881	RDD (ages 21-64); HCFA files (ages 65+)	–	Non-specific	AS	In-person interview with structured questionnaire	Bladder	Inc	1978-1979
Mayne *et al*. (2006) (79)	US	B	30-79	282	Rapid-reporting systems, pathology reports, and medical records	687	RDD (ages 30-64); HCFA rosters (ages 65-79)	1988-1992	Non-specific	Diet carbonated soft drinks	In-person structured questionnaire	EAC, GCA, esophageal SCC, and NCGA	Inc	1993-1995
Mettlin (1989) (102)	US	B	Cases: x̄ = 60.6 Controls: x̄ = 59.7	569	RPMI PEDS	569	RPMI PEDS	Prior to current illness	Non-specific	Diet cola	FFQ	Lung	Inc	1982-1987
Moller-Jensen *et al*. (1983) (41)	Denmark	B	< 75	388	Cancer registry	787	National register	Prior to illness	Saccharin and/or cyclamate	TT, diet drinks, foods	Self-reported *via* interview	Urinary Bladder	Inc	1979-1981

Study	Location	Study Population						Exposure Characterization				Cancer Outcome		
		Sex	Age (yrs)	Cases		Controls								
				N	Source	N	Source	Exposure Period	NSS	Source	Exposure Ascertainment	Cancer Type	Outcome Type	Study Period
Momas *et al*. (1994) (60)	France	M	Cases: x̄ = 67.8<br><br>Controls: x̄ = 64.6 (not in telephone directory), 65.5 (in telephone directory), all >50 yrs	219	Urologists and Hérault Cancer Registry	794	Electoral rolls from Hérault region	Lifetime cumulative	Saccharin	Tablets	Self-reported *via* interview and questionnaire (all cases, some controls) or mailed questionnaire (some controls)	Bladder	Inc	1987-1989
Mommsen *et al*. (1983) (61)	Denmark	F	x̄ = 66.4	47	Hospital	94	National register regional division	–	Saccharin	AS as sugar substitute	Self-reported *via* questionnaire and in-person (cases) or telephone (controls) interview	Bladder	Inc	1977-1980
Morgan and Jain (1974) (62)	–	B	Cases: x̄ = 71.4 (M) 71.5 (F)<br><br>Controls: x̄ = 68.3 (M) 68.9 (F)	58[g]	Hospitals	174[g]	Hospitals	>1 yr	Non-specific	Diet desserts, sugar-free soft drinks, or sugar substitutes	Self-reported *via* mailed questionnaire	TCC of bladder	Inc	–
Morrison (1979) (33)	Seven countries	B	≥40	1,862	Hospitals	10,874	Hospitals	Lifetime	Saccharin and Cyclamates	AS	Self-reported *via* interview and questionnaire	All cancers	Inc	1969-unknown

Study	Location	Study Population						Exposure Characterization				Cancer Outcome		
		Sex	Age (yrs)	Cases		Controls								
				N	Source	N	Source	Exposure Period	NSS	Source	Exposure Ascertainment	Cancer Type	Outcome Type	Study Period
Morrison and Buring (1980) (34)	US	B	21-89	592	Hospitals and medical records	536	Residents Lists	Lifetime	Non-Specific	Dietetic beverages, sugar substitutes, and AS foods	Self- or proxy-reported *via* in-person or telephone interview and questionnaire	Lower urinary tract[h]	Inc	1976-1977
Morrison *et al*. (1982) (35)	UK and Japan	B	21-89	UK: 577; Japan: 348	Hospitals (UK and Japan); Cancer registry (UK)	UK: 817; Japan: 735	Electoral registers	Lifetime and yr before interview/ hospitalization	Non-specific[i]	UK: Diet or low-calorie beverages, sweeteners other than sugar, saccharin, or low-calorie or low-sugar brands; Japan: sugar substitutes added to beverages and foods	Self- or proxy-reported *via* in-person interview and questionnaire	Lower urinary tract[h]	Inc	1976-1978
Najem *et al*. (1982) (63)	US	B	Cases: x̄ = 66.8; Controls: x̄ = 70.9	75	Urologists' offices and hospitals	142	Urologists' offices and hospitals	Lifetime	Non-specific or saccharin	Beverages and TT	Self-reported *via* interview and questionnaire	Bladder	Inc	1978
Nomura *et al*. (1991) (36)	US	B	30-93	261	Hospital records and pathology departments	521	Subjects interviewed by Health Surveillance Program	1976-1985	Saccharin or non-specific	Beverages and TT	In-person interview and FFQ on year prior	Lower urinary tract[j]	Inc	1977-1986

Study	Location	Study Population						Exposure Characterization				Cancer Outcome		
		Sex	Age (yrs)	Cases		Controls								
				N	Source	N	Source	Exposure Period	NSS	Source	Exposure Ascertainment	Cancer Type	Outcome Type	Study Period
Norell *et al*. (1986) (94)	Sweden	B	40-79	99	Surgical departments in Stockholm and Uppsala	Population: 138<br><br>Hospital: 163	Population: from parish registries<br><br>Hospital: patients with inguinal hernia	–	Non-specific	AS/TT[a]	FFQ on diet prior to diagnosis	Exocrine pancreas	Inc	1982-1984
Ohno *et al*. (1986) (64)	Japan	B	20-89	289	Nagoya Bladder Tumor Registry	575	Electoral registers	–	Non-specific	AS/TT[a]	In-person interview of subject/proxy with clarification by telephone	Bladder	Inc	1976-1978
Palomar-Cros *et al.* (2023) (47)	Spain	B	x̄ = 63.07	1,881	Hospitals	3,498	Health care center administrative records	2007-2013	Aspartame, saccharin, and AS (excluding aspartame)	TT sweeteners and beverages	Self-administered FFQ on previous yr	Colorectal	Inc	2008-2013
				351		3,030						Stomach		
				109		1,631						CLL		
		M		972		1,308						Prostate		
		F		1,510		1,674						All breast		
				541		483						Premenopausal breast		
				969		1,191						Postmenopausal breast		
Piper *et al*. (1986) (65)	US	F	20-49	173	Cancer Registry of the NY State Health Department	173	RDD	–	Non-specific	ASBs and/or TT	Self-reported *via* telephone interview and structured questionnaire	Bladder	Inc	1975-1980
Risch *et al*. (1988) (66)	Canada	B	35-79	826	Hospital records and registries	792	Province population listings	–	Saccharin and/or cyclamate, and non-specific	TT, diet soda, and low calorie foods	In-person interview and questionnaire	Bladder	Inc	1979-1982

Study	Location	Study Population						Exposure Characterization				Cancer Outcome		
		Sex	Age (yrs)	Cases		Controls								
				N	Source	N	Source	Exposure Period	NSS	Source	Exposure Ascertainment	Cancer Type	Outcome Type	Study Period
Schulte *et al*. (1986) (67)	US	–	–	45[k]	Dye-intermediary production plant cohort	335	Dye-intermediary production plant cohort	1940-1983	Non-specific	AS	Questionnaire	Bladder	Inc	1940-1983
Silverman *et al*. (1983) (37)	US	B	21-84	391	Hospitals	305	Hospitals	Any time prior to start of the study	Non-specific	TT, diet drinks, and diet foods	Self/proxy-reported *via* in-person/ telephone interview and questionnaire	Lower urinary tract[h]	Inc	1977-1978
						440	RDD and HCFA							
Simon *et al*. (1975) (38)	US	F	Cases: x̄ = 62.6 Controls: x̄ = 62.7	135	Hospitals	390	Hospitals	Adulthood	Cyclamate and saccharin	TT	Self-reported *via* mailed questionnaire	Lower urinary tract[h]	Inc	1965-1971
Singh *et al.* (2020)[l]	US	B	x̄ = 53.6	50	Hospital records	50[m]	Hospital records	Ever	Non-specific	TT sweeteners and beverages	Telephonic survey questionnaire	WDTC	Inc	2004-2014
Sturgeon *et al*. (1994) (68)	US	B	21-84	1,860	SEER cancer registries	3,934	RDD (ages 21-64) and HCFA records (ages 65-84)	Lifetime	Non-specific	AS/TT[a]	Structured in-person interviews	Bladder	Inc	1977-1978
Sullivan (1982) (69)	US	B	21-85	82	–	169	RDD (ages <65) and HCFA (ages >65)	Lifetime	Non-specific	ASB	Self-reported *via* in-person interview and questionnaire	Bladder	Inc	1977-1978
Theodoratou *et al.* (2014) (87)	Scotland	B	16-79	2,062	Surgical units	2,776	Population-based registry	1998-2006	Non-specific	Low calorie drinks	Self-completed FFQ on prior yr	Colorectal	Inc	1999-2006
Wang *et al*. (2013) (70)	US	B	Cases: x̄ = 64.64 Controls: x̄ = 64.20	1,007	Hospitals	1,299	Hospitals	1998[n]	Non-specific	Diet soft drinks	Interview and FFQ on yr prior	Bladder	Inc	1999[n]

Study	Location	Study Population						Exposure Characterization				Cancer Outcome		
		Sex	Age (yrs)	Cases		Controls								
				N	Source	N	Source	Exposure Period	NSS	Source	Exposure Ascertainment	Cancer Type	Outcome Type	Study Period
Wu *et al*. (1997) (88)	US	B	30-65	36	LA County Cancer Surveillance Program	998	Case neighborhood and multi-site cancer study controls	Prior to cancer diagnosis/ interview	Non-specific	AS added to tea and/or coffee	Self- or surrogate-reported *via* interview°	Small intestinal adeno-carcinoma	Inc	1975-1980
Wynder and Goldsmith (1977) (40)	US	B	Cases: x̄ = 61.8 (M), 62.8 (F)	163	Hospitals	153	Hospitals	Lifetime	Saccharin	AS	Self-reported *via* in-person interview and questionnaire	Bladder	Inc	1973-1974
Wynder and Stellman (1980) (71)	US	B	–	367	Hospitals	367	Hospitals	Lifetime	Saccharin	AS and diet beverages	Self-reported *via* in-person interview	Bladder	Inc	1977-1979
Wynder *et al*. (1986) (93)	US	B	Median = 60-69	238	Hospitals	696	Hospitals	Lifetime	Saccharin	Saccharin use	In-person interview with structured questionnaire	Pancreatic	Inc	1981-1984
Yu *et al*. (1997) (39)	China	B	Cases: x̄ = 55.4 (M), 53.8 (F); Controls: x̄ = 55.3 (M), 53.3 (F)	127	Hospital	254	Hospital	Lifetime	Saccharin	–	Self-reported *via* in-person interview	Bladder	Inc	1987-1990

Notes:
AML = Acute Myeloid Leukemia; AS = Artificial Sweetener; ASB = Artificially Sweetened Beverage; B = Both Males and Females; CA = California; CLL = Chronic Lymphocytic Leukemia; COG = Children's Oncology Group; CT = Connecticut; EAC = Esophageal Adenocarcinoma; EGJAC = Esophagogastric Junction Adenocarcinoma; EO-CRC = Early-Onset Colorectal Cancer; F = Females; FFQ = Food Frequency Questionnaire; GCA = Gastric Cardia Adenocarcinoma; HCFA = Health Care Finance Administration; Inc = Incidence; LA = Los Angeles; M = Males; MA = Massachusetts; MPBT = Malignant Primitive Brain Tumor; NCGA = Noncardia Gastric Adenocarcinoma; NJ = New Jersey; NSS = Non-Sugar Sweetener; NY = New York; PEDS = Patient Epidemiologic Data System; PNET = Primitive Neuroectodermal Tumor; RCC = Renal Cell Carcinoma; RDD = Random Digit Dialing; RPCI = Roswell Park Cancer Institute; RPMI = Roswell Park Memorial Institute; SCC = Squamous Cell Carcinoma; SEER = Surveillance, Epidemiology, and End Results; TCC = Transitional Cell Carcinoma; TT = Tabletop; UCSF = University of California San Francisco; US = United States; WDTC = Well-Differentiated Thyroid Cancer; yr = Year.
x̄ = Mean; – = Not Reported.
(a) Study only mentions AS without details on source. We assumed TT (50, 55, 56, 64, 68, 84, 85, 86, 94).
(b) Urinary tract tumors included transitional-cell types from renal pelvises, ureters, and/or bladder (32).
(c) Bunin *et al*. (98) stated that during the time period of gestation of the children in their study, aspartame was the most used AS in soft drinks.
(d) Median time between diagnosis and questionnaire completion was 10 months (5th-95th percentile: 5-16 months) (85).
(e) This includes benign tumor cases (101).
(f) Hardell *et al*. (101) assumed that all low-calorie drinks consumed by subjects contained aspartame.
(g) Numbers of cases and controls were difficult to interpret due to apparent formatting errors in Table III of the study (62).

(h) The authors defined the lower urinary tract as including the bladder, ureter, renal pelvis, or urethra (34, 35, 37, 38).
(i) Morrison *et al*. (35) stated that their findings are based "primarily on the use of saccharin."
(j) Lower urinary tract cancers consisted of 90% bladder, 7% renal pelvis, and 3% ureter cancers (36).
(k) This included 9 confirmed bladder cancer cases and 36 people with atypical bladder cytology, histology, or pathology considered to be indicative of a precancerous state (67).
(l) This study is not cited in the main manuscript but rather only in the supplements. The full reference for this study is listed at the end of this supplement.
(m) Controls in Singh *et al.* (2020) were patients with benign thyroid nodules.
(n) Wang *et al*. (70) did not report the end of the exposure period or the study period. At the time of publication, they stated, "patient recruitment in 1999 and is currently ongoing."
(o) Cases included 31 self-respondent interviews and 5 surrogate interviews. All controls were interviewed in-person (88).

Supplemental Table E.3 Cohort Study Quality Evaluation

Study (Cohort)	Cancer Type(s)	Study Quality Criteria															
		Intake Assessment						Outcome Assessment				Covariates/ Confounding			Sample Selection		
		Specific AS	Valid	Multiple Timepoints	Timing	Source	Dose	Diagnosis	Incidence	Latency	Cancer Type	Model	Confounders	Time-Varying	Comparison	Follow-up	Justification
Bao *et al*. (30) (NIH-AARP Diet and Health Study)	Pancreas	N	Y	N	Y	Y	Y	Y	Y	N	Y	Y	Y	N	Y	Y	Non-specific NSS type (*i.e.*, diet soft drinks); validated FFQs against two 24-hr recalls; exposure collected at single timepoint; ~10% of cases not validated; short duration of follow-up (mean = 7.2 years); insufficient consideration of latency (*i.e.*, 2 yr lag); did not consider time-varying nature of relevant covariates
Bassett *et al*. (111) (MCCS)	Obesity- and non-obesity-related cancers	N	N	N	Y	Y	Y	Y	Y	N	N	Y	Y	N	Y	N	Not specific NSS type (*i.e.*, diet [AS] soft drinks); questionnaire not validated for diet (AS) soft drink consumption; exposure collected at one time point; insufficient consideration of disease latency (*i.e.*, 2 yrs in sensitivity analyses); aggregated cancer outcome (*i.e.*, non-obesity-related & obesity-related cancers); did not consider time-varying nature of relevant covariates
Chazelas *et al*. (83) (NutriNet-Santé)	Colorectal, prostate, and breast	N	Y	Y	Y	Y	Y	Y	Y	N	Y	Y	Y	N	Y	N	Not specific NSS type (*i.e.*, ASB) assessed *via* 24-hr dietary recall; did not consider disease latency; did not consider time-varying nature of some relevant covariates (*e.g.*, diabetes, physical activity)
	All cancers										N						Aggregated cancer outcome (*i.e.*, all cancers)
Debras *et al*. (20) (NutriNet-Santé)	Prostate and breast	Y	Y	Y	Y	Y	Y	Y	Y	N	Y	Y	Y	N	Y	N	Assessed exposure *via* 24-hr dietary recall; did not consider disease latency; did not consider time-varying nature of some relevant covariates (*e.g.*, diabetes, physical activity)
	All and obesity-related cancers										N						Aggregated cancer outcome (*i.e.*, all cancers, obesity-related cancers)
Fulgoni and Drewnowski (2022)[a] (NHANES)	All cancers	Y	Y	N	Y	Y	Y	Y	N	N	N	Y	Y	N	Y	N	Exposure collected at single timepoint *via* 24-hr dietary recall; assessed mortality only; did not consider disease latency; aggregated cancer outcome (*i.e.*, all cancers); did not consider time-varying nature of relevant covariates
Gao *et al*. (2024)[a] (NHANES)	All cancers	Y	N	N	Y	Y	Y	Y	N	N	N	Y	Y	N	Y	N	Exposure collected at single timepoint *via* 24-hr dietary recall; did not report validation of the 24-hr dietary recalls; assessed mortality only; insufficient consideration of latency (*i.e.*, 3 yr lag); aggregated cancer outcome (*i.e.*, all cancers); did not consider time-varying nature of relevant covariates
Heath *et al*. (74) (EPIC)	RCC	N	Y	N	Y	Y	Y	Y	Y	N	Y	Y	Y	N	Y	N	Not specific NSS type (*i.e.*, AS soft drink); exposure collected at one time point; Heath et al. (74) did not report the validity of the questionnaire, but other studies in the same cohort have reported that the questionnaire was validated (*e.g.*, 27); insufficient consideration of disease latency (*i.e.*, 2 yrs); did not consider time-varying nature of relevant covariates
Hodge *et al*. (44) (MCCS)	Kidney, colorectal, gastric cardia, ovarian, endometrial, post-menopausal breast, and prostate	N	N	N	Y	Y	Y	Y	Y	N	Y	Y	Y	N	Y	N	Not specific NSS type (*i.e.*, diet soft drinks); questionnaire not validated; exposure collected at one time point; insufficient consideration of disease latency (*i.e.*, 2 yrs); did not consider time-varying nature of relevant covariates
	Obesity-related cancer										N						Aggregated cancer outcome (*i.e.*, obesity-related cancer)
Hur *et al*. (82) (NHSII)	EO-CRC	N	Y	Y	Y	Y	Y	Y	Y	N	Y	Y	Y	Y	Y	N	Not specific NSS type (*i.e.*, low-calorie carbonated beverages); did not consider disease latency
Inoue-Choi *et al*. (97) (IWHS)	Type 1 and 2 endometrial	N	Y	N	Y	Y	Y	Y	Y	N	Y	Y	Y	N	Y	N	Not specific NSS type (*i.e.*, sugar-free soft drinks); exposure collected at one time point; did not consider disease latency; did not consider time-varying nature of relevant covariates
Jones *et al*. (25) (NIH-AARP and PLCO)	Liver	N	Y	N	Y	Y	Y	Y	Y	Y	Y	Y	Y	N	Y	N	Not specific NSS type (*i.e.*, AS or diet); exposure collected at one time point; did not report the validity of the questionnaire, however, the study homepage (NCI, 2023)[a] states it was validated; did not consider time-varying nature of relevant covariates

Study (Cohort)	Cancer Type(s)	Study Quality Criteria															
		Intake Assessment						Outcome Assessment				Covariates/ Confounding			Sample Selection		
		Specific AS	Valid	Multiple Timepoints	Timing	Source	Dose	Diagnosis	Incidence	Latency	Cancer Type	Model	Confounders	Time-Varying	Comparison	Follow-up	Justification
Lee *et al*. (24) (NHS and HPFS)	RCC	N	Y	Y	Y	Y	Y	N	Y	N	Y	Y	Y	Y	Y	Y	Not specific NSS type (*i.e.*, AS sodas); unclear if all self/proxy-reported cases and deaths were validated; did not consider disease latency
Lim *et al*. (95) (NIH-AARP Diet and Health Study)	Malignant gliomas, HL, MM, NHL, immunoblastic lymphoma and lymphoblastic lymphoma/ leukemia, and non-lymphoid leukemia	Y	Y	N	Y	Y	Y	Y	Y	N	Y	Y	Y	N	Y	N	Exposure collected at one time point; did not consider disease latency; did not consider time-varying nature of relevant covariates
	All hematopoietic cancers, all lymphoid malignancies										N						Aggregated cancer outcome (*i.e.*, all hematopoietic cancers, all lymphoid malignancies)
Liu *et al*. (18) (UK Biobank)	All cancers	N	Y	Y	Y	N	Y	Y	N	N	N	Y	Y	N	Y	Y	Not specific NSS type (*i.e.*, AS coffee); only considered minor source of NSS exposure (*i.e.*, coffee) assessed *via* 24-hr dietary recall; assessed mortality only; insufficient consideration of disease latency (*i.e.*, 2 yrs); aggregated cancer outcome (*i.e.*, all cancers); did not consider time-varying nature of relevant covariates
McCullough *et al*. (21) (CPS-II Nutrition Cohort)	MM, and NHL	Y	Y	Y	Y	Y	Y	Y	Y	Y	Y	Y	Y	N	Y	Y	Did not consider time-varying nature of relevant covariates
McCullough *et al*. (26) (CPS-II)	Esophageal, stomach, liver, pancreatic, gall bladder, lung, small intestinal, colorectal, breast, uterus, ovarian, prostate, kidney, bladder, melanoma, brain, NHL, MM, and leukemia	N	N	N	Y	Y	Y	Y	N	Y	Y	Y	Y	N	Y	N	Not specific NSS type (*i.e.*, diet sodas & iced teas); did not report use of validated questionnaire (*i.e.*, the methods report that they asked a couple of questions at baseline on intake); exposure collected at one time point; assessed mortality only; did not consider time-varying nature of relevant covariates
	All cancers, obesity-related, and laryngeal/oral cavity/pharyngeal										N						Aggregated cancer outcome (*i.e.*, all cancers, obesity-related, and laryngeal/oral cavity/pharyngeal)
Mullee *et al*. (27) (EPIC)	Colorectal, breast, and prostate	N	Y	N	Y	Y	Y	Y	N	N	Y	Y	Y	N	Y	Y	Not specific NSS type (*i.e.*, AS soft drinks); exposure collected at one time point; assessed mortality only; did not consider disease latency; did not consider time-varying nature of relevant covariates
	All cancers									Y	N						Aggregated cancer outcome (*i.e.*, all cancers)

Study (Cohort)	Cancer Type(s)	Study Quality Criteria															
		Intake Assessment						Outcome Assessment				Covariates/ Confounding			Sample Selection		Justification
		Specific AS	Valid	Multiple Timepoints	Timing	Source	Dose	Diagnosis	Incidence	Latency	Cancer Type	Model	Confounders	Time-Varying	Comparison	Follow-up	
Navarrete-Muñoz *et al*. (28) (EPIC)	Pancreatic (adenocarcinoma of the exocrine pancreas)	N	Y	N	Y	Y	Y	Y	Y	Y	Y	Y	Y	N	Y	Y	Not specific NSS type (*i.e.*, AS soft drinks); exposure collected at one time point; did not consider time-varying nature of relevant covariates
Ringel *et al*. (31) (WHI-OS)	Kidney	N	N	N	Y	Y	Y	Y	Y	N	Y	Y	Y	N	Y	Y	Not specific NSS type (*i.e.*, ASBs); exposure collected at one time point; did not consider disease latency; did not consider time-varying nature of relevant covariates
	Bladder												N				Failed to consider key potential confounders
	Urinary tract										N						Aggregated cancer outcome (*i.e.*, urinary tract)
Romanos-Nanclares *et al*. (29) (NHS and NHSII)	Breast	N	Y	Y	Y	Y	Y	Y	Y	Y	Y	Y	Y	Y	Y	N	Not specific NSS type (*i.e.*, ASB)
Romanos-Nanclares *et al*. (22) (NHS and NHSII)	Breast	Y	Y	Y	Y	Y	Y	Y	Y	Y	Y	Y	Y	Y	Y	N	
Schernhammer *et al*. (89) (NHS and HPFS)	Pancreatic	N	Y	Y	Y	Y	Y	Y	Y	N	Y	Y	Y	Y	Y	N	Not specific NSS type (*i.e.*, diet soft drinks); did not consider disease latency
Schernhammer *et al*. (23 (NHS and HPFS)	Leukemia, MM, and NHL	Y	Y	Y	Y	Y	Y	Y	Y	N	Y	Y	Y	Y	Y	N	Did not consider disease latency
Stepien *et al*. (49) (EPIC)	HCC	N	Y	N	Y	Y	Y	Y	Y	N	Y	Y	Y	N	Y	Y	Not specific NSS type (*i.e.*, AS soft drinks); exposure collected at one time point; insufficient consideration of disease latency (*i.e.*, 2 yrs in sensitivity analysis); did not consider time-varying nature of relevant covariates
Wang *et al*. (81) (HPFS, NHS, and NHSII)	Colorectal	N	Y	Y	Y	Y	Y	Y	Y	N	Y	Y	Y	Y	Y	Y	Not specific NSS type (*i.e.*, ASBs); did not consider disease latency
You *et al*. (19) (PLCO)	Lung	N	Y	N	Y	Y	N	Y	Y	N	Y	Y	Y	N	Y	Y	Not specific NSS type (*i.e.*, diet/sugar-free soft drinks); You et al. (19) did not report the validity of the questionnaire, however, the study homepage (NCI, 2023)[a] states it was validated; exposure collected at one time point; did not consider frequency, duration, or intake level of NSS consumption; insufficient consideration of disease latency (*i.e.*, 1 yr); did not consider time-varying nature of relevant covariates
	All cancers										N						Aggregated cancer outcome (*i.e.*, all cancers)
Zamora-Ros *et al*. (2023)[b] (EPIC)	All thyroid, papillary thyroid, and follicular thyroid	N	Y	N	Y	Y	Y	Y	Y	N	Y	Y	Y	N	Y	Y	Not specific NSS type (*i.e.*, ASBs); exposure collected at one time point; did not consider disease latency; did not consider time-varying nature of relevant covariates
Zhang *et al*. (2021)[b] (NHANES)	All cancers	N	Y	N	Y	Y	Y	Y	N	N	N	Y	Y	N	Y	N	Not specific NSS type (*i.e.*, ASBs); exposure collected *via* 24-hr dietary recall at one time point; assessed mortality only; insufficient consideration of disease latency (*i.e.*, 1 yr lag in sensitivity analysis); aggregated cancer outcome (*i.e.*, all cancers); did not consider time-varying nature of relevant covariates

Study (Cohort)	Cancer Type(s)	Study Quality Criteria															
		Intake Assessment						Outcome Assessment				Covariates/ Confounding			Sample Selection		Justification
		Specific AS	Valid	Multiple Timepoints	Timing	Source	Dose	Diagnosis	Incidence	Latency	Cancer Type	Model	Confounders	Time-Varying	Comparison	Follow-up	
Zhang *et al*. (2024)[b] (UK Biobank)	All cancers	N	Y	Y	Y	N	Y	Y	N	N	N	Y	Y	N	Y	Y	Not specific NSS type (*i.e*., AS cereals); only considered minor source of NSS exposure (*i.e*., cereals) assessed *via* 24-hr dietary recall; assessed mortality only; insufficient consideration of disease latency (*i.e*., 2 yrs); aggregated cancer outcome (*i.e*., all cancers); did not consider time-varying nature of relevant covariates
Zhao *et al*. (48) (WHI-OS)	Liver	N	Y	N	Y	Y	Y	Y	Y	N	Y	Y	Y	N	Y	N	Not specific NSS type (*e.g*., ASBs); exposure collected at one time point; did not consider disease latency; did not consider time-varying nature of relevant covariates

Notes:
AS = Artificial Sweetener; ASB = Artificially Sweetened Beverage; CPS-II = Cancer Prevention Study-II; Dx = Diagnosis; EO-CRC = Early-Onset Colorectal Cancer; EPIC = European Prospective Investigation into Cancer and Nutrition; HCC = Hepatocellular Carcinoma; HL = Hodgkin's Lymphoma; HPFS = Health Professionals Follow-up Study; IWHS = Iowa Women's Health Study; MCCS = Melbourne Collaborative Cohort Study; MM = Multiple Myeloma; N = No or Not Reported; N/A = Not Applicable; NHANES = National Health and Nutrition Examination Survey; NHL = Non-Hodgkin's Lymphoma; NHS = Nurses' Health Study; NIH-AARP = National Institutes of Health American Association of Retired Persons; NSS = Non-Sugar Sweetener; PLCO = Prostate, Lung, Colorectal, and Ovarian Cancer Screening Trial; RCC = Renal Cell Carcinoma; UK = United Kingdom; WHI-OS = Women's Health Initiative Observational Study; Y = Yes; yr = Year.
Quality Assessment Categories: Specific AS = assessed specific as (*e.g*., aspartame) or Specific As Can Be Inferred; Valid = Used Validated Questionnaire; Multiple Timepoints = Collected at Multiple Timepoints; Timing = Collected Prior to Cancer Dx; Source = Assessed Major Contributors of AS (*i.e*., Whole Diet, Beverages); Dose = Considered Frequency, Duration or Level of NSS Consumption; Diagnosis = Physician-Diagnosed, or Self-or Proxy-Reported and Validated Clinically, or Recorded in Medical Records, Death Certificates, or Registries; Incidence = Assessed Disease Incidence; Latency = Sufficient Time Between Exposure And outcome to Account for Disease Latency (≥4 Yrs Soft Tissue Tumors, ≥0.5 yrs Lymphohematopoietic Cancers); Cancer Type = Assessed Specific Cancer Type (*i.e*., Not Aggregated Cancer Outcome); Model = Proper Model and Forms of Variables; Confounders = Considered Key Potential Confounders; Comparison = Appropriate Comparison Group; and Follow-Up = <25% of Loss to Follow-Up.
(a) This source is not cited in the main manuscript, but rather only in the supplements. The full reference for this source is listed at the end of this supplement.
(b) These studies are not cited in the main manuscript, but rather only in the supplements. The full reference for these studies are listed at the end of the supplements.

Supplemental Table E.4 Case-Control Study Quality Evaluation

Study	Cancer Type(s)	Study Quality Criteria: Intake Assessment: Specific NSS	Intake Assessment: Valid	Intake Assessment: Timing	Intake Assessment: Source	Intake Assessment: Dose	Outcome Assessment: Diagnosis	Outcome Assessment: Incidence	Outcome Assessment: Latency	Outcome Assessment: Cancer Type	Covariates/ Confounding: Model	Covariates/ Confounding: Confounders	Sample Selection: Controls	Sample Selection: Exclusion	Sample Selection: Participation	Justification
Akdaş *et al*. (50)	Bladder	N	N	N	N	N	Y	Y	N	Y	N	N	N	N	N	Not specific NSS type (*i.e.*, AS); questionnaire not validated, exposure collected at one time point; exposure not collected before cancer diagnosis; only considered minor source of NSS exposure (*i.e.*, TT);[a] did not consider frequency, duration, or intake level of NSS; did not consider disease latency, improper model, covariates, or forms of variables; failed to consider key potential confounders; inappropriate comparison group (*i.e.*, hospital-based); unknown participation rates in cases and controls
Andreatta *et al*. (32)	Urinary tract	N	Y	N	N	Y	Y	Y	N	N	Y	Y	N	Y	N	Not specific NSS type (*i.e.*, saccharin, cyclamate, aspartame, and acesulfame-k combined); exposure collected at one time point; exposure not collected before cancer diagnosis; only considered minor source of NSS (*i.e.*, TT); did not consider disease latency; inappropriate comparison group (*i.e.*, hospital-based); unknown participation rates in controls
Asal *et al*. (77)	RCC	N	N	N	N	N	Y	Y	N	Y	Y	Y	Y	Y	N	Not specific NSS type (*i.e.*, AS or sugar substitutes); questionnaire not validated; exposure collected at one time point; exposure not collected before cancer diagnosis; only considered minor source of NSS exposure (*i.e.*, TT); did not consider frequency, duration, or intake level of NSS consumption; did not consider disease latency; unknown participation rates in controls
Bosetti *et al*. (80)	Endometrial<br>Pancreatic<br>Stomach	Y	Y	N	N	N	Y	Y	N	Y	Y	Y	N	Y	Y	Exposure collected at one time point; exposure not collected before cancer diagnosis; only considered minor source of NSS exposure (*i.e.*, TT); did not consider frequency, duration, or intake level of NSS consumption; did not consider disease latency; inappropriate controls (*i.e.*, hospital-based)
Bruemmer *et al*. (51)	Bladder	N	N	N	Y	Y	Y	Y	N	Y	Y	N	Y	N	N	Not specific NSS type (*i.e.*, diet soda); questionnaire not validated; exposure collected at one time point; exposure not collected before cancer diagnosis; insufficient consideration of disease latency (*i.e.*, 2 yrs); failed to consider key potential confounders, exclusion of 37.6% of cases; differential (>15%) participation rates between cases and controls
Bunin *et al*. (98)	Medulloblastoma/PNET	N	N	N	Y	Y	Y	Y	N	Y	Y	N	Y	N	N	Not specific NSS type (*i.e.*, diet soda); used a modified version of a validated questionnaire but modifications not validated; exposure collected at one time point; exposure not collected before cancer diagnosis; did not consider disease latency; failed to consider key potential confounders; exclusion of >25% of eligible cases and controls; differential (>15%) participation rates between cases and controls
Cabaniols *et al*. (100)	MPBT	Y	N	N	Y	Y	Y	Y	N	Y	Y	N	N	N	N	Questionnaire not validated; participants were asked about "aspartame consumption" we assumed this considered whole diet"; exposure collected at one time point; exposure not collected before cancer diagnosis; did not consider disease latency; failed to consider key potential confounders; inappropriate controls (*i.e.*, hospital-based, exclusion of 28.4% of cases (unknown for controls)
Cartwright *et al*. (43)	Bladder	Y	N	N	N	Y	Y	Y	Y	Y	Y	N	N	N	N	Questionnaire not validated; exposure collected at one time point; exposure not collected before cancer diagnosis; only considered minor source of NSS exposure (*i.e.*, TT); failed to consider key potential confounders; included both prevalent and incident cases; inappropriate comparison group (*i.e.*, hospital-based); unknown number of subjects excluded
Chan *et al*. (90)	Adenocarcinoma of exocrine pancreas	N	Y	N	Y	Y	Y	Y	N	Y	Y	Y	Y	N	Y	Not specific NSS type (*i.e.*, sugar-free carbonated and low-calorie beverages); exposure collected at one time point; exposure not collected before cancer diagnosis; did not consider disease latency; exclusion of 33% of subjects
Chang *et al*. (85)	EO-CRC	N	N	N	N	Y	Y	Y	N	Y	Y	Y	Y	N	N	Not specific NSS type (*i.e.*, AS); questionnaire not validated; exposure collected at one time point; exposure not collected before cancer diagnosis; only considered minor source of NSS exposure (*i.e.*, TT);[a] insufficient consideration of disease latency (*i.e.*, exposure assessed for the time period 2 yrs before questionnaire); excluded >25% of participants with some information how those excluded differ from those included; differential (>15%) participation rates between cases and controls
Davis *et al*. (91)	Pancreatic	N	N	N	Y	Y	Y	Y	N	Y	Y	Y	N	N	N	Not specific NSS type (*i.e.*, diet cola); questionnaire not validated; exposure collected at one time point; exposure not collected before cancer diagnosis; did not consider disease latency; inappropriate comparison group (*i.e.*, hospital-based); unknown number of subjects excluded
Ewertz and Gill (45)	Breast	N	N	N	N	N	Y	Y	N	Y	Y	N	Y	Y	Y	Not specific NSS type (*i.e.*, AS); questionnaire not validated; exposure collected at one time point; exposure collected after cancer diagnosis; only considered minor source of NSS exposure (*i.e.*, TT, added to coffee and tea); did not consider frequency, duration, or intake level; did not consider latency; failed to consider key potential confounders

Study	Cancer Type(s)	Study Quality Criteria														
		Intake Assessment					Outcome Assessment				Covariates/ Confounding		Sample Selection			Justification
		Specific NSS	Valid	Timing	Source	Dose	Diagnosis	Incidence	Latency	Cancer Type	Model	Confounders	Controls	Exclusion	Participation	
Franceschi *et al.* (84)	Colon<br>Rectal	N	Y	N	N	N	Y	Y	N	Y	Y	Y	N	Y	Y	Not specific NSS type (*e.g.*, all AS); exposure collected at one time point; exposure not collected before cancer diagnosis; only considered minor source of NSS exposure (*i.e.*, TT);[a] did not consider frequency, duration, or intake level of NSS consumption; did not consider disease latency; inappropriate controls (*i.e.*, hospital-based)
Gallus *et al.* (46)	Colon<br>Rectal<br>Esophageal<br>Laryngeal<br>Oral and pharyngeal<br>RCC<br>Breast<br>Ovarian<br>Prostate	Y	Y	N	N	Y	Y	Y	N	Y	Y	Y	N	N	N	Exposure collected at one time point; exposure not collected before cancer diagnosis; only considered minor source of NSS exposure (*i.e.*, TT); did not consider disease latency; inappropriate comparison group (*i.e.*, hospital-based); unknown number of subjects excluded
Gold *et al.* (92)	Pancreatic	N	N	N	Y	N	Y	Y	N	Y	Y	Y	Y	N	N	Not specific NSS type (*i.e.*, diet soda); questionnaire not validated; exposure collected at one time point; exposure not collected before cancer diagnosis; did not consider frequency, duration, or intake level of NSS consumption; did not consider disease latency; excluded 30% of cases and 46-50% of controls; differential (>15%) participation rates between cases and controls
Goodman *et al.* (75)	RCC	Y	N	N	Y	Y	Y	Y	N	Y	Y	N	N	Y	Y	Questionnaire not validated; exposure collected at one time point; exposure not collected before cancer diagnosis; did not consider disease latency; failed to consider key potential confounders (*i.e.*, unclear if all variables were included in their conditional logistic regression model); inappropriate comparison group (*i.e.*, hospital-based with some subjects having other cancers)
Gurney *et al.* (99)	Brain	Y	N	N	Y	Y	Y	Y	N	Y	Y	N	N	N	N	Questionnaire not validated; exposure collected at one time point; exposure not collected before cancer diagnosis; did not consider disease latency; failed to consider key potential confounders; inappropriate comparison group (*i.e.*, not age-matched); unknown how many subjects excluded
Hardell *et al.* (101)	Brain	Y	N	N	Y	N	Y	Y	N	Y	Y	N	Y	Y	Y	Not specific NSS type (*i.e.*, low-calorie drinks; assumed to be aspartame); questionnaire not validated; exposure collected at one time point; exposure not collected before cancer diagnosis; did not consider frequency, duration, or intake level of NSS consumption; insufficient consideration of disease latency (*i.e.*, 1 yr); failed to consider key potential confounders; unknown exclusion of participants
Hoover and Strasser (42)	Bladder	N	N	N	Y	Y	Y	Y	Y	Y	Y	Y	Y	Y	Y	Not specific NSS type (*i.e.*, TT and diet drinks); questionnaire not validated; exposure collected at one time point; exposure not collected before cancer diagnosis
Howe *et al.* (52)	Bladder	Y	N	N	Y	Y	Y	Y	N	Y	Y	Y	Y	N	N	Questionnaire not validated; exposure collected at one time point; exposure not collected before cancer diagnosis; did not consider disease latency; exclusion or enrollment rates unknown
Howe *et al.* (53)	Bladder	Y	N	N	Y	Y	Y	Y	N	Y	Y	Y	Y	Y	Y	Questionnaire not validated; exposure collected at one time point; exposure not collected before cancer diagnosis; did not consider disease latency
Ibiebele *et al.* (78)	EAC, EGAC, and esophageal SCC	N	Y	N	Y	N	Y	Y	N	Y	Y	Y	Y	N	Y	Not specific NSS type (*i.e.*, low calorie soft drinks); exposure collected at one time point; exposure not collected before cancer diagnosis; did not consider frequency, duration, or intake level of NSS consumption; did not consider disease latency, excluded >40% of cases and >50% of controls
Iscovich *et al.* (54)	Bladder	Y	N	N	N	N	Y	Y	N	Y	Y	N	Y	N	N	Questionnaire not validated; exposure collected at one time point; exposure not collected before cancer diagnosis; source of saccharin exposure unclear; did not consider frequency, duration, or intake level of NSS consumption; did not consider disease latency; failed to consider key potential confounders; unknown number of controls excluded
Kantor *et al.* (55)	Bladder	N	N	N	N	Y	Y	Y	N	Y	Y	N	Y	Y	Y	Not specific NSS type (*i.e.*, AS); questionnaire not validated; exposure collected at one time point; exposure not collected before cancer diagnosis; only considered minor source of NSS exposure (*i.e.*, TT);[a] did not consider disease latency; failed to consider key potential confounders

Study	Cancer Type(s)	Study Quality Criteria														
		Intake Assessment					Outcome Assessment				Covariates/ Confounding		Sample Selection			Justification
		Specific NSS	Valid	Timing	Source	Dose	Diagnosis	Incidence	Latency	Cancer Type	Model	Confounders	Controls	Exclusion	Participation	
Kantor *et al*. (56)	Bladder SCC<br>Bladder adenocarcinoma<br>Bladder TCC	N	N	N	N	Y	Y	Y	N	Y	Y	N	Y	Y	Y	Not specific NSS type (*i.e.*, AS); questionnaire not validated; exposure collected at one time point; exposure not collected before diagnosis; only considered minor source of NSS exposure (*i.e.*, TT);[a] did not consider disease latency; failed to consider key potential confounders
Kobeissi *et al*. (58)	Bladder	N	N	N	N	Y	Y	Y	N	Y	N	N	N	N	N	Not specific NSS type (*i.e.*, AS); questionnaire not validated; exposure collected at one time point; exposure not collected before diagnosis; unclear what source of NSS was considered; did not consider disease latency; improper model; failed to consider key potential confounders; exclusion of >25% of cases; unclear how many controls were excluded; inappropriate comparison group (*i.e.*, hospital-based)
Li *et al*. (96)	AML	N	Y	N	Y	Y	Y	Y	N	Y	Y	Y	N	N	N	Not specific NSS type (*i.e.*, diet cola); exposure collected at one time point; exposure not collected before cancer diagnosis; did not consider disease latency; inappropriate controls (*i.e.*, hospital-based); unknown number of exclusions
Kessler and Clark (57)	Bladder	Y	N	N	Y	Y	Y	Y	N	Y	Y	Y	N	Y	Y	Questionnaire not validated; exposure collected at one time point; exposure not collected before cancer diagnosis; insufficient consideration of disease latency (*i.e.*, <1 yr); inappropriate comparison group (*i.e.*, hospital-based)
Maclure and Willett (76)	Renal adenocarcinoma	N	Y	N	Y	Y	Y	Y	N	Y	Y	Y	Y	N	N	Not specific NSS type (*i.e.*, diet soft drinks); exposure collected at one time point; exposure not collected before cancer diagnosis; did not consider disease latency; excluded 83% of cases and 45% of controls; differential (≥15%) participation rates between cases and controls
Mahfouz *et al*. (86)	Colorectal	N	N	N	N	N	Y	Y	N	Y	N	N	Y	N	N	Not specific NSS type (*i.e.*, AS); questionnaire not validated; exposure collected at one time point; exposure not collected before cancer diagnosis; only considered minor source of NSS exposure (*i.e.*, TT);[a] did not consider frequency, duration, or intake level of NSS consumption; did not consider disease latency; improper model and forms of variables; failed to consider key potential confounders; unknown number of excluded participants
Marrett *et al*. (59)	Bladder	N	N	N	Y	Y	Y	Y	N	Y	Y	N	Y	Y	Y	Not specific NSS type (*i.e.*, AS); questionnaire not validated; exposure collected at one time point; exposure not collected before cancer diagnosis; did not consider disease latency; failed to consider key potential confounders
Mayne *et al*. (79)	EAC<br>Esophageal SCC<br>GCA<br>NCGA	N	N	N	Y	Y	Y	Y	N	Y	Y	Y	Y	N	N	Not specific NSS type (*i.e.*, diet CSDs); questionnaire not validated; exposure collected at one time point; exposure not collected before cancer diagnosis; insufficient consideration of disease latency (<4 yrs in some cases); unknown exclusion of subjects
Mettlin (102)	Lung	N	N	N	Y	Y	Y	Y	N	Y	Y	Y	N	N	N	Not specific NSS type (*i.e.*, diet cola); questionnaire not validated; exposure collected at one time point; exposure not collected before cancer diagnosis; did not consider disease latency; unknown number of participants excluded; inappropriate controls (*i.e.*, RPMI subjects with other cancers)
Moller-Jensen *et al*. (41)	Urinary bladder	Y	N	N	Y	Y	Y	Y	Y	Y	Y	N	Y	Y	N	Questionnaire not validated; exposure collected after cancer diagnosis; exposure collected at one time point; authors stated there were few differences between cases and controls, but did not discuss which covariates they considered as potential confounders; low participation rate in controls (71.5%)
Momas *et al*. (60)	Bladder	Y	N	N	N	Y	Y	Y	N	Y	Y	Y	N	Y	Y	Questionnaire not validated; exposure collected at one time point; exposure not collected before cancer diagnosis; only considered minor source of NSS exposure (*i.e.*, tablets only); did not consider disease latency; inappropriate controls (*i.e.*, non-compulsory electoral rolls); 71.7% participation rate in controls not listed in telephone directory
Mommsen *et al*. (61)	Bladder	Y	N	N	N	N	Y	Y	N	Y	Y	Y	Y	Y	N	Questionnaire not validated; exposure collected at one time point; exposure not collected before cancer diagnosis; only considered minor source of NSS exposure (*i.e.*, TT); did not consider frequency, duration, or intake level of NSS consumption; did not consider disease latency; differential (>15%) participation rates between cases and controls
Morgan and Jain (62)	Bladder TCC	N	N	N	Y	Y	Y	Y	N	Y	Y	N	N	N	Y	Not specific NSS type (*i.e.*, AS); questionnaire not validated; exposure collected at one time point; exposure not collected before cancer diagnosis; insufficient consideration of disease latency (*i.e.*, 1 yr); failed to consider key potential confounders; inappropriate comparison group (*i.e.*, hospital-based); exclusion of 31% of cases and >43% of controls

Study	Cancer Type(s)	Study Quality Criteria														
		Intake Assessment					Outcome Assessment				Covariates/ Confounding		Sample Selection			Justification
		Specific NSS	Valid	Timing	Source	Dose	Diagnosis	Incidence	Latency	Cancer Type	Model	Confounders	Controls	Exclusion	Participation	
Morrison (33)	All cancers	N	N	N	N	N	Y	Y	N	N	Y	N	N	N	N	Not specific NSS type (combined cyclamate and saccharin); questionnaire not validated; exposure collected at one time point; exposure not collected before cancer diagnosis; only considered minor source of NSS (*i.e.*, TT); did not consider frequency, duration, or intake level of NSS consumption; did not consider disease latency; failed to consider key potential confounders; inappropriate comparison group (*i.e.*, hospital-based);exclusion and enrollment of cases and controls unknown
Morrison and Buring (34)	Lower urinary tract	N	N	N	Y	Y	Y	Y	Y	N	Y	N	Y	Y	Y	Not specific NSS type (*i.e.*, dietetic beverages, sugar substitutes, and dietetic foods); questionnaire not validated; exposure collected at one time point; exposure not collected before cancer diagnosis; failed to consider key potential confounders
Morrison *et al*. (35)	Lower urinary tract	N	N	N	Y	Y	Y	Y	Y	N	Y	N	N	Y	Y	Not specific NSS type (*i.e.*, sugar substitutes and dietetic foods and beverages); questionnaire not validated; exposure collected at one time point; exposure not collected before cancer diagnosis; failed to consider key potential confounders; inappropriate comparison group (*i.e.*, non-compulsory electoral rolls)
Najem *et al*. (63)	Bladder	Y	N	N	Y	Y	Y	Y	N	Y	N	N	N	Y	Y	Questionnaire not validated; exposure collected at one time point; exposure not collected before cancer diagnosis; did not consider disease latency; Improper model (*i.e.*, unmatched analyses); failed to consider key potential confounders; inappropriate comparison group (*i.e.*, hospital-based)
Nomura *et al*. (36)	Lower urinary tract	Y	Y	N	Y	Y	Y	Y	N	N	Y	Y	Y	Y	N	Exposure collected at one timepoint; exposure not collected before cancer diagnosis; did not consider disease latency; differential (>15%) participation rates between cases and controls
Norell *et al*. (94)	Exocrine pancreas	N	N	N	N	N	Y	Y	N	Y	Y	N	Y	Y	Y	Not specific NSS type (*i.e.*, AS); questionnaire not validated; exposure collected at one time point; exposure not collected before cancer diagnosis; only considered minor source of NSS exposure (*i.e.*, TT);[a] did not consider frequency, duration, or intake level of NSS consumption; did not consider disease latency; failed to consider key potential confounders
Ohno *et al*. (64)	Bladder	N	N	N	N	N	Y	Y	N	Y	Y	N	Y	Y	Y	Not specific NSS type (*i.e.*, AS); questionnaire not validated; exposure collected at one time point; exposure not collected before cancer diagnosis; only considered minor source of NSS exposure (*i.e.*, TT);[a] did not consider frequency, duration, or intake level of NSS consumption; did not consider disease latency; failed to consider key potential confounders
Palomar-Cros *et al.* (47)	Colorectal<br>Stomach<br>CLL<br>Prostate<br>All breast<br>Pre-menopausal breast<br>Post-menopausal breast	Y	Y	N	Y	Y	Y	Y	N	Y	Y	Y	N	Y	Y	Exposure collected at one time point; exposure not collected before cancer diagnosis; did not consider disease latency; inappropriate comparison group (*i.e.*, hospital-based)
Piper *et al*. (65)	Bladder	N	N	N	Y	Y	Y	Y	N	Y	Y	N	Y	N	Y	Not specific NSS type (*i.e.*, ASBs and TT sweeteners); questionnaire not validated; exposure collected at one time point; exposure not collected before cancer diagnosis; did not consider disease latency; failed to consider key potential confounders; unknown number of exclusions
Risch *et al*. (66)	Bladder	Y	N	N	Y	Y	Y	Y	N	Y	Y	N	Y	N	Y	Questionnaire not validated; exposure collected at one time point; exposure not collected before cancer diagnosis; did not consider disease latency; failed to consider key potential confounders; exclusion of 33% of cases and 47% of controls
Schulte *et al*. (67)	Bladder	N	N	N	Y	N	Y	Y	N	Y	N	N	Y	N	N	Not specific NSS type (*i.e.*, AS and diet soda); questionnaire not validated; exposure collected at one time point; unclear if exposure collected before or after diagnosis; did not consider frequency, duration, or intake level of NSS consumption; did not consider disease latency; combined atypical bladder cancer with confirmed bladder cancer cases; improper model or forms of variables; failed to consider key potential confounders; unknown number of subjects excluded

Study	Cancer Type(s)	Study Quality Criteria: Intake Assessment					Outcome Assessment				Covariates/ Confounding		Sample Selection			Justification
		Specific NSS	Valid	Timing	Source	Dose	Diagnosis	Incidence	Latency	Cancer Type	Model	Confounders	Controls	Exclusion	Participation	
Silverman *et al*. (37)	Lower urinary tract	N	N	N	Y	N	Y	Y	N	N	N	Y	Y	Y	N	Not specific NSS type (*i.e.*, TT, diet drinks, diet foods); questionnaire not validated; exposure collected at one time point; exposure not collected before cancer diagnosis; did not consider frequency, duration, or intake level of NSS consumption; did not consider disease latency; improper model (*i.e.*, unmatched analyses); exclusion of 29% of hospital-based controls; inappropriate selection of some controls (*i.e.*, hospital-based); differential (>15%) participation rates between cases and hospital-based controls
Simon *et al*. (38)	Lower urinary tract	Y	N	N	N	N	Y	Y	N	N	N	N	N	Y	Y	Questionnaire not validated; exposure collected at one time point; exposure not collected before cancer diagnosis; only considered minor source of NSS exposure (*i.e.*, TT sweetener); did not consider frequency, duration, or intake level of NSS consumption; did not consider disease latency; improper model (*i.e.*, unmatched analyses); failed to consider key potential confounders; Inappropriate selection of controls (*i.e.*, hospital-based); exclusion of 28% of controls
Singh *et al*. (2020)[b]	WDTC	N	N	N	Y	N	Y	Y	N	Y	Y	N	N	N	N	Not specific NSS type (*i.e.*, AS); questionnaire not validated; exposure collected at one time point; exposure not collected before cancer diagnosis; did not consider frequency, duration, or intake level of NSS consumption; did not consider disease latency; failed to consider key potential confounders; unknown enrollment rate of participants; inappropriate comparison group (*i.e.*, hospital/disease-based)
Sturgeon *et al*. (68)	Bladder	N	N	N	N	Y	Y	Y	N	Y	Y	Y	Y	N	Y	Not specific NSS type (*i.e.*, AS); questionnaire not validated; exposure collected at one time point; exposure not collected before cancer diagnosis; only considered minor source of NSS exposure (*i.e.*, TT);[a] did not consider disease latency; exclusion of >47% of cases and >41% of controls
Sullivan (69)	Bladder	N	N	N	Y	Y	Y	Y	N	Y	N	N	Y	N	N	Not specific NSS type (*i.e.*, ASBs); questionnaire not validated; exposure collected at one time point; exposure not collected before cancer diagnosis; did not consider disease latency; unclear what model was used; failed to consider key potential confounders; unknown number of controls excluded
Theodoratou *et al*. (87)	Colorectal	N	Y	N	Y	Y	Y	Y	N	Y	Y	Y	Y	N	Y	Not specific NSS type (*i.e.*, low calorie drinks); exposure collected at one time point; exposure not collected before cancer diagnosis; did not consider disease latency; exclusion of >25% of participants
Wang *et al*. (70)	Bladder	N	Y	N	Y	Y	Y	Y	N	Y	Y	Y	N	Y	Y	Not specific NSS type (*i.e.*, diet soft drink); exposure collected at one time point; exposure not collected before cancer diagnosis; did not consider disease latency; inappropriate control group (*i.e.*, hospital-based)
Wu *et al*. (88)	Small intestinal adenocarcinoma	N	N	N	N	N	Y	Y	N	Y	Y	N	N	N	N	Not specific NSS type (*i.e.*, AS); questionnaire not validated; exposure collected at one time point; exposure not collected before cancer diagnosis; only considered minor source of NSS exposure (*i.e.*, TT); did not consider frequency, duration, or intake level of NSS consumption; did not consider disease latency; failed to consider key potential confounders; inappropriate controls (*i.e.*, >97% of controls had another cancer); unknown number of participants excluded
Wynder and Goldsmith (40)	Bladder	Y	N	N	N	Y	Y	Y	Y	Y	N	N	N	Y	Y	Questionnaire not validated; exposure not collected before cancer diagnosis; exposure collected at one time point; unknown sources of NSS exposure; improper model (*i.e.*, unclear if it was a matched analyses); failed to consider key potential confounders; inappropriate comparison group (*i.e.*, hospital-based)
Wynder and Stellman (71)	Bladder	Y	N	N	Y	N	Y	Y	N	Y	Y	Y	N	Y	Y	Questionnaire not validated; exposure collected at one time point; exposure not collected before cancer diagnosis; did not consider frequency, duration, or intake level of NSS consumption; did not consider disease latency; inappropriate controls (*i.e.*, hospital-based); unknown number of controls excluded
Wynder *et al*. (93)	Pancreatic	Y	N	N	N	N	Y	Y	N	Y	N	N	N	N	N	Questionnaire not validated; exposure collected at one time point; exposure not collected before cancer diagnosis; only considered minor source of NSS exposure (*i.e.*, TT); did not consider frequency, duration, or intake level of NSS consumption; did not consider disease latency; improper model or forms of variables; failed to consider key potential confounders; inappropriate controls (*i.e.*, hospital-based); unknown number of subjects excluded
Yu *et al*. (39)	Bladder	Y	N	N	N	Y	Y	Y	Y	Y	Y	Y	N	Y	Y	Questionnaire not validated; exposure collected at one time point; exposure not collected before cancer diagnosis; unclear what the NSS sources were considered; inappropriate comparison group (*i.e.*, hospital-based)

Notes:
AS = Artificial Sweetener; ASB = Artificially Sweetened Beverage; CSD = Carbonated Soft Drink; Dx = Diagnosis; EAC = Esophageal Adenocarcinoma; GCA = Gastric Cardia Adenocarcinoma; N = No or Not Reported; NCGA = Noncardia Gastric Carcinoma; NSS = Non-Sugar Sweetener; SCC = Squamous Cell Carcinoma; RPMI = Roswell Park Memorial Institute; TT = Tabletop; Y = Yes; yr = Year.
Quality Assessment Categories: Specific NSS = Assessed Specific NSS (*e.g.*, aspartame) or Specific NSS Can Be Inferred; Valid = Used Validated Questionnaire; Timing = Collected Prior to Cancer Dx; Source = Assessed Major Contributors of AS (*i.e.*, Whole Diet, Beverages); Dose = Considered Frequency, Duration or Level of NSS Consumption; Diagnosis = Physician-Diagnosed, or Self-or Proxy-Reported and Validated Clinically, or Recorded in Medical Records, Death Certificates, or Registries; Incidence = Assessed Disease Incidence; Latency = Sufficient Time Between Exposure and

Outcome to Account for Disease Latency (≥4 Yrs Soft Tissue Tumors, ≥0.5 Yrs Lymphohematopoietic Cancers); Cancer Type = Assessed Specific Cancer Type (*i.e.*, Not Aggregated Cancer Outcome); Model = Proper Model and Forms of Variables; Confounders = Considered Key Potential Confounders; Controls = Appropriate Controls; Exclusion = Exclusion of <25% of Participants or >75% Participation; And Participation = Nondifferential (≤15%) Participation Rates.

(a) TT was assumed, study only mentions evaluation of AS intake and did not define the source (50, 55, 56, 64, 68, 84, 85, 86, 94).

(b) This study is not cited in the main manuscript, but rather only in the supplements. The full reference for this study is listed at the end of the supplements.

Supplement F: Breast Cancer

Cohort Studies

Non-Specific NSSs

Risk estimates for non-specific NSSs and breast cancer ranged from 0.79 to 1.20 across studies, with estimates below and above 1; most were close to 1 and none were statistically significant (20, 27, 29). Romanos-Nanclares et al. (29) examined risk by type of breast cancer and reported no association between NSSs and ER+, ER-, luminal B, HER2, or basal- like breast cancer. They reported a small decreased risk of luminal A breast cancer associated with increasing ASB consumption (p_{trend} = 0.02) (29).

Several studies examined breast cancer risk by menopausal status. Debras et al. (20) reported similar risks for low and high NSS intake compared to no intake (low intake hazard ratio [HR] = 1.11, 95% CI: 0.95-1.30; high intake: 1.16, 95% CI: 0.97-1.38; p_{trend} = 0.064). Results were similar when stratified by menopausal status (pre-menopausal: low intake HR = 1.09, 95% CI: 0.84-1.40; high intake HR = 1.15, 95% CI: 0.89-1.50; post-menopausal: low intake HR = 1.09, 95% CI: 0.89-1.35, high intake HR = 1.20, 95% CI: 0.97-1.50). Hodge et al. (44) examined only post-menopausal breast cancer risk and reported no association with artificially sweetened (AS) soft drink consumption. McCullough et al. (26) only evaluated breast cancer risk in post-menopausal women and reported a small increase in risk with each additional ASB consumed per day (HR = 1.03, 95% CI: 1.00-1.06, p_{trend} = 0.032).

Ace-K

Only Debras et al. (20) evaluated ace-K. Risk estimates ranged from 1.09 to 1.17; none were statistically significant. Results were similar when analyses were stratified by menopausal status.

Aspartame

Debras et al. (20) and Romanos-Nanclares et al. (22) were the only two studies to evaluate aspartame and they reported mixed results. Debras et al. (20) reported a small increased risk of breast cancer for high aspartame consumers (HR = 1.22, 95% CI: 1.01-1.48, $p_{trend} = 0.036$). Results were similar but no longer statistically significant in analyses that stratified by menopausal status (20). Romanos-Nanclares et al. (22) reported no association between any level of aspartame intake and invasive overall, ER-, luminal B, HER-2, or basal-like breast cancers, and a decreased risk of ER+ ($p_{trend} = 0.01$) and luminal A ($p < 0.01$) breast cancers associated with increasing aspartame intake.

Sucralose

Only Debras et al. (20) evaluated sucralose and breast cancer risk, and risk estimates were null for low (HR = 1.04, 95% CI: 0.84-1.30) and high consumers (HR = 0.93, 95% CI: 0.71-1.22) compared to non-consumers ($p_{trend} = 0.786$). Results were similar when stratified by menopausal status (pre-menopausal: low intake HR = 1.30, 95% CI: 0.94-1.79; high intake HR = 1.20, 95% CI: 0.81-1.78, $p_{trend} = 0.144$; post-menopausal: low intake HR = 0.78, 95% CI: 0.56-1.08, high intake HR = 0.88, 95% CI: 0.63-1.23, $p_{trend} = 0.209$).

Case-Control Studies

Non-specific NSSs

Ewertz and Gill (45) reported no association between NSS use in coffee and tea and breast cancer, and noted that "the various types of artificial sweeteners used, mainly saccharin and cyclamate, did not affect the risk of breast cancer." Gallus et al. (46) reported a decreased risk of breast cancer associated with increased intake of AS sachets or tablets combined (p_{trend} = 0.015) and with any use of non-saccharin (mainly aspartame) sachets or tablets (odds ratio [OR] = 0.80, 95% CI: 0.65-0.97). Palomar-Cros et al. (47) reported no association between non-aspartame AS intake and breast cancer overall. Results were similar in analyses stratified by menopausal status.

Aspartame

Only Palomar-Cros et al. (47) evaluated aspartame intake and breast cancer risk. They reported no association with breast cancer overall or among women without diabetes. Among women with diabetes, increased aspartame intake was associated with a decreased risk of breast cancer (p_{trend} = 0.03). There was no association between aspartame intake and either pre- or post-menopausal breast cancer.

Saccharin

Two studies (46, 47) evaluated saccharin and breast cancer risk. Gallus et al. (46) reported no association between saccharin tablet or sachet use and breast cancer (OR = 1.01, 95% CI: 0.77-1.33). Palomar-Cros et al. (47) reported no association with any level of saccharin intake and

breast cancer and no dose-response (p_{trend} = 0.4). They also reported no association between saccharin and breast cancer in analyses stratified by diabetes status.

Supplemental Table F.1 NSS and Breast Cancer Cohort Study Results

Study	Reference Group	Outcome Type	Cancer Type	Statistical Analysis							
				**Risk Metric**	**NSS**	**Group**	**Exposed Cases**	**Expected Cases or Exposed Non-Cases**	**Risk Estimate (95% CI)**	**p_{Trend}**	**Covariate Adjustment**
Debras *et al*. (2022) (20)	Non-consumers	Inc	Breast	HR	Total AS	**Intake Level[a,b]**					Age, sex, BMI, height, % weight gain during follow-up, physical activity, smoking status, number of smoked cigarettes in pack-yrs, educational level, number of 24-hr dietary records, family history of cancer, prevalent diabetes, energy intake without alcohol, daily intake of alcohol, sodium, saturated fatty acids, fiber, sugar, fruit and vegetables, whole-grain foods, and dairy products, and other AS, age at menarche, age at first child, number of biological children, baseline menopausal status, OC use at baseline and during follow-up, and hormonal treatment for menopause at baseline and during follow-up
						Low	229	15,452	1.11 (0.95-1.30)	0.064	
						High	194	15,487	1.16 (0.97-1.38)		
					Aspartame	Low	176	11,823	1.09 (0.92-1.29)	**0.036**	
						High	156	11,844	**1.22 (1.01-1.48)**		
					Acesulfame-K	Low	232	14,346	1.11 (0.95-1.30)	0.086	
						High	166	14,413	1.17 (0.96-1.43)		
					Sucralose	Low	93	5,679	1.04 (0.84-1.30)	0.786	
						High	60	5,690	0.93 (0.71-1.22)		
			Pre-menopausal breast		Total AS	Low	91	12,125	1.09 (0.84-1.40)	0.267	
						High	83	12,134	1.15 (0.89-1.50)		
					Aspartame	Low	73	9,248	1.08 (0.83-1.42)	0.564	
						High	61	9,239	1.07 (0.79-1.46)		
					Acesulfame-K	Low	97	11,207	1.17 (0.91-1.51)	0.441	
						High	67	11,238	1.07 (0.78- 1.48)		
					Sucralose	Low	45	4,520	1.30 (0.94-1.79)	0.144	
						High	30	4,414	1.20 (0.81-1.78)		
			Post-menopausal breast		Total AS	Low	125	5,212	1.09 (0.89-1.35)	0.087	
						High	124	5,214	1.20 (0.97-1.50)		
					Aspartame	Low	100	4,044	1.13 (0.91-1.42)	0.060	
						High	98	4,047	1.24 (0.98-1.57)		
					Acesulfame-K	Low	120	4,922	1.09 (0.88-1.35)	0.211	
						High	114	4,928	1.15 (0.91-1.47)		
					Sucralose	Low	39	1,945	0.78 (0.56-1.08)	0.209	
						High	39	1,946	0.88 (0.63-1.23)		
Hodge *et al*. (2018) (44)	Never or <1/mo	Inc	Post-menopausal breast	HR	Non-specific	**Frequency of AS Soft Drink Consumption[c]**					Age, sex, SEIFA, country of birth, alcohol intake, smoking status, physical activity, Mediterranean diet score, SS soft drink consumption, and waist circumference
						1-3/mo	69	–	0.94 (0.73-1.22)	0.51	
						1-6/wk	101		0.90 (0.72-1.12)		
						≥1/d	60		0.95 (0.73-1.25)		
McCullough *et al*. (2022) (26)	Never consume ASBs	Mort	Post-menopausal breast	HR	Non-specific	**ASB Consumption Among Post-Menopausal Women[d]**					Age, sex, race/ethnicity, smoking, marital status, education, consumption of red and processed meat, fruits and vegetables, alcohol, and SSBs, parity, age at menarche, estrogen use, OC use, age at first live birth, and menopausal status
						<1 drink/d	–	–	1.02 (0.94-1.10)	0.153	
						1 drink/d			0.97 (0.88-1.06)		
						2+ drink/d			1.10 (1.00-1.20)		
						Continuous (per 1 drink/d)			**1.03 (1.00-1.06)**	–	
Mullee *et al*. (2019) (27)	<1 glass/mo[e]	Mort	Breast	HR	Non-specific	**AS Soft Drink Consumption (Glasses)[e]**					Age, sex, EPIC center, BMI, physical activity, education, alcohol consumption, smoking status, intensity, and duration, ever use of contraceptive pill, menopausal status, ever use of menopausal hormone therapy, intakes of total energy, red and processed meat, fruits and vegetables, coffee, fruit and vegetable juice, and SS soft drinks
						1-4/mo	–	–	**0.79 (0.63-0.98)**	0.38	
						>1-6/wk			0.90 (0.74-1.10)		
						≥1/d			0.85 (0.59-1.22)		

Study	Reference Group	Outcome Type	Cancer Type	Statistical Analysis							
				Risk Metric	NSS	Group	Exposed Cases	Expected Cases or Exposed Non-Cases	Risk Estimate (95% CI)	p_{Trend}	Covariate Adjustment
Romanos-Nanclares *et al.* (2021) (29)	<1/mo	Inc	All breast[f]	HR	Non-specific	**Cumulative Average ASB Intake**					Age, calendar yr, SSB intake, race, age at menarche, age at menopause, postmenopausal hormone use, OC use history, parity and age at first birth, breastfeeding history, family history of breast cancer, history of benign breast disease, height, alcohol intake, total caloric intake, physical activity, BMI at age 18 yrs, modified AHEI score, SES, change in weight since age 18, and cohort
						1-4/mo	1,531	–	1.01 (0.95-1.07)	0.08	
						>1-<7/wk	4,197		0.98 (0.94-1.03)		
						≥1/d	2,474		0.96 (0.91-1.02)		
	Non-consumers					Per serving/d	–		0.99 (0.97-1.01)	–	
	<1/mo		ER+			1-4/mo	1,011		1.03 (0.95-1.11)	0.18	
						>1-<7/wk	2,715		0.97 (0.91-1.03)		
						≥1/d	1,562		0.97 (0.90-1.04)		
	Non-consumers					Per serving/d	–		0.97 (0.94-1.00)	–	
	<1/mo		ER-			1-4/mo	213		0.95 (0.81-1.12)	0.92	
						>1-<7/wk	603		1.01 (0.89-1.15)		
						≥1/d	376		0.98 (0.85-1.14)		
	Non-consumers					Per serving/d	–		1.01 (0.97-1.07)	–	
	<1/mo		Luminal A			1-4/mo	381		1.05 (0.93-1.19)	**0.02**	
						>1-<7/wk	939		0.95 (0.86-1.05)		
						≥1/d	563		0.90 (0.80-1.01)		
	Non-consumers					Per serving/d	–		**0.94 (0.90-0.98)**	–	
	<1/mo		Luminal B			1-4/mo	154		1.04 (0.85-1.26)	0.19	
						>1-<7/wk	419		0.98 (0.84-1.14)		
						≥1/d	270		1.12 (0.94-1.33)		
	Non-consumers					Per serving/d	–		1.03 (0.97-1.10)	–	
	<1/mo		HER2			1-4/mo	37		1.04 (0.70-1.55)	0.63	
						>1-<7/wk	86		0.90 (0.66-1.25)		
						≥1/d	46		0.85 (0.58-1.26)		
	Non-consumers					Per serving/d	–		1.03 (0.90-1.18)	–	
	<1/mo		Basal-like			1-4/mo	31		0.81 (0.53-1.22)	0.79	
						>1-<7/wk	109		1.11 (0.83-1.50)		
						≥1/d	74		1.04 (0.74-1.46)		
	Non-consumers					Per serving/d	–		0.97 (0.86-1.09)	–	

Study	Reference Group	Outcome Type	Cancer Type	Statistical Analysis							Covariate Adjustment
				Risk Metric	NSS	Group	Exposed Cases	Expected Cases or Exposed Non-Cases	Risk Estimate (95% CI)	p_{Trend}	
Romanos-Nanclares *et al.* (2024) (22)	No intake	Inc	Invasive	HR	Aspartame	**Quartile of Cumulative Average Intake (mg/d)[g]**					Age, calendar yr, race, age at menarche, age at menopause, postmenopausal hormone use, OC use history, parity and age at first birth, breastfeeding history, family history of breast cancer, history of benign breast disease, BMI at age 18, height, cumulative average physical activity, intake of alcohol, total calories, and total sugar, AHEI score (with SSBs and alcohol removed), neighborhood-based SES, and weight change since age 18
						Quartile 1 (0.18-24.3)	2,470	–	1.01 (0.95-1.07)	0.24	
						Quartile 2 (24.3-77.5)	2,281		0.96 (0.90-1.02)		
						Quartile 3 (77.6-178.1)	2,158		0.96 (0.90-1.02)		
						Quartile 4 (178.1-1,393.3)	1,827		0.97 (0.90-1.03)		
						Per 200 mg/d increase	–		0.99 (0.96-1.02)	–	
			ER+			Quartile 1 (0.18-24.3)	1,761	–	1.05 (0.97-1.13)	**0.01**	
						Quartile 2 (24.3-77.5)	1,620		1.00 (0.93-1.08)		
						Quartile 3 (77.6-178.1)	1,431		0.94 (0.87-1.02)		
						Quartile 4 (178.1-1,393.3)	1,168		0.94 (0.86-1.02)		
						Per 200 mg/d increase	–		**0.95 (0.92-0.99)**	–	
			ER-			Quartile 1 (0.18-24.3)	335	–	0.98 (0.84-1.14)	0.93	
						Quartile 2 (24.3-77.5)	305		0.91 (0.77-1.06)		
						Quartile 3 (77.6-178.1)	346		1.07 (0.91-1.25)		
						Quartile 4 (178.1-1,393.3)	273		0.97 (0.81-1.15)		
						Per 200 mg/d increase	–		1.02 (0.95-1.10)	–	
			Luminal A			Quartile 1 (0.18-24.3)	611	–	1.13 (1.00-1.27)	**<0.01**	
						Quartile 2 (24.3-77.5)	521		0.98 (0.87-1.11)		
						Quartile 3 (77.6-178.1)	449		0.93 (0.82-1.06)		
						Quartile 4 (178.1-1,393.3)	344		**0.84 (0.73-0.97)**		
						Per 200 mg/d increase	–		**0.88 (0.83-0.94)**	–	

Study	Reference Group	Outcome Type	Cancer Type	Statistical Analysis: Risk Metric	NSS	Group	Exposed Cases	Expected Cases or Exposed Non-Cases	Risk Estimate (95% CI)	p_{Trend}	Covariate Adjustment
			Luminal B			Quartile 1 (0.18-24.3)	273	–	1.10 (0.92-1.32)	0.76	
						Quartile 2 (24.3-77.5)	235		0.99 (0.82-1.20)		
						Quartile 3 (77.6-178.1)	209		1.01 (0.82-1.23)		
						Quartile 4 (178.1-1,393.3)	177		1.08 (0.87-1.33)		
						Per 200 mg/d increase	–		1.04 (0.95-1.14)	–	
			HER-2			Quartile 1 (0.18-24.3)	57	–	1.04 (0.72-1.51)	0.82	
						Quartile 2 (24.3-77.5)	45		0.84 (0.56-1.26)		
						Quartile 3 (77.6-178.1)	42		0.90 (0.59-1.36)		
						Quartile 4 (178.1-1,393.3)	37		0.95 (0.61-1.48)		
						Per 200 mg/d increase	–		1.00 (0.82-1.23)	–	
			Basal-like			Quartile 1 (0.18-24.3)	52	–	0.88 (0.61-1.26)	0.06	
						Quartile 2 (24.3-77.5)	57		1.02 (0.71-1.47)		
						Quartile 3 (77.6-178.1)	69		1.34 (0.95-1.91)		
						Quartile 4 (178.1-1,393.3)	55		1.27 (0.87-1.86)		
						Per 200 mg/d increase	–		1.06 (0.90-1.24)	–	

Notes:
AHEI = Alternate Healthy Eating Index; AS = Artificial Sweetener; ASB = Artificially Sweetened Beverage; BMI = Body Mass Index; CI = Confidence Interval; d = Day; EPIC = European Prospective Investigation into Cancer and Nutrition; ER = Estrogen Receptor; HER-2 = Human Epidermal Growth Factor Receptor; hr = Hour; HR = Hazard Ratio; Inc = Incidence; kg = Kilogram; m = Meter; MET = Metabolic Equivalent; mg = Milligram; mL = Milliliter; mo = Month; Mort = Mortality; NHS = Nurses' Health Study; NSS = Non-Sugar Sweetener; OC = Oral Contraceptive; SEIFA = Socio-Economic Indexes for Areas; SES = Socioeconomic Status; SS = Sugar-Sweetened; SSB = Sugar-Sweetened Beverage; wk = Week; yr = Year.
– = Not Reported.
Bolded values indicate statistical significance.
(a) Debras *et al*. (20) also evaluated breast cancer risk for consumers *vs.* non-consumers, using three-category intake models, and 12 sensitivity analyses that considered adjustment for additional confounders, exclusion or restriction of certain participants, and the time-dependent nature of AS exposures, and results were generally similar to those from the main analyses.
(b) Higher *vs*. lower consumers were separated by the median value. For female consumers, the median value of each sweetener was: 19.00 of mg/d total AS, 15.39 mg/d of aspartame, 5.50 mg/d of acesulfame-K, and 3.43 mg/d of sucralose (20).
(c) Hodge *et al*. (44) reported similar associations when excluding the first 2 yrs of follow-up. They did not observe an association when modeling linear trends on a log hazard scale.
(d) In analyses that controlled for BMI (among all subjects and never smokers only), and among smokers only with no control for BMI, the HR per 1 drink/d was no longer statistically significant. When considering BMI status (*i.e*., normal, overweight, or obese), the HR based on 1 drinks/d consumption was statistically significantly decreased (0.84, 95% CI: 0.73-0.97). Sensitivity analyses excluding the first 2 yrs of follow-up or with stratification by 10-yr follow-up time also had minimal impact (26).
(e) One glass = ~250 mL (27).
(f) Romanos-Nanclares *et al*. (29) reported similar risk estimates for those who had no change in beverage consumption over follow-up compared to those whose consumption change. They reported decreasing risk with increasing consumption only with 0-4 yr lag (p_{trend} = 0.03). ASB consumption of >1-<7/wk was associated with a decreased risk of breast cancer (HR = 0.93, 95% CI: 0.88-0.99) in analyses with >12-16 yr lags. Risks did not differ based on menopausal status, BMI, activity level or AHEI level, or when all breast cancers were combined. There was a statistically significant interaction with BMI among ER-positive breast cancers ($p_{interaction}$ < 0.01).
(g) Romanos-Nanclares *et al*. (22) reported that results remained unchanged after excluding cases occurring in the first 10-yrs of follow-up. The authors also evaluated the potential for an interaction by current BMI, menopausal status, alcohol intake, SSB intake, and self-reported history of diabetes among all invasive breast cancers and for ER+ and ER- breast cancers separately. There were no significant interactions reported in analyses of all invasive or ER- breast cancers. There was an interaction reported for ER+ breast cancers and menopausal status ($p_{interaction}$ = 0.04) where a decrease in risk was observed with increasing aspartame among premenopausal women (p_{trend} = 0.02) and no trend was observed among postmenopausal women (p_{trend} = 0.64).

Supplemental Table F.2 NSS and Breast Cancer Case-Control Study Results

Study	Outcome Type	Cancer Type	Statistical Analysis							
			Risk Metric	NSS	Group	Exposed Cases	Exposed Controls	Risk Estimate (95% CI)	p_{Trend}	Covariate Adjustment
Ewertz and Gill (1990) (45)	Inc	Breast	RR	Non-specific	**AS Usage in Coffee and Tea**					Age at diagnosis and place of residence
					No	1,216	1,090	Ref	–	
					Yes	147	133	0.94 (0.73-1.20)		
Gallus *et al*. (2007) (46)	Inc	Breast	OR	Non-specific	**All AS Consumption (Sachets or Tablets/d)[a]**					Age, sex, study center, education, tobacco smoking, alcohol drinking, BMI, total energy intake, consumption of hot beverages, parity, and menopausal status/age at menopause
					0	2,244	2,206	Ref	**0.015**	
					>0-2	210	216	0.97 (0.79-1.19)		
					>2	115	166	**0.70 (0.54-0.91)**		
					Per sachet or tablet/d	–	–	**0.94 (0.89-0.99)**	–	
				Saccharin	**Saccharin Consumption (Sachets or Tablets/d)**					
					0	2,456	2,468	Ref	–	
					>0	113	120	1.01 (0.77-1.33)		
				Non-specific (mainly aspartame)	**AS (Other Than Saccharin) Consumption (Sachets or Tablets/d)**					
					0	2,350	2,318	Ref	–	
					>0	219	270	**0.80 (0.65-0.97)**		
Palomar-Cros *et al*. (2023) (47)	Inc	All breast	OR	Aspartame[b]	**Level of Intake[c]**					Age, study center, education, smoking, radiation exposure, total WCRF score continuous, total energy intake, total sugar intake, family history of breast cancer, night shift work, menopause, nulliparity, age at first child, use of hormonal contraceptives, and aspartame or other AS consumption[d]
					All Participants					
					Non-consumers	1,146	1,255	Ref	0.2	
					Medium	265	313	0.82 (0.67-1.01)		
					High	99	106	0.94 (0.69-1.28)		
					Participants Without Diabetes					
					Non-consumers	1,065	1,159	Ref	0.4	
					Medium	242	286	0.81 (0.66-1.01)		
					High	91	89	1.04 (0.75-1.45)		
					Participants with Diabetes					
					Non-consumers	81	96	Ref	**0.03**	
					Medium	23	27	0.73 (0.33-1.57)		
					High	8	17	**0.28 (0.08–0.83)**		
				Other AS (excluding aspartame)[b,e]	*All Participants*					
					Non-consumers	924	995	Ref	0.3	
					Medium	434	501	0.95 (0.8-1.13)		
					High	152	178	0.90 (0.7-1.16)		

Study	Outcome Type	Cancer Type	Statistical Analysis							
			Risk Metric	NSS	Group	Exposed Cases	Exposed Controls	Risk Estimate (95% CI)	p_{Trend}	Covariate Adjustment
				Saccharin	*All Participants*					Age, sex, study center, education, smoking, radiation exposure, total WCRF score continuous, total energy intake, total sugar intake, and other sources of sweeteners
					Non-consumers	1,101	1,192	Ref	0.4	
					Medium	272	313	1.00 (0.82-1.21)		
					High	137	169	0.88 (0.67-1.13)		
					Participants Without Diabetes					
					Non-consumers	1,055	1,137	Ref	0.3	
					Medium	228	258	0.97 (0.79-1.20)		
					High	115	139	0.86 (0.65-1.14)		
					Participants with Diabetes					
					Non-consumers	46	55	Ref	0.9	
					Medium	44	55	0.99 (0.51-1.94)		
					High	22	30	0.93 (0.40-2.11)		
		Premenopausal breast		Aspartame[b]	Non-consumers	377	309	Ref	0.1	Age, study center, education, smoking, radiation exposure, total WCRF score continuous, total energy intake, total sugar intake, family history of breast cancer, night shift work, menopause, nulliparity, age at first child, use of hormonal contraceptives, and aspartame or other AS consumption[c]
					Medium	121	126	0.80 (0.57-1.13)		
					High	43	48	0.74 (0.45-1.21)		
				Other AS (excluding aspartame)[d]	Non-consumers	351	295	Ref	0.6	
					Medium	136	136	0.86 (0.62-1.20)		
					High	54	52	0.97 (0.61-1.55)		
		Postmenopausal breast		Aspartame[b]	Non-consumers	769	946	Ref	0.8	
					Medium	144	187	0.84 (0.65-1.10)		
					High	56	58	1.15 (0.76-1.74)		
				Other AS (excluding aspartame)[b]	Non-consumers	573	700	Ref	0.4	
					Medium	298	365	0.97 (0.79-1.20)		
					High	98	126	0.87 (0.63-1.19)		

Notes:
AS = Artificial Sweetener; ASB = Artificially Sweetened Beverage; BMI = Body Mass Index; CI = Confidence Interval; d = Day; Inc = Incidence; NSS = Non-Sugar Sweetener; OR = Odds Ratio; Ref = Reference; RR = Relative Risk; TT = Tabletop; WCRF = World Cancer Research Fund.
– = Not Reported.
Bolded values indicate statistical significance.
(a) Gallus *et al*. (46) also conducted analyses for continuous AS consumption (*i.e*., per sachet or tablet/d) and stratified by BMI and age. An association was only observed among individuals with a BMI <25 kg/m^2 (OR = 0.90, 95% CI: 0.83-0.99).
(b) Palomar-Cros *et al*. (47) used "public sources of nutritional information (https://es.openfoodfacts.org/) to determine the most common type of sweetener in each of these food items." They combined non-saccharin TT use, which they reported to be primarily aspartame, and low or no calorie soft drinks into the aspartame category. For the "Other AS" intake category the authors combined TT saccharin and gaseosa, which is an ASB beverage in Spain that reported is usually sweetened with saccharin and cyclamate.
(c) Sex-specific quartiles among consumers and controls were used to compare moderate (<3rd quartile) and high (≥3rd quartile) consumers to non-consumers (reference) (47).

(d) Sensitivity analyses adjusting for individual confounders (*i.e*., BMI, dietary fiber, red meat, physical activity, and alcohol) instead of the WCRF score, with and without adjusting for BMI, yielded similar results. Adjustment for individual confounders in addition to weight change from the prior year yielded a lower risk for all breast cancers associated with medium intake of aspartame-containing products among all participants (OR = 0.79, 95% CI: 0.64-0.98) (47).
(e) Results stratified by diabetes status were similar and not statistically significant. In sensitivity analyses, when considering only the consumption of low- or no-calorie soft drinks, some lower odds for all breast cancers were observed among all participants and participants without diabetes (47).

Supplement G: Liver Cancer

Cohort Studies

Non-specific NSSs

All four cohort studies evaluated NSSs mixtures. Risk estimates ranged from 0.78 to 1.18; most were close to 1 (25, 26, 48, 49). All four studies evaluated risk by level of NSS intake. Jones et al. (25) evaluated risk of liver cancer associated with the intake of diet sweetened beverages, diet soda, or diet fruit punch stratified by diabetes status. They reported a small increased risk of liver cancer associated with each additional diet sweetened beverage per day (HR = 1.13, 95% CI: 1.02-1.25) and diet soda per day (HR = 1.13, 95% CI: 1.01-1.27) among participants with diabetes followed for ≤12 yrs. There were no increased risks associated with increased consumption of all diet sweetened beverages or diet sodas among participants with diabetes followed >12 years, or among participants without diabetes for either follow-up duration. There were also no associations with diet fruit punch among those with or without diabetes regardless of follow-up duration. Stepien et al. (49) reported a small increased risk of hepatocellular carcinoma (HCC) associated with each increased serving of ASB per week (HR = 1.06, 95% CI: 1.03-1.09).

In contrast, Zhao et al. (48) reported no increased risk of liver cancer among those consuming 1 to 6 ASBs per week (HR = 0.99, 95% CI: 0.65-1.51) or those consuming ≥1 ASB per day (HR = 1.17, 95% CI: 0.70-1.94), and no overall trend (p_{trend} = 0.66). McCullough et al. (26) reported no increased risk of liver cancer with increased ASB consumption with any level of intake and no trend overall (p_{trend} = 0.256), or in analyses of men (p_{trend} = 0.083) or women (p_{trend} = 0.964) separately. In analyses stratified by sex and smoking status, the authors reported no increased risks

for male and female non-smokers combined, or female non-smokers, regardless of BMI status. The reported statistically significant trend in male non-smokers ($p_{trend} = 0.040$) is inconsistent with all other permutations evaluated and was no longer significant after adjustment for body mass index (BMI) ($p_{trend} = 0.335$). In analyses excluding the first 2 years of follow-up, or stratified by 10-years of follow-up time, McCullough et al. (26) did not report any increased risk of liver cancer associated with ASBs.

Supplemental Table G.1 NSS and Liver Cancer Cohort Study Results

Study	Reference Group	Outcome Type	Cancer Type	Statistical Analysis: Risk Metric	NSS	Group	Exposed Cases	Expected Cases or Exposed Non-Cases	Risk Estimate (95% CI)	p_{Trend}	Covariate Adjustment
Jones *et al*. (2022) (25)	–	Inc	Liver	HR	Non-specific	**Diet Sweetened Beverage Consumption (per 1/d) by Yrs of Follow-up**					Age, sex, race/ethnicity, BMI, smoking, alcohol use, study, and total energy intake
						Without Diabetes					
						≤12 yrs	–	–	1.01 (0.92-1.11)	–	
						>12 yrs			0.99 (0.86-1.15)		
						With Diabetes					
						≤12 yrs	–	–	**1.13 (1.02-1.25)**	–	
						>12 yrs			0.82 (0.64-1.05)		
						**Diet Soda Consumption (per 1/d) by Yrs of Follow-up**					
						Without Diabetes					
						≤12 yrs	–	–	1.00 (0.90-1.11)	–	
						>12 yrs			0.98 (0.83-1.15)		
						With Diabetes					
						≤12 yrs	–	–	**1.13 (1.01-1.27)**	–	
						>12 yrs			0.78 (0.59-1.03)		
						**Diet Fruit Punch Consumption (per 1/d) by Yrs of Follow-up**					
						Without Diabetes					
						≤12 yrs	–	–	1.06 (0.80-1.41)	–	
						>12 yrs			1.11 (0.74-1.65)		
						With Diabetes					
						≤12 yrs	–	–	1.17 (0.92-1.48)	–	
						>12 yrs			1.01 (0.61-1.69)		

Study	Reference Group	Outcome Type	Cancer Type	Statistical Analysis							Covariate Adjustment
				Risk Metric	NSS	Group	Exposed Cases	Expected Cases or Exposed Non-Cases	Risk Estimate (95% CI)	p_{Trend}	
McCullough *et al*. (2022) (26)	Never consume ASBs	Mort	Liver	HR	Non-specific	**ASB Consumption**[a]					Age, sex, race/ethnicity, smoking, marital status, education, consumption of red and processed meat, fruits and vegetables, alcohol, and SSBs
						<1 drink/d	–	–	1.09 (0.96-1.23)	0.256	
						1 drink/d			1.09 (0.94-1.26)		
						2+ drink/d			1.05 (0.90-1.22)		
						Continuous (per 1 drink/d)			1.01 (0.97-1.05)	–	
						Men					
						<1 drink/d	–	–	1.18 (0.99-1.41)	0.083	
						1 drink/d			1.16 (0.95-1.43)		
						2+ drink/d			1.12 (0.90-1.39)		
						Continuous (per 1 drink/d)			1.02 (0.97-1.09)	–	
						Women					
						<1 drink/d	–	–	1.01 (0.85-1.19)	0.964	
						1 drink/d			1.02 (0.83-1.25)		
						2+ drink/d			1.00 (0.80-1.24)		
						Continuous (per 1 drink/d)			1.00 (0.94-1.07)	–	
Stepien *et al*. (2016) (49)	–	Inc	HCC	HR	Non-specific	**ASB Intake**[b]					Age, sex, study center, non-alcoholic energy intake, BMI, sex-specific physical activity, education level, alcohol intake at recruitment and alcohol intake pattern, smoking intensity, duration, and history, and diabetes status
						Continuous (per serving/wk)	101	–	**1.06 (1.03-1.09)**	–	

Study	Reference Group	Outcome Type	Cancer Type	Statistical Analysis							
				Risk Metric	NSS	Group	Exposed Cases	Expected Cases or Exposed Non-Cases	Risk Estimate (95% CI)	p_{Trend}	Covariate Adjustment
Zhao *et al*. (2023) (48)	Never consume to ≤3 servings/mo	Inc	Liver	HR	Non-specific	**ASB Consumption Category (Servings)[c]**					Age, sex, total energy intake, race and ethnicity, education, smoking status, alcohol consumption, BMI, physical activity, NSAID use, family history of cancer, prior OC use, postmenopausal hormone therapy, and self-reported diabetes
						1-6/wk	–	–	0.99 (0.65-1.51)	0.66	
						≥1/d			1.17 (0.70-1.94)		

Notes:

AHEI = Alternative Healthy Eating Index; AmpV = Ampulla of Vater; ASB = Artificially Sweetened Beverage; BMI = Body Mass Index; CI = Confidence Interval; d = Day; EBD = Extrahepatic Bile Duct; GB = Gallbladder; GBTC = Gallbladder and Biliary Tract Cancer; HCC = Hepatocellular Carcinoma; HR = Hazard Ratio; IHBC = Intrahepatic Bile Duct Carcinoma; Inc = Incidence; mo = Month; Mort = Mortality; NSAID = Non-Steroidal Anti-Inflammatory Drug; NSS = Non-Sugar Sweetener; OC = Oral Contraceptive; SSB = Sugar-Sweetened Beverage; wk = Week; yr = Year.

– = Not Reported.

Bolded values indicate statistical significance.

(a) In analyses conducted among never smokers only, only the p_{trend} for men was statistically significant (0.040; increasing HR with increasing consumption). When analyses controlled for BMI (among all subjects or never smokers), or by BMI status (*i.e*., normal, overweight, or obese), no results were statistically significant. Sensitivity analyses excluding the first 2 yrs of follow-up or with stratification by 10-yr follow-up time produced similar results (26).

(b) Authors conducted stratified analyses for other liver cancer subtypes (IHBC, GBTC, EBD, GB, and AmpV) but stated that they only reported the risk estimate for HCC because the finding was statistically significant (49).

(c) Results were similar after additional adjustment for coffee or tea intake, history of liver diseases, modified AHEI, added sugar, waist-hip ratio, or if excluding adjustments for BMI and self-reported diabetes. Results were also similar after excluding cases diagnosed in the first 2 yrs of follow-up, those with liver diseases or diabetes at baseline, participants in clinical trials, or participants with alcohol intake of 1+ drinks/d (48).

Supplement H: Urinary System

Bladder Cancer

Cohort Studies

Non-specific NSS

Both cohort studies only evaluated non-specific ASB intake and bladder cancer risk (26, 31), and neither reported any associations. Most risk estimates were close to 1, ranging from 0.94-1.09. Ringel et al. (31) reported no increased risk of bladder cancer incidence among frequent (1-6 times/week) (HR = 0.99, 95% CI: 0.78-1.26) or daily (HR = 0.75, 95% CI: 0.53-1.06) ASB consumers compared to people who consumed fewer than 1 ASBs/week. McCullough et al. (26) reported no increased risk with increased ASB intake overall (p_{trend} = 0.7984) or in analyses stratified by sex.

Case-Control Studies

Non-specific NSS

Twenty case-control studies evaluated NSS mixtures and bladder cancer risk (42, 50-53, 55, 56, 58, 59, 62-70). Although not specified, NSSs consumed before 1982, which was the focus of many of these studies, were almost exclusively saccharin.

All studies that evaluated the overall risk (*e.g.*, ever *vs.* never, any *vs.* none, higher *vs.* lower) between ever or any intake of NSSs and bladder cancer reported no associations (42, 50, 63, 65, 67). Sullivan (69) reported that, overall, cases had an earlier age of first ASB intake, a longer

duration of intake, and more glasses of ASB/week compared to controls (all $p \leq 0.03$). However, the authors noted that "the number of glasses of artificially sweetened beverage per week actually consumed by the patients [cases] who drank these products and the actual number of years of consumption of those who drank artificially sweetened beverages were not significantly different from those in the control group."

Sturgeon et al. (68) evaluated bladder cancer risk by tumor grade and invasiveness. Compared to those consuming <1,689 mg/day of NSS, there was no increased risk in those consuming ≥1,689 mg/day of NSS of either low (I/II) or high (III/IV) grade invasive, or low grade noninvasive, bladder cancer, but there was an increased risk of noninvasive high grade bladder cancer (relative risk [RR] = 3.0, 95% CI: 1.5-6.3).

Several studies evaluated the risk of bladder cancer in analyses stratified by sex and reported inconsistent results. Howe et al. (53), Hoover and Strasser (42), and Marrett et al. (59) reported no increased risk of bladder cancer for males or females who consumed NSSs. Two studies reported decreased sex-specific risks of bladder cancer with NSS intake. Morgan and Jain (62) reported no increased risk of transitional cell carcinoma (TCC) associated with prolonged regular AS use (>1 year) among men (RR = 1.0, CI: not reported) and a decreased risk among women (RR = 0.35, CI: not reported). One study (64) reported a decreased risk of bladder cancer among men (RR = 0.67, 95% CI: 0.49-0.93) and women (RR = 0.45, 95% CI: 0.24-0.84) with ever use of AS. Finally, Howe et al. (52) reported no association with ever use of AS among females (OR = 0.6 CI: not reported) but an increased risk among males (OR = 1.6, CI: not reported). Howe et al.

(52) reported a similar magnitude of risk for ever consuming diet food as were reported for ever AS use, but results were not statistically significant for either sex.

Five studies examined levels of NSS intake and bladder cancer risk. Results were mixed. Two studies reported no increased risk of bladder cancer with increased NSS intake. Wang et al. (70) reported no increased risk with increased diet soft drink consumption (p_{trend} = 0.713), and in unadjusted analyses Kobeissi et al. (58) reported no association with frequency of NSS intake (χ^2 p = 0.074).

Howe et al. (53) reported an increased risk of bladder cancer in those consuming the highest amount of tabletop AS tablets per day (≥9 tablets) (OR = 2.8, CI: not reported), but no association in those consuming <9 tablets per day. Kantor et al. (55) evaluated risk of bladder cancer with level of AS intake and family history of UTC. Compared to those with a family history of UTC and no AS use (reference group), there was an increased risk of bladder cancer among those with a family history and no AS use (OR = 1.5, 95% CI: 1.1-2.1) and among those with a family history of UTC consuming 240-719 mg/week (OR = 2.0, 95% CI: 1.1-3.6), but not for those with a family history of UTC consuming <240 mg/week (OR = 1.5, 95% CI: 0.8-2.7). There was also an increased risk among those with a family history of UTC consuming the highest amount of AS (≥720 mg/week) (OR = 2.1, 95% CI: 1.0-4.4).

Kantor et al. (56) was the only study to assess several subtypes of bladder cancer (*i.e.*, squamous cell carcinoma [SCC], adenocarcinoma, and TCC). They reported no associations with any level of AS intake and any subtype.

Three studies examined bladder cancer risk with level of NSS intake stratified by sex and reported mixed results. Bruemmer et al. (51) reported no increased risk with increasing intake for either sex. Risch et al. (66) reported no association between intake of low-calorie foods in males or females. They reported a small decreased risk in males consuming <4 diet sodas per week compared to never consumers (OR = 0.44, 95% CI: 0.21-0.89), but there was no association observed among higher consumers or among women.

Hoover and Strasser (42) reported an increased risk with increased average daily use of tabletop sweeteners among females ($p_{trend} = 0.03$) but not males. Risk estimates ranged from 0.73 to 1.42 across categories of intake, but none were statistically significant. The authors reported no increased risk in either sex with increased number of daily servings of diet drinks. There were no CIs presented and no trend among males or with increased daily servings of diet drinks or any combination of tabletop sweetener use level and number of diet drinks per day. Hoover and Strasser (42) conducted several sub-analyses in white males and females and reported an increased risk among low-risk (*i.e.*, never smoked cigarettes, handled dye, rubber, leather, ink, or paint on any job) white women with increased daily tabletop use ($p_{trend} <0.01$), duration of ≥2 tabletop uses ($p_{trend} <0.01$), and duration of ≥2 diet drinks per day ($p_{trend} <0.05$), but not with average daily servings of diet drinks ($p_{trend} = 0.14$). They reported an increased risk with increasing tabletop use or diet drink intake (both $p_{trends} < 0.01$) among white men who smoked >40 cigarettes/day. No associations were reported between bladder cancer and NSS use among non-smoking males or female smokers. It should be noted the numbers of cases in these sub-analysis categories were quite small (*e.g.*, only three cases consumed ≥3 diet drinks per day).

Three studies evaluated saccharin and cyclamate intake combined and bladder cancer risk (41, 57, 66). Kessler and Clark (57) reported no increased risk of bladder cancer incidence with any use of either saccharin or cyclamate (OR = 1.03, 95% CI: 0.77-1.38) or saccharin and cyclamate (OR = 1.00, 95% CI: 0.73-1.37). In analyses stratified by source of NSS intake, there were no increased risks reported for cyclamate and saccharin used as tabletop sweeteners (OR = 0.89, 95% CI: 0.67-1.18), in diet beverages (OR = 0.97, 95% CI: 0.72-1.31), in diet foods (OR = 1.02, 95% CI: 0.72-1.45) or from all sources (*i.e.*, total NSS consumption) (OR = 0.98, 95% CI: 0.75-1.28). Results were similar in analyses stratified by sex, and there were no associations reported across any level of intake. The only association reported by Kessler and Clark (57) was an increased risk in non-smoking men (OR = 2.61, 95% CI: 1.20-5.67), but there was no increased risk reported for male smokers, or female smokers, or non-smokers. Moller-Jensen et al. (41) reported no increased risk of urinary bladder cancer incidence with ever use of saccharin and cyclamate among all participants (OR = 0.78, 95% CI: 0.58-1.05). They also did not report any increased risks for females with any regular use (OR = 1.03, 95% CI: 0.59-1.79) or with any duration of use or with increased amount of tabletop use/day. In contrast, Moller-Jensen et al. (41) reported a decreased risk among males with regular use of any saccharin and cyclamate (OR = 0.69, 95% CI: 0.49-0.98) or regular tabletop use (OR = 0.66, 95% CI: 0.46-0.95). They did not report any associations with amount of tabletop use/day, but they report a decreased risk among those with the longest duration of use (≥15 years) (OR = 0.48, 95% CI: 0.23-0.98). Risch et al. (66) reported no association between tabletop use of cyclamate or saccharin use and ever use in males (OR = 0.95, 95% CI: 0.72-1.25) or females (OR = 1.15, 95% CI: 0.75-1.26). Results were similar when limited to nonsmokers or those who reported ever use over 10 years prior to diagnosis.

Cyclamate

Three case-control studies evaluated cyclamate intake and bladder cancer risks (41, 57, 66). Kessler and Clark (57) reported no association between any use of cyclamate in all participants (OR = 0.97, 95% CI: 0.71-1.32) or in males (OR = 1.15, 95% CI: 0.78-1.70) or females (OR = 0.61, 95% CI: 0.34-1.14) separately. Neither Moller-Jensen et al. (41) or Risch et al. (66) reported an increased risk in analyses stratified by sex (neither evaluated bladder cancer risk in males and females combined). Moller-Jensen et al. (41) reported no association between ever use of cyclamate in males (OR = 0.72, 95% CI: 0.26-2.04) or females (OR = 1.33, 95% CI: 0.22-8.13). Risch et al. (66) reported no increased risk with increased daily tabletop use among males (>0-1 use/day OR = 0.54, 95% CI: 0.21-1.44; >1 use/day OR = 1.44, 95% CI: 0.55-3.77) or females (>0-1 use/day OR = 1.57, 95% CI: 0.53—4.66; >1 use/day OR = 1.21, 95% CI: 0.32-4.54). They also reported no increased risk with each 20-years of lifetime use for either sex (males OR = 1.09, 95% CI: 0.60-1.97; females OR = 0.92, 95% CI: 0.63-1.36).

Saccharin

Saccharin intake and bladder cancer was evaluated in 13 case-control studies (39-41, 43, 52-54, 57, 60, 61, 63, 66, 71).

Most of the studies reported no association overall (54, 57, 60, 63). Mommsen et al. (61) reported no statistically significant association with bladder cancer (RR = 7.52, CI: not reported). Notably,

there were a very small number of cases and controls who reported any saccharin intake in this study (*i.e.*, 6 cases, 2 controls).

Only two studies evaluated risk of bladder cancer with levels of saccharin intake in all participants. Howe et al. (53) reported an increased risk of bladder cancer in those consuming the highest amount of tabletop AS tablets per day (>8 tablets) (OR = 3.1, CI: not reported), but no association in those consuming 8 or fewer tablets per day. Yu et al. (39) reported an increased risk of bladder cancer with increasing frequency of saccharin use (p_{trend} = 0.0007) and with duration of saccharin use (p_{trend} = 0.0001).

Four studies evaluated bladder cancer and saccharin stratified by sex. Three of the studies reported no association between saccharin intake and bladder cancer risk in either sex (41, 57, 71). Cartwright et al. (43) reported an increased risk of bladder cancer among non-smoking males (RR = 2.2, 95% CI: 1.3-3.8) but not among males who smoked (RR = 0.9, 95% CI: 0.6-1.3), or among female smokers or non-smokers.

Three studies examined bladder cancer risk with increasing saccharin intake by sex. Risch et al. (66) reported no associations between any level of saccharin intake and bladder cancer for either males or females. Risch et al. (66) and Wynder and Goldsmith (40) reported no association with increased duration of intake in either sex. Howe et al. (52) reported an increased risk of bladder cancer with increased average frequency of tablet use (p_{trend} = 0.018) and increased duration of use (p_{trend} = 0.031), but there were no associations reported for any level of tablet use (*i.e.*, <2,500 or

≥2,500 per year) when stratified by duration of use (*i.e.*, <3 years, or ≥3 years). Howe et al. (52) did not report risks with saccharin use for females.

Kidney Cancer

Cohort Studies

Non-specific NSS

All five cohort studies evaluated non-specific NSS intake and kidney cancer. Heath et al. (74) reported no increased risk of renal cell carcinoma (RCC) incidence in all subjects (HR = 1.02, 95% CI: 0.96-1.08), men (OR = 0.99, 95% CI: 0.91-1.08), and women (OR = 1.05, 95% CI: 0.96-1.14). They reported similar results for risk of RCC mortality. Lee et al. (24) also reported no increased risk of RCC incidence with each increase in NSS serving/day (RR = 0.97, 95% CI: 0.82-1.15). Hodge et al. (44) and McCullough et al. (26) reported no increased risk with any level of ASB intake (range of risk estimates: 0.66-1.08), and neither reported a trend with increasing intake (*i.e.*, both studies reported p_{trend} >0.4). McCullough et al. (26) reported similar risk estimates in analyses stratified by sex. Ringel et al. (31) reported an increased risk of incident kidney cancer among those with frequent ASB intake (1-6/week) (HR = 1.34, 95%: 1.03-1.75), but no increased risk among those with daily ASB intake (≥1/day) (HR = 1.14, 95% CI: 0.80-1.62).

Case-Control Studies

Non-specific NSS

All four case-control studies estimated kidney cancer risks associated with various NSS intake. Maclure and Willett (76) reported an increased risk of renal adenocarcinoma incidence with increased diet soft drink intake (p_{trend} = 0.03), and in those drinking ≥2 cups/day compared to those drinking 0-2 cups/month (OR = 2.7, 95% CI: 1.1-6.5), but no increased risk among those drinking 1-7 cups/week (OR = 1.2, 95% CI: 0.7-2.0). When the lowest two intake categories were combined

as the reference (<2 cups/day), the risk in those consuming ≥2 cups/day was no longer significant (OR = 1.4, 95% CI: 0.68-2.9). Goodman et al. (75) reported no increased risk of RCC incidence associated with any consumption of diet beverage (OR = 0.95, 95% CI: 0.65-1.38) or any level of lifetime AS use (1.99 g OR = 0.93, 95% CI: 0.56-1.53; ≥200 g OR = 0.94, 95% CI: 0.59-1.48). Results for consumption of diet beverage and lifetime AS use were similar when stratified by sex. Gallus et al. (46) reported no increased risk of RCC incidence with increased intake of any AS sachets or tablets (p_{trend} = 0.632). Gallus et al. (46) also evaluated the risk of RCC with any intake of non-saccharin AS sachets or tablets, which they reported to be mostly aspartame (OR = 1.03, 95% CI: 0.73-1.46).

Asal et al. (77) reported an increased risk of RCC incidence with ever use of saccharin or cyclamate among males (OR = 2.1, 95% CI: 1.3-3.5), but not females (OR = 1.3, 95% CI: 0.7-2.4).

Saccharin

Two case-control studies evaluated saccharin intake and RCC. Gallus et al. (46) reported no association with any saccharin use (OR = 0.79, 95% CI: 0.49-1.28) and Goodman et al. (75) reported no association with ≥30 mg of saccharin/day (OR = 1.29, 95% CI: 0.86-1.94). Goodman et al. (75) reported similar results in analyses stratified by sex.

Supplemental Table H.1 NSS and Bladder Cancer Cohort Study Results

Citation	Reference Group	Outcome Type	Cancer Type	Statistical Analysis							Covariate Adjustment
				Risk Metric	NSS	Group	Exposed Cases	Expected Cases or Exposed Non-Cases	Risk Estimate (95% CI)	p_{Trend}	
McCullough *et al*. (2022) (26)	Never consume ASBs	Mort	Bladder	HR	Non-specific	**ASB Consumption**[a]					Age, sex, race/ethnicity, smoking, marital status, education, consumption of red and processed meat, fruits and vegetables, alcohol, and SSBs
						<1 drink/d	–	–	0.94 (0.83-1.06)	0.794	
						1 drink/d			0.99 (0.86-1.14)		
						2+ drink/d			1.04 (0.90-1.20)		
						Continuous (per 1 drink/d)			1.00 (0.95-1.04)	–	
						Men					
						<1 drink/d	–	–	0.94 (0.80-1.10)	0.574	
						1 drink/d			0.97 (0.81-1.17)		
						2+ drink/d			1.09 (0.91-1.31)		
						Continuous (per 1 drink/d)			1.00 (0.95-1.05)	–	
						Women					
						<1 drink/d	–	–	0.96 (0.79-1.16)	0.784	
						1 drink/d			1.02 (0.81-1.29)		
						2+ drink/d			0.96 (0.75-1.23)		
						Continuous (per 1 drink/d)			0.98 (0.92-1.06)	–	
Ringel *et al*. (2023) (31)	Rare consumers (never or <1/wk)	Inc	Bladder	HR	Non-specific	**ASB Consumption Rate**					Age, sex, race and ethnicity, nSES, smoking, and water consumption
						Frequent (1-6/wk)	106	–	0.99 (0.78-1.26)	–	
						Daily (≥1/d)	47		0.75 (0.53-1.06)		

Notes:
ASB = Artificially Sweetened Beverage; BMI = Body Mass Index; CI = Confidence Interval; d = Day; HR = Hazard Ratio; Inc = Incidence; Mort = Mortality; nSES = Neighborhood Socioeconomic Status; NSS = Non-Sugar Sweetener; SSB = Sugar-Sweetened Beverage; wk = Week; yr = Year.
– = Not Reported.
(a) In analyses that additionally controlled for BMI, were conducted among never smokers only, or were stratified by BMI status (*i.e.*, normal, overweight, or obese), all results were not statistically significant. Sensitivity analyses excluding the first 2 yrs of follow-up or with stratification by 10-yr follow-up time also had minimal impact (26).

Supplemental Table H.2 NSS and Kidney Cancer Cohort Study Results

Study	Reference Group	Outcome Type	Cancer Type	Statistical Analysis: Risk Metric	NSS	Group	Exposed Cases	Expected Cases or Exposed Non-Cases	Risk Estimate (95% CI)	p_{Trend}	Covariate Adjustment
Heath *et al*. (2021) (74)	Unexposed	Inc	RCC	HR	Non-specific	**Continuous (per 100 g/d)[a]**					Age, sex, country, education attainment, smoking status, alcohol consumption, physical activity, juice intake, total soft drink intake, sugar-sweetened soft drink, BMI, and total energy intake
						All subjects	589	–	1.02 (0.96-1.08)	–	
						Women	242		1.05 (0.96-1.14)		
						Men	347		0.99 (0.91-1.08)		
		Mort				All subjects	265		1.06 (0.99-1.14)		
						Women	123		1.08 (0.98-1.19)		
						Men	142		1.04 (0.93-1.15)		
Hodge *et al*. (2018) (44)	Never or <1/mo	Inc	Kidney	HR	Non-specific	**Frequency of AS Soft Drink Consumption[b]**					Age, sex, SEIFA, country of birth, alcohol intake, smoking status, physical activity, Mediterranean diet score, SS soft drink consumption, and waist circumference
						1–3/mo	11	–	0.71 (0.38-1.36)	0.48	
						1–6/wk	12		0.66 (0.36-1.22)		
						>1/d	9		0.92 (0.46-1.84)		
Lee *et al*. (2006) (24)	Unexposed	Inc	RCC	RR	Non-specific	Per 1 serving/d	–	–	0.97 (0.82-1.15)	–	Age, sex, calendar time, BMI, history of hypertension, parity (NHS only), history of diabetes (NHS only), smoking status, total energy intake, multivitamin use (HPFS only), and alcohol intake
McCullough *et al*. (2022) (26)	Never consume ASBs	Mort	Kidney	HR	Non-specific	**ASB Consumption[c]**					Age, sex, race/ethnicity, smoking, marital status, education, consumption of red and processed meat, fruits and vegetables, alcohol, and SSBs
						<1 drink/d	–	–	0.98 (0.87-1.11)	0.412	
						1 drink/d			1.08 (0.94-1.24)		
						2+ drink/d			1.04 (0.90-1.20)		
						Continuous (per 1 drink/d)			1.01 (0.97-1.05)	–	
						Men					
						<1 drink/d	–	–	0.96 (0.80-1.14)	0.377	
						1 drink/d			1.09 (0.90-1.33)		
						2+ drink/d			1.07 (0.88-1.31)		
						Continuous (per 1 drink/d)			1.03 (0.97-1.08)	–	
						Women					
						<1 drink/d	–	–	1.00 (0.84-1.18)	0.776	
						1 drink/d			1.07 (0.87-1.30)		
						2+ drink/d			1.01 (0.81-1.25)		
						Continuous (per 1 drink/d)			0.99 (0.94-1.06)	–	
Ringel *et al*. (2023) (31)	Rare consumers (never or <1/wk)	Inc	Kidney	HR	Non-specific	**ASB Consumption**					Age, race and ethnicity, nSES, smoking, BMI, history of hypertension, and HEI
						Frequent (1-6/wk)	91	–	**1.34 (1.03-1.75)**	–	
						Daily (≥1/d)	48		1.14 (0.80-1.62)		

Notes:
AS = Artificial Sweetener; ASB = Artificially Sweetened Beverage; BMI = Body Mass Index; CI = Confidence Interval; d = Day; g = Gram; HEI = Healthy Eating Index; HPFS = Health Professionals Follow-up Study; HR = Hazard Ratio; Inc = Incidence; mo = Month; Mort = Mortality; NHS = Nurses Health Study; nSES = Neighborhood Socioeconomic Status; NSS = Non-Sugar Sweetener; RCC = Renal Cell Carcinoma; RR = Relative Risk; SSB = Sugar-Sweetened Beverage; wk = Week; yr = Year.
– = Not Reported.
Bolded values indicate statistical significance.
(a) Heath *et al*. (74) also evaluated RCC incidence and mortality risk by intake level categories of AS soft drinks as well as overall and sex-stratified risks after additional adjustment for fruit and vegetable intake, exclusion of the first 2 yrs of follow-up, or exclusion of those reporting diabetes at baseline. All results were similar to the main analysis and were not statistically significant.
(b) Hodge *et al*. (44) reported similar associations when excluding the first 2 yrs of follow-up and did not observe an association when evaluating linear trends on a log hazard scale.
(c) In analyses that additionally controlled for BMI, were conducted among never smokers only, or were stratified by BMI status (*i.e*., normal, overweight, or obese), all results were not statistically significant. Sensitivity analyses excluding the first 2 yrs of follow-up or with stratification by 10-yr follow-up time also had minimal impact (26).

Supplemental Table H.3 NSS and Bladder Cancer Case-Control Study Results

Citation	Outcome Type	Cancer Type	Statistical Analysis							Covariate Adjustment
			Risk Metric	NSS	Group	Exposed Cases	Exposed Controls	Risk Estimate (95% CI)	p_{Trend}	
Akdaş *et al*. (1990) (50)	Inc	Bladder	–[a]	Non-specific	AS non-users	175	186	Ref	–	–
					AS users	19	8	**<0.05**[a]		
Bruemmer *et al*. (1997) (51)	Inc	Bladder	OR	Non-specific	**Diet Soft Drink Intake**					Age, county, and smoking
					Men					
					≤1/mo	136	161	Ref	0.15	
					>1/mo-1/wk	14	15	1.0 (0.4-2.2)		
					>1/wk-7/wk	37	32	1.4 (0.8-2.5)		
					>7/wk	15	12	1.6 (0.7-3.6)		
					Women					
					≤1/mo	35	107	Ref	0.44	
					>1/mo-1/wk	6	22	1.5 (0.5-4.4)		
					>1/wk-7/wk	11	40	0.8 (0.3-1.8)		
					>7/wk	8	16	2.3 (0.8-6.3)		
Cartwright *et al*. (1981) (43)	Inc	Bladder	RR	Saccharin	**Regular[b] Saccharin Consumption as a Sugar Substitute**					Age, sex, smoking, and type of case (*i.e*., prevalent or incident)
					Male Non-smokers					
					Non-users	183	335	Ref	–	
					Users	33	27	**2.2 (1.3-3.8)**		
					Male Smokers					
					Non-users	344	346	Ref	–	
					Users	71	81	0.9 (0.6-1.3)		
					Female Non-smokers					
					Non-users	96	162	Ref	–	
					Users	16	19	1.6 (0.8-3.2)		
					Female Smokers					
					Non-users	81	76	Ref	–	
					Users	17	14	1.2 (0.5-2.6)		
Hoover and Strasser (1980) (42)[c]	Inc	Bladder	RR	Non-specific	**History of AS Use**					Race, cigarette smoking, coffee drinking, and occupational exposure
					Never used	1,707	3,321	Ref	–	
					Ever used diet drink	869	1,708	0.97 (0.87-1.07)		
					Ever used TT	828	1,540	1.04 (0.93-1.16)		
					Ever used diet food	370	681	1.05 (0.91-1.22)		
					Ever used any form	1,293	2,455	1.01 (0.92-1.11)		
					Males					
					Never used	1,349	2,554	Ref	–	
					Ever used diet drink	607	1,204	0.95 (0.84-1.07)		
					Ever used TT	592	1,066	1.04 (0.92-1.18)		
					Ever used diet food	240	442	1.02 (0.85-1.22)		
					Ever used any form	909	1,723	0.99 (0.89-1.10)		
					Females					
					Never used	358	767	Ref	–	
					Ever used diet drink	262	504	1.02 (0.83-1.25)		
					Ever used TT	236	474	1.04 (0.84-1.28)		
					Ever used diet food	130	239	1.13 (0.87-1.47)		
					Ever used any form	384	732	1.07 (0.89-1.29)		

Citation	Outcome Type	Cancer Type	Statistical Analysis							
			Risk Metric	NSS	Group	Exposed Cases	Exposed Controls	Risk Estimate (95% CI)	p_{Trend}	Covariate Adjustment
					**Average Daily Use of TT Sweeteners**					Age, race, and cigarette smoking
					Males					
					Never used AS	1,349	2,554	Ref	0.43	
					<1	109	190	1.09		
					1-1.9	105	229	0.88		
					2-3.9	164	299	1.08		
					4-5.9	62	118	0.97		
					≥6	39	59	1.05		
					Females					
					Never used AS	358	767	Ref	**0.03**	
					<1	39	113	0.73		
					1-1.9	56	96	1.28		
					2-3.9	72	110	1.42		
					4-5.9	22	45	0.99		
					≥6	16	20	1.36		
					**Average Daily Servings of Diet Drinks**					
					Males					
					Never used AS	1,349	2,554	Ref	0.36	
					<1	349	723	0.93		
					1-1.9	107	207	0.93		
					2-2.9	48	63	1.44		
					≥3	25	41	1.01		
					Females					
					Never used AS	358	767	Ref	0.17	
					<1	146	294	1.01		
					1-1.9	44	108	0.83		
					2-2.9	24	29	1.72		
					≥3	15	20	1.37		
					**Average Number of Daily Uses of TT by Average Number of Daily Diet Drinks**[d]					Age, sex, and race
					No Daily Diet Drinks					
					None	1,707	3,321	Ref	–	
					<3	189	367	1.02		
					3-5	80	136	1.15		
					≥6	18	34	0.99		
					<2 Daily Diet Drinks					
					None	314	638	0.94	–	
					<3	212	417	0.98		
					3-5	59	146	0.76		
					≥6	28	34	1.53		
					≥2 Daily Diet Drinks					
					None	38	60	1.21	–	
					<3	35	54	1.26		
					3-5	20	25	1.56		
					≥6	7	8	1.64		

Citation	Outcome Type	Cancer Type	Statistical Analysis							Covariate Adjustment
			Risk Metric	NSS	Group	Exposed Cases	Exposed Controls	Risk Estimate (95% CI)	p_{Trend}	
					**Use of AS Among Low-Risk[e] White Females**					Age
					Never Used AS	130	402	Ref	–	
					Ever used TT	82	210	1.2		
					Ever used diet drink	71	219	1.1		
					Average Daily Use of TT					
					<1	15	53	0.9	**<0.01**	
					1-1.9	17	43	1.2		
					2-2.9	21	36	1.8		
					≥3	22	38	1.8		
					≥2 TT by Duration (yrs)					
					5	14	34	1.3	**<0.01**	
					5-9	13	22	1.8		
					≥10	16	18	2.7		
					Average Daily Servings of Diet Drinks					
					<1	36	132	0.9	0.14	
					1-1.9	16	43	1.2		
					2-2.9	7	14	1.6		
					≥3	3	6	1.6		
					≥2 Diet Drinks by Duration (yrs)					
					5	1	6	0.5	**<0.05**	
					5-9	3	7	1.4		
					≥10	6	7	3.0		
					**Average Daily Consumption of AS Among White Males Who Smoked More than 40 Cigarettes Daily[f]**					
					Never used AS	104	167	Ref	–	
					Use of TT					
					<1	12	15	1.28	**0.01**	
					1-1.9	19	14	2.07		
					2-3.9	16	13	1.96		
					4-5.9	8	10	1.33		
					≥6	7	7	1.86		
					Servings of Diet Drinks					
					<1	39	53	1.20	**0.01**	
					1-1.9	14	19	1.20		
					2-2.9	10	5	3.33		
					≥3	6	4	2.62		

Citation	Outcome Type	Cancer Type	Statistical Analysis							Covariate Adjustment
			Risk Metric	NSS	Group	Exposed Cases	Exposed Controls	Risk Estimate (95% CI)	p_{Trend}	
Howe *et al*. (1977) (52)[g]	Inc	Bladder	OR	Non-specific	**Use of AS**					Age, sex, and neighborhood
					Males					
					Never	407	433	Ref	–	
					Ever	73	47	**1.6**		
					Females					
					Never	134	122	Ref	–	
					Ever	18	30	0.6		
					**Diet Food Consumption**					
					Males					
					Never	–	–	Ref	–	
					Ever			1.2		
					Females					
					Never	–	–	Ref	–	
					Ever			0.5		
				Saccharin	**Average Frequency of Use (Tablets/yr) Among Males**					
					Never	397	419	Ref	**0.018**	
					<2,500	42	29	1.5 (1.0- –)		
					≥2,500	16	7	2.1 (0.9- –)		
					**Total Duration of Use (yrs) Among Males**					
					Never	397	419	Ref	**0.031**	
					<3	30	22	1.4 (0.9- –)		
					≥3	28	14	**2.0 (1.2- –)**		
					**Frequency of Use (Tablets/yr) by Duration of Use (yrs) Among Males**					
					Never	407	433	Ref	–	
					<3 yrs					
					<2,500	25	17	1.6	–	
					≥2,500	6	5	1.3		
					≥3 yrs					
					<2,500	18	12	1.6	–	
					≥2,500	10	2	5.3		
Howe *et al*. (1980) (53)[g]	Inc	Bladder	RR	Non-specific	**Dietetic Drink Consumption**					Age, sex, and neighborhood
					Males					
					Never	–	–	Ref	–	
					Ever			0.8 (0.2-3.3)		
					Females					
					Never	–	–	Ref	–	
					Ever			0.9 (0.2-3.0)		
					**Average Frequency of AS Use (Tablets/d)**					Age, sex, neighborhood, smoking, occupation, use of non-public water supply, bladder infection, kidney infection, diabetes, education, aspirin use, and coffee consumption
					1-4	–	–	0.9	–	
					5-6			1.6		
					7-8			1.1		
					≥9			**2.8**		
				Saccharin	1-4			0.9		
					4-8			1.4		
					>8			**3.1**		
Iscovich *et al*. (1987) (54)	Inc	Bladder	–	Saccharin	Users of saccharin	–	–	No association	–	Age and smoking

Citation	Outcome Type	Cancer Type	Statistical Analysis							Covariate Adjustment
			Risk Metric	NSS	Group	Exposed Cases	Exposed Controls	Risk Estimate (95% CI)	p_{Trend}	
Kantor *et al*. (1985) (55)	Inc	Bladder	RR	Non-specific	**Use of AS (mg/wk) Among Subjects with Family History of UTC**					Age, sex, race, and family history of urinary tract cancer
					No family history of UTC and no AS use	–	–	Ref	–	
					Family history of UTC and no AS use	84	114	**1.5 (1.1-2.1)**		
					Family history of UTC and AS use (1-239)	22	35	1.5 (0.8-2.7)		
					Family history of UTC and AS use (240-719)	24	24	**2.0 (1.1-3.6)**		
					Family history of UTC and AS use (≥720)	18	15	2.1 (1.0-4.4)		
Kantor *et al*. (1988) (56)	Inc	SCC of bladder	RR	Non-specific	**Use of AS (mg/wk)**					Age and sex
					Nonuser	22	–	Ref	NS	
					1-239	1		0.2 (0.1-1.4)		
					240-719	8		1.9 (0.7-4.5)		
					720+	2		0.8 (0.2-3.6)		
		Adenocarcinoma of bladder			Nonuser	16		Ref		
					1-239	2		0.6 (0.1-2.7)		
					240-719	3		1.0 (0.2-3.7)		
					720+	3		1.6 (0.3-6.0)		
		TCC of bladder			Nonuser	1,552		Ref		Age, sex, and race
					1-239	277		0.9 (0.7-1.0)		
					240-719	271		1.0 (0.8-1.2)		
					720+	177		1.1 (0.9-1.3)		
Kessler and Clark (1978) (57)	Inc	Bladder	OR	Saccharin	**All Participants**					Age, sex, race, and current marital status
					Nonuser	–	–	Ref	–	
					Any use			1.06 (0.79-1.42)		
				Cyclamate	Any use			0.97 (0.71-1.32)		
				Saccharin and/or cyclamate	Any use			1.03 (0.77-1.38)		
				Saccharin and cyclamate	Any use			1.00 (0.73-1.37)		
				Saccharin	**Males**					
					Nonuser	–	–	Ref	–	
					Any use			1.13 (0.80-1.62)		
				Cyclamate	Any use			1.15 (0.78-1.70)		
				Saccharin and/or cyclamate	Any use			1.11 (0.78-1.58)		
				Saccharin and cyclamate	Any use			1.18 (0.80-1.76)		
				Saccharin	**Females**					
					Nonuser	–	–	Ref	–	
					Any use			0.82 (0.48-1.43)		
				Cyclamate	Any use			0.61 (0.34-1.14)		
				Saccharin and/or cyclamate	Any use			0.77 (0.44-1.33)		
				Saccharin and cyclamate	Any use			0.63 (0.35-1.12)		

Citation	Outcome Type	Cancer Type	Statistical Analysis							Covariate Adjustment
			Risk Metric	NSS	Group	Exposed Cases	Exposed Controls	Risk Estimate (95% CI)	p_{Trend}	
				Saccharin and cyclamate	**Source of NNS[h] Used More than Occasionally**					
					Nonuser[i]	–	–	Ref	–	
					Any TT			0.89 (0.67-1.18)		
					Any diet beverage			0.97 (0.72-1.31)		
					Any diet food			1.02 (0.72-1.45)		
					Total NNS			0.98 (0.75-1.28)		
					Men					
					Any TT	–	–	0.88 (0.62-1.25)	–	
					Any diet beverage			0.95 (0.65-1.38)		
					Any diet food			1.22 (0.79-1.90)		
					Total NNS			0.97 (0.70-1.35)		
					Women					
					Any TT	–	–	0.91 (0.55-1.50)	–	
					Any diet beverage			1.00 (0.61-1.63)		
					Any diet food			0.72 (0.39-1.32)		
					Total NNS			1.00 (0.63-1.59)		
					**Any Use of NNS Based on Multiple Logistic Regression**					Age, sex, race, occupation, smoking habit, diabetes, marital status, education, overweight, dieting, and memory
					Nonuser[i]	–	–	Ref	–	
					Men			1.11 (0.78-1.58)		
					Nonsmoking[j] men			**2.61 (1.20-5.67)**		
					Women			0.80 (0.47-1.39)		
					Both sexes			1.04 (0.80-1.40)		
					**Level of Any NNS[k] Exposure**					
					Men					
					Nonuser[i]	–	–	Ref	–	
					Low			1.09 (0.66-1.80)		
					Medium			1.39 (0.84-2.32)		
					High			0.97 (0.56-1.67)		
					Women					
					Nonuser[i]	–	–	Ref	–	
					Low			1.19 (0.59-2.42)		
					Medium			0.71 (0.29-1.77)		
					High			0.69 (0.32-1.48)		
Kobeissi *et al*. (2013) (58)	Inc	Bladder	–	Non-specific	**Frequency of intake**					None
					Never	40	71	χ^2 p = 0.074	–	
					Rarely	3	7			
					Frequently	1	7			
					Always	10	5			
Marrett *et al*. (1985) (59)	Inc	Bladder	OR	Non-specific	**AS Lifetime Uses (Including Foods, Beverages, and TT Sweeteners)**					Sex
					Males					
					<10	–	–	Ref	–	
					≥10			0.7		
					Females					
					<10	–	–	Ref	–	
					≥10			1.1		

Citation	Outcome Type	Cancer Type	Statistical Analysis							
			Risk Metric	NSS	Group	Exposed Cases	Exposed Controls	Risk Estimate (95% CI)	p_{Trend}	Covariate Adjustment
Moller-Jensen *et al*. (1983) (41)[l]	Inc	Urinary Bladder	OR	Saccharin and cyclamate	**Males and Females**					Age
					Never use	299	576	Ref	–	
					Ever use	81	200	0.78 (0.58-1.05)		
				Saccharin	**Males**					
					Never use	–	–	Ref	–	
					Ever use			0.68 (0.45-1.02)		
				Cyclamate	Never use			Ref		
					Ever use			0.72 (0.26-2.04)		
				Saccharin and cyclamate	Never used regularly[m]	229	433	Ref		
					Ever regular use, any	55	150	**0.69 (0.49-0.98)**		
					Ever regular use, TT	48	137	**0.66 (0.46-0.95)**		
					Ever Consumed AS					Age, smoking
					Never	221	427	Ref	–	
					Ever	55	147	0.65 (0.49-1.00)		
					Duration of Regular Use (yrs)					Age
					Never	229	433	Ref	–	
					0-4	18	41	0.83 (0.46-1.47)		
					5-9	8	27	0.57 (0.25-1.26)		
					10-14	11	33	0.90 (0.43-1.83)		
					≥15	10	34	**0.48 (0.23-0.98)**		
					Average Amount of TT Sweeteners Consumed – Ever Use (Tablets/day)					
					Never use	241	453	Ref	–	
					1-4	9	24	0.71 (0.32-1.54)		
					5-9	9	28	0.60 (0.28-1.29)		
					10-14	10	19	0.99 (0.45-2.16)		
					≥15	12	23	0.98 (0.48-2.01)		
				Saccharin	**Females**					
					Never Use	–	–	Ref	–	
					Ever Use			1.04 (0.51-2.09)		
				Cyclamate	Never Use			Ref		
					Ever Use			1.33 (0.22-8.13)		
				Saccharin or cyclamate	Never regular use[m]	70	143	Ref		
					Ever regular use, any	26	50	1.06 (0.61-1.85)		
					Ever regular use, TT	21	41	1.05 (0.57-1.91)		
					Ever Consumed AS					Age, smoking
					Never	69	143	Ref	–	
					Ever	26	50	1.03 (0.59-1.79)		
					Duration of Regular Use (yrs)					Age
					Never use	70	143	Ref	–	
					0-4	12	13	2.17 (0.93-5.07)		
					5-14	3	14	0.48 (0.13-1.72)		
					15+	5	10	0.81 (0.26-2.51)		

Citation	Outcome Type	Cancer Type	Statistical Analysis							Covariate Adjustment
			Risk Metric	NSS	Group	Exposed Cases	Exposed Controls	Risk Estimate (95% CI)	p_{Trend}	
					Average Amount of TT Sweeteners Consumed – Ever Use (Tablets/day)					
					Never use	77	154	Ref	–	
					1-4	4	3	2.67 (0.61-11.61)		
					5-9	2	9	0.44 (0.10-2.03)		
					10-14	7	5	2.80 (0.90-8.75)		
					15+	3	4	1.50 (0.33-6.83)		
Momas *et al*. (1994) (60)	Inc	Bladder	OR	Saccharin	**Saccharin Intake (Lifetime Tablets)**					Age, sex, lifelong tobacco smoking, lifelong coffee drinking, spice consumption, occupation, residence in non-Mediterranean area, infrequent consumption of carrots/spinach/marrows, lifelong alcohol-drinking, and birthplace in Mediterranean area
					<365	140	641	Ref	–	
					≥365	21	44	1.5 (0.8-3.0)		
Mommsen *et al*. (1983) (61)	Inc	Bladder	RR	Saccharin	Non-users	–	–	Ref	–	Age, sex, and geographic area
					All subjects	6	2	**6.7 (1.5-30.2)**		
						–	–	7.52 (–)		Age, sex, geographic area, cheroot smoker, cigarette smoker, use of estrogen, industrial work, coffee consumption, and work with chemical materials
					Never smokers			**3.3 (1.4-7.8)**		Age, sex, smoking, and geographic area
Morgan and Jain (1974) (62)	Inc	TCC of bladder	RR	Non-specific	**Prolonged Regular Use (*i.e.*, Period Probably Exceeding 1 yr) of Any AS**[n]					Age and sex
					Women					
					Non-user	–[o]	–[o]	Ref	–	
					User	–[o]	–[o]	**0.35**		
					Men					
					Non-user	–[o]	–[o]	Ref	–	
					User	–[o]	–[o]	1.00		
Najem *et al*. (1982) (63)	Inc	Bladder	RR	Non-specific	**Diet Beverage Consumption**					Age, sex, race, place of birth, census tract, and source of bladder cancer case, history of tobacco-related heart disease
					No	46	93	Ref	–	
					Yes	28	49	1.2 (0.6-2.1)		
				Saccharin	**Saccharin Consumption**					
					Never or occasional	62	123	Ref	–	
					Regular	12	19	1.3 (0.6-2.8)		
Ohno *et al*. (1985) (64)	Inc	Bladder	RR	Non-specific	**Use of AS**					Age, sex, smoking, and residence
					Males					
					Never	122	194	Ref	–	
					Ever	102	238	**0.67 (0.49-0.93)**		
					Females					
					Never	40	60	Ref	–	
					Ever	25	83	**0.45 (0.24-0.84)**		
Piper *et al*. (1986) (65)	Inc	Bladder	OR	Non-specific	**Use of ASBs and/or TT sweeteners**					Age group, sex, and area code
					Non-regular (<100 uses ever)	–	–	Ref	–	
					Regular (≥100 uses ever)	77	74	1.1 (0.7-1.7)		

Citation	Outcome Type	Cancer Type	Statistical Analysis							Covariate Adjustment
			Risk Metric	NSS	Group	Exposed Cases	Exposed Controls	Risk Estimate (95% CI)	p_{Trend}	
Risch *et al*. (1988) (66)	Inc	Bladder	OR	Saccharin or cyclamate[p]	**Regular TT AS Use**					Age group, sex, area of residence, lifetime cigarette consumption, and history of diabetes
					Males					
					Never	–	–	Ref	–	
					Ever			0.95 (0.72-1.25)		
					Ever (nonsmokers only)			1.00 (0.14-7.17)		
					Ever (use prior to 10 yrs before diagnosis only)			1.12 (0.75-1.67)		
					Females					
					Never	–	–	Ref	–	
					Ever			1.15 (0.75-1.76)		
					Ever (nonsmokers only)			1.04 (0.40-2.71)		
					Ever (use prior to 10 yrs before diagnosis only)			1.44 (0.76-2.71)		
				Saccharin	**TT Saccharin Use (per d)**					
					Males					
					Never	–	–	Ref	–	
					>0-1			1.04 (0.62-1.74)		
					>1-3			1.17 (0.72-1.91)		
					>3			0.81 (0.53-1.26)		
					Total lifetime intake (per 30 usage-yrs)			1.01 (0.86-1.18)		
					Females					
					Never	–	–	Ref	–	
					>0-1			1.46 (0.77-2.78)		
					>1-3			0.95 (0.42-2.15)		
					>3			1.27 (0.61-2.63)		
					Total lifetime intake (per 30 usage-yrs)			0.96 (0.79-1.16)		
				Cyclamate	**TT Cyclamate Use (per d)**					
					Males					
					Never	–	–	Ref	–	
					>0-1			0.54 (0.21-1.44)		
					>1			1.44 (0.55-3.77)		
					Total lifetime intake (per 20 usage-yrs)			1.09 (0.60-1.97)		
					Females					
					Never	–	–	Ref	–	
					>0-1			1.57 (0.53-4.66)		
					>1			1.21 (0.32-4.54)		
					Total lifetime intake (per 20 usage-yrs)			0.92 (0.63-1.36)		

Citation	Outcome Type	Cancer Type	Statistical Analysis							Covariate Adjustment
			Risk Metric	NSS	Group	Exposed Cases	Exposed Controls	Risk Estimate (95% CI)	p_{Trend}	
				Non-specific	**Use of Low-Calorie Foods (per d)**					
					Males					
					Never	–	–	Ref	–	
					>0-1			1.17 (0.76-1.79)		
					>1			0.75 (0.35-1.60)		
					Total lifetime intake (per 5 usage-yrs)			1.01 (0.82-1.24)		
					Ever regularly used prior to 10 yrs before diagnosis			1.11 (0.57-2.15)		
					Females					
					Never	–	–	Ref	–	
					>0-1			0.85 (0.49-1.48)		
					>1			2.18 (0.68-7.01)		
					Total lifetime intake (per 5 usage-yrs)			1.53 (1.00-2.34)		
					Ever regularly used prior to 10 yrs before diagnosis			0.88 (0.35-2.24)		
					**Use of Diet Soda (per wk)**					
					Males					
					Never	–	–	Ref	–	
					>0-4			**0.44 (0.21-0.89)**		
					>4			0.65 (0.34-1.23)		
					Total lifetime intake (per 10 usage-yrs)			1.00 (0.74-1.35)		
					Females					
					Never	–	–	Ref	–	
					>0-4			2.05 (0.88-4 77)		
					>4			1.94 (0.69-5.40)		
					Total lifetime intake (per 10 usage-yrs)			1.81 (0.83-3.94)		
Schulte *et al*. (1986) (67)	Inc	Bladder	–	Non-specific	Use of AS or diet soft drinks	–	–	No association[q]	–	–
Sturgeon *et al*. (1994) (68)	Inc	Bladder	RR	Non-specific	**AS Use (mg/d)**[r]					Age, sex, race, cigarette use, history of urinary infections and bladder stones, coffee consumption, family history of urinary tract cancer, high-risk occupation, and education
					Noninvasive Low Grade (I/II)					
					<1,680	668	–	Ref	–	
					≥1,680	19		1.1 (0.7-1.9)		
					Noninvasive High Grade (III/IV)					
					<1,680	146	–	Ref	–	
					≥1,680	9		**3.0 (1.5-6.3)**		
					Invasive Low Grade (I/II)					
					<1,680	188	–	Ref	–	
					≥1,680	4		0.8 (0.3-2.3)		
					Invasive High Grade (III/IV)					
					<1,680	293	–	Ref	–	
					≥1,680	13		1.7 (0.9-3.2)		

Citation	Outcome Type	Cancer Type	Statistical Analysis							Covariate Adjustment
			Risk Metric	NSS	Group	Exposed Cases	Exposed Controls	Risk Estimate (95% CI)	p_{Trend}	
Sullivan (1982) (69)	Inc	Bladder	–	Non-specific	**ASB Usage**[s]					–
					Age began use	–	–	***p* = 0.003**	–	
					Yrs of use			***p* = 0.03**		
					Glasses/wk			***p* = 0.02**		
Wang *et al*. (2013) (70)	Inc	Bladder	OR	Non-specific	**Diet Soft Drink Consumption (Servings/d)**					Age, sex, ethnicity, energy intake, and smoking
					Never	705	589	Ref	0.713	
					0.1-0.85	294	197	0.96 (0.77-1.19)		
					0.86+	300	221	1.06 (0.85-1.32)		
Wynder and Goldsmith (1977) (40)	Inc	Bladder	RR	Saccharin	**Duration of AS Use (yrs)**					Sex, race, hospital status and age at diagnosis
					Males					
					Nonuser	119	108	Ref	–	
					<5	8	9	0.8 (0.3-2.2)		
					5-14	4	6	0.6 (0.2-2.2)		
					≥15	1	1	0.9 (0.1-14.8)		
					Females					
					Nonuser	27	24	Ref	–	
					<5	2	3	0.6 (0.1-3.9)		
					5-14	1	1	0.9 (0.1-15.4)		
					≥15	1	1	0.9 (0.1-15.4)		
Wynder and Stellman (1980) (71)	Inc	Bladder	RR	Saccharin	**AS Use**					Age, sex, hospital, and hospital-room status[t]
					Males					
					Never	61	165	Ref	–	
					Ever	19	57	0.93 (0.68-1.28)		
					Females					
					Never	13	38	Ref	–	
					Ever	6	8	0.62 (0.26-1.40)		
					**Diet Beverage Use**					
					Males					
					Never	46	211	Ref	–	
					Ever	6	39	0.85 (0.55-1.17)		
					Females					
					Never	15	40	Ref	–	
					Ever	1	9	0.60 (0.27-1.29)		
Yu *et al*. (1997) (39)	Inc	Bladder	OR	Saccharin	**Use of Saccharin (Times/yr)**					Sex, age, area of residence, income, education, occupation, pack-yrs, taking analgesics, and history of urinary system disease
					None	84	211	Ref	**0.0007**	
					1-18	21	27	1.9 (0.9-4.1)		
					≥19	22	16	**3.9 (1.8-8.7)**		
					**Number of Yrs of Saccharin Use**					
					None	84	210	Ref	**0.0001**	
					1-14	14	32	1.4 (0.6-3.1)		
					≥15	29	12	**5.1 (2.3-11.6)**		

Notes:
AS = Artificial Sweetener; ASB = Artificially Sweetened Beverages; BMI = Body Mass Index; CI = Confidence Interval; d = Day; Inc = Incidence; mg = Milligram; Mo = Month; NE = Northeastern; NNS = Nonnutritive Sweetener; NS = Not Significant; NSS = Non-Sugar Sweetener; OR = Odds Ratio; RCC = Renal Cell Carcinoma; Ref = Reference; RR = Relative Risk; SCC = Squamous Cell Carcinoma; SE = Standard Error; TCC = Transitional Cell Carcinoma; TT = Tabletop; UTC = Urinary Tract Cancer; wk = Week; yr = Year.
– = Not Reported.
Bolded values indicate statistical significance.
(a) Akdaş *et al*. (50) did not report a risk estimate, only a *p* value calculated using a t test to compare exposure percentages in cases and controls.
(b) Cartwright *et al*. (43) defined "saccharin takers" as subjects regularly consuming saccharin for at least a yr, at least 5 yrs prior to the state of bladder cancer. "Regular consumption" was not defined.

(c) In results based on duration of exposure, lowest risks were seen in subjects with the longest use of AS. An erratic positive trend was observed for estimated lifetime consumption (daily use × duration) of TT among females. No consistent patterns emerged when considering yrs since first exposure (42).
(d) Hoover and Strasser (42) also reported an increased risk among those who used ≥3 servings of TT and ≥2 drinks daily or who used >0 diet drinks and ≥6 servings of TT (RR = 1.45, 95% CI: 1.00-2.10) after controlling for age, sex, race, smoking, occupational exposures, region, and education.
(e) Low risk subjects were defined as those who never smoked cigarettes, handled dye, rubber, leather, ink, or paint on any job (42).
(f) Hoover and Strasser (42) reported a non-significant decreasing trend with increased daily use of diet drinks among non-smoking males and a positive trend with diet drink consumption among heavy smoking females (>20 cigarettes daily). No consistent trends were observed for AS consumption among men and women who smoked less than 40 or 20 cigarettes daily (42).
(g) Howe *et al*. (52) and Howe *et al*. (53) used data from the same set of cases and controls. All statistical tests from these studies are one-sided.
(h) Kessler and Clark (57) provide additional results by diet beverage/food type (*e.g.*, diet cola, diet candy) and NSS source (*e.g.*, powder, tablets), however, an association was only observed for diet ice cream consumption among women (RR = 3.50, 95% CI: 1.15-10.63) and both sexes combined (RR = 1.68, 95% CI: 1.02-3.45).
(i) Kessler and Clark (57) did not explicitly state what the reference group was; this is assumed based on context.
(j) No associations were reported for male smokers or female participants stratified by smoking status (57).
(k) Kessler and Clark (57) reported no associations by source of NNS (*i.e.*, TT, carbonated diet sodas, any diet beverages, and any diet foods).
(l) Current use was also examined, and the results were null (41).
(m) Regular use for ≥3 mos (41).
(n) Morgan and Jain (62) also reported RR <1 in analyses considering each of the three categories of AS (*i.e.*, diet desserts, sugar-free soft drinks, and sugar substitutes). Consideration of smoking history did not change risk estimates.
(o) Numbers of cases and controls were difficult to interpret due to apparent formatting errors in Table III of the study (62).
(p) Risch *et al*. (66) reported results for non-specific TT AS, but in the methods specified "artificial sweeteners were classified by brand name and date of use as saccharin, cyclamate, or both"; therefore we assumed non-specific AS to be either saccharin or cyclamate.
(q) Schulte *et al*. (67) reported similar AS use by cases and controls overall and when combining "true" and atypical cases.
(r) Sturgeon *et al*. (68) reported no associations between AS use and stage or grade of tumor separately except for use of ≥1,680 mg/d and Grade III/IV transitional cell carcinoma of the bladder (OR = 2.2, 95% CI: 1.3-3.6).
(s) Sullivan (69) only reported *p* values and the mean for each category for cases and controls as reported in the table. However, they also reported that "the number of glasses of artificially sweetened beverage per week actually consumed by the patients who drank these products and the actual number of years of consumption of those who drank artificially sweetened beverages were not significantly different from those in the control group."
(t) Wynder and Stellman (71) state that the RR estimates did not vary appreciably when further controlled for history of diabetes, obesity, occupation, education, religion, and coffee or tea consumption. They also reported no association when analyses were restricted to current long-term (≥10 yrs) smokers of cigarettes.

Supplemental Table H.4 NSS and Kidney Cancer Case-Control Study Results

Citation	Outcome Type	Cancer Type	Statistical Analysis							
			Risk Metric	NSS	Group	Exposed Cases	Exposed Controls	Risk Estimate (95% CI)	P_{Trend}	Covariate Adjustment
Asal *et al*. (1988) (77)	Inc	RCC	OR	Saccharin and cyclamate	**Use of AS or Sugar Substitutes**					Age, smoking, and weight
					Males					
					Never	137	161	Ref	–	
					Ever	65	33	**2.1 (1.3-3.5)**		
					Females					–
					Never	65	101	Ref	–	
					Ever	40	40	1.3 (0.7-2.4)		
Gallus *et al*. (2007) (46)	Inc	RCC	OR	Non-specific	**All AS Consumption (Sachets or Tablets/d)[a]**					Age, sex, study center, education, tobacco smoking, alcohol drinking, BMI, total energy intake, and consumption of hot beverages
					0	687	1,368	Ref	0.632	
					>0-2	39	81	0.87 (0.58-1.30)		
					>2	41	85	0.96 (0.64-1.42)		
					Per sachet or tablet/d	–	–	0.99 (0.91-1.07)	–	
				Saccharin	**Saccharin Consumption (Sachets or Tablets/d)**					
					0	741	1,474	Ref	–	
					>0	26	60	0.79 (0.49-1.28)		
				Non-specific (mainly aspartame)	**AS (Other than Saccharin) Consumption (Sachets or Tablets/d)**					
					0	710	1,425	Ref	–	
					>0	57	109	1.03 (0.73-1.46)		
Goodman *et al*. (1986) (75)	Inc	RCC	OR	Saccharin	**Use of Saccharin Additives[b]**					Hospital, sex, race, age, and time of admission
					Never	193	205	Ref	–	
					Ever	73	61	1.29 (0.86-1.94)		
					Males					
					Never	142	148	Ref	–	
					Ever	47	41	1.21 (0.74-1.97)		
					Females					
					Never	51	57	Ref	–	
					Ever	26	20	1.50 (0.71-3.17)		
				Non-specific	**Use of Diet Beverages[b]**					
					Never	193	190	Ref	–	
					Ever	74	77	0.95 (0.65-1.38)		
					Males					
					Never	144	138	Ref	–	
					Ever	45	51	0.85 (0.53-1.34)		
					Females					
					Never	49	52	Ref	–	
					Ever	29	26	1.25 (0.65-2.39)		
					**Lifetime Use of AS (g)**					
					0	165	161	Ref	–	
					1-199	38	40	0.93 (0.56-1.53)		
					200+	49	51	0.94 (0.59-1.48)		
					Males					
					0	123	119	Ref	–	
					1-199	25	28	0.87 (0.47-1.58)		
					200+	31	32	0.95 (0.54-1.65)		

Citation	Outcome Type	Cancer Type	Statistical Analysis							Covariate Adjustment
			Risk Metric	NSS	Group	Exposed Cases	Exposed Controls	Risk Estimate (95% CI)	P_{Trend}	
					Females					
					0	42	42	Ref	–	
					1-199	13	12	1.08 (0.42-2.86)		
					200+	18	19	0.94 (0.39-2.20)		
Maclure and Willett (1990) (76)	Inc	Renal adenocarcinoma	OR	Non-specific	**Diet Soft Drink Intake Level**					Age, sex, income, education, high occupational status, history of cardiovascular disease, smoking, obesity, NE European ancestry, exposure to asbestos 30 yrs prior, phenacetin use, and history of hypertension or kidney stones
					Low (0-2 cup/mo)	–	–	Ref	**0.03**	
					Moderate (1-7 cups/wk)			1.2 (0. 70-2.0)		
					High (≥2 cups/d)			**2.7 (1.1-6.5)**		
					Low and Moderate Intake as Reference					
					Low and Moderate (<2 cups/d)	–	–	Ref	–	
					High (≥2 cups/d)			1.4 (0.68-2.9)		

Notes:
AS = Artificial Sweetener; ASB = Artificially Sweetened Beverages; BMI = Body Mass Index; CI = Confidence Interval; d = Day; g = Gram; Inc = Incidence; mg = Milligram; mo = Month; NE = Northeastern; NSS = Non-Sugar Sweetener; OR = Odds Ratio; RCC = Renal Cell Carcinoma; Ref = Reference; RR = Relative Risk; TT = Tabletop; wk = Week; yr = Year.
– = Not Reported
Bolded values indicate statistical significance.
(a) Gallus *et al*. (46) reported no association between continuous AS consumption (*i.e*., per sachet or tablet/d) and RCC or within any strata of BMI, sex, or age.
(b) "A user was considered a consumer of 30 mg of additive per day or 110 mg of diet beverages per week for a period of one year or more" (75).

Supplement I: Gastrointestinal System

Esophageal Cancer

Cohort Studies

Non-specific NSSs

In a study of men and women in the CPS-II cohort, McCullough et al. (26) reported no increased risk of esophageal cancer mortality with any level of ASB intake compared to those who reported never consuming ASBs (<1 drink/day HR = 1.01, 95% CI: 0.88-1.15; 1 drink/day HR = 0.93, 95% CI: 0.79-1.09; ≥2 drinks/day HR = 0.97, 95% CI: 0.83-1.15; p_{trend} = 0.554). Results stratified by sex were similar to the overall results. In analyses with additional BMI adjustments, analyses excluding the first 2 years of follow-up, and analyses stratified by 10-years of follow-up time, McCullough et al. (26) did not report any increased risk of esophageal cancer with ASBs.

Case-Control Studies

Non-specific NSSs

Risk estimates (ORs) of esophageal cancer and NSS ranged from 0.43 to 1.24, but most were below 1 and some were statistically significant (46, 78, 79). Mayne et al. (79) reported a decreased risk of incident esophageal adenocarcinoma (EAC) (OR = 0.52, 95% CI: 0.32-0.83) and esophageal squamous cell carcinoma (HR = 0.43, 95% CI: 0.23-0.82) when comparing the highest quartile of diet soft drink consumption with the lowest. Ibiebele et al. (78) also reported a decreased risk of esophageal SCC with any low calorie soft drink consumption (OR = 0.46, 95% CI: 0.25-0.85). Ibiebele et al. (78) reported no association between low calorie soft drink

consumption and EAC (OR = 0.71, 95% CI: 0.37-1.37) or esophagogastric junction adenocarcinoma (OR = 0.77, 95% CI: 0.46-1.29). Gallus et al. (46) observed no associations between any level of tabletop NSS consumption and esophageal cancer overall or stratified by BMI, sex, or age.

Saccharin

Gallus et al. (46) reported no association between any tabletop saccharin consumption and esophageal cancer (OR = 1.58, 95% CI: 0.59-4.25).

Stomach Cancer

Cohort Studies

Non-specific NSSs

Hodge et al. (44) reported no association between the frequency of NSS soft drink consumption and gastric cardia cancer risk among Australians in the MCCS cohort ($p_{trend} = 0.21$). McCullough et al. (26) reported no increased risk of stomach cancer mortality with any level of ASB intake compared to those who reported never consuming ASBs (<1 drink/day HR = 0.94, 95% CI: 0.82-1.07; 1 drink/day HR = 0.94, 95% CI: 0.80-1.11; ≥2 drinks/day HR = 1.10, 95% CI: 0.94-1.28; $p_{\text{trend}} = 0.580$). Results stratified by sex were similar to the overall results. No associations were reported in analyses with additional BMI adjustment, analyses excluding the first 2 years of follow-up, or in analyses stratified by BMI status, or 10-year follow-up time (26).

Case-Control Studies

Non-specific NSSs

Three studies investigated associations between non-specific NSS intake and incident stomach cancer. Two studies reported no association (47, 80). Bosetti et al. (80) reported no association between stomach cancer and use of any low-calorie sweeteners (OR = 0.80, 95% CI: 0.45-1.43), or low-calorie sweeteners excluding saccharin (OR = 0.86, 95% CI: 0.45-1.67) compared to non-users. Palomar-Cros et al. (47) compared medium (<3rd quartile) and high (≥3rd quartile) consumers of NSS other than aspartame to non-consumers and reported no association for either intake level (OR = 1.13, 95% CI: 0.87-1.47, and OR = 1.23, 95% CI: 0.84-1.77, respectively; $p_{\text{trends}} > 0.2$). Mayne et al. (79) reported a decreased risk of incident gastric cardia adenocarcinoma

(HR = 0.50, 95% CI: 0.31-0.81) and noncardia gastric adenocarcinoma (HR = 0.58, 95% CI: 0.38-0.90) when comparing the highest quartile of diet soft drink consumption with the lowest.

Aspartame

Only Palomar-Cros et al. (47) evaluated aspartame consumption and the risk of stomach cancer and reported no association with medium (<3rd quartile) or high (≥3rd quartile) intake (OR = 1.01, 95% CI: 0.69-1.46, and OR = 1.09, 95% CI: 0.62-1.83, respectively; $p_{\text{trend}} = 0.8$) when compared with non-consumers, and no dose-response trend.

Saccharin

Two studies analyzed saccharin consumption and risk of stomach cancer and neither reported an association (47, 80). Bosetti et al. (80) reported no increased risk of incident stomach cancer with any saccharin consumption compared to non-consumers (OR = 0.65, 95% CI: 0.25-1.68). Palomar-Cros et al. (47) did not report an association between medium or high saccharin consumption compared to non-consumers (OR = 0.93, 95% CI: 0.67-1.28; OR: 1.24, 95% CI: 0.83-1.80, respectively; $p_{\text{trend}} = 0.5$).

Colorectal Cancer

Cohort Studies

Non-specific NSSs

Risk estimates for colorectal cancer and non-specific NSS ranged from 0.65 to 1.22 across studies, with most estimates being close to 1 and not statistically significant (26, 27, 44, 81-83). Hodge et al. (44), Chazelas et al. (83), and Mullee et al. (27) reported no association overall between any level of ASB intake and colorectal cancer, and none reported significant trends ($p_{\text{trends}} \geq 0.21$).

McCullough et al. (26) reported no increased risk of colorectal cancer mortality with any level of ASB intake compared to those who reported never consuming ASBs (<1 drink/day HR = 0.99, 95% CI: 0.94-1.05; 1 drink/day HR = 1.03, 95% CI: 0.97-1.11; ≥2 drinks/day HR = 0.97, 95% CI: 0.90-1.04; $p_{\text{trend}} = 0.704$). Results stratified by sex, excluding the first 2 years of follow-up, or stratified by 10 years of follow-up time, were similar to the overall results.

Wang et al. (81) evaluated colorectal cancer risk stratified by sex in the HPFS (males) and NHS and NHS-II (females) cohorts, and reported no association with any quintile of cumulative average energy adjusted servings of ASBs in either sex (all risk estimates ranged from 1.01 to 1.15 and none were statistically significant). They also reported no significant trends (men $p_{\text{trend}} = 0.31$; women $p_{\text{trend}} = 0.78$). Hur et al. (82) evaluated risk of early onset colorectal cancer (EO-CRC) and ASB in the NHS-II cohort and reported no association with any level of ASB intake and no dose-response ($p_{trend} = 0.11$). In an analysis evaluating the effect of substituting SSB consumption with ASB consumption (*i.e.*, including SSB and ASB intake in the same model and calculating the RR

based on the difference between coefficients), the risk of incident EO-CRC decreased for each increase in ASB consumed per day (RR = 0.83, 95% CI: 0.69-0.99) which was similar to the effect of substitution of SSBs with water (RR = 0.89, 95% CI: 0.74-1.07).

Case-Control Studies

Non-specific NSSs

All seven case-control studies evaluated NSS mixtures and incident colorectal cancer risk, with most results close to 1 and some statistically significant risks both above and below 1 (46, 47, 84-88). Palomar-Cros et al. (47) reported no association between NSS consumption (excluding aspartame) in medium (OR = 1.01, 95% CI: 0.88-1.16) or high (OR = 0.99, 95% CI: 0.81-1.21) consumers when considering all participants combined (p_{trend} = 0.9) or in participants without diabetes (medium OR = 0.94, 95% CI: 0.81-1.1; high OR = 0.85, 95% CI: 0.66-1.1; p_{trend} = 0.2). In participants with diabetes, the authors reported an increased risk of colorectal cancer among medium and high consumers (OR = 1.54, 95% CI: 1.05-2.25, and OR = 1.58, 95% CI: 1.05-2.41, respectively, p_{trend} = 0.03). These associations were no longer significant in sensitivity analyses where additional confounders were considered (*e.g.*, dietary fiber intake, red meat intake, physical activity level). Mahfouz et al. (86) reported an increased risk of colorectal cancer among NSS consumers (OR = 20.8, 95% CI: 2.7-159.7) when compared with non-consumers, although it is not clear how many cases and controls this finding is based on; the wide CI indicates statistical instability.

All five other studies reported no associations. Chang et al. (85) reported no association between NSS intake and incident EO-CRC overall or in analyses stratified by sex. Similarly, Wu et al.

(88), reported no association between tabletop NSS use in tea or coffee and small intestinal adenocarcinoma overall in analyses stratified by sex. Franceschi et al. (84) reported no association with either colon (OR = 0.9, 95% CI: 0.7-1.1) or rectal cancer (OR = 0.8, 95% CI: 0.6-1.0) among participants who reported ever use of NSS. Gallus et al. (46) reported no association between colon or rectal cancer and any level of NSS sachet or tablet use, and no dose-response trend was observed ($p_{trend} = 0.338$). Theodoratou et al. (87) reported no association between colorectal cancer and low-calorie drink consumption (per quartile increase in consumption OR = 0.95, 95% CI: 0.88-1.10).

Aspartame

Palomar-Cros et al. (47) was the only study that evaluated aspartame intake and colorectal cancer. The authors reported a decreased risk of incident colorectal cancer among all participants, as well as among those without diabetes, who had medium aspartame intake compared to non-users (<3rd quartile; OR = 0.76, 95% CI: 0.62-0.93, and OR = 0.74, 95% CI: 0.59-0.93, respectively), but there were no associations reported with higher intake levels. They also reported no association with any level of intake among participants with diabetes.

Saccharin

Gallus et al. (46) reported no association between saccharin consumption and incident colon (OR = 0.95, 95% CI: 0.67-1.35) or rectal cancer (OR = 0.93, 95% CI: 0.60-1.45). Palomar-Cros et al. (47) reported no association between any level of saccharin intake and colorectal cancer risk, and

they reported a *negative* dose-response trend among participants without diabetes (p_{trend} = 0.02) but not among all participants or participants with diabetes.

Pancreatic Cancer

Cohort Studies

Non-specific NSSs

Risk estimates for pancreatic cancer and NSS mixtures ranged from 0.69 to 1.35 across studies, with most estimates being above 1 and a few reaching statistical significance (26, 28, 30, 89). Bao et al. (30) reported an increased risk of incident primary adenocarcinoma of the exocrine pancreas among participants in the fourth quintile of diet soft drink consumption (259-494.4 g/day) compared to those who never drank diet soft drinks (RR = 1.35, 95% CI: 1.03-1.77). Risk estimates reported for other quintiles ranged from 1.00 to 1.25, and none were statistically significant, including the highest quintile of consumption (RR = 1.25, 95% CI: 0.94-1.66); no dose-response trend was observed (p_{trend} = 0.19). Navarrete-Muñoz et al. (28) reported no increased risk of pancreatic cancer with any level of AS soft drink consumption overall, and no trend (p_{trend} = 0.81). There was no association reported overall or for males in linear analyses considering each increase of 100 g/day or 336 g/day (*i.e.*, one 12 oz serving). Among females, they reported an increased risk per 100 g/day increase (HR = 1.09, 95% CI: 1.03-1.15), but a decreased risk per 336 g/day increase (HR = 0.69, 95% CI: 0.44-1.09). Schernhammer et al. (89) reported no association between frequency of diet soft drink consumption and pancreatic cancer overall or stratified by sex, and there were no significant trends (p_{trends} > 0.5).

McCullough et al. (26) reported an increased risk of pancreatic cancer weakly associated with consuming 1 and ≥2 drinks/day among all participants (HR = 1.09, 95% CI: 1.01-1.18, and HR = 1.16, 95% CI: 1.07-1.26, respectively) and men (HR = 1.14, 95% CI: 1.01-1.29, and HR = 1.20,

95% CI: 1.06-1.37, respectively). They reported an increased risk in women who consumed ≥2 drinks/day (HR = 1.13, 95% CI: 1.02-1.25), but no association with lower levels of intake. Dose-response trends were reported for all participants combined, and men and women separately ($p_{\text{trends}} \leq 0.01$). They reported an association with each drink/day increase overall (HR = 1.04, 95% CI: 1.02-1.06), and for men (HR = 1.05, 95% CI: 1.02-1.09) and women (HR = 1.03, 95% CI: 1.00-1.06) separately. Similar findings were observed in analyses with additional BMI adjustment, in analyses of non-smokers with and without BMI controlled, in analyses excluding the first 2 years of follow-up, and in analyses stratified by 10-year follow-up time. No adjustments were made for diabetes, a key risk factor for pancreatic cancer.

Case-Control Studies

Non-specific NSSs

Five case-control studies examined NSS mixture consumption and pancreatic cancer risk, with risk estimates ranging from 0.47-1.8 across studies; estimates were both above and below 1 and few were statistically significant (80, 90-94, 94). Bosetti et al. (80) and Norell et al. (94) reported no increased risk of pancreatic cancer with any use of NSS. Bosetti et al. (80) reported no increased risk with any low-calorie sweetener (OR = 0.62, 95% CI: 0.37-1.04), or any non-saccharin low calorie sweetener (OR = 1.16, 95% CI: 0.66-2.04). Norell et al. (94) reported no increased risk of incident pancreatic cancer in analyses using either hospital (OR = 1.1, 95% CI: 0.6-2.0) or population-based controls (OR = 1.2, 95% CI: 0.7-2.0). Gold et al. (92) reported decreased risk of incident pancreatic cancer among diet soda consumers compared to hospital-based controls (OR = 0.47, 95% CI: 0.27-0.83), but no association with population-based controls (OR = 0.66, 95% CI: 0.38-1.2).

Davis et al. (91) and Chan et al. (90) evaluated pancreatic cancer risk by level of NSS intake. Davis et al. (91) reported no association between diet cola consumption and pancreatic cancer incidence or mortality among occasional (<1/day) or habitual (≥1/day) consumers. Chan et al. (90) reported an increased risk of incident adenocarcinoma of the exocrine pancreas among all participants and male consumers of ≥1 sugar-free carbonated beverage per day (OR = 1.5, 95% CI: 1.1-2.1, and OR = 1.8, 95% CI: 1.1-2.8, respectively) compared with non-consumers. A similar relationship was observed with low-calorie cola consumption (OR = 1.7, 95% CI: 1.2-2.4, and OR = 1.8, 95% CI: 1.1-2.9, respectively); however, no associations were reported for low-calorie caffeine-free cola or other low-calorie carbonated beverages. Chan et al. (90) did not report an increased risk among female consumers of any type of low calorie beverage and reported no statistically significant dose-response trends overall for either sex.

Saccharin

Bosetti et al. (80) reported a decreased risk of incident pancreatic cancer among saccharin users compared to non-users (OR = 0.19, 95% CI: 0.08-0.46). In contrast, Wynder et al. (93) did not find an association between saccharin use and incident pancreatic cancer when comparing users to non-users (OR not reported).

Supplemental Table I.1 NSS and Esophageal Cancer Cohort Study Results

Study	Reference Group	Outcome Type	Cancer Type	Statistical Analysis							Covariate Adjustment
				Risk Metric	NSS	Group	Exposed Cases	Expected Cases or Exposed Non-Cases	Risk Estimate (95% CI)	p_{Trend}	
McCullough *et al.* (2022) (26)	Never consume ASBs	Mort	Esophageal	HR	Non-specific	**ASB Consumption**[a]					Age, sex, race/ethnicity, smoking, marital status, education, consumption of red and processed meat, fruits and vegetables, alcohol, and SSBs
						<1 drink/d	–	–	1.01 (0.88-1.15)	0.554	
						1 drink/d			0.93 (0.79-1.09)		
						≥2 drink/d			0.97 (0.83-1.15)		
						Continuous (per 1 drink/d)			1.00 (0.96-1.05)	–	
						Men					
						<1 drink/d	–	–	0.97 (0.82-1.15)	0.946	
						1 drink/d			1.01 (0.83-1.22)		
						≥2 drink/d			1.01 (0.83-1.23)		
						Continuous (per 1 drink/d)			1.01 (0.96-1.06)	–	
						Women					
						<1 drink/d	–	–	1.11 (0.89-1.39)	0.326	
						1 drink/d			0.78 (0.58-1.07)		
						≥2 drink/d			0.91 (0.67-1.22)		
						Continuous (per 1 drink/d)			0.99 (0.91-1.07)	–	

Notes:
ASB = Artificially Sweetened Beverage; BMI = Body Mass Index; CI = Confidence Interval; d = Day; HR = Hazard Ratio; Mort = Mortality; NSS = Non-Sugar Sweetener; SSB = Sugar-Sweetened Beverage; yr = Year.
– = Not Reported.
(a) Risks were decreased among men who never smoked and consumed <1 drink/d (BMI-adjusted HR = 0.65, 95% CI: 0.42-0.98). There were no increased risks reported from analyses adjusted for BMI, analyses of never smokers with no control for BMI, or analyses stratified by BMI status (*i.e.*, normal, overweight, or obese). Sensitivity analyses excluding the first 2 yrs of follow-up or with stratification by 10-yr follow-up time produced similar results (26).

Supplemental Table I.2 NSS and Stomach Cancer Cohort Study Results

Study	Reference Group	Outcome Type	Cancer Type	Statistical Analysis							
				Risk Metric	NSS	Group	Exposed Cases	Expected Cases or Exposed Non-Cases	Risk Estimate (95% CI)	p_{Trend}	Covariate Adjustment
Hodge *et al.* (2018) (44)	Never or <1/mo	Inc	Gastric cardia	HR	Non-specific	**Frequency of AS Soft Drink Consumption**[a]					Age, sex, SEIFA, country of birth, alcohol intake, smoking status, physical activity, Mediterranean diet score, SS soft drink consumption, and waist circumference
						1-4/mo	–	–	1.08 (0.91-1.28)	0.21	
						>1-6/wk			1.02 (0.85-1.22)		
						≥1/d			1.22 (0.91-1.64)		
McCullough *et al*. (2022) (26)	Never consume ASBs	Mort	Stomach	HR	Non-specific	**ASB Consumption**[b]					Age, sex, race/ethnicity, smoking, marital status, education, consumption of red and processed meat, fruits and vegetables, alcohol, and SSBs
						<1 drink/d	–	–	0.94 (0.82-1.07)	0.580	
						1 drink/d			0.94 (0.80-1.11)		
						2+ drink/d			1.10 (0.94-1.28)		
						Continuous (per 1 drink/d)			1.01 (0.97-1.06)	–	
						Men					
						<1 drink/d	–	–	0.89 (0.73-1.08)	0.351	
						1 drink/d			0.99 (0.79-1.23)		
						2+ drink/d			1.16 (0.94-1.43)		
						Continuous (per 1 drink/d)			1.04 (0.98-1.10)	–	
						Women					
						<1 drink/d	–	–	0.97 (0.81-1.16)	0.957	
						1 drink/d			0.90 (0.71-1.14)		
						2+ drink/d			1.04 (0.83-1.32)		
						Continuous (per 1 drink/d)			0.98 (0.92-1.05)	–	

Notes:
AS = Artificial Sweetener; BMI = Body Mass Index; CI = Confidence Interval; d = Day; HR = Hazard Ratio; Inc = Incidence mo = Month; Mort = Mortality; NSS = Non-Sugar Sweetener; SEIFA = Socio-Economic Indexes for Areas; SS = Sugar Sweetened; SSB = Sugar-Sweetened Beverage, wk = Week; Yr = Year.
– = Not Reported.
(a) Hodge *et al*. (44) reported similar associations when excluding the first 2 yrs of follow-up. They did not observe an association when evaluating linear trends on a log hazard scale.
(b) There were no increased risks in analyses that controlled for BMI, that were conducted among never smokers only (with and without control for BMI), or that were stratified by BMI status (*i.e.*, normal, overweight, or obese). Sensitivity analyses excluding the first 2 yrs of follow-up or with stratification by 10-yr follow-up time produced similar results (26).

Supplemental Table I.3 NSS and Colorectal Cancer Cohort Study Results

Study	Reference Group	Outcome Type	Cancer Type	Statistical Analysis: Risk Metric	NSS	Group	Exposed Cases	Expected Cases or Exposed Non-Cases	Risk Estimate (95% CI)	p_{Trend}	Covariate Adjustment
Chazelas *et al.* (2019) (83)	Quartile 1[a]	Inc	Colorectal	sHR	Non-specific	**Sex-Specific Quartile[a] of ASB Intake[b]**					Age, sex, energy intake (without alcohol), sugar intake from non-sugary drink sources, alcohol, sodium, lipid, fruit, and vegetable intakes, BMI, height, physical activity, smoking status, number of 24-hr dietary records, family history of cancer, educational level, and at baseline: type 2 diabetes, hypertension, major cardiovascular event, and dyslipidemia
						Quartile 2	29	25,285	0.65 (0.37-1.14)	0.40	
						Quartile 3	4	25,315	0.84 (0.21-3.42)		
						Quartile 4	14	25,296	0.80 (0.44-1.46)		
						Per 10 mL/d	166	101,091	1.02 (0.94-1.10)	0.60	
Hodge *et al.* (2018) (44)	Never or <1/mo	Inc	Colorectal	HR	Non-specific	**Frequency of AS Soft Drink Consumption[c]**					Age, sex, SEIFA, country of birth, alcohol intake, smoking status, physical activity, Mediterranean diet score, SS soft drink consumption, and waist circumference
						1-3/mo	77	–	0.87 (0.68-1.11)	0.46	
						1-6/wk	125		1.15 (0.95-1.40)		
						≥1/d	51		0.79 (0.60-1.06)		
Hur *et al.* (2021) (82)	<1 serving/wk	Inc	EO-CRC	RR	Non-specific	**ASB Intake in Adulthood (Servings)[d]**					Age, energy/caloric intake, race, height, BMI, menopausal status and menopausal hormone use, family history of colorectal cancer, pack-years of smoking, physical activity, regular use of aspirin, regular use of NSAIDs, current use of multivitamins, intake of alcohol, red and process meat, dietary fiber, total folate and total calcium, AHEI-2010 score without sugar-sweetened beverages and alcohol, and lower endoscopy due to screening or for other indications within the past 10 yrs
						1/wk-<1/d	33	–	1.20 (0.73-1.98)	0.11	
						1/d-<2/d	19		0.86 (0.48-1.54)		
						≥2/d	25		0.73 (0.42-1.27)		
	–					Per 1/d increase	–		0.93 (0.83-1.04)	–	
						**Substitution of ASB for SSB[e]**					Age, energy/caloric intake, race, height, BMI, menopausal status and menopausal hormone use, family history of colorectal cancer, pack-years of smoking, physical activity, regular use of aspirin, regular use of NSAIDs, current use of multivitamins, intake of alcohol, red and process meat, dietary fiber, total folate and total calcium, AHEI-2010 score without sugar-sweetened beverages and alcohol, lower endoscopy due to screening or for other indications within the past 10 yrs, and SSB consumption
						Per 1/d increase	–	–	**0.83 (0.69-0.99)**	–	
McCullough *et al*. (2022) (26)	Never consume ASBs	Mort	Colorectal	HR	Non-specific	**ASB Consumption[f]**					Age, sex, race/ethnicity, smoking, marital status, education, consumption of red and processed meat, fruits and vegetables, alcohol, and SSBs
						<1 drink/d	–	–	0.99 (0.94-1.05)	0.704	
						1 drink/d			1.03 (0.97-1.11)		
						≥2 drink/d			0.97 (0.90-1.04)		
						Continuous (per 1 drink/d)			1.00 (0.98-1.02)	–	
						Men					
						<1 drink/d	–	–	1.05 (0.96-1.15)	0.680	
						1 drink/d			1.00 (0.90-1.12)		
						≥2 drink/d			1.01 (0.91-1.13)		
						Continuous (per 1 drink/d)			1.01 (0.98-1.04)	–	

Study	Reference Group	Outcome Type	Cancer Type	Statistical Analysis							Covariate Adjustment
				Risk Metric	NSS	Group	Exposed Cases	Expected Cases or Exposed Non-Cases	Risk Estimate (95% CI)	p_{Trend}	
						Women					
						<1 drink/d	–	–	0.97 (0.90-1.04)	0.431	
						1 drink/d			1.05 (0.97-1.14)		
						≥2 drink/d			0.94 (0.86-1.03)		
						Continuous (per 1 drink/d)			1.00 (0.97-1.02)	–	
Mullee *et al*. (2019) (27)	<1 glass/mo[g]	Mort	Colorectal	HR	Non-specific	**AS Soft Drink Consumption (Glasses)[g]**					Age, sex, EPIC center, BMI, physical activity, education, alcohol consumption, smoking status, intensity, and duration, ever use of contraceptive pill, menopausal status, ever use of menopausal hormone therapy, intakes of total energy, red and processed meat, fruits and vegetables, coffee, fruit and vegetable juice, and SS soft drinks
						1-4/mo	–	–	1.08 (0.91-1.28)	0.21	
						>1-6/wk			1.02 (0.85-1.22)		
						≥1/d			1.22 (0.91-1.64)		
Wang *et al*. (2022) (81)	Quintile 1	Inc	Colorectal	HR	Non-specific	**Cumulative Average Energy Adjusted Servings of ASBs by Quintile**					Age, calendar year of current questionnaire, race, family history of cancer, history of endoscopy, total alcohol intake, physical activity, smoking status and pack years of smoking, total caloric intake, regular aspirin use, and menopausal status and postmenopausal hormone use for women
						Men (HPFS)					
						2	–	–	1.06 (0.88-1.27)	0.31	
						3			1.15 (0.97-1.37)		
						4			1.11 (0.93-1.32)		
						5			1.14 (0.95-1.38)		
						Continuous			1.06 (0.95-1.19)	–	
						Women (NHS + NHS II)					
						2	–	–	1.06 (0.92-1.23)	0.78	
						3			1.08 (0.93-1.25)		
						4			1.01 (0.87-1.18)		
						5			1.07 (0.91-1.25)		
						Continuous			1.01 (0.95-1.07)	–	

Notes:
AHEI = Alternative Healthy Eating Index; AS = Artificial Sweetener; ASB = Artificially Sweetened Beverage; BMI = Body Mass Index; CI = Confidence Interval; d = Day; EPIC = European Prospective Investigation into Cancer and Nutrition; EO-CRC = Early-Onset Colorectal Cancer; HPFS = Health Professionals Follow-Up Study; hr = Hour; HR = Hazard Ratio; Inc = Incidence; mL = Milliliter; mo = Month; Mort = Mortality; NHS = Nurses' Health Study; NSAIDs = Non-Steroidal Anti-Inflammatory Drugs; NSS = Non-Sugar Sweetener; RR = Risk Ratio; SEIFA = Socio-Economic Indexes for Areas; sHR = Standardized Hazard Ratio; SS = Sugar-Sweetened; SSB = Sugar-Sweetened Beverage; wk = Week; yr = Year.
– = Not Reported.
Bolded values indicate statistical significance.
(a) ASB quartiles were 2.7, 4.7, and 7.9 mL/d in men and 4.6, 7.7, and 11.6 mL/d in women (83).
(b) Colorectal cancer risk was modeled accounting for competing risks of death and other cancers. Chazelas *et al*. (83) also evaluated risks stratified by sex, BMI at baseline, and % weight change, and in 22 sensitivity analyses adjusting for various potential confounders. Results from these analyses were similar to those in the main analysis and not statistically significant.
(c) Hodge *et al*. (44) reported similar associations when excluding the first 2 yrs of follow-up. They did not observe an association when evaluating linear trends on a log hazard scale.
(d) Hur *et al.* (82) also evaluated EO-CRC risk after adjustment for fruit juice consumption; results were similar to those in the primary analysis and not statistically significant.
(e) Hur *et al.* (82) described this analysis as "adding SSBs and the alternative beverage in the same multivariable model. RR was calculated using the difference in the two β-coefficients, with 95% CI using the corresponding variances and covariance."
(f) In analyses that controlled for BMI, risks among all subjects and women consuming 2+ drinks/d were decreased (men and women combined p_{trend} = 0.034). When stratified by BMI status (*i.e.*, normal, overweight, or obese), the risks associated with 2+ drinks/d among those with normal weight decreased (HR = 0.88, 95% CI: 0.77-0.99, p_{trend} = 0.027). Sensitivity analyses excluding the first 2 yrs of follow-up or with stratification by 10-yr follow-up time produced similar results (26).
(g) One glass = ~250 mL (27).

Supplemental Table I.4 NSS and Pancreatic Cancer Cohort Study Results

Study	Reference Group	Outcome Type	Cancer Type	Statistical Analysis							Covariate Adjustment
				**Risk Metric**	**NSS**	**Group**	**Exposed Cases**	**Expected Cases or Exposed Non-Cases**	**Risk Estimate (95% CI)**	p_{Trend}	
Bao *et al*. (2008) (30)	Never drinkers	Inc	Pancreatic (primary adenocarcinoma of exocrine pancreas)[a]	RR	Non-specific	**Quintile of Diet Soft Drink Consumption (g/d) with 2-yr Lag[b]**				0.19	Age, sex, race, education, BMI, alcohol, smoking, physical activity, energy-adjusted red meat consumption, energy adjusted folate consumption, total energy consumption, and regular soft drink consumption
						1 (0.1-26.3)	137	–	1.22 (0.94-1.59)		
						2 (26.4-76.9)	86		1.00 (0.74-1.35)		
						3 (77.0-258.9)	99		1.00 (0.75-1.34)		
						4 (259.0-494.4)	118		**1.35 (1.03-1.77)**		
						5 (494.5-4,897.6)	102		1.25 (0.94-1.66)		
McCullough *et al*. (2022) (26)	Never consume ASBs	Mort	Pancreatic	HR	Non-specific	**ASB Consumption[c]**					Age, sex, race/ethnicity, smoking, marital status, education, consumption of red and processed meat, fruits and vegetables, alcohol, and SSBs
						<1 drink/d	–	–	1.03 (0.97-1.11)	**<0.0001**	
						1 drink/d			**1.09 (1.01-1.18)**		
						2+ drink/d			**1.16 (1.07-1.26)**		
						Continuous (per 1 drink/d)			**1.04 (1.02-1.06)**	–	
						Men					
						<1 drink/d	–	–	0.99 (0.88-1.11)	**0.001**	
						1 drink/d			**1.14 (1.01-1.29)**		
						2+ drink/d			**1.20 (1.06-1.37)**		
						Continuous (per 1 drink/d)			**1.05 (1.02-1.09)**	–	
						Women					
						<1 drink/d	–	–	1.05 (0.97-1.14)	**0.010**	
						1 drink/d			1.06 (0.96-1.18)		
						2+ drink/d			**1.13 (1.02-1.25)**		
						Continuous (per 1 drink/d)			**1.03 (1.00-1.06)**	–	
Navarrete-Muñoz *et al*. (2016) (28)	Quintile 1 (0.1-2.0 g/d)	Inc	Pancreatic (adenocarcinoma of the exocrine pancreas)	HR	Non-specific	**AS Soft Drink Consumption (g/d)**					Age, sex, center, educational level, physical activity, smoking status, diabetes, energy intake, BMI, and consumption of alcohol, juice, and SS soft drinks
						Nondrinker	340	–	0.89 (0.58-1.36)	0.81	
						Quintile 2 (2.1-9.9)	50		1.12 (0.68-1.84)		
						Quintile 3 (9.9-28.6)	53		1.09 (0.69-1.73)		
						Quintile 4 (28.7-92.2)	42		0.99 (0.61-1.60)		
						Quintile 5 (>92.2)	47		0.99 (0.61-1.60)		
	Non-drinkers					**Continuous AS Soft Drink Consumption (per 100 g/d)[d]**					
						All subjects	–	–	1.02 (0.96- 1.08)[e]	–	
						Females	373		**1.09 (1.03-1.15)**		Age, sex, center, educational level, physical activity, smoking status, and consumption of alcohol, juice, and SS soft drinks
						Males	313		0.91 (0.80-1.04)		
						**Continuous AS Soft Drink Consumption (per 336 g/d; 12 oz)[d]**					
						All subjects	–	–	1.13 (0.94-1.36)	–	
						Females			0.69 (0.44-1.09)		
						Males			1.25 (1.03-1.52)		

				Statistical Analysis							
**Study**	**Reference Group**	**Outcome Type**	**Cancer Type**	**Risk Metric**	**NSS**	**Group**	**Exposed Cases**	**Expected Cases or Exposed Non-Cases**	**Risk Estimate (95% CI)**	**p_{Trend}**	**Covariate Adjustment**
Schernhammer *et al*. (2005) (89)	<1/mo	Inc	Pancreatic	RR[f]	Non-specific	**Frequency of Diet Soft Drink Consumption**					Age, sex, follow-up cycle, diabetes, smoking, caloric intake, physical activity, other soft drink consumption, and BMI
						NHS & HPFS (Women & Men)					
						1-12/mo	116	–	1.08 (0.85-1.38)	0.98	
						>3/wk	108		1.02 (0.79-1.32)		
						NHS (Women)					
						1-12/mo	62	–	1.10 (0.78-1.55)	0.64	
						>3/wk	66		1.12 (0.79-1.59)		
						HPFS (Men)					
						1-12/mo	54	–	1.08 (0.76-1.53)	0.52	
						>3/wk	42		0.89 (0.60-1.33)		

Notes:
AS = Artificial Sweetener; ASB = Artificially Sweetened Beverage; BMI = Body Mass Index; CI = Confidence Interval; d = Day; g = Gram; HPFS = Health Professionals Follow-up Study; HR = Hazard Ratio; Inc = Incidence; mo = Month; Mort = Mortality; NHS = Nurses' Health Study; NSS = Non-Sugar Sweetener; oz = Ounce; RR = Risk Ratio; SS = Sugar-Sweetened; SSB = Sugar-Sweetened Beverage; wk = Week; yr = Year.
– = Not Reported.
Bolded values indicate statistical significance.
(a) Bao *et al*. (30) excluded endocrine pancreatic tumors.
(b) Results from minimally adjusted (age and sex only) or unlagged analyses were similar to fully adjusted lagged results. There were no differences in risk when results were stratified by obesity, level of physical activity, or smoking status (30).
(c) In analyses that additionally controlled for BMI (among all subjects or either male or female never smokers), risks were increased for both 2+ drinks/d and per 1 drink/d, with increasing risk trends. In analyses with stratification by BMI status (*i.e*., normal, overweight, or obese), only HRs for overweight women based on 2+ drinks/d and per 1 drink/d (including increasing risk trends) were statistically significant. Sensitivity analyses excluding the first 2 yrs of follow-up or with stratification by 10-yr follow-up time produced similar results (26).
(d) Navarrete-Muñoz *et al*. (28) reported no effect modification by age at diagnosis, BMI, waist circumference, physical activity, or smoking status, but the association between pancreatic cancer and AS soft-drinks was modified by sex in both the 100 g/d and 336 g/d analyses (p_{trend} = 0.004).
(e) Navarrete-Muñoz *et al*. (28) reported no statistically significant increased risks of pancreatic cancer and AS soft drink consumption in country-specific analyses.
(f) RRs were reported for intake frequency of all diet soft drinks. Analyses stratified by consumption of diet colas or other diet soft drinks (non-diet colas), BMI, and physical activity reported similar non-statistically significant results (89).

Supplemental Table I.5 NSS and Esophageal Cancer Case-Control Study Results

Study	Outcome Type	Cancer Type	Statistical Analysis							Covariate Adjustment
			Risk Metric	NSS	Group	Exposed Cases	Exposed Controls	Risk Estimate (95% CI)	p_{Trend}	
Gallus *et al*. (2007) (46)	Inc	Esophageal	OR	Non-specific	**All AS Consumption (Sachets or Tablets/d)[a]**					Age, sex, study center, education, tobacco smoking, alcohol drinking, BMI, total energy intake, and consumption of hot beverages
					0	286	683	Ref	0.784	
					>0-2	6	31	0.78 (0.29-2.11)		
					>2	12	28	1.24 (0.54-2.81)		
					Per sachet or tablet/d	–	–	1.01 (0.85-1.21)	–	
				Saccharin	**Saccharin Consumption (Sachets or Tablets/d)**					
					0	296	724	Ref	–	
					>0	8	19	1.58 (0.59-4.25)		
				Non-specific (mainly aspartame)	**AS (Other than Saccharin) Consumption (Sachets or Tablets/d)**					
					0	294	702	Ref	–	
					>0	10	40	0.77 (0.34-1.75)		
Ibiebele *et al*. (2008) (78)	Inc	EAC	OR	Non-specific	**Consumption of Low-calorie Soft Drinks Only**					Age, sex, BMI, heartburn and acid reflux symptoms, cumulative history of smoking in pack yrs, alcohol intake status, education, total energy intake, and total vegetable intake
					Never	50	295	Ref	–	
					Ever	23	172	0.71 (0.37-1.37)		
		EGJAC			Never	72	295	Ref		
					Ever	35	172	0.77 (0.46-1.29)		
		Esophageal SCC			Never	74	295	Ref		
					Ever	22	172	**0.46 (0.25-0.85)**		
Mayne *et al*. (2006) (79)	Inc	EAC	OR	Non-specific	**Level of Diet Carbonated Soft Drink Consumption**					Age, sex, center, race, proxy interview status, average adult BMI, mean caloric intake, consumption of beer, wine, and liquor, consumption of meat, cigarettes per day, education, income, and frequency of reflux symptoms
					Low (quartile 1)	–	–	Ref	–	
					High (quartile 4)			**0.52 (0.32-0.83)**		
		Esophageal SCC			Low (quartile 1)			Ref		
					High (quartile 4)			**0.43 (0.23-0.82)**		

Notes:
AS = Artificial Sweetener; BMI = Body Mass Index; CI = Confidence Interval; d = Day; EAC = Esophageal Adenocarcinoma; EGJAC = Esophagogastric Junction Adenocarcinoma; GCA = Gastric Cardia Adenocarcinoma; Inc = Incidence; NCGA = Noncardia Gastric Adenocarcinoma; NSS = Non-Sugar Sweetener; OR = Odds Ratio; Ref = Reference; SCC = Squamous Cell Carcinoma; yr = Year.
– = Not Reported.
Bolded values indicate statistical significance.
(a) Gallus *et al*. (46) also conducted analyses for continuous AS consumption (*i.e.*, per sachet or tablet/d) and esophageal cancer stratified by BMI, sex, and age. No associations were observed.

Supplemental Table I.6 NSS and Stomach Cancer Case-Control Study Results

Study	Outcome Type	Cancer Type	Statistical Analysis							
			Risk Metric	NSS	Group	Exposed Cases	Exposed Controls	Risk Estimate (95% CI)	p_{Trend}	Covariate Adjustment
Bosetti *et al.* (2009) (80)	Inc	Stomach	OR	Non-specific	**Consumption of Any Low-Calorie Sweeteners**					Age, sex, study center, yr of interview, education, BMI, tobacco smoking, history of diabetes, consumption of hot beverages, and total energy intake
					Nonusers	207	471	Ref	–	
					Users	23	71	0.80 (0.45-1.43)		
					**Consumption of Any Low-Calorie Sweeteners (Excluding Saccharin)[a]**					
					Nonusers	213	491	Ref		
					Users	17	51	0.86 (0.45-1.67)		
				Saccharin	**Consumption of Saccharin**					
					Nonusers	224	521	Ref		
					Users	6	23	0.65 (0.25-1.68)		
Mayne *et al*. (2006) (79)	Inc	GCA	OR	Non-specific	**Level of Diet Carbonated Soft Drink Consumption**					Age, sex, center, race, proxy interview status, average adult BMI, mean caloric intake, consumption of beer, wine, and liquor, consumption of meat, cigarettes per day, education, income, and frequency of reflux symptoms
					Low (quartile 1)	–	–	Ref	–	
					High (quartile 4)			**0.50 (0.31-0.81)**		
		NCGA			Low (quartile 1)			Ref		
					High (quartile 4)			**0.58 (0.38-0.90)**		
Palomar-Cros *et al.* (2023) (47)	Inc	Stomach	OR	Aspartame[b]	**Level of Intake[c]**					Age, sex, study center, education, smoking, radiation exposure, total WCRF score continuous, total energy intake, total sugar intake, family history of colorectal cancer, night shift work, and aspartame or other AS consumption[e]
					All Participants[d]					
					Non-consumers	290	2,435	Ref	0.8	
					Medium	42	430	1.01 (0.69-1.46)		
					High	19	165	1.09 (0.62-1.83)		
				Other AS (excluding aspartame)[b]	Non-consumers	188	1,755	Ref	0.2	
					Medium	117	945	1.13 (0.87-1.47)		
					High	46	330	1.23 (0.84-1.77)		
				Saccharin	Non-consumers	255	2,166	Ref	0.5	Age, sex, study center, education, smoking, radiation exposure, total WCRF score continuous, total energy intake, total sugar intake, and other sources of sweeteners
					Medium	56	565	0.93 (0.67-1.28)		
					High	40	299	1.24 (0.83-1.80)		

Notes:
AS = Artificial Sweetener; BMI = Body Mass Index; CI = Confidence Interval; EO-CRC = Early-Onset Colorectal Cancer; GCA = Gastric Cardia Adenocarcinoma; Inc = Incidence; NSAID = Non-Steroidal Anti-Inflammatory Drugs; NCGA = Noncardia Gastric Adenocarcinoma; NSS = Non-Sugar Sweetener; OR = Odds Ratio; Ref = Reference; WCRF = World Cancer Research Fund; Wk = Week; Yr = Year.
– = Not Reported.
Bolded values indicate statistical significance.
(a) Bosetti *et al.* (80) reported that any low-calorie sweeteners (excluding saccharin) were "mostly aspartame."
(b) Palomar-Cros *et al*. (47) used "public sources of nutritional information (https://es.openfoodfacts.org/) to determine the most common type of sweetener in each of these food items." They combined non-saccharin TT use, which they reported to be primarily aspartame, and low or no calorie soft drinks into the aspartame category. For the "Other AS" intake category the authors combined TT saccharin and gaseosa, which is an ASB beverage in Spain that reported is usually sweetened with saccharin and cyclamate.
(c) Sex-specific quartiles among consumers and controls were used to compare moderate (<3rd quartile) and high (≥3rd quartile) consumers to non-consumers (reference) (47).
(d) No associations with stomach cancer were reported when analyses were stratified by diabetes status (though a positive trend was observed among participants with diabetes for both aspartame and other AS) or when considering consumption of low- or no-calorie soft drinks (though a negative trend was observed among participants with diabetes) or saccharin only (47).
(e) Sensitivity analyses adjusting for individual confounders (*i.e*., BMI, dietary fiber, red meat, physical activity, and alcohol) instead of the WCRF score with and without BMI adjustment produced similar results. When additionally adjusting for weight change from the prior year, participants with diabetes and a medium intake of aspartame had an increased risk of stomach cancer (OR = 2.81, 95% CI: 1.12-7.01) with a negative exposure-response trend (p_{trend} = 0.05); all other results were similar to the main analysis results (47).

Supplemental Table I.7 NSS and Colorectal Cancer Case-Control Study Results

Study	Outcome Type	Cancer Type	Statistical Analysis							Covariate Adjustment
			Risk Metric	NSS	Group	Exposed Cases	Exposed Controls	Risk Estimate (95% CI)	p_{Trend}	
Chang *et al*. (2021) (85)	Inc	EO-CRC	OR	Non-specific	**Intake of AS (Times/wk)**[a]					Age, sex, family history of colorectal cancer, regular aspirin/NSAID use, smoking, physical activity, BMI, alcohol consumption, red/processed meat intake, total fruit and vegetable intake, high-fiber food intake, and calcium supplement use
					<1	116	173	Ref	–	
					1-6	31	53	1.19 (0.68-2.08)		
					≥7	28	27	1.66 (0.89-3.13)		
Franceschi *et al*. (1997) (84)	Inc	Colon	OR	Non-specific	**Use of AS**					Age, sex, study center, education, physical activity, and total energy intake
					Never	–	–	Ref	–	
					Ever			0.9 (0.7-1.1)		
		Rectal			Never			Ref		
					Ever			0.8 (0.6-1.0)		
Gallus *et al*. (2007) (46)	Inc	Colon	OR	Non-specific	**AS Consumption (Sachets or Tablets/d)**[b]					Age, sex, study center, education, tobacco smoking, alcohol drinking, BMI, total energy intake, and consumption of hot beverages
					0	1,096	3,683	Ref	0.338	
					>0-2	70	245	0.91 (0.68-1.21)		
					>2	59	226	0.89 (0.65-1.21)		
					Per sachet or tablet/d	–	–	0.96 (0.90-1.02)	–	
				Saccharin	**Saccharin Consumption (Sachets or Tablets/d)**					
					0	1,181	4,004	Ref	–	
					>0	44	150	0.95 (0.67-1.35)		
				Non-specific (mainly aspartame)	**AS (Other than Saccharin) Consumption (Sachets or Tablets/d)**					
					0	1,137	3,827	Ref	–	
					>0	88	327	0.90 (0.70-1.16)		
		Rectal		Non-specific	**AS Consumption (Sachets or Tablets/d)**[b]					
					0	664	3,683	Ref	0.127	
					>0-2	32	245	0.77 (0.52-1.13)		
					>2	32	226	0.80 (0.54-1.19)		
					Per sachet or tablet/d	–	–	0.94 (0.86-1.02)	–	
				Saccharin	**Saccharin Consumption (Sachets or Tablets/d)**					
					0	703	4,004	Ref	–	
					>0	25	150	0.93 (0.60-1.45)		
				Non-specific (mainly aspartame)	**AS (Other than Saccharin) Consumption (Sachets or Tablets/d)**					
					0	689	3,827	Ref	–	
					>0	39	327	0.71 (0.50-1.02)		
Mahfouz *et al*. (2014) (86)	Inc	Colorectal	OR	Non-specific	**Use of AS**					Family history of colorectal cancer[c]
					None	–	–	Ref	–	
					Any			**20.8 (2.7-159.7)**		

Study	Outcome Type	Cancer Type	Statistical Analysis							
			Risk Metric	NSS	Group	Exposed Cases	Exposed Controls	Risk Estimate (95% CI)	p_{Trend}	Covariate Adjustment
Palomar-Cros *et al.* (2023) (47)	Inc	Colorectal	OR	Aspartame[d]	**Level of Intake[e]**					Age, sex, study center, education, smoking, radiation exposure, total WCRF score continuous, total energy intake, total sugar intake, family history of colorectal cancer, night shift work, and aspartame or other AS consumption[f]
					All Participants					
					Non-consumers	1,620	2,796	Ref	0.1	
					Medium	172	508	**0.76 (0.62-0.93)**		
					High	89	194	0.94 (0.71-1.25)		
					Participants Without Diabetes					
					Non-consumers	1,358	2,438	Ref	**0.04**	
					Medium	127	422	**0.74 (0.59-0.93)**		
					High	57	147	0.87 (0.61-1.21)		
					Participants with Diabetes					
					Non-consumers	262	358	Ref	1.0	
					Medium	45	86	0.82 (0.52-1.27)		
					High	32	47	1.09 (0.63-1.87)		
				Other AS (excluding aspartame)[d,g]	*All Participants*					
					Non-consumers	1,070	2,047	Ref	0.9	
					Medium	586	1,062	1.01 (0.88-1.16)		
					High	225	389	0.99 (0.81-1.21)		
					Participants Without Diabetes					
					Non-consumers	991	1,889	Ref	0.2	
					Medium	430	862	0.94 (0.81-1.1)		
					High	121	256	0.85 (0.66-1.1)		
					Participants with Diabetes					
					Non-consumers	79	158	Ref	**0.03**	
					Medium	156	200	**1.54 (1.05-2.25)**		
					High	104	133	**1.58 (1.05–2.41)**		
				Saccharin	*All Participants*					Age, sex, study center, education, smoking, radiation exposure, total WCRF score continuous, total energy intake, total sugar intake, and other sources of sweeteners
					Non-consumers	1,365	2,512	Ref	0.3	
					Medium	329	631	0.96 (0.81-1.12)		
					High	187	355	0.91 (0.74-1.12)		
					Participants Without Diabetes					
					Non-consumers	1,262	2,335	Ref	**0.02**	
					Medium	192	448	0.87 (0.71-1.05)		
					High	88	224	0.75 (0.57-1.00)		
					Participants with Diabetes					
					Non-consumers	103	177	Ref	0.2	
					Medium	137	137	1.29 (0.90-1.86)		
					High	99	131	1.32 (0.89-1.97)		
Theodoratou *et al.* (2014) (87)	Inc	Colorectal	OR	Non-specific	Each quartile of consumption[h]	–	–	0.95 (0.88-1.10)[i]	–	–[j]

Study	Outcome Type	Cancer Type	Statistical Analysis						
			Risk Metric	NSS	Group	Exposed Cases	Exposed Controls	Risk Estimate (95% CI)	p_{Trend}	Covariate Adjustment
Wu *et al*. (1997) (88)	Inc	Small intestinal adenocarcinoma	OR	Non-specific	**Adding AS to Tea and/or Coffee**					Age, sex, and ethnicity
					Never	28	780	Ref	–	
					Ever (tea or coffee)	4	152	0.7 (0.2-2.0)		
					Ever (tea and coffee)	4	66	1.5 (0.5-4.4)		
					Males					
					Never	16	556	Ref	–	
					Ever (tea or coffee)	1	89	0.4 (–)		
					Ever (tea and coffee)	1	42	0.8 (–)		
					Females					
					Never	12	224	Ref	–	
					Ever (tea or coffee)	3	63	0.9 (–)		
					Ever (tea and coffee)	3	24	2.2 (–)		

Notes:
AS = Artificial Sweetener; BMI = Body Mass Index; CI = Confidence Interval; d = Day; EO-CRC = Early-Onset Colorectal Cancer; Inc = Incidence; NSAID = Non-Steroidal Anti-Inflammatory Drugs; NSS = Non-Sugar Sweetener; OR = Odds Ratio; Ref = Reference; SSB = Sugar-Sweetened Beverage; WCRF = World Cancer Research Fund; wk = Week; yr = Year.
– = Not Reported.
Bolded values indicate statistical significance.
(a) Chang *et al*. (85) did not observe any associations in analyses stratified by sex or anatomical subsite (proximal colon or distal colon/rectum).
(b) Gallus *et al*. (46) also conducted analyses for continuous AS consumption (*i.e*., per sachet or tablet/d) and colon and rectal cancer with stratifications by BMI, sex, and age. No associations were observed.
(c) It is not clear what covariates were controlled for in the multiple regression analysis (86)
(d) Palomar-Cros *et al*. (47) used "public sources of nutritional information (https://es.openfoodfacts.org/) to determine the most common type of sweetener in each of these food items." They combined non-saccharin TT use, which they reported to be primarily aspartame, and low- or no- calorie soft drinks into the aspartame category. For the "Other AS" intake category the authors combined TT saccharin and gaseosa, which is an ASB beverage in Spain that reported is usually sweetened with saccharin and cyclamate.
(e) Sex-specific quartiles among consumers and controls were used to compare moderate (<3rd quartile) and high (≥3rd quartile) consumers to non-consumers (reference) (47).
(f) There was no longer an association between high intake of other AS and colorectal cancer among participants with diabetes in sensitivity analyses adjusted for individual confounders (*i.e*., BMI, dietary fiber, red meat, physical activity, and alcohol) instead of the WCRF score with and without adjustment for BMI, and additionally adjusting for weight change. All other results were similar, including when adjusting for dairy products (47).
(g) In sensitivity analyses, when considering only the consumption of low- or no-calorie soft drinks, lower risks of colorectal cancer from medium intake were observed among participants without diabetes (OR = 0.75, 95% CI: 0.58-0.96) (47).
(h) Statistical analyses were conducted using forward and reverse stepwise regression, resulting in ORs for variables without a unique list of covariates. The variable "low calorie drinks" was selected in 166 models using forward stepwise regression and in 153 models using backward stepwise regression. Low-calorie drink intake was evaluated based on quartile of consumption, but quartiles were not defined by Theodoratou *et al*. (87).
(i) Mean, minimum, and maximum values of the effect estimates for low-calorie drinks after forward or backward stepwise regression (*i.e*., values are the same for both regressions) (87).
(j) Theodoratou *et al*. (87) considered the following covariates: family history, deprivation, sex, age, NSAIDs, alcohol, physical activity, BMI, smoking, food variables (*e.g*., juice, high energy snacks, SSBs), and nutrient variables (*e.g*., energy, fiber, magnesium). The risk estimate for low-calorie drinks is the bootstrapped OR after the data were run 1,000 times. The selected covariates for each bootstrap are likely to be different.

Supplemental Table I.8 NSS and Pancreatic Cancer Case-Control Study Results

Study	Outcome Type	Cancer Type	Statistical Analysis							
			Risk Metric	NSS	Group	Exposed Cases	Exposed Controls	Risk Estimate (95% CI)	p_{Trend}	Covariate Adjustment
Bosetti *et al*. (2009) (80)	Inc	Pancreatic	OR	Non-specific	**Consumption of Any Low-Calorie Sweeteners**					Age, sex, study center, yr of interview, education, BMI, tobacco smoking, history of diabetes, consumption of hot beverages, and total energy intake
					Nonusers	281	571	Ref	–	
					Users	45	80	0.62 (0.37-1.04)		
					**Consumption of Any Low-Calorie Sweeteners (Excluding Saccharin)**[a]					
					Nonusers	291	602	Ref	–	
					Users	35	49	1.16 (0.66-2.04)		
				Saccharin	**Consumption of Saccharin**					
					Nonusers	316	618	Ref	–	
					Users	10	34	**0.19 (0.08-0.46)**		
Chan *et al*. (2009) (90)[b]	Inc	Adenocarcinoma of exocrine pancreas	OR	Non-specific	**Consumption of Sweetened Beverages**					Age, sex, energy intake, BMI, race, education, smoking, diabetes, physical activity, and diet (*i.e.*, red meat, white meat, vegetable and fruit, eggs, fish, dairy, whole grain, refined grain, sweets and other categories of sweetened beverages)
					Total Sugar-Free Carbonated Beverages (Drinks/d)					
					Men and Women Combined					
					0	310	1,016	Ref	0.2	
					<1	124	487	0.8 (0.7-1.1)		
					≥1	92	198	**1.5 (1.1-2.1)**		
					Men					
					0	–	–	Ref	0.4	
					<1			1.2 (0.8-1.7)		
					≥1			**1.8 (1.1-2.8)**		
					Women					
					0	–	–	Ref	0.3	
					<1			0.6 (0.4-1.0)		
					≥1			1.4 (0.9-2.3)		
					Low-Calorie Cola Beverages					
					Men and Women Combined					
					<1/mo	381	1,272	Ref	0.06	
					1-3/mo	34	122	1.0 (0.7-1.5)		
					1-6/wk	54	197	0.9 (0.7-1.3)		
					≥ 1/d	57	110	**1.7 (1.2-2.4)**		
					Men					
					<1/mo	–	–	Ref	0.06	
					1-3/mo			1.0 (0.6-1.8)		
					1-6/wk			1.1 (0.7-1.7)		
					≥1/d			**1.8 (1.1-2.9)**		
					Women					
					<1/mo	–	–	Ref	0.4	
					1-3/mo			1.0 (0.5-1.9)		
					1-6/wk			0.8 (0.4, 1.4)		
					≥1/d			1.6 (0.9-2.8)		

Study	Outcome Type	Cancer Type	Statistical Analysis							
			Risk Metric	NSS	Group	Exposed Cases	Exposed Controls	Risk Estimate (95% CI)	p_{Trend}	Covariate Adjustment
Davis *et al*. (2023) (91)	Inc	Pancreatic	OR	Non-specific	**Diet Cola Consumption**[c]					Age, sex, survey year, smoking status, BMI, total vegetable servings per week, processed meat servings per week, family history of pancreatic cancer, and non-diet colas and soft drinks
					Never	144	561	Ref	–	
					Occasional (<1/d)	36	151	0.96 (0.62-1.49)		
					Habitual (≥1/ d)	33	140	0.92 (0.59-1.43)		
	Mort		HR		**Mortality <1 yr Since Diagnosis by Diet Cola Consumption**					Age, sex, survey year, smoking status, BMI, histology, stage at diagnosis, surgery status, radiation status, total vegetable servings per week, and non-diet colas and soft drinks
					Never	137	7	Ref	–	
					Occasional (<1/d)	35	1	1.14 (0.75-1.73)		
					Habitual (≥1/ d)	31	2	1.05 (0.70-1.59)		
Gold *et al*. (1985) (92)	Inc	Pancreatic	OR	Non-specific	**Consumption of Diet Soda**					Age, sex, race, religion, occupation, smoking, alcohol consumption, and hospitals and date of admission (hospital controls) or telephone exchange (population controls)
					Never	–	–	Ref	–	
					Ever (compared to hospital controls)			**0.47 (0.27-0.83)**		
					Ever (compared to population controls)			0.66 (0.38-1.2)		
Norell *et al*. (1986) (94)	Inc	Pancreatic	RR	Non-specific	**Use of AS**					Age, sex, and parish/ surgical departments
					Hospital Controls					
					Nonusers	78	137	Ref	–	
					Users	18	26	1.1 (0.6-2.0)[d]		
					Population Controls					
					Nonusers	78	113	Ref	–	
					Users	18	25	1.2 (0.7-2.0)[d]		
Wynder *et al*. (1986) (93)	Inc	Pancreatic	OR	Saccharin	Nonusers	–	–	Ref	–	–
					Users			Not significant[e]		

Notes:
AS = Artificial Sweetener; BMI = Body Mass Index; CI = Confidence Interval; d = Day; HR = Hazard Ratio; Inc = Incidence; mo = Month; Mort = Mortality; OR = Odds Ratio; Ref = Reference; RR = Relative Risk; wk = Week; yr = Year.
– = Not Reported.
Bolded values indicate statistical significance.
(a) Bosetti *et al.* (80) reported that any low-calorie sweeteners (excluding saccharin) were "mostly aspartame."
(b) Chan *et al*. (90) additionally presented analyses by low-calorie caffeine-free cola and other low-calorie carbonated beverage consumption. No associations or trends were observed for either analysis.
(c) No pancreatic cancer risks by diet cola consumption stratified by smoking status, BMI, and sex were statistically significant except for an increased risk among habitual consumers (1+/d) who are current smokers (RR = 3.34, 95% CI: 1.12-9.98) (91).
(d) Norell *et al*. (94) report 90% CIs.
(e) Wynder *et al*. (93) did not report a risk estimate.

Supplement J: Lymphohematopoietic System

Leukemia

Cohort Studies

Non-specific NSSs

Two cohort studies evaluated leukemia and NSSs mixtures. McCullough et al. (26) reported no association between intake of <1, 1, or ≥2 ASBs per day and leukemia; HRs ranged from 0.99 to 1.03. Findings were similar in a linear analysis and in analyses among only non-smokers or stratified by BMI or sex. Schernhammer et al. (23) reported no increased risk of leukemia risk with any level of diet soda intake (<1/week HR = 1.06, 95% CI: 0.75-1.48; 1-3.9/week HR = 1.30, 95% CI: 0.95-1.78; 4-6.9/week HR = 1.26, 95% CI: 0.84-1.87; ≥1/day HR = 1.42, 95% CI: 1.00-2.02), and the overall trend was not significant (p_{trend} = 0.05). Results were similar in analyses stratified by sex.

Aspartame

Two cohort studies assessed the relationship between aspartame intake and leukemia; neither reported an association (23, 95). Lim et al. (95) reported no association between non-lymphoid leukemia and any level of aspartame intake, including the highest level compared to non-consumers (>400 mg/day RR = 1.25, 95% CI: 0.82-1.91) and reported no significant dose-response trend (p_{trend} = 0.19). Similarly, Schernhammer et al. (23) reported no increased risk of leukemia with any level of aspartame intake, including the highest level compared to non-consumers (Quartile 4 HR = 1.23, 95% CI: 0.80-1.91), and there was no significant dose-response trend (p_{trend} = 0.31). Results were similar when stratified by sex.

Case-Control Studies

Non-specific NSSs

Li et al. (96) reported no association between any level of diet cola intake and acute myeloid leukemia (AML) for males or females, and also did not report a significant trend for either sex (females $p_{trend} = 0.97$; males $p_{trend} = 0.56$).

Non-Hodgkin's Lymphoma

Cohort Studies

Non-specific NSSs

McCullough et al. (21), and Schernhammer et al. (23) reported no association between NSS mixtures and NHL overall. McCullough et al. (21) also assessed the relationship between NSSs and several NHL subtypes (*i.e.*, diffuse large B-cell lymphoma [DLBCL], chronic lymphoid leukemia [CLL]/small lymphocytic lymphoma [SLL], follicular lymphoma, other B-cell lymphomas), and reported no associations. Schernhammer et al. (23) reported no association between NSS and NHL in women (RR = 1.00, 95% CI: 0.78-1.26, p_{trend} = 0.999); they reported an increased risk in men who consumed one or more servings of diet soda a day compared to non-consumers (RR = 1.31, 95% CI: 1.01-1.72), but there was no evidence of a dose-response trend (p_{trend} = 0.11).

Aspartame

Lim et al. (95) reported no association between aspartame and NHL for any intake level, including the highest intake level of >200 mg/day compared to non-consumers (RR = 1.02, 95% CI: 0.69, 1.52, p_{trend} = 0.84). Schernhammer et al. (23) reported no association with any level of aspartame intake and NHL, and no dose-response (p_{trend} = 0.12). After stratifying by sex, Schernhammer et al. (23) reported no association among women for any intake level, including the highest level of ≥129 mg per day (RR = 0.91, 95% CI: 0.69-1.20) and no dose-response (p_{trend} = 0.48). In men, they reported an increased risk of NHL in the highest consumers (≥143 mg/day) compared to non-consumers (RR = 1.64, 95% CI: 1.17-2.29) and a significant dose-response trend (p_{trend} = 0.002).

McCullough et al. (21) examined risk of NHL overall and of several NHL subtypes (*i.e.*, DLBCL, CLL/SLL, follicular lymphoma, other B-cell lymphomas). They reported no association between aspartame intake and CLL/SLL or follicular lymphoma overall or in either males or females separately. Compared to those who were in the first quintile of aspartame consumption, they reported an increased risk of NHL overall, DLBCL, and other B-cell lymphomas in quintiles 2 or 3 (RR range: 1.29-1.82) but not for quintiles 4 or 5 (RR range: 1.01-1.39), and there were no dose-response trends ($p_{trends} > 0.51$). In analyses stratified by sex, results for males were similar to the overall results, except for an increased risk of DLBCL in quintile 5 (RR = 1.95, 85% CI = 1.04-3.66), but no trend ($p_{trend} = 0.17$). There was no association between aspartame intake and NHL overall, DLBCL, or other B-cell lymphoma in women.

Case-Control Studies

Non-specific NSSs

One case-control study evaluated associations between NSSs and CLL and reported no association with any level of intake and no dose-response ($p_{trend} = 0.2$) (47). Palomar-Cros et al. (47) also reported no association between consumption of low- or no-calorie soft drinks and CLL in analyses stratified by diabetes status.

Aspartame

Palomar-Cros et al. (47) reported no association between any level of aspartame intake and CLL, and no dose-response ($p_{trend} = 0.6$).

Saccharin

Palomar-Cros et al. (47) reported no association with any level of saccharin intake and CLL and no dose-response ($p_{\text{trend}} = 0.4$). They also reported no association between saccharin and CLL in analyses stratified by diabetes status.

Multiple Myeloma

Cohort Studies

Non-specific NSS

Risk estimates for NSS and multiple myeloma ranged from 0.70 to 1.29 and none were statistically significant (21, 23). The results were mixed when analyses were stratified by sex; Schernhammer et al. (23) reported an increased risk of multiple myeloma among men (p_{trend} = 0.01), but not among women (p_{trend} = 0.79). McCullough et al. (21) did not report any associations in analyses stratified by sex.

Aspartame

Lim et al. (95), McCullough et al. (21), and Schernhammer et al. (23) reported no association between aspartame and multiple myeloma overall, with risk estimates ranging from 0.74-1.39. The results were mixed in analyses stratified by sex. McCullough et al. (21) did not report an association among men but reported a decreased risk of multiple myeloma among women for each 50 mg/day increase in aspartame intake (RR = 0.77, 95% CI: 0.60-0.99). Schernhammer et al. (23) reported a decreased risk among women who consumed up to 19 mg/day of aspartame (RR = 0.40, 95% CI: 0.22-0.74), but no association at any higher level of intake and no overall trend (p_{trend} = 0.48). They reported an increased risk in men who consumed >0-19 mg/ day (RR = 3.33, 95% CI: 1.48-7.49), 60-142 mg/day (RR = 2.96, 95% CI: 1.25-6.96), and ≥143 mg/ day (RR = 3.36, 95%: 1.38-8.19) compared to non-consumers. They did not report an increased risk in men who consumed between 19 and 59 mg/day (RR = 1.70, 95% CI: 0.68-4.23) and the overall trend was not significant (p_{trend} = 0.05).

Supplemental Table J.1 NSS and Leukemia Cohort Study Results

Study	Reference Group	Outcome Type	Cancer Type	Statistical Analysis							Covariate Adjustment
				Risk Metric	NSS	Group	Exposed Cases	Expected Cases or Exposed Non-Cases	Risk Estimate (95% CI)	p_{Trend}	
Lim *et al.* (2006) (95)	No aspartame consumption	Inc	Non-lymphoid leukemia	RR	Aspartame	**Intake Levels (mg/d)**					Age, sex, ethnicity, BMI, and history of diabetes
						>0-<100	62	–	0.93 (0.68-1.27)	0.19	
						100-<200	47		1.31 (0.93-1.85)		
						200-<400	20		1.05 (0.65-1.70)		
						≥400	29		1.25 (0.82-1.91)		
McCullough *et al*. (2022) (26)	Never consume ASBs	Mort	Leukemia	HR	Non-specific	**ASB Consumption[a]**					Age, sex, race/ethnicity, smoking, marital status, education, consumption of red and processed meat, fruits and vegetables, alcohol, and SSBs
						<1 drink/d	–	–	0.99 (0.91-1.07)	0.705	
						1 drink/d			1.03 (0.94-1.14)		
						2+ drink/d			1.01 (0.91-1.12)		
						Continuous (per 1 drink/d)			1.00 (0.97-1.03)	–	
						Men					
						<1 drink/d	–	–	0.97 (0.86-1.10)	0.569	
						1 drink/d			1.10 (0.95-1.26)		
						2+ drink/d			1.01 (0.87-1.18)		
						Continuous (per 1 drink/d)			1.01 (0.97-1.05)	–	
						Women					
						<1 drink/d	–	–	1.00 (0.89-1.11)	0.935	
						1 drink/d			0.98 (0.86-1.12)		
						2+ drink/d			1.02 (0.88-1.17)		
						Continuous (per 1 drink/d)			1.00 (0.96-1.04)	–	

Study	Reference Group	Outcome Type	Cancer Type	Statistical Analysis							
				Risk Metric	NSS	Group	Exposed Cases	Expected Cases or Exposed Non-Cases	Risk Estimate (95% CI)	p_{Trend}	Covariate Adjustment
Schernhammer *et al.* (2012) (23)[b]	No diet soda/ aspartame consumption	Inc	Leukemia	RR	Non-specific	**Diet Soda Consumption (Servings)**					Age, questionnaire cycle, SS soda consumption or total sugar intake, fruit and vegetable consumption, multivitamin use, intakes of alcohol, saturated fat, animal protein, and total energy, race, BMI, height, discretionary physical activity, smoking history, menopausal status, and use of HRT (women only)
						<1/wk	64	–	1.06 (0.75-1.48)	0.05	
						1-3.9/wk	86		1.30 (0.95-1.78)		
						4-6.9/wk	40		1.26 (0.84-1.87)		
						≥1/d	64		1.42 (1.00-2.02)		
						Men					
						<1/wk	33	–	1.07 (0.68-1.68)	0.13	
						1-3.9/wk	49		1.51 (1.00-2.28)		
						4-6.9/wk	19		1.29 (0.75-2.24)		
						≥1/d	33		1.47 (0.92-2.35)		
						Women					
						<1/wk	31	–	1.04 (0.63-1.73)	0.20	
						1-3.9/wk	37		1.05 (0.64-1.72)		
						4-6.9/wk	21		1.21 (0.68-2.17)		
						≥1/d	31		1.36 (0.80-2.31)		
					Aspartame	**Aspartame Intake (mg/d)[c]**					
						Quartile 1	35	–	0.86 (0.56-1.33)	0.31	
						Quartile 2	55		1.47 (1.00-2.17)		
						Quartile 3	40		1.17 (0.77-1.79)		
						Quartile 4	39		1.23 (0.80-1.91)		
						Men					
						>0-<19	14	–	0.89 (0.45-1.77)	0.17	
						19-59	23		1.69 (0.91-3.12)		
						60-142	19		1.55 (0.81-2.94)		
						≥143	18		1.56 (0.79-3.06)		
						Women					
						>0-<19	21	–	0.85 (0.48-1.48)	0.94	
						19-55	32		1.34 (0.81-2.21)		
						56-128	21		0.95 (0.54-1.66)		
						≥129	21		1.04 (0.58-1.85)		

Notes:
ASB = Artificially Sweetened Beverage; BMI = Body Mass Index; CI = Confidence Interval; d = Day; g = Grams; HPFS = Health Professionals Follow-up Study; HR = Hazard Ratio; HRT = Hormone Replacement Therapy; Inc = Incidence; mg = milligram; mo = Month; Mort = Mortality; NSS = Non-Sugar Sweetener; RR = Relative Risk; SS = Sugar-Sweetened; SSB = Sugar-Sweetened Beverage; wk = Week.
– = Not Reported.
Bolded values indicate statistical significance.

(a) There were no statistically significant results for analyses adjusted for or stratified by BMI status (*i.e*., normal, overweight, or obese) or for never smokers (with or without controlling for BMI). Sensitivity analyses excluding the first 2 yrs of follow-up or by total yrs of follow-up produced similar results (26).
(b) Schernhammer *et al*. (23) also evaluated leukemia risk associated with diet soda intake stratified by median alcohol intake (<6 g/d and ≥6 g/d) among men in HPFS, 1986-2006. There were no statistically significant results for any diet soda consumption levels among those consuming ≥ median alcohol intake ($p_{trend} = 0.40$). There was also no evidence of a dose response trend among those consuming < median alcohol ($p_{trend} = 0.38$). A statistically significant increased risk was reported for those consuming < median alcohol and >1-3.9 diet sodas/wk (RR = 2.14, 95% CI: 1.13-4.07, but there were no statistically significant findings for any other level of diet soda consumption (*i.e*., <1/wk, 4-6.9/wk, 1-1.9/d, or ≥2/d).
(c) Schernhammer *et al*. (23) did not provide values for quartiles of aspartame intake in analyses of men and women combined.

Supplemental Table J.2 NSS and Non-Hodgkin's Lymphoma Cohort Study Results

Study	Reference Group	Outcome Type	Cancer Type	Statistical Analysis							Covariate Adjustment
				Risk Metric	NSS	Group	Exposed Cases	Expected Cases or Exposed Non-Cases	Risk Estimate (95% CI)	p_{Trend}	
Lim *et al.* (2006) (95)	No aspartame consumption	Inc	SLL & CLL	RR	Aspartame	**Intake Levels (mg/d)**					Age, sex, ethnicity, BMI, and history of diabetes
						>0-<100	60	–	1.03 (0.75-1.41)	0.84	
						100-<200	36		1.16 (0.79-1.71)		
						>200	36		1.02 (0.69-1.52)		
			Immunoblastic lymphoma and lymphoblastic lymphoma/ leukemia			>0	22		0.77 (0.42-1.42)	0.40	
McCullough *et al.* (2014) (21)	Nondrinkers	Inc	NHL (excluding MM)	RR	Non-specific	**AS Carbonated Beverage Intake (Cans)[a]**					Age, sex, diabetes, BMI, smoking status, energy intake, and SSB intake
						>0-3/mo	349	–	1.13 (0.96-1.33)	0.45	
						1-4/wk	261		1.02 (0.86-1.22)		
						5-6/wk	33		0.79 (0.55-1.14)		
						≥1/d	83		0.97 (0.75-1.26)		
						Continuous (per 1/d)	–		1.00 (0.98-1.02)	–	
			DLBCL			>0-3/mo	90		1.23 (0.89-1.70)	0.89	
						1-4/wk	71		1.12 (0.80-1.59)		
						5-6/wk	30		0.92 (0.59-1.45)		
						≥1/d	–		–		
						Continuous (per 1/d)			1.01 (0.98-1.04)	–	
			CLL/SLL			>0-3/mo	92		0.97 (0.72-1.32)	0.15	
						1-4/wk	68		0.89 (0.64-1.23)		
						5-6/wk	27		0.71 (0.45-1.12)		
						≥1/d	–		–		
						Continuous (per 1/d)			0.99 (0.95-1.02)	–	
			Follicular lymphoma			>0-3/mo	46		1.08 (0.70-1.66)	0.85	
						1-4/wk	36		0.97 (0.61-1.54)		
						5-6/wk	19		0.98 (0.55-1.74)		
						≥1/d	–		–		
						Continuous (per 1/d)			1.00 (0.96-1.04)	–	

Study	Reference Group	Outcome Type	Cancer Type	Statistical Analysis							
				Risk Metric	NSS	Group	Exposed Cases	Expected Cases or Exposed Non-Cases	Risk Estimate (95% CI)	p_{Trend}	Covariate Adjustment
			Other B-cell lymphoma			>0-3/mo	72		1.41 (0.97-2.06)	0.59	
						1-4/wk	51		1.24 (0.82-1.88)		
						5-6/wk	23		1.13 (0.67-1.91)		
						≥1/d	–		–		
						Continuous (per 1/d)			1.00 (0.96-1.04)	–	
	Quintile 1		NHL (excluding MM)		Aspartame	**Aspartame Intake (Median at Baseline, mg/d)**					
						Q2 (3.6)	221	–	**1.31 (1.08-1.60)**	0.83	
						Q3 (12.6)	215		**1.29 (1.06-1.58)**		
						Q4 (35.8)	203		1.18 (0.96-1.45)		
						Q5 (145)	172		1.07 (0.86-1.33)		
						Continuous (per 50)	–		1.00 (0.96-1.04)	–	
						Men					
						Q2 (4.9)	118	–	**1.42 (1.07-1.88)**	0.29	
						Q3 (14.4)	110		**1.45 (1.09-1.92)**		
						Q4 (47.6)	104		1.25 (0.94-1.68)		
						Q5 (155)	98		1.27 (0.94-1.72)		
						Continuous (per 50)	--		1.02 (0.97-1.07)	–	
						Women					
						Q2 (3.6)	103	–	1.20 (0.90-1.59)	0.41	
						Q3 (9.8)	105		1.14 (0.86-1.51)		
						Q4 (31.9)	99		1.11 (0.83-1.47)		
						Q5 (127)	74		0.87 (0.63-1.19)		
						Continuous (per 50)	–		0.97 (0.90-1.04)	–	
			DLBCL			*All Subjects*					
						Q2 (3.6)	63	–	**1.82 (1.22-2.72)**	0.51	
						Q3 (12.6)	56		**1.62 (1.07-2.45)**		
						Q4 (35.8)	50		1.38 (0.90-2.11)		
						Q5 (145)	49		1.39 (0.90-2.16)		
						Continuous (per 50)	–		1.02 (0.94-1.10)	–	

Study	Reference Group	Outcome Type	Cancer Type	Statistical Analysis							
				Risk Metric	NSS	Group	Exposed Cases	Expected Cases or Exposed Non-Cases	Risk Estimate (95% CI)	p_{Trend}	Covariate Adjustment
						Men					
						Q2 (4.9)	32	–	**2.04 (1.13-3.71)**	0.17	
						Q3 (14.4)	30		**2.04 (1.11-3.75)**		
						Q4 (47.6)	25		1.58 (0.84-2.97)		
						Q5 (155)	28		**1.95 (1.04-3.66)**		
						Continuous (per 50)	–		1.07 (0.99-1.17)	–	
						Women					
						Q2 (3.6)	31	–	1.66 (0.96-2.87)	0.59	
						Q3 (9.8)	26		1.28 (0.72-2.27)		
						Q4 (31.9)	25		1.19 (0.67-2.14)		
						Q5 (127)	21		0.97 (0.52-1.81)		
						Continuous (per 50)	–		0.92 (0.80-1.07)	–	
			CLL/SLL			*All Subjects*					
						Q2 (3.6)	58	–	1.05 (0.73-1.52)	0.40	
						Q3 (12.6)	55		1.02 (0.70-1.48)		
						Q4 (35.8)	52		0.95 (0.65-1.39)		
						Q5 (145)	43		0.85 (0.56-1.29)		
						Continuous (per 50)	–		0.96 (0.88-1.05)	–	
						Men					
						Q2 (4.9)	32	–	1.03 (0.62-1.71)	0.91	
						Q3 (14.4)	30		1.20 (0.72-1.99)		
						Q4 (47.6)	25		0.98 (0.58-1.66)		
						Q5 (155)	28		1.05 (0.61-1.80)		
						Continuous (per 50)	–		0.98 (0.88-1.08)	–	
						Women					
						Q2 (3.6)	31	–	1.06 (0.62-1.79)	0.20	
						Q3 (9.8)	26		0.85 (0.49-1.47)		
						Q4 (31.9)	25		0.92 (0.53-1.61)		
						Q5 (127)	21		0.65 (0.34-1.25)		
						Continuous (per 50)	–		0.92 (0.78-1.09)	–	

Study	Reference Group	Outcome Type	Cancer Type	Statistical Analysis							Covariate Adjustment
				Risk Metric	NSS	Group	Exposed Cases	Expected Cases or Exposed Non-Cases	Risk Estimate (95% CI)	p_{Trend}	
			Follicular lymphoma			*All Subjects*					
						Q2 (3.6)	31	–	1.41 (0.84-2.39)	0.72	
						Q3 (12.6)	27		1.22 (0.71-2.10)		
						Q4 (35.8)	29		1.21 (0.71-2.09)		
						Q5 (145)	28		1.20 (0.69-2.11)		
						Continuous (per 50)	–		1.03 (0.94-1.13)	–	
						Men					
						Q2 (4.9)	12	–	1.34 (0.59-3.08)	0.28	
						Q3 (14.4)	11		1.33 (0.57-3.11)		
						Q4 (47.6)	14		1.46 (0.65-3.30)		
						Q5 (155)	15		1.60 (0.70-3.65)		
						Continuous (per 50)	–		1.03 (0.91-1.18)	–	
						Women					
						Q2 (3.6)	19	–	1.45 (0.73-2.86)	0.58	
						Q3 (9.8)	16		1.15 (0.56-2.35)		
						Q4 (31.9)	15		1.05 (0.51-2.17)		
						Q5 (127)	13		0.91 (0.42-1.97)		
						Continuous (per 50)	–		1.02 (0.88-1.17)	–	
			Other B-cell lymphoma			*All Subjects*					
						Q2 (3.6)	37	–	1.18 (0.74-1.89)	0.63	
						Q3 (12.6)	48		**1.58 (1.01-2.46)**		
						Q4 (35.8)	43		1.39 (0.88-2.20)		
						Q5 (145)	29		1.01 (0.60-1.68)		
						Continuous (per 50)	–		0.94 (0.85-1.05)	–	
						Men					
						Q2 (4.9)	22	–	1.35 (0.71-2.54)	0.89	
						Q3 (14.4)	22		1.50 (0.79-2.84)		
						Q4 (47.6)	21		1.34 (0.70-2.56)		
						Q5 (155)	15		1.02 (0.50-2.10)		
						Continuous (per 50)	–		0.98 (0.86-1.11)	–	

Study	Reference Group	Outcome Type	Cancer Type	Statistical Analysis: Risk Metric	NSS	Group	Exposed Cases	Expected Cases or Exposed Non-Cases	Risk Estimate (95% CI)	p_{Trend}	Covariate Adjustment
						Women					
						Q2 (3.6)	15	–	0.97 (0.48-1.96)	0.56	
						Q3 (9.8)	26		1.61 (0.86-2.99)		
						Q4 (31.9)	22		1.44 (0.75-2.75)		
						Q5 (127)	14		0.99 (0.47-2.06)		
						Continuous (per 50)	–		0.90 (0.74-1.09)	–	
Schernhammer *et al.* (2012) (23)[b]	No diet soda/ aspartame consumption	Inc	NHL	RR	Non-specific	**Diet Soda Consumption (Servings)**					Age, questionnaire cycle, SS soda consumption or total sugar intake, fruit and vegetable consumption, multivitamin use, intakes of alcohol, saturated fat, animal protein, and total energy, race, BMI, height, discretionary physical activity, smoking history, menopausal status, and use of HRT (women only)
						<1/wk	289	–	1.04 (0.89-1.22)	0.28	
						1-3.9/wk	297		0.96 (0.82-1.13)		
						4-6.9/wk	140		0.89 (0.72-1.09)		
						≥1/d	237		1.13 (0.94-1.34)		
						Men					
						<1/wk	122	–	1.12 (0.88-1.43)	0.11	
						1-3.9/wk	124		1.06 (0.83-1.34)		
						4-6.9/wk	53		0.96 (0.69-1.32)		
						≥1/d	100		**1.31 (1.01-1.72)**		
						Women					
						<1/wk	167	–	0.98 (0.79-1.22)	0.999	
						1-3.9/wk	173		0.90 (0.72-1.11)		
						4-6.9/wk	87		0.85 (0.65-1.10)		
						≥1/d	137		1.00 (0.78-1.26)		
					Aspartame	**Aspartame Intake (mg/d)**[c]					
						Quartile 1	169	–	0.93 (0.76-1.13)	0.12	
						Quartile 2	175		1.02 (0.83-1.24)		
						Quartile 3	140		0.88 (0.71-1.09)		
						Quartile 4	155		1.16 (0.93-1.43)		
						Men					
						>0-<19	55	–	0.92 (0.65-1.29)	**0.002**	
						19-59	65		1.13 (0.82-1.57)		
						60-142	49		0.98 (0.68-1.40)		
						≥143	69		**1.64 (1.17-2.29)**		

Study	Reference Group	Outcome Type	Cancer Type	Statistical Analysis							
				Risk Metric	NSS	Group	Exposed Cases	Expected Cases or Exposed Non-Cases	Risk Estimate (95% CI)	p_{Trend}	Covariate Adjustment
						Women					
						>0-<19	114	–	0.94 (0.74-1.20)	0.48	
						19-55	110		0.96 (0.75-1.22)		
						56-128	91		0.83 (0.64-1.08)		
						≥129	86		0.91 (0.69-1.20)		

Notes:

BMI = Body Mass Index; CI = Confidence Interval; CLL = Chronic Lymphocytic Leukemia; d = Day; DLBCL = Diffuse Large B-Cell Lymphoma; HPFS = Health Professionals Follow-up Study; HR = Hazard Ratio; HRT = Hormone Replacement Therapy; Inc = Incidence; mg = Milligram; mL = Milliliter; MM = Multiple Myeloma; mo = Month; Mort = Mortality; NHL = Non-Hodgkin's Lymphoma; NSS = Non-Sugar Sweetener; RR = Relative Risk; SLL = Small Lymphocytic Lymphoma; SSB = Sugar-Sweetened Beverage; wk = Week; yrs = Years.

– = Not Reported.

Bolded values indicate statistical significance.

(a) McCullough *et al*. (21) also provided results stratified by sex. Results were generally similar, except males consuming >0-3 cans/mo had an increase in risk of NHL (including MM) and NHL (excluding MM) (RR = 1.31, 95% CI: 1.07-1.63 and RR = 1.40, 95% CI: 1.11-1.77, respectively).

(b) Schernhammer *et al*. (23) also evaluated NHL risks associated with diet soda intake stratified by median alcohol intake (<6 g/d and ≥6 g/d) among men in HPFS, 1986-2006. Statistically significant results were only reported for those consuming low alcohol (<6 g/d and ≥2 diet sodas/d RR = 2.34, 95% CI: 1.46-3.76, p_{trend} = 0.004).

(c) Schernhammer *et al*. (23) did not provide aspartame intake quartile values for men and women combined.

Supplemental Table J.3 NSS and Multiple Myeloma Cohort Study Results

Study	Reference Group	Outcome Type	Cancer Type	Statistical Analysis							Covariate Adjustment
				Risk Metric	NSS	Group	Exposed Cases	Expected Cases or Exposed Non-Cases	Risk Estimate (95% CI)	p_{Trend}	
Lim *et al.* (2006) (95)	No aspartame consumption	Inc	MM	RR	Aspartame	**Intake Levels (mg/d)**					Age, sex, ethnicity, BMI, and history of diabetes
						>0 -<100	57	–	0.85 (0.62-1.17)	0.40	
						100-<200	48		1.39 (0.99-1.96)		
						200-<400	20		1.13 (0.70-1.83)		
						≥400	21		1.03 (0.64-1.66)		
McCullough *et al.* (2014) (21)	Nondrinkers	Inc	MM	RR	Non-specific	**AS Carbonated Beverage Intake (Cans)[a]**					Age, sex, diabetes, BMI, smoking status, energy intake, and SSB intake
						>0-3/mo	75	–	1.15 (0.81-1.63)	0.05	
						1-4/wk	42		0.71 (0.47-1.07)		
						5-6/wk	22		0.70 (0.42-1.17)		
						≥1/d	–		–		
						Continuous (per 1/d)			0.97 (0.92-1.01)	–	
			MM		Aspartame	**Aspartame Intake (Median at Baseline, mg/d)**					
						All subjects					
						Q2 (3.6)	45	–	1.18 (0.77-1.81)	0.14	
						Q3 (12.6)	45		1.18 (0.77-1.81)		
						Q4 (35.8)	31		0.74 (0.46-1.20)		
						Q5 (145)	34		0.83 (0.51-1.33)		
						Continuous (per 50)	–		0.93 (0.84-1.04)	–	
						Men					
						Q2 (4.9)	23	–	1.11 (0.62-1.98)	0.85	
						Q3 (14.4)	26		1.37 (0.77-2.42)		
						Q4 (47.6)	18		0.84 (0.45-1.57)		
						Q5 (155)	22		1.05 (0.57-1.95)		
						Continuous (per 50)	–		1.00 (0.90-1.11)	–	
						Women					
						Q2 (3.6)	22	–	1.23 (0.66-2.30)	0.05	
						Q3 (9.8)	19		0.98 (0.52-1.88)		
						Q4 (31.9)	13		0.63 (0.31-1.31)		
						Q5 (127)	12		0.59 (0.28-1.26)		
						Continuous (per 50)	–		**0.77 (0.60-0.99)**	--	

Study	Reference Group	Outcome Type	Cancer Type	Statistical Analysis							
				Risk Metric	NSS	Group	Exposed Cases	Expected Cases or Exposed Non-Cases	Risk Estimate (95% CI)	p_{Trend}	Covariate Adjustment
Schernhammer *et al.* (2012) (23)[b]	No diet soda/ aspartame consumption	Inc	MM	RR	Non-specific	**Diet Soda Consumption (Servings)**					Age, questionnaire cycle, SS soda consumption or total sugar intake, fruit and vegetable consumption, multivitamin use, intakes of alcohol, saturated fat, animal protein, and total energy, race, BMI, height, discretionary physical activity, smoking history, menopausal status, and use of HRT (women only)
						<1/wk	55	–	0.91 (0.63-1.30)	0.10	
						1-3.9/wk	63		0.94 (0.66-1.33)		
						4-6.9/wk	35		1.00 (0.65-1.52)		
						≥1/d	53		1.29 (0.89-1.89)		
						Men					
						<1/wk	27	–	1.17 (0.70-1.96)	**0.01**	
						1-3.9/wk	23		1.04 (0.61-1.78)		
						4-6.9/wk	12		1.08 (0.55-2.12)		
						≥1/d	29		**2.02 (1.20-3.40)**		
						Women					
						<1/wk	28	–	0.71 (0.43-1.17)	0.79	
						1-3.9/wk	40		0.86 (0.54-1.37)		
						4-6.9/wk	23		0.95 (0.55-1.63)		
						≥1/d	24		0.79 (0.45-1.36)		
					Aspartame	**Aspartame Intake (mg/d)[c]**					
						Quartile 1	31	–	0.86 (0.53-1.41)	0.44	
						Quartile 2	36		0.92 (0.59-1.44)		
						Quartile 3	39		1.16 (0.75-1.81)		
						Quartile 4	28		1.03 (0.62-1.72)		
						Men					
						>0-<19	17	–	**3.33 (1.48-7.49)**	0.05	
						19-59	11		1.70 (0.68-4.23)		
						60-142	14		**2.96 (1.25-6.96)**		
						≥143	13		**3.36 (1.38-8.19)**		
						Women					
						>0-<19	14	–	**0.40 (0.22-0.74)**	0.48	
						19-55	25		0.76 (0.46-1.27)		
						56-128	25		0.83 (0.50-1.39)		
						≥129	15		0.59 (0.32-1.09)		

Notes:
ASB = Artificially Sweetened Beverage; BMI = Body Mass Index; CI = Confidence Interval; d = Day; g = Grams; HR = Hazard Ratio; HRT = Hormone Replacement Therapy; Inc = Incidence; mg = Milligram; mL = Milliliter; MM = Multiple Myeloma; mo = Month; Mort = Mortality; NSS = Non-Sugar Sweetener; RR = Relative Risk; SS = Sugar-Sweetened; SSB = Sugar-Sweetened Beverage; wk = Week; yr = Year.
– = Not Reported.
Bolded values indicate statistical significance.

(a) McCullough *et al*. (21) also provided results stratified by sex and results were similar.
(b) Schernhammer *et al*. (23) also evaluated risk of MM associated with diet soda intake stratified by median alcohol intake (<6 g/d and ≥6 g/d) among men in HPFS, 1986-2006. Statistically significant results were only reported for those consuming low amounts of alcohol (<6 g/d) and ≥2 diet sodas/d (RR = 3.79, 95% CI: 1.80-8.00, p_{trend} = 0.002).
(c) Schernhammer *et al*. (23) did not provide aspartame intake quartile values for men and women combined.

Supplemental Table J.4 NSS and Leukemia Case-Control Study Results

Study	Outcome Type	Cancer Type	Statistical Analysis							Covariate Adjustment
			Risk Metric	NSS	Group	Exposed Cases	Exposed Controls	Risk Estimate (95% CI)	p_{Trend}	
Li *et al*. (2006) (96)	Inc	AML	OR	Non-specific	**Diet Cola Consumption (Drinks/d)[a]**					Age, education, smoking status, and monthly intake of calories
					Males					
					0	36	162	Ref	0.56	
					3.5[a]	12	30	0.75 (0.31-1.81)		
					>3.5	8	29	1.62 (0.57-4.62)		
					Females					
					0	35	131	Ref	0.97	
					3.5[a]	9	49	0.92 (0.41-2.06)		
					>3.5	11	38	0.63 (0.23-1.72)		

Notes:
AML = Acute Myeloid Leukemia; CI = Confidence Interval; d = Day; Inc = Incidence; NSS = Non-Sugar Sweetener; OR = Odds Ratio; Ref = Reference.
(a) Li *et al*. (96) reported risk of AML for three categories of diet cola consumption. In the table they report that risks are assessed per category of daily drink consumption, but in the text they say that risks are assessed by weekly drink consumption. They report risks for three categories of diet cola consumption, including the reference category, but do not specify the upper limit of the lowest category (*i.e*. reference category) or the lower limit of the middle category.

Supplemental Table J.5 NSS and Non-Hodgkin's Lymphoma Case-Control Study Results

Study	Outcome Type	Cancer Type	Statistical Analysis							
			Risk Metric	NSS	Group	Exposed Cases	Exposed Controls	Risk Estimate (95% CI)	p_{Trend}	Covariate Adjustment
Palomar-Cros *et al*. (2023) (47)	Inc	CLL	OR	Aspartame[a]	**Level of Intake[b]**					Age, sex, study center, education, smoking, radiation exposure, total WCRF score continuous, total energy intake, total sugar intake, and aspartame or other AS consumption[d]
					All Participants[c]					
					Non-consumers	89	1,294	Ref	0.6	
					Medium	9	235	0.56 (0.25-1.08)		
					High	11	102	1.76 (0.84-3.41)		
				Other AS (excluding aspartame)[a]	Non-consumers	57	961	Ref	0.2	
					Medium	38	492	1.35 (0.86-2.08)		
					High	14	178	1.36 (0.70-2.51)		
				Saccharin	Non-consumers	74	1,174	Ref	0.4	Age, sex, study center, education, smoking, radiation exposure, total WCRF score continuous, total energy intake, total sugar intake, and other sources of sweeteners
					Medium	21	293	1.08 (0.63-1.78)		
					High	14	164	1.35 (0.70-2.45)		

Notes:

AS = Artificial Sweetener; ASB = Artificially Sweetened Beverage; CI = Confidence Interval; CLL = Chronic Lymphocytic Leukemia; d = Day; Inc = Incidence; NSS = Non-Sugar Sweetener; OR = Odds Ratio; Ref = Reference; TT = Tabletop; WCRF = World Cancer Research Fund.

(a) Palomar-Cros *et al*. (47) used "public sources of nutritional information (https://es.openfoodfacts.org/) to determine the most common type of sweetener in each of these food items." They combined non-saccharin TT use, which they reported to be primarily aspartame, and low or no calorie soft drinks into the aspartame category. For the "Other AS" intake category, the authors combined TT saccharin and gaseosa, which is an ASB beverage in Spain that is usually sweetened with saccharin and cyclamate.

(b) Sex-specific quartiles among consumers and controls were used to compare moderate (<3rd quartile) and high (≥3rd quartile) consumers to non-consumers (reference) (47).

(c) No associations were reported in analyses stratified by diabetes status or in sensitivity analyses based on consumption of low- or no-calorie soft drinks (47).

(d) Sensitivity analyses adjusted for individual confounders (*i.e.*, BMI, dietary fiber, red meat, physical activity, and alcohol) instead of the WCRF score, with or without adjustment for BMI or weight change from prior year, produced similar results (47).

Supplement K: Female Reproductive System

Ovarian Cancer

Cohort Studies

Non-specific NSSs

Both cohort studies evaluated ovarian cancer risk and NSS mixtures. Risk estimates ranged from 0.80 to 1.39; most estimates were slightly higher than 1 (44, 26). McCullough et al. (26) reported an increased risk of ovarian cancer mortality among those who consumed one ASB/day (HR = 1.17, 95% CI: 1.05-1.32), but there was no association with intake of <1 (OR = 1.07, 95% CI: 0.97-1.18), or ≥2 (OR = 1.03, 95% CI: 0.91-1.17) ASBs/day, and no dose-response (p_{trend} = 0.137). Hodge et al. (44) reported no increased risk with any level of NSS intake and no dose-response (p_{trend} = 0.17).

Case-Control Studies

Non-specific NSSs

Gallus et al. (46) reported a decreased risk of ovarian cancer with increasing AS consumption (p_{trend} = <0.01). The decreased risk was reported across all levels of intake (>0-2 sachets or tablets OR = 0.68, 95% CI: 0.49-0.95; >2 sachets or tablets OR = 0.56, 95% CI: 0.38-0.81). Gallus et al. (46) reported no association between any non-saccharin AS intake (reported to be mainly aspartame) and ovarian cancer (OR = 0.75, 95% CI: 0.56-1.00).

Saccharin

Gallus et al. (46) reported a decreased risk of ovarian cancer with any saccharin intake compared to those who did not report using saccharin tablets or sachets (OR = 0.46, 95% CI: 0.29-0.74).

Uterine Cancer

Cohort Studies

Non-specific NSSs

All three cohort studies evaluated uterine cancer risk and NSS mixtures (26, 44, 97). Risk estimates ranged from 0.58 to 1.18, with estimates below and above 1; most were close to 1 (26, 44, 97). None of the studies reported an association between any level of NSS intake and uterine cancer, but McCullough et al. (26) reported a statistically significant trend with increasing ASB consumption (p_{trend} = 0.049), but the trend was not statistically significant in analyses that controlled for BMI (p_{trend} = 0.878).

Case-Control Studies

Non-specific NSSs

Bosetti et al. (80) reported no association between uterine cancer and intake of any low-calorie sweeteners (OR = 0.96, 95% CI: 0.67-1.40) or non-saccharin low-calorie sweeteners (reported to be mostly aspartame) (OR = 1.07, 95% CI: 0.71-1.61).

Saccharin

Bosetti et al. (80) reported no association between endometrial cancer and saccharin intake (OR = 0.71, 95% CI: 0.36-1.38).

Supplemental Table K.1 NSS and Ovarian Cancer Cohort Study Results

Study	Reference Group	Outcome Type	Cancer Type	Statistical Analysis							
				Risk Metric	NSS	Group	Exposed Cases	Expected Cases or Exposed Non-Cases	Risk Estimate (95% CI)	p_{Trend}	Covariate Adjustment
Hodge *et al*. (2018) (44)	Never or <1/mo	Inc	Ovarian	HR	Non-specific	**Frequency of AS Soft Drink Consumption**[a]					Age, sex, SEIFA, country of birth, alcohol intake, smoking status, physical activity, Mediterranean diet score, SS soft drink consumption, and waist circumference
						1-3/mo	20	–	0.80 (0.38-1.69)	0.17	
						1-6/wk	24		1.39 (0.83-2.34)		
						≥1/d	11		1.37 (0.72-2.61)		
McCullough *et al*. (2022) (26)	Never consume ASBs	Mort	Ovarian	HR	Non-specific	**ASB Consumption**[b]					Age, sex, race/ethnicity, smoking, marital status, education, consumption of red and processed meat, fruits and vegetables, alcohol, and SSBs, parity, age at menarche, estrogen use, OC use, age at first live birth, menopausal status, hysterectomy, uterine surgery, surgically induced menopause, and history of oophorectomy
						<1 drink/d	–	–	1.07 (0.97-1.18)	0.137	
						1 drink/d			**1.17 (1.05-1.32)**		
						2+ drink/d			1.03 (0.91-1.17)		
						Continuous (per 1 drink/d)			1.01 (0.98-1.05)	–	

Notes:
AS = Artificial Sweetener; ASB = Artificially Sweetened Beverage; BMI = Body Mass Index; CI = Confidence Interval; d = Day; HR = Hazard Ratio; Inc = Incidence; mo = Month; NSS = Non-Sugar Sweetener; OC = Oral Contraceptive; SEIFA = Socio-Economic Indexes for Areas; SS = Sugar-Sweetened; SSB = Sugar-Sweetened Beverage; wk = Week; yr = Year.
– = Not Reported.
Bolded values indicate statistical significance.
(a) Hodge *et al*. (44) reported similar associations when excluding the first 2 yrs of follow-up. They did not observe an association when evaluating linear trends on a log hazard scale.
(b) In analyses conducted among never smokers (with or without BMI adjustment), risks for those who consumed 1 drink/d was no longer statistically significant. When considering BMI status (*i.e*., normal, overweight, or obese), those with normal weight that consumed 1 drink/d had an elevated risk (HR = 1.25, 95% CI: 1.07-1.45). Sensitivity analyses excluding the first 2 yrs of follow-up or with stratification by 10-yr follow-up time also produced similar results (26).

Supplemental Table K.2 NSS and Uterine Cancer Cohort Study Results

Study	Reference Group	Outcome Type	Cancer Type	Statistical Analysis							
				Risk Metric	NSS	Group	Exposed Cases	Expected Cases or Exposed Non-Cases	Risk Estimate (95% CI)	p_{Trend}	Covariate Adjustment
Hodge *et al.* (2018) (44)	Never or <1/mo	Inc	Endometrial	HR	Non-specific	**Frequency of AS Soft Drink Consumption**[a]					Age, sex, SEIFA, country of birth, alcohol intake, smoking status, physical activity, Mediterranean diet score, SS soft drink intake, and waist circumference
						1-3/mo	9	–	0.58 (0.29-1.16)	0.78	
						1-6/wk	23		1.11 (0.70-1.77)		
						≥1/d	10		0.81 (0.42-1.55)		
Inoue-Choi *et al.* (2013) (97)	No sugar-free beverage consumption	Inc	Type 1 endometrial	HR	Non-specific	**Sugar-Free Beverages Intake Level (Servings/wk)**					Age, smoking, physical activity, alcohol use, estrogen use, age at menarche, age at menopause, number of live births, history of diabetes, coffee intake, and BMI[b]
						>0-0.0002	36	–	0.69 (0.48-1.00)	0.31	
						0.0003-0.4	93		0.85 (0.65-1.11)		
						0.5-2.8	125		1.03 (0.80-1.32)		
						2.8-64.1	100		0.77 (0.59-1.01)		
			Type 2 endometrial			>0-0.0002	8		0.78 (0.34-1.79)	0.95	
						0.0003-0.4	13		0.66 (0.33-1.30)		
						0.5-2.8	21		1.09 (0.61-1.95)		
						2.8-64.1	17		0.89 (0.48-1.68)		

Study	Reference Group	Outcome Type	Cancer Type	Statistical Analysis							
				Risk Metric	NSS	Group	Exposed Cases	Expected Cases or Exposed Non-Cases	Risk Estimate (95% CI)	p_{Trend}	Covariate Adjustment
McCullough *et al*. (2022) (26)	Never consume ASBs	Mort	Uterine	HR	Non-specific	**ASB Consumption**[c]					Age, sex, race/ethnicity, smoking, marital status, education, red and processed meat, fruits and vegetables, alcohol, and SSB consumption, parity, age at menarche, estrogen use, OC use, age at first live birth, menopausal status, hysterectomy, uterine surgery, and surgically induced menopause
						<1 drink/d	–	–	1.04 (0.90-1.19)	**0.049**	
						1 drink/d			1.07 (0.90-1.26)		
						2+ drink/d			1.18 (1.00-1.40)		
						Continuous (per 1 drink/d)			1.04 (0.99-1.09)	–	

Notes:
AS = Artificial Sweetener; ASB = Artificially Sweetened Beverage; BMI = Body Mass Index; CI = Confidence Interval; d = Day; HR = Hazard Ratio; Inc = Incidence; mo = Month; NSS = Non-Sugar Sweetener; OC = Oral Contraceptive; SEIFA = Socio-Economic Indexes for Areas; SS = Sugar-Sweetened; SSB = Sugar-Sweetened Beverage; wk = Week; yr = Year.
– = Not Reported.
Bolded values indicate statistical significance.
(a) Hodge *et al*. (44) reported similar associations when excluding the first 2 yrs of follow-up. They did not observe an association when evaluating linear trends on a log hazard scale.
(b) Models excluding women who reported history of diabetes at baseline had similar results (97).
(c) There were no analyses that controlled for BMI, were conducted only among never smokers, or stratified results by BMI status (*i.e.*, normal, overweight, or obese) that had statistically significant results. Sensitivity analyses excluding the first 2 yrs of follow-up or with stratification by 10-yr follow-up time produced similar results as the main model (26).

Supplemental Table K.3 NSS and Ovarian Cancer Case-Control Study Results

Study	Outcome Type	Cancer Type	Statistical Analysis							Covariate Adjustment
			Risk Metric	NSS	Group	Exposed Cases	Exposed Controls	Risk Estimate (95% CI)	p_{Trend}	
Gallus *et al.* (2007) (46)	Inc	Ovarian	OR	Non-specific	**All AS Consumption (Sachets or Tablets/d)[a]**					Age, sex, study center, education, tobacco smoking, alcohol drinking, BMI, total energy intake, consumption of hot beverages, parity, and menopausal status/ age at menarche
					0	936	2,053	Ref	**<0.001**	
					>0-2	55	182	**0.68 (0.49-0.95)**		
					>2	40	176	**0.56 (0.38-0.81)**		
					Per sachet or tablet/d	–	–	**0.87 (0.80-0.94)**	–	
				Saccharin	**Saccharin Consumption (Sachets or Tablets/d)**					
					0	1,007	2,285	Ref	–	
					>0	24	126	**0.46 (0.29-0.74)**		
				Non-specific (mainly aspartame)	**AS (Other than Saccharin) Consumption (Sachets or Tablets/d)**					
					0	958	2,175	Ref	–	
					>0	73	236	0.75 (0.56-1.00)		

Notes:
AS = Artificial Sweetener; BMI = Body Mass Index; CI = Confidence Interval; d = Day; Inc = Incidence; NSS = Non-Sugar Sweetener; OR = Odds Ratio; Ref = Reference.
– = Not Reported.
Bolded values indicate statistical significance.
(a) Gallus *et al*. (46) also conducted analyses of continuous AS consumption (*i.e*., per sachet or tablet/d) and ovarian cancer stratified by BMI and age. Decreased risks were observed among women with BMI <25 kg/m^2 (OR = 0.80, 95% CI: 0.69-0.93) and ≥25 kg/m^2 (OR = 0.91, 95% CI: 0.82-0.99) and ages <60 (OR = 0.88, 95% CI: 0.79-0.96) and ≥60 years (OR = 0.85, 95% CI: 0.74-0.98).

Supplemental Table K.4 NSS and Uterine Cancer Case-Control Study Results

Study	Outcome Type	Cancer Type	Statistical Analysis							Covariate Adjustment
			Risk Metric	NSS	Group	Exposed Cases	Exposed Controls	Risk Estimate (95% CI)	p_{Trend}	
Bosetti *et al.* (2009) (80)	Inc	Endometrial	OR	Non-specific	**Consumption of Any Low-Calorie Sweeteners**					Age, study center, yr of interview, education, BMI, tobacco smoking, history of diabetes, consumption of hot beverages, and total energy intake
					Nonusers	378	780	Ref	–	
					Users	73	123	0.96 (0.67-1.40)		
					**Consumption of Any Low-Calorie Sweeteners (Excluding Saccharin)[a]**					
					Nonusers	394	816	Ref	–	
					Users	58	87	1.07 (0.71-1.61)		
				Saccharin	**Consumption of Saccharin**					
					Nonusers	436	867	Ref	–	
					Users	16	39	0.71 (0.36-1.38)		

Notes:
BMI = Body Mass Index; CI = Confidence Interval; Inc = Incidence; NSS = Non-Sugar Sweetener; OR = Odds Ratio; Ref = Reference; yr = Year.
– = Not Reported.
(a) Bosetti *et al.* (80) reported that low-calorie sweeteners excluding saccharin were "mostly aspartame."

Supplement L: Prostate Cancer

Cohort Studies

Non-specific NSSs

All four cohort studies assessed NSS mixtures. Risk estimates ranged from 0.81 to 1.36; most were close to 1 and not statistically significant (20, 26, 27, 44). All four studies evaluated risk by level of NSS intake, and three reported no increased risk with any level of intake (20, 26, 44) and no significant dose-response trends ($p_{\text{trends}} > 0.05$). Mullee et al. (27) evaluated monthly, weekly and daily consumption of AS soft drinks and reported an increased risk in those who consumed AS soft drinks weekly (HR = 1.36, 95% CI: 1.05-1.78), but no increased risk in those who consumed more frequently (*i.e.*, daily) (HR = 1.05, 95% CI: 0.64-1.75), and they reported no dose-response trend ($p_{\text{trend}} = 0.53$).

Ace-K

Debras et al. (20) was the only study to evaluate ace-K and prostate cancer. They reported no increased risk associated with low (HR = 1.06, 95% CI: 0.81-1.39) or high (HR = 1.18, 95% CI: 0.82-1.71) intake and no dose-response ($p_{\text{trend}} = 0.365$).

Aspartame

Debras et al. (20) was the only study to evaluate aspartame and prostate cancer. They reported no association between low (HR = 0.95, 95% CI: 0.70-1.30) or high (HR = 1.28, 95% CI: 0.91-1.79) intake and no dose-response ($p_{\text{trend}} = 0.280$).

Sucralose

Debras et al. (20) was the only study to evaluate sucralose and prostate cancer. They reported no association between low (HR = 0.86, 95% CI: 0.57-1.30) or high (HR = 1.01, 95% CI: 0.57-1.77) intake and no dose-response (p_{trend} = 0.699).

Case-Control Studies

Non-specific NSSs

Two case-control studies evaluated non-specific NSSs and prostate cancer; risk estimates ranged from 0.81 to 1.23 (46, 47). Neither study reported an association with any level of NSS intake and prostate cancer and neither reported a dose-response trend (p_{trend} > 0.1).

Aspartame

Palomar-Cros et al. (47) was the only case-control study to evaluate aspartame intake and prostate cancer risk. They reported no association with medium (OR = 0.81, 95% CI: 0.61-1.07) or high (OR = 0.96, 95% CI: 0.63-1.46) levels of intake compared to non-users, and no dose-response trend (p_{trend} = 0.4).

Saccharin

Both Palomar-Cros et al. (47) and Gallus et al. (46) evaluated saccharin intake and prostate cancer risk. Gallus et al. (46) reported no association between any saccharin tablet or sachet use and

prostate cancer (OR = 0.91, 95% CI: 0.59-1.40). Palomar-Cros et al. (47) reported no association between medium (OR = 0.84, 95% CI: 0.65-1.07) or high (OR = 0.78, 95% CI: 0.57-1.07) use of saccharin tablets or sachets compared to non-users and no dose-response trend (p_{trend} = 0.06).

Supplemental Table L.1 NSS and Prostate Cancer Cohort Study Results

Study	Reference Group	Outcome Type	Cancer Type	Statistical Analysis							
				Risk Metric	NSS	Group	Exposed Cases	Expected Cases or Exposed Non-Cases	Risk Estimate (95% CI)	p_{Trend}	Covariate Adjustment
Debras *et al.* (2022) (20)	Non-consumers	Inc	Prostate	HR	Total AS	**Level of Intake[a,b]**					Age, sex, BMI, height, % weight gain during follow-up, physical activity, smoking status, number of smoked cigarettes in pack-yrs, educational level, number of 24-hr dietary records, family history of cancer, prevalent diabetes, energy intake without alcohol, daily intake of alcohol, sodium, saturated fatty acids, fiber, sugar, fruit and vegetables, whole-grain foods, and dairy products, and other AS
						Low	63	3,242	0.92 (0.70-1.22)	0.274	
						High	58	3,248	1.26 (0.94-1.68)		
					Aspartame	Low	49	2,297	0.95 (0.70-1.30)	0.280	
						High	44	2,307	1.28 (0.91-1.79)		
					Acesulfame-K	Low	76	2,947	1.06 (0.81-1.39)	0.365	
						High	39	2,984	1.18 (0.82-1.71)		
					Sucralose	Low	25	1,208	0.86 (0.57-1.30)	0.699	
						High	13	1,230	1.01 (0.57-1.77)		
Hodge *et al*. (2018) (44)	Never or <1/mo	Inc	Aggressive prostate	HR	Non-Specific	**Frequency of AS Soft Drink Consumption[c]**					Age, sex, SEIFA, country of birth, alcohol intake, smoking status, physical activity, Mediterranean diet score, SS soft drink consumption, and waist circumference
						1–3/mo	33	–	0.94 (0.65-1.36)	0.66	
						1–6/wk	50		1.09 (0.80-1.48)		
						>1/d	17		0.81 (0.49-1.33)		
McCullough *et al*. (2022) (26)	Never consume ASBs	Mort	Prostate	HR	Non-specific	**ASB Consumption[d]**					Age, sex, race/ethnicity, smoking, marital status, education, consumption of red and processed meat, fruits and vegetables, alcohol, and SSBs
						<1 drink/d	–	–	0.99 (0.92-1.07)	0.258	
						1 drink/d			0.91 (0.83-1.00)		
						2+ drink/d			0.98 (0.89-1.08)		
						Continuous (per 1 drink/d)			0.99 (0.97-1.02)	–	

Study	Reference Group	Outcome Type	Cancer Type	Statistical Analysis							
				Risk Metric	NSS	Group	Exposed Cases	Expected Cases or Exposed Non-Cases	Risk Estimate (95% CI)	p_{Trend}	Covariate Adjustment
Mullee *et al.* (2019) (27)	<1 glass/ mo[e]	Mort	Prostate	HR	Non-specific	**AS Soft Drink Consumption (Glasses)[e]**					Age, sex, EPIC center, BMI, physical activity, education, alcohol consumption, smoking status, intensity, and duration, ever use of contraceptive pill, menopausal status, ever use of menopausal hormone therapy,[f] intakes of total energy, red and processed meat, fruits and vegetables, coffee, fruit and vegetable juice, and SS soft drinks
						1-4/mo	–	–	1.23 (0.95-1.60)	0.53	
						>1-6/wk			**1.36 (1.05-1.78)**		
						≥1/d			1.05 (0.64-1.75)		

Notes:
AS = Artificial Sweetener; ASB = Artificially Sweetened Beverage; BMI = Body Mass Index; CI = Confidence Interval; d = day; EPIC = European Prospective Investigation into Cancer and Nutrition; HPFS = Health Professionals Follow-up Study; hr = Hour; HR = Hazard Ratio; Inc = Incidence; mg = Milligrams; mL = Milliliter; mo = Month; Mort = Mortality; NHS = Nurses' Health Study; NSS = Non-Sugar Sweetener; SEIFA = Socio-Economic Indexes for Areas; SS = Sugar-Sweetened; SSB = Sugar-Sweetened Beverage; wk = week; yr = Year.
– = Not Reported.
Bolded values indicate statistical significance.
(a) Debras *et al*. (20) also evaluated prostate cancer risk for consumers *vs.* non-consumers, using three category intake models, and in 12 sensitivity analyses that considered adjustment for additional confounders, exclusion or restriction of certain participants, and the time-dependent nature of AS exposures. Results were generally similar to those from main model analyses.
(b) High *vs*. low consumers were separated at the median for each sweetener type: 17.44 mg/d in men and 19.00 mg/d in women for total AS, 14.45 mg/d in men and 15.39 mg/d in women for aspartame, 5.06 mg/d in men and 5.50 mg/d in women for acesulfame-K, and 3.46 mg/d in men and 3.43 mg/d in women for sucralose (20).
(c) Hodge *et al*. (44) reported similar associations when excluding the first 2 yrs of follow-up. They did not observe an association when evaluating linear trends on a log hazard scale.
(d) In analyses conducted among never smokers only (with or without BMI controlled) or stratified by BMI status (*i.e.*, normal, overweight, or obese), among those with normal weight, risks were decreased in those with 1 drink/d consumption. Sensitivity analyses excluding the first 2 yrs of follow-up or with stratification by 10-yr follow-up time produced similar results as the main analysis (26).
(e) One glass = ~250 mL (27).
(f) Mullee *et al*. (27) stated that they adjusted for ever use of contraceptive pill, menopausal status, and ever use of menopausal hormone therapy even though this would not seem necessary for analyses of prostate cancer.

Supplemental Table L.2 NSS and Prostate Cancer Case-Control Study Results

Citation	Outcome Type	Cancer Type	Statistical Analysis							
			Risk Metric	NSS	Group	Exposed Cases	Exposed Controls	Risk Estimate (95% CI)	p_{Trend}	Covariate Adjustment
Gallus *et al*. (2007) (46)	Inc	Prostate	OR	Non-specific	**All AS Consumption (Sachets or Tablets/d)[a]**					Age, sex, study center, education, tobacco smoking, alcohol drinking, BMI, total energy intake, and consumption of hot beverages
					0	1,179	1,335	Ref	0.492	
					>0-2	56	63	0.97 (0.66-1.43)		
					>2	59	52	1.19 (0.80-1.79)		
					Per sachet or tablet/d	–	–	1.03 (0.95-1.12)	–	
				Saccharin	**Saccharin Consumption (Sachets or Tablets/d)**					
					0	1,252	1,402	Ref	–	
					>0	42	49	0.91 (0.59-1.40)		
				Non-specific (mainly aspartame)	**AS (Other than Saccharin) Consumption (Sachets or Tablets/d)**					
					0	1,217	1,382	Ref	–	
					>0	77	68	1.23 (0.86-1.76)		
Palomar-Cros *et al*. (2023) (47)	Inc	Prostate	OR	Aspartame[b]	**Level of Intake[c]**					Age, study center, education, smoking, radiation exposure, total WCRF score continuous, total energy intake, total sugar intake, family history of prostate cancer, night shift work, and aspartame or other AS consumption[e]
					All Participants[d]					
					Non-consumers	814	1,070	Ref	0.4	
					Medium	110	172	0.81 (0.61-1.07)		
					High	48	66	0.96 (0.63-1.46)		
				Other AS (excluding aspartame)[b]	Non-consumers	564	727	Ref	0.1	
					Medium	314	420	0.95 (0.78-1.16)		
					High	94	161	0.78 (0.57-1.05)		
				Saccharin	Non-consumers	731	923	Ref	0.06	Age, sex, study center, education, smoking, radiation exposure, total WCRF score continuous, total energy intake, total sugar intake, and other sources of sweeteners
					Medium	154	239	0.84 (0.65-1.07)		
					High	87	146	0.78 (0.57-1.07)		

Notes:
AS = Artificial Sweetener; BMI = Body Mass Index; CI = Confidence Interval; d = Day; Inc = Incidence; NSS = Non-Sugar Sweetener; OR = Odds Ratio; Ref = Reference; WCRF = World Cancer Research Fund.
– = Not Reported.
(a) Gallus *et al*. (46) also conducted analyses for continuous AS consumption (*i.e.*, per sachet or tablet/d) and prostate cancer stratified by BMI and age. No associations were observed.
(b) Palomar-Cros *et al*. (47) used "public sources of nutritional information (https://es.openfoodfacts.org/) to determine the most common type of sweetener in each of these food items." They combined non-saccharin TT use, which they reported to be primarily aspartame, and low or no calorie soft drinks into the aspartame category. For the "Other AS" intake category the authors combined TT saccharin and gaseosa, which is an ASB beverage in Spain that is usually sweetened with saccharin and cyclamate.

(c) Sex-specific quartiles among were used to compare moderate (<3rd quartile) and high (≥3rd quartile) consumers to non-consumers (reference) (47).
(d) In sensitivity analyses, when considering only consumption of low- or no-calorie soft drinks, lower prostate cancer risks were associated with medium intake in all participants (OR = 0.72, 95% CI: 0.53-0.98) and participants without diabetes (OR = 0.75, 95% CI: 0.58-0.96). No other associations were reported in analyses stratified by diabetes status. High intake of other AS among all participants was associated with lower risks of low-grade prostate cancer (OR = 0.59, 95% CI: 0.38-0.90) with a negative exposure-response trend (p_{trend} = 0.04)(47).
(e) Sensitivity analyses adjusted for individual confounders (*i.e*., BMI, dietary fiber, red meat, physical activity, and alcohol) instead of the WCRF score with and without adjustment for BMI, weight change from prior year, and consumption of dairy products did not change results (47).

Supplement M: Brain Cancer

Cohort Studies

Non-specific NSS

Only one cohort study evaluated NSS mixtures and brain cancer (26). This study reported no association between any level of ASB intake and brain cancer mortality and no dose-response (p_{trend} = 0.714). Results were similar when stratified by sex (women p_{trend} = 0.76; men p_{trend} = 0.91) (26). All risk estimates were close to 1. There were no associations in never-smokers or in sensitivity analysis controlling for BMI. In analyses stratified by BMI status (*i.e.*, normal, overweight, obese), overweight subjects consuming less than 1 drink per day had an elevated risk of brain cancer mortality (HR = 1.19, 95% CI: 1.02-1.40), but there were no associations with higher levels of intake or in other BMI categories.

Aspartame

Lim et al. (95) reported no association between any level of aspartame intake and glioma and no dose-response (p_{trend} = 0.05). All risk estimates were <1 (range: 0.66-0.99) and not statistically significant.

Case-Control Studies

Non-specific NSS

Two case-control studies evaluated non-specific NSS and brain cancer (98, 101). Bunin et al. (98) reported no association between childhood brain cancer (*i.e.*, medulloblastoma/primitive neuroectodermal tumors) and any level of maternal consumption of non-specific NSS during the periconceptional (p_{trend} = 0.35) or mid-pregnancy (p_{trend} = 0.44) periods; risk estimates range from 1.2 to 1.5. Hardell et al. (101) reported no association between brain cancer incidence and any low-calorie beverage consumption (OR = 1.70, 95% CI: 0.84-3.44).

Aspartame

Cabaniols et al. (100) reported no increased risk of malignant primitive brain tumors in individuals who regularly consumed aspartame (*i.e.*, ≥1/week) compared to those who consumed aspartame <1/week (OR = 1.02, 95% CI: 0.57-1.85). Gurney et al. (99) reported no association between aspartame consumption from whole diet or diet beverages during different potential windows of susceptibility (*i.e.*, childhood and maternal consumption) and childhood brain cancer. Risk estimates ranged from 0.6 to 1.6.

Supplemental Table M.1 NSS and Brain Cancer Cohort Study Results

Study	Reference Group	Outcome Type	Cancer Type	Statistical Analysis: Risk Metric	NSS	Group	Exposed Cases	Expected Cases or Exposed Non-Cases	Risk Estimate (95% CI)	p_{Trend}	Covariate Adjustment
Lim *et al*. (2006) (95)	No aspartame consumption	Inc	Gliomas	RR	Aspartame	**Intake Levels (mg/d)**					Age, sex, ethnicity, BMI, and history of diabetes
						>0-<100	83	–	0.99 (0.75-1.29)	0.05	
						100-<200	32		0.70 (0.48-1.03)		
						200-<400	16		0.66 (0.39-1.12)		
						≥400	22		0.73 (0.46-1.15)		
McCullough *et al*. (2022) (26)	Never consume ASBs	Mort	Brain	HR	Non-specific	**ASB Consumption**[a]					Age, sex, race/ethnicity, smoking, marital status, education, consumption of red and processed meat, fruits and vegetables, alcohol, and SSBs
						<1 drink/d	–	–	1.06 (0.96-1.18)	0.714	
						1 drink/d			0.99 (0.87-1.12)		
						2+ drink/d			0.96 (0.84-1.11)		
						Continuous (per 1 drink/d)			0.98 (0.95-1.02)	–	
						Men					
						<1 drink/d	–	–	1.02 (0.86-1.21)	0.910	
						1 drink/d			1.06 (0.87-1.29)		
						2+ drink/d			0.95 (0.77-1.18)		
						Continuous (per 1 drink/d)			0.99 (0.93-1.05)	–	
						Women					
						<1 drink/d	–	–	1.10 (0.96-1.26)	0.764	
						1 drink/d			0.94 (0.79-1.12)		
						2+ drink/d			0.98 (0.82-1.17)		
						Continuous (per 1 drink/d)			0.98 (0.93-1.04)	–	

Notes:

ASB = Artificially Sweetened Beverage; BMI = Body Mass Index; CI = Confidence Interval; d = Day; HR = Hazard Ratio; Inc = Incidence; mg = Milligrams; Mort = Mortality; NSS = Non-Sugar Sweetener; RR = Relative Risk; SSB = Sugar Sweetened Beverage; yr = Year.

– = Not Reported.

(a) In analyses by BMI status (*i.e*., normal, overweight, obese), overweight subjects consuming <1 drink/d had a statistically significantly elevated HR (1.19, 95% CI: 1.02-1.40). In analyses of never smokers (with or without controlling for BMI) and analyses of all subjects controlled for BMI, no results were statistically significant. Sensitivity analyses excluding the first 2 yrs of follow-up or with stratification by 10-yr follow-up time produced similar results (26).

Supplemental Table M.2 NSS and Brain Cancer Case-Control Study Results

Study	Outcome Type	Cancer Type	Statistical Analysis							Covariate Adjustment
			Risk Metric	NSS	Group	Exposed Cases	Exposed Controls	Risk Estimate (95% CI)	p_{Trend}	
Bunin *et al*. (2005) (98)	Inc	Medulloblastoma/ PNET	OR	Non-specific	**Diet Soda Intake**					Income level, mother's race, age of child at interview, date of interview, gained weight as a result of nausea/vomiting, number cigarettes/d, total calories, and regular soda intake
					Periconception					
					<1/mo	–	–	Ref	0.35	
					≥1/mo-1/d			1.2 (0.7-2.1)		
					≥2/d			1.3 (0.8-2.4)		
					Midpregnancy					
					<1/mo	–	–	Ref	0.44	
					≥1/mo-1/d			1.5 (0.8-2.6)		
					≥2/d			1.3 (0.7-2.5)		
Cabaniols *et al*. (2011) (100)	Inc	MPBT	OR	Aspartame	**Aspartame Intake Frequency**					Age and sex
					Non-consumers (<1/wk)	–	–	Ref	–	
					Regular consumers (≥1/wk)	30	30	1.02 (0.57-1.85)		
Gurney *et al*. (1997) (99)	Inc	Childhood Brain	OR	Aspartame	**All Dietary Sources**					Age at diagnosis or reference date, birth year, sex, and study site
					No consumption	–	–	Ref	–	
					Any consumption	17	26	1.1 (0.5-2.6)		
					Age at First Consumption (yrs)					
					<3	7	12	1.0 (0.3-3.1)	–	
					3-7	10	13	1.2 (0.4-3.6)		
					Yrs of Consumption					
					<2	9	14	1.2 (0.4-3.3)	–	
					≥2	8	11	1.1 (0.3-3.4)		
					Frequency of Consumption (Times/wk)					
					<1	7	8	1.6 (0.5-5.2)	–	
					≥1	10	18	0.9 (0.3-2.4)		
					**Diet Drinks**					
					No consumption	–	–	Ref	–	
					Any consumption	9	19	0.9 (0.3-2.4)		
					Age at First Consumption (yrs)					
					<3	4	8	0.8 (0.2-3.1)	–	
					3-8	5	10	1.0 (0.3-3.4)		
					Yrs of Consumption					
					<2	4	10	0.8 (0.2-3.1)	–	
					≥2	5	8	0.9 (0.3-3.4)		

Study	Outcome Type	Cancer Type	Statistical Analysis							
			Risk Metric	NSS	Group	Exposed Cases	Exposed Controls	Risk Estimate (95% CI)	p_{Trend}	Covariate Adjustment
					Frequency of Consumption (Times/wk)					
					<1	5	8	1.2 (0.3-4.5)	–	
					≥1	4	11	0.6 (0.2-2.3)		
					**Maternal Aspartame Consumption**					
					All Sources					
					No consumption	–	–	Ref	–	
					Any consumption	9	22	0.7 (0.3-1.7)		
					During pregnancy	7	19	0.6 (0.2-1.7)		
					1st trimester	6	18	0.6 (0.2-1.6)		
					2nd trimester	7	18	0.7 (0.3-1.8)		
					3rd trimester	6	18	0.6 (0.2-1.6)		
					While breastfeeding	5	14	0.7 (0.2-2.0)		
					Diet Drinks					
					No consumption	–	–	Ref	–	
					Any consumption	5	11	0.9 (0.3-2.8)		
					During pregnancy	3	9	0.7 (0.2-2.7)		
					1st trimester	3	9	0.7 (0.2-2.7)		
					2nd trimester	3	6	1.1 (0.3-5.1)		
					3rd trimester	2	7	0.6 (0.1-3.2)		
					While breastfeeding	4	8	1.1 (0.3-4.0)		
Hardell *et al.* (2001) (101)	Inc	Brain	OR	Non-specific	**Low Calorie Beverage Consumption**					Age, sex, and geographical area
					None	–	–	Ref	–	
					Any			1.70 (0.84-3.44)		

Notes:
CI = Confidence Interval; d = Day; Inc = Incidence; mo = Month; MPBT = Malignant Primitive Brain Tumors; NSS = Non-Sugar Sweetener; OR = Odds Ratio; PNET = Primitive Neuroectodermal Tumors; Ref = Reference; wk = Week; yr = Year.
– = Not Reported.

Supplement N: Lung Cancer

Cohort Studies

Non-specific NSS

Two cohort studies evaluated lung cancer and NSSs (19, 26). Both studies reported a decreased risk of lung cancer associated with NSS intake; most risk estimates were <1 and were statistically significant. McCullough et al. (26) reported a decreased risk of lung cancer mortality associated with all levels of ASB consumption compared to those who reported never consuming ASBs (<1 drink/day HR = 0.89, 95% CI: 0.86-0.93; 1 drink/day HR = 0.88, 95% CI: 0.84-0.92; ≥2 drinks/day HR = 0.87, 95% CI: 0.83-0.91; $p_{trend} < 0.0001$). Results were similar in analyses stratified by sex (men $p_{trend} < 0.0001$; women $p_{trend} < 0.0001$). You et al. (19) reported no association between diet soft drink consumption and lung cancer incidence in all participants (HR = 0.78, 95% CI: 0.61-0.99), males (HR = 1.08, 95% CI: 0.82-1.42), and never smokers (HR = 0.65, 95% CI: 0.36-1.18), and a decreased risk among women (HR = 0.78, 95% CI: 0.61-0.99) and ever/current smokers (HR = 0.82, 95% CI: 0.68-0.99).

Case-Control Studies

Non-specific NSS

Mettlin (102) reported a decreased risk of lung cancer for individuals who consumed 1 (RR = 0.45, 95% CI: 0.25-0.83) or ≥2 (RR = 0.48, 95% CI: 0.27-0.87) diet cola drinks/day compared to non-consumers, but no association for those who consumed <1/day (RR = 0.72, 95% CI: 0.50-1.05).

Supplemental Table N.1 NSS and Lung Cancer Cohort Study Results

Study	Reference Group	Outcome Type	Cancer Type	Statistical Analysis							
				Risk Metric	NSS	Group	Exposed Cases	Expected Cases or Exposed Non-Cases	Risk Estimate (95% CI)	p_{Trend}	Covariate Adjustment
McCullough *et al*. (2022) (26)	Never consume ASBs	Mort	Lung	HR	Non-specific	**ASB Consumption[a]**					Age, sex, race/ethnicity, smoking, marital status, education, consumption of red and processed meat, fruits and vegetables, alcohol, and SSBs
						<1 drink/d	–	–	**0.89 (0.86-0.93)**	**<0.0001**	
						1 drink/d			**0.88 (0.84-0.92)**		
						≥2 drinks/d			**0.87 (0.83-0.91)**		
						Continuous (per 1 drink/d)			**0.96 (0.95-0.97)**	–	
						Men					
						<1 drink/d	–	–	**0.91 (0.86-0.96)**	**<0.0001**	
						1 drink/d			**0.88 (0.82-0.95)**		
						≥2 drinks/d			**0.90 (0.84-0.96)**		
						Continuous (per 1 drink/d)			**0.97 (0.95-0.99)**	–	
						Women					
						<1 drink/d	–	–	**0.87 (0.83-0.92)**	**<0.0001**	
						1 drink/d			**0.86 (0.81-0.91)**		
						≥2 drinks/d			**0.82 (0.77-0.87)**		
						Continuous (per 1 drink/d)			**0.95 (0.93-0.96)**	–	
You *et al*. (2022) (19)	No soft drink consumption	Inc	Lung	HR	Non-specific	**Diet Soft Drink Consumption Only[b]**					Age, sex, race, study center, arm, total energy intake, alcohol consumption, smoking status, BMI categories, physical activity, education, red meat intake, amounts of fruits and vegetables, coffee, family history of lung cancer, and estrogen use (female subgroup only)
						Overall	487	35,070	0.89 (0.75-1.07)	–	
						Males	234	13,700	1.08 (0.82-1.42)		
						Females	487	35,070	**0.78 (0.61-0.99)**		
						Never smokers	37	16,098	0.65 (0.36-1.18)		
						Ever/current smokers	450	18,966	**0.82 (0.68-0.99)**		

Notes:
ASB = Artificially Sweetened Beverage; BMI = Body Mass Index; CI = Confidence Interval; d = Day; HR = Hazard Ratio; Inc = Incidence; Mort = Mortality; NSCLC = Non-Small Cell Lung Cancer; NSS = Non-Sugar Sweetener; SCLC = Small Cell Lung Cancer; SSB = Sugar-Sweetened Beverage; yr = Year.
– = Not Reported.
Bolded values indicate statistical significance.

(a) There were no associations in analyses of never smokers (with or without control for BMI). Results were not substantially different when analyses were controlled for BMI among all subjects. When analyses were stratified by BMI status (*i.e.*, normal, overweight, or obese), some results among those who were obese were no longer statistically significant (*i.e.*, <1 drink/d, 1 drink/d, and per 1 drink/d exposures). Sensitivity analyses excluding the first 2 yrs of follow-up or with stratification by 10-yr follow-up time also produced similar results (26).
(b) Results from cause-specific multivariable Cox regression models and competing risks Fine-Gray models were largely similar, though there was no longer an association between consumption of only diet soft drinks and lung cancer among ever/current smokers in competing risks models. In sensitivity analyses of NSCLC and SCLC, diet soft drink only consumption among never smokers was associated with a decreased SCLC risk (HR = 0.04, 95% CI: 0.00-0.35) (19).

Supplemental Table N.2 NSS and Lung Cancer Case-Control Study Results

Citation	Outcome Type	Cancer Type	Statistical Analysis							
			Risk Metric	NSS	Group	Exposed Cases	Exposed Controls	Risk Estimate (95% CI)	p_{Trend}	Covariate Adjustment
Mettlin (1989) (102)	Inc	Lung	RR	Non-specific	**Level of Diet Cola Consumption (Drinks/d)**					Age, sex, residence, smoking history, beta-carotene intake, and education level
					Never	452	287	Ref	–	
					<1	74	107	0.72 (0.50-1.05)		
					1	21	34	**0.45 (0.25-0.83)**		
					≥2	22	41	**0.48 (0.27-0.87)**		

Notes:
CI = Confidence Interval; d = Day; Inc = Incidence; NSS = Non-Sugar Sweetener; Ref = Reference; RR = Relative Risk.
– = Not Reported.
Bolded values indicate statistical significance.

References Cited in Supplements C, D, and/or E Not Cited in Main Manuscript

Fulgoni VL III, Drewnowski A. No association between low-calorie sweetener (LCS) use and overall cancer risk in the nationally representative database in the US: Analyses of NHANES 1988-2018 data and 2019 public-use linked mortality files. Nutrients 2022;14(23):4957. doi: 10.3390/nu14234957.

Gao Y, Yin L, Zhang Y, Li X, Liu L. Associations of saccharin intake with all-cause, cardiovascular and cancer mortality risk in USA adults. Br. J. Nutr. Epub 2024. doi: 10.1017/S0007114524002034.

National Cancer Institute (NCI) [Internet]. 2023 Mar 15 [cited 2024 November 4]. Diet history questionnaire: validation studies. Available from: https://epi.grants.cancer.gov/dhq/about/validation.html.

Singh N, Singh Lubana S, Arora S, Sachmechi I. A study of artificial sweeteners and thyroid cancer risk. J. Clin. Med. Res. 2020;12(8):492-498. doi: 10.14740/jocmr4258.

Zamora-Ros R, Cayssials V, Cleries R, Torrents M, Byrnes G, Weiderpass E, et al. Sweetened beverages are associated with a higher risk of differentiated thyroid cancer in the EPIC cohort: A dietary pattern approach. Eur. J. Nutr. 2023;62(1):105-114. doi: 10.1007/s00394-022-02953-5.

Zhang L, Ma C, Huang H, Li D, Zhang D, Wu T, et al. Association of unsweetened and sweetened cereal consumption with all-cause and cause-specific mortality: A large prospective population-based cohort study. Food Funct. 2024;15(19):10151-10162. doi: 10.1039/d4fo03761h.

Zhang YB, Chen JX, Jiang YW, Xia PF, Pan A. Association of sugar-sweetened beverage and artificially sweetened beverage intakes with mortality: An analysis of US National Health and Nutrition Examination Survey. Eur. J. Nutr. 2021;60(4):1945-1955. doi: 10.1007/s00394-020-02387-x.
